# Trends and projections of kidney cancer incidence at the global and national levels, 1990–2030: a Bayesian age-period-cohort modeling study

**DOI:** 10.1186/s40364-020-00195-3

**Published:** 2020-05-13

**Authors:** Zhebin Du, Wei Chen, Qier Xia, Oumin Shi, Qi Chen

**Affiliations:** 1 Department of Urology, Renji Hospital affiliated to Shanghai Jiaotong University School of Medicine, Shanghai, 200127 China; 2 Department of Urology, Pudong New Area People’s Hospital, Shanghai, China; 3 Health Science Center, Shenzhen Second People’s Hospital, The First Affiliated Hospital of Shenzhen University, Shenzhen, 518020 China

**Keywords:** Kidney cancer, Incidence, Prediction, Temporal trends, Modeling study

## Abstract

**Background:**

Identifying the temporal trends of kidney cancer (KC) incidence in both the past and the future at the global and national levels is critical for KC prevention.

**Methods:**

We retrieved annual KC case data between 1990 and 2017 from the Global Burden of Disease (GBD) online database. The average annual percentage change (AAPC) was used to quantify the temporal trends of KC age-standardized incidence rates (ASRs) from 1990 to 2017. Bayesian age-period-cohort models were used to predict KC incidence through 2030.

**Results:**

Worldwide, the number of newly diagnosed KC cases increased from 207.3 thousand in 1990 to 393.0 thousand in 2017. The KC ASR increased from 4.72 per 100,000 to 4.94 per 100,000 during the same period. Between 2018 and 2030, the number of KC cases is projected to increase further to 475.4 thousand (95% highest density interval [HDI] 423.9, 526.9). The KC ASR is predicted to decrease slightly to 4.46 per 100,000 (95% HDI 4.06, 4.86). A total of 90, 2, and 80 countries or territories are projected to experience increases, remain stable, and experience decreases in KC ASR between 2018 and 2030, respectively. In most developed countries, the KC incidence is forecasted to decrease irrespective of past trends. In most developing countries, the KC incidence is predicted to increase persistently through 2030.

**Conclusions:**

KC incidence is predicted to decrease in the next decade, and this predicted decrease is mainly driven by the decreases in developed countries. More attention should be placed on developing countries.

## Introduction

Kidney cancer (KC) develops from the renal parenchyma, and approximately 70% of KC cases are clear renal cell carcinomas [1]. According to the latest statistics, there were more than 400 thousand newly diagnosed KC cases and nearly 180 thousand KC-related deaths in 2018 [2]. The KC incidence is highly heterogeneous worldwide, with North America having the highest incidence, followed by Western Europe and Australia [3]. In South America, Africa, and Asia, the KC incidences are relatively low [4, 5]. Within continents, KC incidence rates also differ by country. Across Europe, the incidence ranged more than fourfold: from 4.5 per 100,000 in Albania to approximately 16.8 per 100,000 in the Belarus [6]. Additionally, the temporal trends of KC incidence vary worldwide [7]. For example, in the USA, the rate was 8.0/100,000 in males in 1975 and increased steadily to 13.4/100,000 in 2012. In contrast, Austria and Poland have reported significantly decreasing rates since the early 2000s [7].

Many factors, including lifestyle changes, exposure to risk factors, and expanding coverage of tumor detection and reporting, have contributed to the temporal trends of KC incidence. Incidence trends can serve as a good indicator of shifting disease patterns and changing risk factors within a population [8] and are of importance for KC prevention. More importantly, since the marked alterations in risk factors over the last decades [9, 10], KC incidence might be subsequently changed in the near future. Further knowledge of the future trends of KC incidence is therefore critical for understanding and planning in regard to this disease burden and permits the modification of the national health system to respond to future challenges. Previous studies have described KC incidence, but these studies were retrospective in nature and consequently lacked insight into the future KC burden [5, 11–13]. Additionally, the number of cancer cases or deaths is the total number of people within a population who have either been diagnosed with or die from cancer, and this is greatly influenced by the size and age composition of the population. This information is critical to understanding and planning for the disease burden. To address this limitation, we used a Bayesian age-period-cohort (APC) model on KC incidence at the global and nation levels between 1990 and 2017 to project both the future number of cancer cases and incidence through 2030. Our predictions are of importance for the re-allocation of limited medical resources and to update the prevention strategies for KC.

## Materials and methods

### Study data

We collected annual KC case data between 1990 and 2017 by sex, region (195 countries or territories), age (from under 5 to ≥80 years in 5-year intervals) from the Global Burden of Disease (GBD) online query tool [14]. The general procedures for data collection and processing in the GBD study have been detailed and validated elsewhere [15, 16]. In brief, the annual number of newly diagnosed KC cases was sought from individual cancer registries or aggregated databases of cancer registry data such as “Cancer Incidence in Five Continents (CI5)”, EUREG, SEER, or NORDCAN. The ICD-10 codes (C62-C62.92, Z80.43, and Z85.47-Z85.48) and ICD-9 codes (186–186.9, V10.47-V10.48, and V16.43) were used to identify KC cases [15]. The national sociodemographic index (SDI), a composite index measuring average achievement in several basic dimensions of country development, was collected from the GBD database. We also retrieved the corresponding population data for each country or territory by year (1990–2030), sex, and age (from under 5 to ≥80 years in 5-year intervals) from the United Nations Department of Economics and Social Affairs (DESA) Population Division. Only 185 countries or territories were available at population data.

### Statistical analysis

#### Model selection

**Fig. 1:**
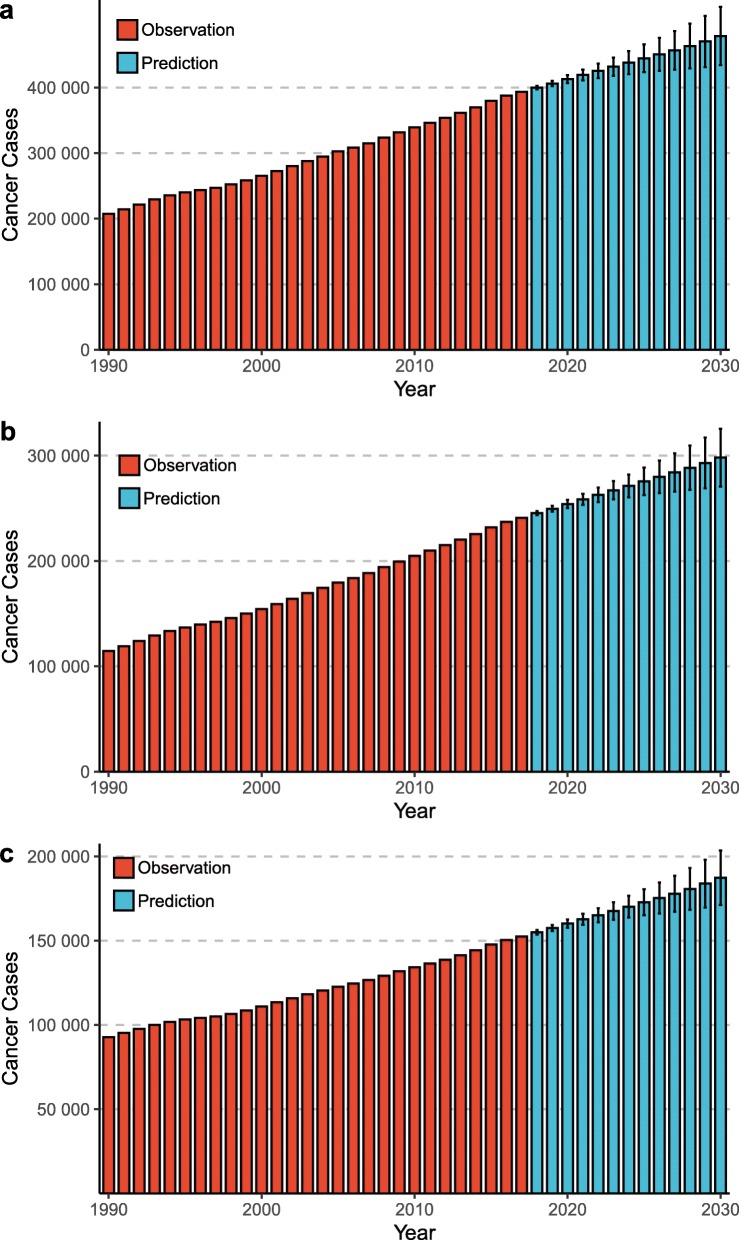
The increasing trends in the numbers of kidney cancer cases between 1990 and 2030 at the global level by sex (**a**, both sexes; **b**, male; **c**, female). The error bar denotes the 95% highest density interval (HDI) of the prediction values

Several models, including the Joinpoint model, age-period-cohort (APC) model, Nordpred model, and Bayesian APC model, have been previously used to predict cancer incidence based on population data [17–20]. We first conducted a model selection in terms of model prediction performance. KC case data from the USA, France, Brazil, Indonesia, and Vietnam, in which the KC incidence ranged from 2.5 per 100,000 to 13.0 per 100,000, were retrieved. These case data were then split into two intervals (1990–2012 and 2013–2017). We used the data between 1990 and 2012 to train the five prediction models (i.e., APC, Bayesian APC, Nordpred, nature-spline, and Joinpoint). KC incidence data between 2013 and 2017 were predicted and compared with the observational values in the same period. The prediction error rate was applied to assess the model performance. The error rate was calculated as $\left(\hat{y}-\mathrm{y}\right)/\mathrm{y}$, where $\hat{y}$ and y denote the prediction values and the observational values, respectively. The results of model selection are shown in S-Figure 1. Because of the relatively lower error rate of the Bayesian APC model, we used it to predict the KC cases and incidence rates through 2030.

The rationalities of the Bayesian APC model have been previously described [21]. Briefly, since the expectation that effects adjacent in time might be similar, the second-order random walk (RW2) model with inverse-gamma prior distribution was used for age, period and cohort effects. RW2 assumes an independent mean-zero normal distribution of the second differences of all time effects. This is a natural target for smoothing since the second differences in APC models are identifiable. Consider the age effects, for which the RW2 prior is identified as follows:

$$ f\left(a\left|{\kappa}_a\right.\right)\infty {\kappa}_a\frac{I-2}{2}\exp \left(-\frac{\kappa_a}{2}\sum \limits_{i=3}^I{\left({a}_i-2{a}_{i-1}+{a}_{i-2}\right)}^2\right)={\kappa}_a\frac{I-2}{2}\exp \left(-\frac{1}{2}{a}^T Qa\right) $$

$$ Q={\kappa}_a\left[\begin{array}{l}1\kern1em 2\kern1em 1\\ {}-2\kern1em 5\kern1em -4\kern1em 1\\ {}1\kern1em -4\kern1em 6\kern1em -4\kern1em 1\\ {}\kern1em \mathrm{O}\kern1em \mathrm{O}\kern1em \mathrm{O}\kern1em \mathrm{O}\kern1em \mathrm{O}\\ {}\kern2em 1\kern1em -4\kern1em 6\kern1em -4\kern1em 1\\ {}\kern4em 1\kern1em -4\kern1em 5\kern1em -2\\ {}\kern6em 1\kern1em -2\kern1em 1\end{array}\right] $$

where *i* denotes the age index that ranges from 1 to *I* = 17 in this study, because we projected the cancer incidence of people aged 0 to 84, and age was divided into 17 groups. Moreover, *κ*_*α*_^− 1^ denotes the variance parameter. Note that *Q* is rank deficient. To complete the RW2 model specification, we use the usual conjugate hyperprior for precision, *κ*_*α*_ *~ Gamma(α, λ).* This leads to the full conditional *κ*_*α*_*|α ~ Gamma(α + 0.5 rank(Q), λ + 0.5α’Qα)*, which may be directly simulated [20]*.* In this study, we used the parameter values *α =* 0.5, 1, and 1 and *λ =* 0.0005, 0.00005, and 0.00005 for age, period, and cohort effects, respectively. The World-2000 population was used to standardize the KC incidence rates. To ensure the smoothness of predictions, countries or territories that experienced a striking fluctuation in KC case numbers within a small time interval were excluded. A total of 172 countries or territories were finally included.

### Quantifying the KC incidence trends

The average annual percentage change (AAPC) was used to quantify the temporal trends of KC age-standardized incidence rates (ASRs) in 1990–2017 and 2018–2030, which indicate the past trends and future trends, respectively. A regression line was fitted to the natural logarithm of the rates, i.e., *y = α + βx + ɛ,* where *y =* ln (ASR) and *x* = calendar year, and the AAPC was calculated as 100 × (*exp*(*β*)-1) [22]. To overcome over dispersion, the AAPC of 2018–2030 was calculated with the inverse of the standardized error (i.e., 1/*se*) of the estimated incidence rate as the weights in the regression models [20].

### Sensitivity analysis

Because the KC case data in the GBD database were estimates from surveillance data instead of the surveillance data itself [23], we conducted a sensitivity analysis to verify the robustness of the prediction results derived from our models. Herein, we collected the KC case data from the Cancer Incidence in Five Continents *plus* (*CI5p*) database. Bayesian APC model was used to predict the KC cases and incidence rates based on the surveillance data from *CI5p* database [24]. Cancer surveillance data that covering more population and having a longer time span were preferable. Finally, data from Australia (from 1993 to 2012 and covering 7 cancer registries), Spain (from 1993 to 2010 and covering9 cancer registries), France (from 1998 to 2010 and covering 9 cancer registries), Italy (from 1998 to 2010 and covering 8 cancer registries), and the USA (from 1990 to 2012 and covering 9 cancer registries) were used. All statistical analyses were conducted in the R program (R core team, V3.5.1). A *P* value less than 0.05 was deemed statistically significant.

## Results

### KC case numbers and incidence, 1990–2017

**Fig. 2:**
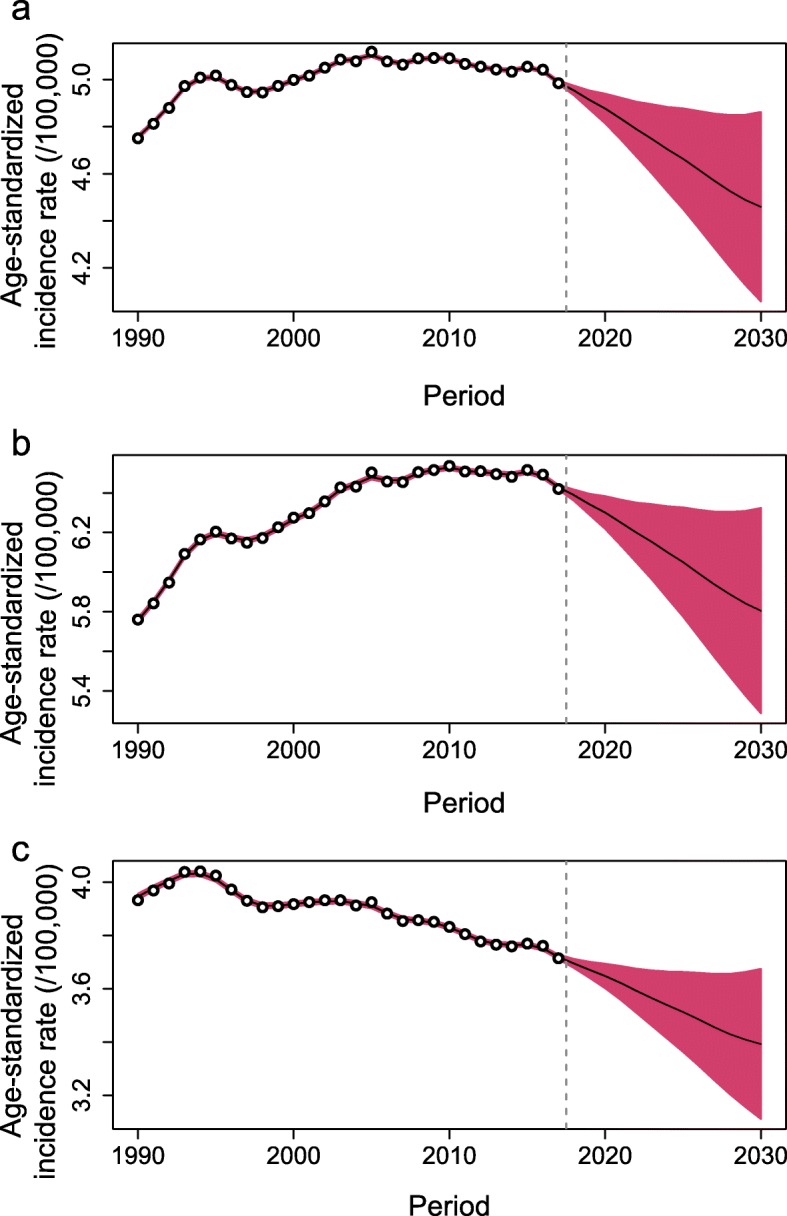
The temporal trends of age-standardized incidence rates (ASRs, per 100,000) of kidney cancer between 1990 and 2030 at the global level in both sexes (**a**), males (**b**), and females (**c**). The open dots represent the observational values from GBD dataset, and the brick red shadow denotes the 95% highest density interval of prediction values. The predictive mean value is shown as a black solid line. The vertical dashed line indicates where the prediction starts

**Fig. 3:**
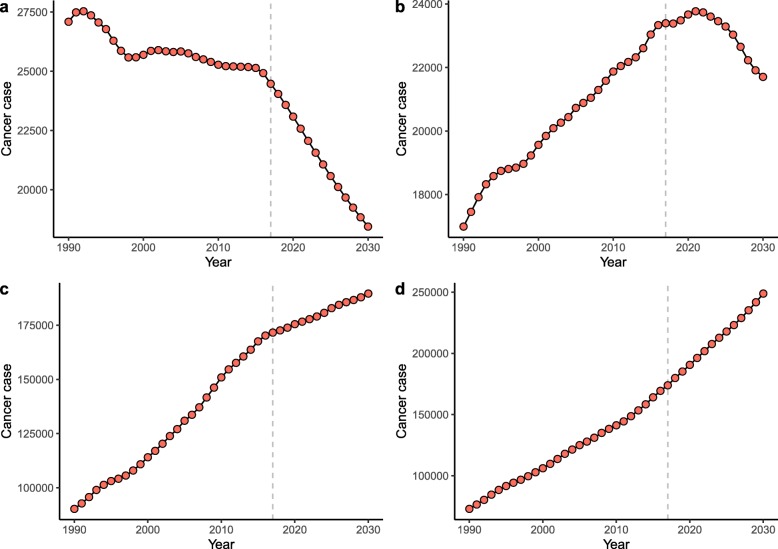
The changing trends in the number of kidney cancer cases between 1990 and 2030 by age (**a**, 0–19 years; **b**, 20–39 years; **c**, 40–64 years; **d**, ≥65 years). The vertical dashed line indicates where the prediction starts

**Fig. 4:**
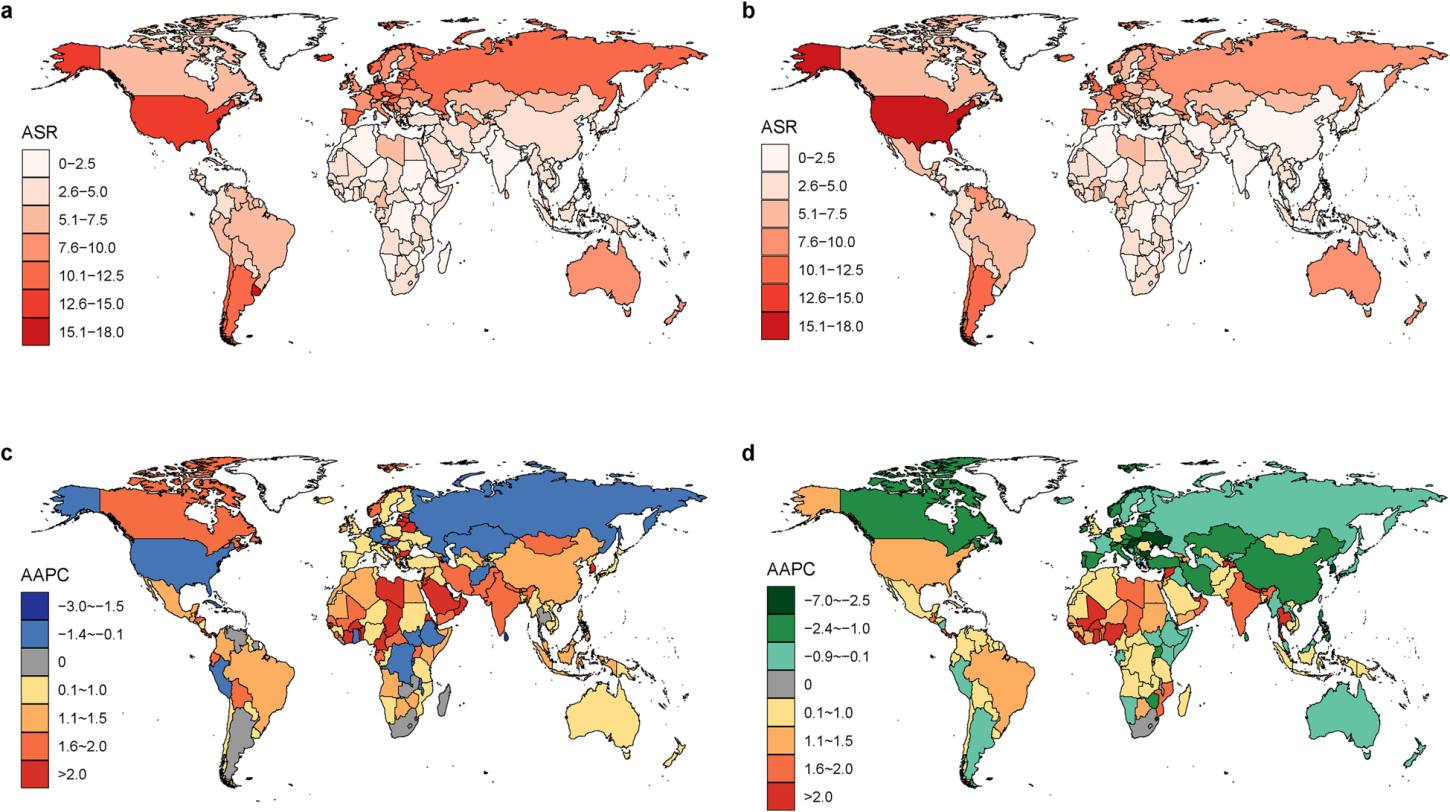
The global distribution and the average annual percentage changes (AAPCs) in age-standardized incidence rates (ASRs, per 100,000) of kidney cancer at the national level. (**a** ASR of kidney cancer in 2017; **b** ASR of kidney cancer in 2030; **c** AAPC of kidney cancer ASR between 1990 and 2017; **d** AAPC of kidney cancer ASR between 2018 and 2030)

Worldwide, the number of newly diagnosed KC cases increased from 207.3 thousand in 1990 to 393.0 thousand in 2017, and the KC ASR increased from 4.72 per 100,000 to 4.94 per 100,000 during the same period (AAPC = 0.14, 95% confidence interval [CI] 0.08, 0.20) (Table 1; Figs. 1 and 2). The case numbers increased in both sexes (Table 1; Fig. 1). The ASR increased significantly among males (AAPC = 0.38, 95% CI, 0.30, 0.46). In contrast, a significant decrease in ASR was detected among females (AAPC = − 0.26, 95% CI -0.30, − 0.23). The KC case numbers increased in all age groups, with the exception of people aged 0–19 years (Table 1; Fig. 3). The most pronounced increase was found in older people (≥ 65 years), among whom the case number increased by more than 100 thousand between 1990 and 2017. At the national level, the highest KC ASR was found in Uruguay (16.15 per 100,000), followed by Slovakia, Iceland, and the Czech Republic in 2017 (Fig. 4a). During the study period, a total of 134, 8, and 30 countries or territories experienced increases, remained stable, and experienced decreases in KC ASR, respectively (Fig. 4c; S-Table 1). The greatest increase was detected in Armenia (AAPC = 6.24, 95% CI 5.12, 7.36), followed by Bulgaria and Belarus (Fig. 4c; S-Table 1). The most pronounced decrease was found in Sri Lanka (AAPC = − 2.71, 95% CI -3.85, − 1.56), followed by Trinidad and Tobago and Qatar (Fig. 4c; S-Table 1).

**Table 1:** The case numbers and incidence rates of kidney cancer between 1990 and 2030 at the global level

	1990		2017		2030		AAPC (95% CI) of ASR	
	No. of cases (× 1000)	ASR (/100,000)	No. of cases (×1000)	ASR (/100,000)	No. of cases (×1000) (95% HDI)	ASR (/100,000) (95% HDI)	1990–2017	2018–2030
Overall	207.3	4.72	393.0	4.94	475.4 (423.9, 526.9)	4.46 (4.06, 4.86)	0.14 (0.08, 0.20)*	−0.97 (−0.99, −0.95) *
Sex								
Male	114.6	5.65	240.8	6.38	298.1 (270.7, 325.6)	5.81 (5.29, 6.32)	0.38 (0.30, 0.46)*	−0.82 (−0.84, − 0.80)*
Female	92.7	3.96	152.3	3.68	187.4 (171.3, 203.4)	3.39 (3.11, 3.67)	−0.26 (− 0.30, − 0.23)*	−0.73 (− 0.75, − 0.72)*
Age years^a^								
0–19	27.0	–	24.4	–	18.4 (15.5, 21.4)	–	–	–
20–39	17.0	–	23.3	–	21.7 (19.7, 23.7)	–	–	–
40–64	90.3	–	171.5	–	189.6 (172.5, 206.7)	–	–	–
65+	73.0	–	173.8	–	248.8 (226.4, 271.3)	–	–	–

*ASR* Age-standardized incidence rate, *AAPC* Average annual percentage change, *CI* Confidence interval, *HDI* Highest density interval

^a^, for each age group, only the number of cancer cases is shown because the ASR was not available when the age was grouped

*, *P* < 0.001

### KC case numbers and incidence, 2018–2030

Between 2018 and 2030, the KC case number will further increase to 475.4 thousand (95% highest density interval [HDI] 423.9, 526.9) (Table 1; Fig. 1). The KC ASR will decrease slightly to 4.46 per 100,000 (95% HDI 4.06, 4.86) during the same period (AAPC = − 0.97, 95% CI -0.99, − 0.95) (Table 1; Fig. 2). A decreasing trend is expected for both sexes, although the case numbers will still increase (Table 1; Fig. 2). The case numbers are predicted to decrease for people aged 0–19 years and 20–39 years between 2018 and 2030. However, a persistent increase is expected for people aged 40–64 years and ≥ 65 years (Table 1; Fig. 3). S-Tables 2 and 3 show the predicted KC case numbers and ASRs at the national level. Briefly, the case numbers will increase in all 172 countries or territories from 2018 to 2030. The temporal trends of KC ASR varied from country to country. In 2030, the highest KC ASR will be found in Uruguay (17.71 per 100,000), followed by the USA and Iceland (Fig. 4b; S-Table 3). A total of 90, 2, and 80 countries or territories will experience increases, remain stable, and experience decreases in KC ASR between 2018 and 2030 (Fig. 4d; S-Table 1). The greatest increase is expected in the United Arab Emirates (AAPC = 3.68, 95% 3.63, 3.73), followed by Burkina Faso and Ghana. The most pronounced decrease is expected in Ukraine (AAPC = − 6.62, 95% CI -6.65, − 6.58), followed by Croatia and Slovakia (Fig. 4d; S-Table 1).

### Correlations between past trends and future trends of KC incidence

**Fig. 5:**
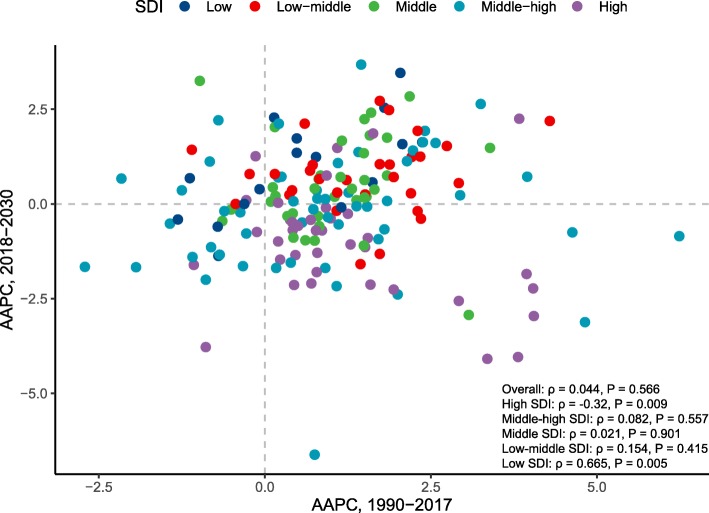
The correlations between the average annual percentage changes (AAPCs) in kidney cancer incidence in 1990–2017 and that in 2018–2030 at the national level, by sociodemographic index (SDI). The ρ and *P* values were derived from Pearson correlation tests

Between 1990 and 2030, 18 and 72 countries or territories experienced a continuous decrease and increase in KC ASR, respectively. Ten countries or territories experienced a decrease in the past but will experience an unfavorable increase in the future. For example, we found that the decreasing trend of KC ASR will be reversed in the USA after 2017. In contrast, a total of 61 countries or territories will experience a significant decrease in KC ASR in the future despite the past increases in these regions. Figure 5 displays the correlations between past trends and future trends of KC ASR. No significant association was found when taking all countries into consideration as a whole (ρ = 0.044, *P* = 0.566). However, a significant negative association was detected for countries with a high SDI (ρ = − 0.320, *P* = 0.009), which means that most developed countries will undergo a favorable decrease in KC ASR between 2018 and 2030. In contrast, for countries with low SDI, a significant positive association was found (ρ = 0.665, *P* = 0.005), which means that past trends will remain in the future in most countries.

The results of the sensitivity analysis are shown in S-Figure 2. Generally, the predictions based on the GBD data and *CI5plus* data were comparable in all five countries. The predicted trends of KC ASR based on GBD data were similar to these based on *CI5plus* data, although the ASR values differed to some extent. These disparities were mainly ascribed to the differences in population coverage rate between these two databases.

## Discussion

Kidney cancer (KC) is a malignancy whose incidence varies widely worldwide. Although KC incidence is relatively low compared to bladder and prostate cancer incidence rates, KC is of particular relevance in certain regions, such as Europe and North America, because of locally high incidence rates and significantly increasing rates in most countries in recent decades [5, 13, 15]. In the current study, we used GBD data to both describe the temporal trends of KC incidence over the last three decades and predicted its future trends in the next decade at the global and national levels. Globally, the number of KC cases is expected to increase consistently from 1990 through 2030, whereas the KC ASR is expected to decrease after 2017. The future decreasing trend was consistent in both sexes and in approximately half of all countries or territories. Of note, more than half of countries or territories, particularly developing regions, are expected to experience a significant increase in KC ASR between 2018 and 2030. These unfavorable trends might constitute a major obstacle for KC management and prevention in the near future.

The established risk factors, both environmental and genetic, for KC have been widely investigated and well documented [4, 25]. The impact of smoking on KC risk is modest, with an approximate 30% increased risk in current smokers and a 15% increased risk in former smokers compared with the risk among never smokers [26]. In developed countries, it is estimated that 6 and 24% of kidney cancer deaths are a result of tobacco smoking among females and males, respectively [13]. Fortunately, these proportions were shown to have decreased in the last decade, which was mainly ascribed to the “smoke-free” campaign in these countries [27, 28]. In contrast, overweight or obesity, another established risk factor for KC, has increased strikingly over the past four decades [29, 30]. Moreover, the global adult per-capita alcohol consumption increased from 5.9 L to 6.5 L and is forecasted to reach 7.6 L by 2030 [31]. These alarming increases might drive an unexpected increase in KC incidence rates worldwide. For example, the KC incidence experienced an unfavorable reversal in the USA after 2017, despite the prior decrease. Additionally, the KC incidence trend was also predicted to be increasing in both the UK and Germany, whereas the incidence trend in the surrounding countries was decreasing. We speculated that this increase might be attributed to the following reasons: 1) the dramatic increases in overweight and obesity and alcohol use [32, 33]; 2) immigrants from Africa and Asia might contribute to some extent [34]; and 3) the increase among blacks, especially in the USA, might surpass the decrease among whites [7]. The underlying causes need further investigation, and the unexpected increase indicates that KC remains a hard-to-ignore health concern in those highly developed countries.

For most countries in Europe and Australia, we observed a favorable decrease in KC incidence after 2017, which might largely drive the global declining trend. The rising incidence of KC over the last decades in Western populations has been attributed to the increased use of imaging techniques, which can result in incidental findings of small renal masses and has been reported to contribute as much as 50% to the overall incidence [7, 35]. The declining trends therefore might be ascribed to not only the reduction in risk factors but also to the plateau of imaging utilization. Although a relatively low incidence rate was observed, a consistent increase was observed in most countries in Latin America, Africa, South Asia, and Southeast Asia from 1990 to 2017. This increase was predicted to remain through 2030. We speculated that this increase might be partly explained by the following causes: 1) the increasing KC detection rates and reporting rates [7]; 2) the growing population, particularly the aging population [20]; 3) shifting trend toward the adoption of Western diets, change in occupational patterns, increased high-risk behaviors (e.g., excessive calorie intake and physical inactivity), and changes in established cancer risk factors (e.g., smoking and obesity) [20, 36]; and 4) the increasing prevalence of chronic kidney diseases [37, 38]. Given the persistent increase, KC might be one of the main public health concerns in the near future in countries that previously had a lighter disease burden.

Our study has limitations. First, the GBD data were estimates from mathematical models based on surveillance data rather than surveillance data itself. However, the GBD study provides global-scale data and offers us an unprecedented opportunity to explore the global disease burden. Additionally, to ensure the robustness of our predictive results, we conducted sensitivity analyses based on observations from the *CI5plus* database. Whereas only five countries were included to validate the prediction values because of the limited data availability. This incomplete validation might limit the clinical value of our study. Second, the temporal trends of KC incidence in both the past and the future might be partly influenced by the detection and reporting rates, which reflect the quality of cancer registry data for each country. Cancer registry data can be biased in multiple ways. For example, changes between coding systems can lead to artificial differences in disease estimates; however, this bias has been adjusted by mapping the different coding systems to the GBD causes. Misclassification of metastatic sites as primary cancer can lead to overestimation of cancer sites that are common sites for metastases such as the brain or liver. Third, the dearth of histological information of KC in the GBD database prevented us from pinpointing the KC incidence trends by histological subtype. Despite these limitations, using the most up-to-date data and advanced modeling strategies, our study provides a comprehensive understanding of KC incidence from the past to the future.

## Conclusions

In summary, the KC incidence was predicted to decrease in the next decade. However, both the past and the future trends of KC incidence were highly heterogeneous from country to country. In most developed countries, the KC incidence is forecasted to decrease irrespective of past trends. In most developing countries, the KC incidence is expected to increase persistently through 2030. The long-term best practice approach must include the primary prevention of smoking and obesity, alongside careful monitoring of trends using high-quality population-based cancer registries and corresponding national registration sources.

## Supplementary information

**Additional file 1: Figure S1.** The prediction error rate of five models based on data from Brazil, France, Indonesia, USA, and Vietnam. **Figure S2.** The predictions of kidney cancer incidence based on GBD data and IARC data (CI5 plus database). (IARC data: Australia, 1993–2012; Spain, 1993–2010; France, 1998–2010; Italy, 1998–2010; USA, 1990–2012). The blue triangles were point estimates of kidney cancer incidence based on CI5p data. The white open dots were point estimates of kidney cancer incidence based on GBD data.

**Additional file 2: Table S1.** The AAPC of kidney cancer incidence in 1990–2017 and 2018–2030.

**Additional file 3: Table S2.** The predicting kidney cancer cases at the national level from 2018 to 2030.

**Additional file 4: Table S3.** The age-standardized incidence rate of kidney cancer at the national level from 1990 to 2030.

## Data Availability

The datasets supporting the conclusions of this article are included within the article.

## Abbreviations

KC: Kidney cancer
AAPC: Average annual percentage change
ASR: Age-standardized incidence rate

## References

[1] Gansler T, Fedewa S, Amin MB, Lin CC, Jemal A (2018). Trends in reporting histological subtyping of renal cell carcinoma: association with cancer center type. Hum Pathol.

[2] Bray F, Ferlay J, Soerjomataram I, Siegel RL, Torre LA, Jemal A (2018). Global cancer statistics 2018: GLOBOCAN estimates of incidence and mortality worldwide for 36 cancers in 185 countries. CA Cancer J Clin.

[3] Capitanio U, Bensalah K, Bex A, Boorjian SA, Bray F, Coleman J (2019). Epidemiology of renal cell carcinoma. Eur Urol.

[4] Scelo G, Larose TL. Epidemiology and risk factors for kidney cancer. J Clin Oncol. 2018;36(36):JCO2018791905. 10.1200/JCO.2018.79.1905 PMC6299342 30372394

[5] Li P, Znaor A, Holcatova I, Fabianova E, Mates D, Wozniak MB (2015). Regional geographic variations in kidney cancer incidence rates in European countries. Eur Urol.

[6] International Angency for Research on Cancer (2018). GLOBOCAN.

[7] Znaor A, Lortet-Tieulent J, Laversanne M, Jemal A, Bray F (2015). International variations and trends in renal cell carcinoma incidence and mortality. Eur Urol.

[8] Smittenaar CR, Petersen KA, Stewart K, Moitt N (2016). Cancer incidence and mortality projections in the UK until 2035. Br J Cancer.

[9] GBD 2016 Alcohol Collaborators. Alcohol use and burden for 195 countries and territories, 1990–2016: a systematic analysis for the global burden of disease study 2016. Lancet. 2018;392:1015–35. 10.1016/S0140-6736(18)31310-2 PMC6148333 30146330

[10] NCD Risk Factor Collaboration (NCD-RisC). Worldwide trends in body-mass index, underweight, overweight, and obesity from 1975 to 2016: a pooled analysis of 2416 population-based measurement studies in 128.9 million children, adolescents, and adults. Lancet. 2017;390:2627–42. 10.1016/S0140-6736(17)32129-3 PMC5735219 29029897

[11] van de Schans SA, Aben KK, Mulders PF, Haanen JB, van Herpen C, Verhoeven RH (2012). Modest improvement in 20 years of kidney cancer care in the Netherlands. Eur J Cancer.

[12] Innos K, Sepp T, Baburin A, Kotsar A, Lang K, Padrik P (2019). Increasing kidney cancer incidence and survival in Estonia: role of age and stage. Acta Oncol.

[13] Dy GW, Gore JL, Forouzanfar MH, Naghavi M, Fitzmaurice C (2017). Global burden of urologic cancers, 1990-2013. Eur Urol.

[14] Global Burden of Disease Collaborative Network (2018). Global burden of disease study 2017 (GBD 2017) results.

[15] Fitzmaurice C, Abate D, Abbasi N, Abbastabar H, Abd-Allah F, Abdel-Rahman O, et al. Global, regional, and National Cancer Incidence, mortality, years of life lost, years lived with disability, and disability-adjusted life-years for 29 cancer groups, 1990 to 2017: a systematic analysis for the global burden of disease study. JAMA Oncol. 2019;5(12):1749–68. 10.1001/jamaoncol.2019.2996 PMC6777271 31560378

[16] GBD 2017 Disease and Injury Incidence and Prevalence Collaborators. Global, regional, and national incidence, prevalence, and years lived with disability for 354 diseases and injuries for 195 countries and territories, 1990–2017: a systematic analysis for the global burden of disease study 2017. Lancet. 2018;392:1789–858. 10.1016/S0140-6736(18)32279-7 PMC6227754 30496104

[17] Jung KW, Won YJ, Kong HJ, Lee ES (2019). Prediction of cancer incidence and mortality in Korea, 2019. Cancer Res Treat.

[18] Wu J, Yang S, Xu K, Ding C, Zhou Y, Fu X (2018). Patterns and trends of liver cancer incidence rates in eastern and southeastern Asian countries (1983–2007) and predictions to 2030. Gastroenterology.

[19] Soares SCM, Dos Santos KMR, de Morais Fernandes FCG, Barbosa IR, de Souza DLB (2019). Testicular cancer mortality in Brazil: trends and predictions until 2030. BMC Urol.

[20] Liu Z, Jiang Y, Fang Q, Yuan H, Cai N, Suo C (2019). Future of cancer incidence in Shanghai, China: predicting the burden upon the ageing population. Cancer Epidemiol.

[21] Riebler A, Held L (2017). Projecting the future burden of cancer: Bayesian age-period-cohort analysis with integrated nested Laplace approximations. Biom J.

[22] Gao S, Yang WS, Bray F, Va P, Zhang W, Gao J (2012). Declining rates of hepatocellular carcinoma in urban Shanghai: incidence trends in 1976-2005. Eur J Epidemiol.

[23] Akinyemiju T, Abera S, Ahmed M, Alam N, Alemayohu MA, Allen C (2017). The burden of primary liver cancer and underlying etiologies from 1990 to 2015 at the global, regional, and National Level: results from the global burden of disease study 2015. JAMA Oncol.

[24] Bray F, Colombet M, Mery L, Piñeros M, Znaor A, Zanetti R (2017). Cancer incidence in five continents, Vol. XI (electronic version).

[25] Schmidt LS, Linehan WM (2016). Genetic predisposition to kidney cancer. Semin Oncol.

[26] Cumberbatch MG, Rota M, Catto JW, La Vecchia C (2016). The role of tobacco smoke in bladder and kidney carcinogenesis: a comparison of exposures and meta-analysis of incidence and mortality risks. Eur Urol.

[27] Bayer R, Bachynski KE (2013). Banning smoking in parks and on beaches: science, policy, and the politics of denormalization. Health Aff (Millwood).

[28] Currie LM, Clancy L (2011). The road to smoke-free legislation in Ireland. Addiction..

[29] Gortmaker SL, Swinburn BA, Levy D, Carter R, Mabry PL, Finegood DT (2011). Changing the future of obesity: science, policy, and action. Lancet..

[30] Swinburn BA, Kraak VI, Allender S, Atkins VJ, Baker PI, Bogard JR (2019). The global Syndemic of obesity, undernutrition, and climate change: the lancet commission report. Lancet..

[31] Manthey J, Shield KD, Rylett M, Hasan OSM, Probst C, Rehm J. Global alcohol exposure between 1990 and 2017 and forecasts until 2030: a modelling study. Lancet. 2019. 10.1016/S0140-6736(18)32744-2 31076174

[32] Sung H, Siegel RL, Torre LA, Pearson-Stuttard J, Islami F, Fedewa SA (2019). Global patterns in excess body weight and the associated cancer burden. CA Cancer J Clin.

[33] Bhaskaran K, Douglas I, Forbes H, dos Santos Silva I, Leon DA, Smeeth L (2014). Body-mass index and risk of 22 specific cancers: a population-based cohort study of 5.24 million UK adults. Lancet.

[34] Lipworth L, Tarone RE, McLaughlin JK (2011). Renal cell cancer among African Americans: an epidemiologic review. BMC Cancer.

[35] Sun M, Thuret R, Abdollah F, Lughezzani G, Schmitges J, Tian Z (2011). Age-adjusted incidence, mortality, and survival rates of stage-specific renal cell carcinoma in North America: a trend analysis. Eur Urol.

[36] Chow WH, Dong LM, Devesa SS (2010). Epidemiology and risk factors for kidney cancer. Nat Rev Urol.

[37] Hasan M, Sutradhar I, Gupta RD, Sarker M (2018). Prevalence of chronic kidney disease in South Asia: a systematic review. BMC Nephrol.

[38] Abd ElHafeez S, Bolignano D, D'Arrigo G, Dounousi E, Tripepi G, Zoccali C (2018). Prevalence and burden of chronic kidney disease among the general population and high-risk groups in Africa: a systematic review. BMJ Open.

