# Supplementary material for: Trends and projections of kidney cancer incidence at the global and national levels, 1990–2030: a Bayesian age-period-cohort modeling study
Source: Biomark Res. 2020 May 13;8:16. doi: 10.1186/s40364-020-00195-3 (PMC7222434; doi:10.1186/s40364-020-00195-3)

S-Figure 1. The prediction error rate of five models based on data from Brazil, France, Indonesia, USA, and Vietnam.


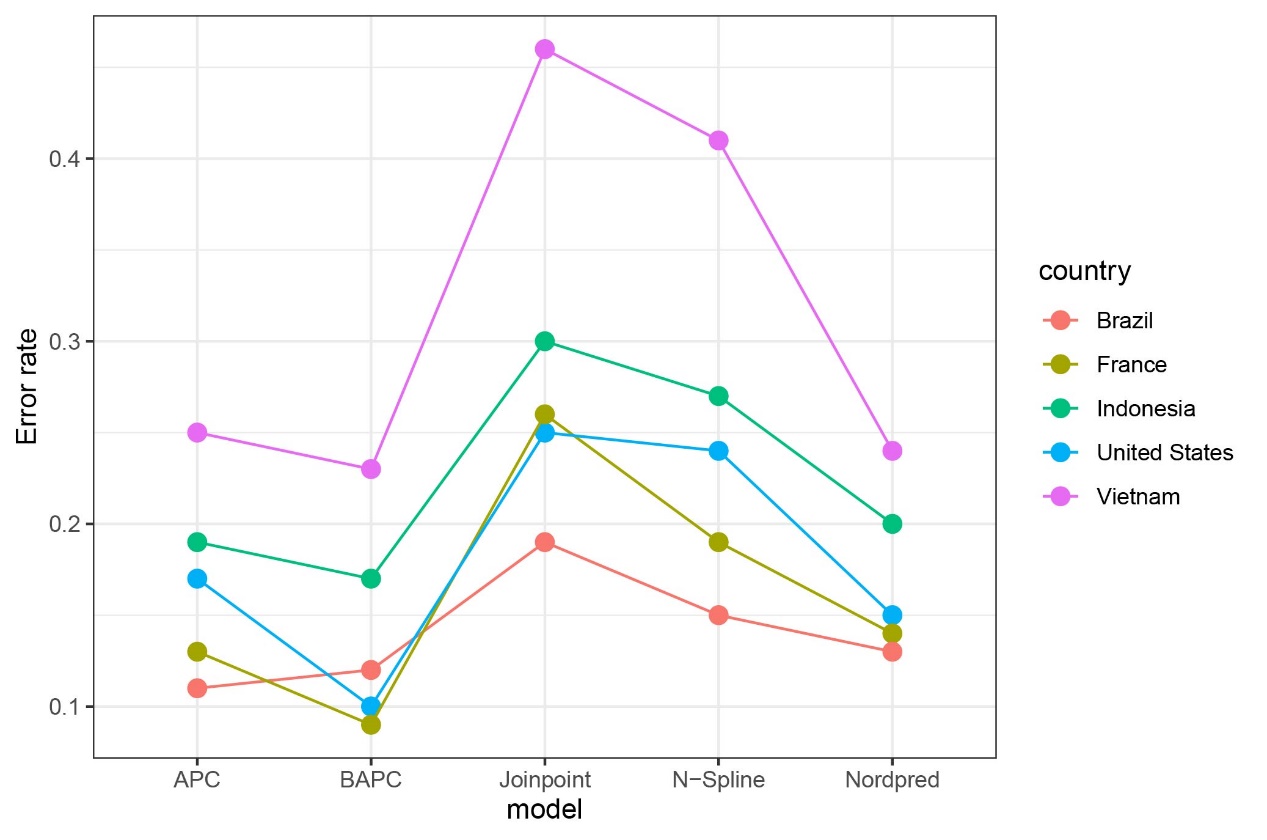


S-Figure 2. The predictions of kidney cancer incidence based on GBD data and IARC data (CI5 plus database). (IARC data: Australia, 1993-2012; Spain, 1993-2010; France, 1998-2010; Italy, 1998-2010; USA, 1990-2012). The blue triangles were point estimates of kidney cancer incidence based on CI5p data. The white open dots were point estimates of kidney cancer incidence based on GBD data.


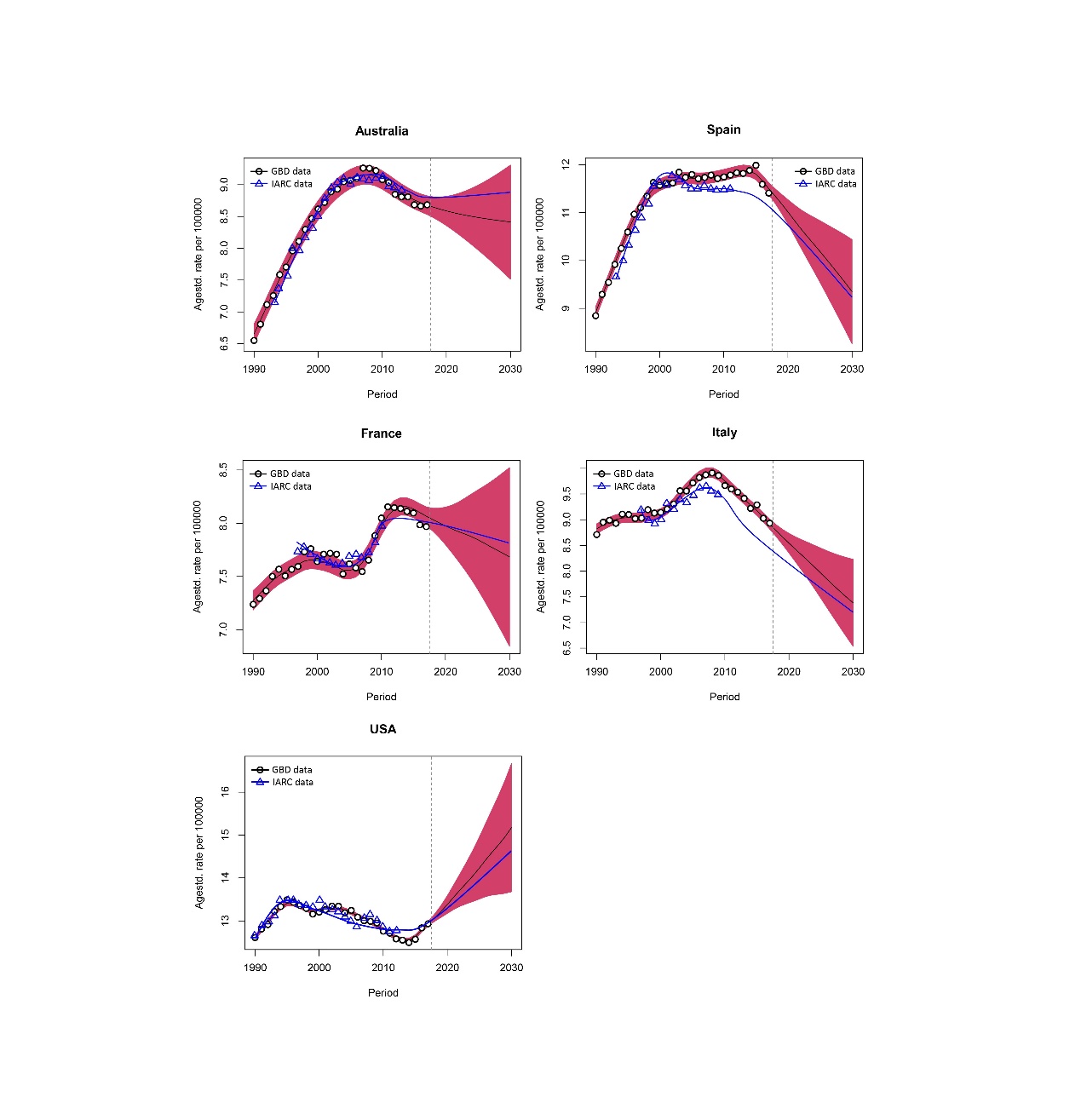

Supplement: Supplementary file 1 — Additional file 1: Figure S1. The prediction error rate of five models based on data from Brazil, France, Indonesia, USA, and Vietnam. Figure S2. The predictions of kidney cancer incidence based on GBD data and IARC data (CI5 plus database). (IARC data: Australia, 1993–2012; Spain, 1993–2010; France, 1998–2010; Italy, 1998–2010; USA, 1990–2012). The blue triangles were point estimates of kidney cancer incidence based on CI5p data. The white open dots were point estimates of kidney cancer incidence based on GBD data. [file 40364_2020_195_MOESM1_ESM.docx]
