# Supplementary material for: Trends and projections of kidney cancer incidence at the global and national levels, 1990–2030: a Bayesian age-period-cohort modeling study
Source: Biomark Res. 2020 May 13;8:16. doi: 10.1186/s40364-020-00195-3 (PMC7222434; doi:10.1186/s40364-020-00195-3)
Supplement: Supplementary file 2 — Additional file 2: Table S1. The AAPC of kidney cancer incidence in 1990–2017 and 2018–2030. [file 40364_2020_195_MOESM2_ESM.docx]

S-Table 1. The AAPC of kidney cancer incidence in 1990 – 2017 and 2018 – 2030.

| Region | 1990-2017 | | | | 2018-2030 | | | |
| --- | --- | --- | --- | --- | --- | --- | --- | --- |
|  | AAPC | 95% CI | | P value | AAPC | 95% CI | | P value |
| Afghanistan | -1.13 | -1.42 | -0.83 | 0 | 0.68 | 0.62 | 0.74 | 0 |
| Albania | 1.71 | 1.41 | 2.02 | 0 | -0.93 | -0.94 | -0.92 | 0 |
| Algeria | 1.26 | 1.15 | 1.37 | 0 | 0.3 | 0.29 | 0.3 | 0 |
| Angola | 1.23 | 1.13 | 1.33 | 0 | 0.63 | 0.63 | 0.64 | 0 |
| Argentina | -0.37 | -0.75 | 0 | 0.051 | -0.22 | -0.3 | -0.14 | 0 |
| Armenia | 6.24 | 5.12 | 7.36 | 0 | -0.85 | -0.92 | -0.78 | 0 |
| Australia | 0.92 | 0.64 | 1.21 | 0 | -0.1 | -0.12 | -0.08 | 0 |
| Austria | -1.07 | -1.14 | -1 | 0 | -1.61 | -1.63 | -1.6 | 0 |
| Azerbaijan | -0.83 | -1.11 | -0.55 | 0 | 1.12 | 1.1 | 1.14 | 0 |
| Bahamas | -0.61 | -0.71 | -0.5 | 0 | -0.19 | -0.21 | -0.18 | 0 |
| Bahrain | -1.09 | -1.26 | -0.91 | 0 | -1.4 | -1.41 | -1.39 | 0 |
| Bangladesh | 0.37 | 0.16 | 0.57 | 0.001 | 0.24 | 0.2 | 0.28 | 0 |
| Barbados | -1.43 | -1.71 | -1.15 | 0 | -0.52 | -0.59 | -0.45 | 0 |
| Belarus | 4.63 | 3.38 | 5.89 | 0 | -0.75 | -0.83 | -0.68 | 0 |
| Belgium | 0.46 | 0.18 | 0.75 | 0.003 | -1.35 | -1.37 | -1.33 | 0 |
| Belize | 3.39 | 2.84 | 3.95 | 0 | 1.48 | 1.43 | 1.53 | 0 |
| Benin | 2.74 | 2.66 | 2.82 | 0 | 1.53 | 1.51 | 1.55 | 0 |
| Bhutan | 1.6 | 1.15 | 2.05 | 0 | 2.41 | 2.16 | 2.67 | 0 |
| Bolivia | 1.53 | 1.41 | 1.65 | 0 | 0.19 | 0.14 | 0.25 | 0 |
| Bosnia and Herzegovina | 1.11 | 0.92 | 1.3 | 0 | -0.54 | -0.55 | -0.53 | 0 |
| Botswana | 1.41 | 1.36 | 1.46 | 0 | 1.37 | 1.33 | 1.41 | 0 |
| Brazil | 1.1 | 1.05 | 1.15 | 0 | 1.08 | 1.07 | 1.09 | 0 |
| Bulgaria | 4.82 | 3.9 | 5.76 | 0 | -3.12 | -3.15 | -3.1 | 0 |
| Burkina Faso | 2.04 | 1.88 | 2.21 | 0 | 3.46 | 3.45 | 3.46 | 0 |
| Burundi | -0.7 | -0.74 | -0.65 | 0 | -1.37 | -1.38 | -1.36 | 0 |
| Cambodia | 0.41 | 0.39 | 0.42 | 0 | 0.36 | 0.33 | 0.39 | 0 |
| Cameroon | 2.92 | 2.74 | 3.1 | 0 | 0.55 | 0.53 | 0.58 | 0 |
| Canada | 1.51 | 1.1 | 1.93 | 0 | -1.14 | -1.15 | -1.14 | 0 |
| Central African Republic | 1.62 | 1.56 | 1.68 | 0 | 0.57 | 0.54 | 0.61 | 0 |
| Chad | 2.07 | 2.02 | 2.12 | 0 | 1.58 | 1.57 | 1.58 | 0 |
| Chile | 0.78 | 0.67 | 0.89 | 0 | 0.36 | 0.34 | 0.38 | 0 |
| China | 1.08 | 0.88 | 1.27 | 0 | -2.17 | -2.21 | -2.14 | 0 |
| Colombia | 1.31 | 1.19 | 1.43 | 0 | 0.4 | 0.39 | 0.41 | 0 |
| Comoros | 1.51 | 1.22 | 1.79 | 0 | 0.25 | 0.19 | 0.32 | 0 |
| Costa Rica | 2.41 | 2.29 | 2.54 | 0 | 1.93 | 1.93 | 1.93 | 0 |
| Croatia | 3.64 | 3.04 | 4.25 | 0 | -5.07 | -5.13 | -5.02 | 0 |
| Cuba | -1.25 | -1.66 | -0.84 | 0 | 0.36 | 0.32 | 0.4 | 0 |
| Cyprus | 3.83 | 3.66 | 4 | 0 | 2.25 | 2.19 | 2.31 | 0 |
| Czech Republic | 0.7 | 0.19 | 1.21 | 0.009 | -2.1 | -2.11 | -2.08 | 0 |
| Democratic Republic of the Congo | -0.23 | -0.45 | -0.01 | 0.045 | 0.79 | 0.76 | 0.82 | 0 |
| Denmark | 1.55 | 1.25 | 1.85 | 0 | -0.9 | -0.92 | -0.87 | 0 |
| Djibouti | 4.29 | 3.83 | 4.75 | 0 | 2.19 | 2.15 | 2.22 | 0 |
| Dominica | 1.64 | -4.74 | 8.44 | 0.611 | 0.38 | 0.37 | 0.39 | 0 |
| Ecuador | 1.65 | 1.17 | 2.13 | 0 | 1.34 | 1.31 | 1.37 | 0 |
| Egypt | 1.49 | 1.45 | 1.54 | 0 | 0.19 | 0.14 | 0.24 | 0 |
| El Salvador | 1.05 | 1.01 | 1.1 | 0 | 2.84 | 2.76 | 2.93 | 0 |
| Equatorial Guinea | 2.18 | 2.14 | 2.22 | 0 | -0.39 | -0.43 | -0.35 | 0 |
| Eritrea | 2.35 | 1.91 | 2.79 | 0 | -2.96 | -3.03 | -2.9 | 0 |
| Estonia | 4.05 | 2.87 | 5.25 | 0 | -0.41 | -0.42 | -0.4 | 0 |
| Ethiopia | -1.31 | -1.43 | -1.19 | 0 | -4.21 | -4.91 | -3.5 | 0 |
| Fiji | -0.51 | -0.53 | -0.48 | 0 | -0.15 | -0.18 | -0.12 | 0 |
| Finland | 0.42 | 0.27 | 0.57 | 0 | -0.69 | -0.7 | -0.68 | 0 |
| France | 0.38 | 0.31 | 0.46 | 0 | -0.42 | -0.44 | -0.41 | 0 |
| Gabon | 1.8 | 1.58 | 2.02 | 0 | -0.67 | -0.72 | -0.62 | 0 |
| Gambia | 2.3 | 2.2 | 2.4 | 0 | 1.93 | 1.87 | 1.99 | 0 |
| Georgia | 1.09 | 0.44 | 1.75 | 0.002 | 1.48 | 1.44 | 1.53 | 0 |
| Germany | -0.28 | -0.35 | -0.22 | 0 | 0.1 | 0.07 | 0.13 | 0 |
| Ghana | -0.98 | -1.57 | -0.39 | 0.002 | 3.25 | 3.24 | 3.26 | 0 |
| Global | 0.14 | 0.08 | 0.2 | 0 | -0.97 | -0.99 | -0.95 | 0 |
| Greece | 1.25 | 1.05 | 1.45 | 0 | -0.26 | -0.28 | -0.24 | 0 |
| Guam | 2.14 | 1.85 | 2.43 | 0 | 1.13 | 1.03 | 1.23 | 0 |
| Guatemala | 1.16 | 0.55 | 1.76 | 0.001 | 1.67 | 1.63 | 1.71 | 0 |
| Guinea | 0.77 | 0.66 | 0.89 | 0 | 1.24 | 1.23 | 1.25 | 0 |
| Guinea-Bissau | 2.37 | 2.26 | 2.48 | 0 | 1.63 | 1.58 | 1.67 | 0 |
| Guyana | 0.09 | 0.01 | 0.17 | 0.03 | 0.06 | 0.04 | 0.09 | 0 |
| Haiti | -0.44 | -0.64 | -0.24 | 0 | 0 | -0.03 | 0.04 | 0.868 |
| Honduras | 1.84 | 1.8 | 1.89 | 0 | 0.75 | 0.69 | 0.81 | 0 |
| Hungary | -0.89 | -1.02 | -0.75 | 0 | -3.78 | -3.84 | -3.72 | 0 |
| Iceland | 0.99 | 0.85 | 1.13 | 0 | -0.38 | -0.39 | -0.37 | 0 |
| India | 1.58 | 1.48 | 1.68 | 0 | 1.81 | 1.8 | 1.83 | 0 |
| Indonesia | 1.38 | 1.22 | 1.55 | 0 | 0.1 | 0.08 | 0.12 | 0 |
| Iran | 2 | 1.74 | 2.26 | 0 | -2.39 | -2.39 | -2.38 | 0 |
| Iraq | 0.75 | 0.57 | 0.92 | 0 | -0.97 | -0.99 | -0.96 | 0 |
| Ireland | 1.29 | 0.95 | 1.64 | 0 | -1.07 | -1.09 | -1.04 | 0 |
| Israel | 0.2 | 0.01 | 0.39 | 0.04 | -0.99 | -1 | -0.99 | 0 |
| Italy | 0.23 | 0.07 | 0.39 | 0.008 | -1.47 | -1.49 | -1.45 | 0 |
| Ivory Coast | 2.21 | 2.05 | 2.37 | 0 | 1.24 | 1.21 | 1.26 | 0 |
| Jamaica | -0.7 | -1.17 | -0.24 | 0.005 | 2.21 | 2.21 | 2.22 | 0 |
| Japan | 0.86 | 0.62 | 1.11 | 0 | -0.7 | -0.72 | -0.68 | 0 |
| Jordan | 2.23 | 2.2 | 2.26 | 0 | 1.41 | 1.37 | 1.46 | 0 |
| Kazakhstan | -0.81 | -1.27 | -0.36 | 0.001 | -1.14 | -1.16 | -1.11 | 0 |
| Kenya | 1.08 | 0.97 | 1.2 | 0 | -0.18 | -0.21 | -0.16 | 0 |
| Kuwait | -0.68 | -1.02 | -0.35 | 0 | -1.34 | -1.35 | -1.32 | 0 |
| Kyrgyzstan | 0.61 | 0.1 | 1.11 | 0.021 | -0.96 | -0.97 | -0.95 | 0 |
| Laos | 0.8 | 0.67 | 0.94 | 0 | -0.32 | -0.34 | -0.3 | 0 |
| Latvia | 4.04 | 3.18 | 4.91 | 0 | -2.23 | -2.27 | -2.19 | 0 |
| Lebanon | 1.54 | 1.35 | 1.72 | 0 | -0.07 | -0.09 | -0.05 | 0 |
| Lesotho | 2.2 | 1.95 | 2.45 | 0 | 0.28 | 0.28 | 0.29 | 0 |
| Liberia | 0.14 | -0.36 | 0.64 | 0.564 | 2.28 | 2.27 | 2.3 | 0 |
| Libya | 2.57 | 2.49 | 2.64 | 0 | 1.61 | 1.6 | 1.62 | 0 |
| Lithuania | 3.94 | 3.15 | 4.73 | 0 | -1.85 | -1.9 | -1.8 | 0 |
| Luxembourg | -0.12 | -0.15 | -0.1 | 0 | -0.74 | -0.8 | -0.68 | 0 |
| Macedonia | 3.95 | 3.6 | 4.3 | 0 | 0.72 | 0.7 | 0.75 | 0 |
| Madagascar | -0.08 | -0.26 | 0.09 | 0.339 | 0.39 | 0.35 | 0.43 | 0 |
| Malawi | -0.31 | -0.49 | -0.12 | 0.002 | 0 | -0.03 | 0.02 | 0.709 |
| Malaysia | 1.38 | 1.27 | 1.48 | 0 | -0.06 | -0.08 | -0.04 | 0 |
| Mali | 1.8 | 1.68 | 1.92 | 0 | 2.54 | 2.5 | 2.58 | 0 |
| Malta | 1.63 | 1.6 | 1.67 | 0 | 1.86 | 1.82 | 1.91 | 0 |
| Mauritania | 1.09 | 1.03 | 1.15 | 0 | 0.3 | 0.29 | 0.3 | 0 |
| Mauritius | 0.95 | 0.79 | 1.11 | 0 | -0.35 | -0.37 | -0.33 | 0 |
| Mexico | 1.5 | 1.39 | 1.61 | 0 | 0.17 | 0.14 | 0.19 | 0 |
| Moldova | 0.56 | 0.41 | 0.7 | 0 | -0.49 | -0.53 | -0.44 | 0 |
| Mongolia | 1.84 | 1.53 | 2.15 | 0 | 0.08 | 0.05 | 0.12 | 0 |
| Montenegro | -0.33 | -0.36 | -0.3 | 0 | -1.64 | -1.78 | -1.5 | 0 |
| Morocco | 1.14 | 1.12 | 1.16 | 0 | 0.71 | 0.69 | 0.73 | 0 |
| Mozambique | 0.48 | 0.06 | 0.9 | 0.027 | 1.73 | 1.71 | 1.76 | 0 |
| Myanmar | 0.82 | 0.6 | 1.03 | 0 | -0.59 | -0.6 | -0.58 | 0 |
| Namibia | 0.43 | 0.15 | 0.71 | 0.004 | -0.89 | -0.92 | -0.86 | 0 |
| Nepal | 1.73 | 1.38 | 2.08 | 0 | 2.72 | 2.68 | 2.76 | 0 |
| Netherlands | 0.77 | 0.56 | 0.98 | 0 | -0.7 | -0.72 | -0.69 | 0 |
| New Zealand | 0.5 | 0.4 | 0.6 | 0 | -0.58 | -0.59 | -0.58 | 0 |
| Nicaragua | 0.42 | 0.34 | 0.5 | 0 | -0.25 | -0.3 | -0.2 | 0 |
| Niger | 0.48 | 0.31 | 0.64 | 0 | 1.35 | 1.33 | 1.37 | 0 |
| Nigeria | 0.6 | 0.44 | 0.77 | 0 | 2.12 | 2.12 | 2.12 | 0 |
| North Korea | 0.34 | 0.27 | 0.42 | 0 | -0.32 | -0.37 | -0.28 | 0 |
| Norway | 1.94 | 1.61 | 2.26 | 0 | -2.26 | -2.3 | -2.21 | 0 |
| Oman | 2.38 | 2.36 | 2.4 | 0 | 1.63 | 1.59 | 1.67 | 0 |
| Pakistan | 1.94 | 1.81 | 2.08 | 0 | 0.71 | 0.71 | 0.71 | 0 |
| Palestine | 0.84 | 0.72 | 0.97 | 0 | 0.75 | 0.74 | 0.77 | 0 |
| Panama | 3.25 | 3.2 | 3.29 | 0 | 2.64 | 2.63 | 2.64 | 0 |
| Papua New Guinea | 0.68 | 0.61 | 0.75 | 0 | 0.88 | 0.85 | 0.91 | 0 |
| Paraguay | 0.16 | 0.12 | 0.21 | 0 | 0.21 | 0.16 | 0.25 | 0 |
| Peru | -0.64 | -0.95 | -0.32 | 0 | -0.45 | -0.5 | -0.4 | 0 |
| Philippines | 1.49 | 1.22 | 1.76 | 0 | -1.1 | -1.11 | -1.08 | 0 |
| Poland | 0.79 | 0.66 | 0.91 | 0 | -1.29 | -1.31 | -1.28 | 0 |
| Portugal | 0.39 | 0.2 | 0.59 | 0 | -1.55 | -1.61 | -1.48 | 0 |
| Puerto Rico | 0.2 | 0.01 | 0.4 | 0.042 | 0.03 | -0.01 | 0.08 | 0.139 |
| Qatar | -1.94 | -2.11 | -1.77 | 0 | -1.67 | -1.8 | -1.54 | 0 |
| Republic of Congo | 0.81 | 0.76 | 0.86 | 0 | 0.66 | 0.63 | 0.68 | 0 |
| Romania | 0.91 | 0.82 | 1.01 | 0 | 0.13 | 0.08 | 0.19 | 0 |
| Russia | -0.28 | -0.56 | -0.01 | 0.042 | -0.78 | -0.91 | -0.65 | 0 |
| Rwanda | -1.1 | -1.63 | -0.57 | 0 | 1.43 | 1.42 | 1.44 | 0 |
| Saint Lucia | -0.89 | -0.98 | -0.8 | 0 | -2 | -2.1 | -1.9 | 0 |
| Saudi Arabia | 2.94 | 2.62 | 3.26 | 0 | 0.23 | 0.22 | 0.24 | 0 |
| Senegal | 1.88 | 1.78 | 1.98 | 0 | 1.04 | 0.99 | 1.09 | 0 |
| Serbia | 0.91 | 0.76 | 1.07 | 0 | -1.69 | -1.74 | -1.65 | 0 |
| Sierra Leone | 1.87 | 1.76 | 1.97 | 0 | 2.48 | 2.47 | 2.48 | 0 |
| Singapore | 0.41 | 0.34 | 0.48 | 0 | -0.48 | -0.5 | -0.46 | 0 |
| Slovakia | 3.35 | 2.5 | 4.2 | 0 | -4.09 | -4.1 | -4.07 | 0 |
| Slovenia | 1.59 | 1.23 | 1.96 | 0 | -2.13 | -2.18 | -2.08 | 0 |
| Solomon Islands | 2.3 | 1.95 | 2.64 | 0 | -0.19 | -0.22 | -0.17 | 0 |
| Somalia | 1.15 | 0.99 | 1.32 | 0 | -0.09 | -0.13 | -0.05 | 0 |
| South Africa | 0.43 | -0.14 | 1 | 0.135 | 0.07 | -0.01 | 0.14 | 0.067 |
| South Korea | 2.92 | 2.4 | 3.43 | 0 | -2.56 | -2.6 | -2.51 | 0 |
| South Sudan | -0.71 | -0.83 | -0.58 | 0 | -0.6 | -0.61 | -0.6 | 0 |
| Spain | 0.78 | 0.53 | 1.03 | 0 | -1.8 | -1.83 | -1.77 | 0 |
| Sri Lanka | -2.71 | -3.85 | -1.56 | 0 | -1.66 | -1.69 | -1.63 | 0 |
| Sudan | 0.72 | 0.46 | 0.99 | 0 | 1.03 | 1.02 | 1.05 | 0 |
| Suriname | 0.12 | -0.07 | 0.3 | 0.213 | 0.44 | 0.4 | 0.47 | 0 |
| Swaziland | 3.07 | 2.26 | 3.89 | 0 | -2.93 | -2.93 | -2.92 | 0 |
| Sweden | 0.69 | 0.58 | 0.81 | 0 | -0.42 | -0.45 | -0.38 | 0 |
| Switzerland | 0.44 | 0.04 | 0.84 | 0.033 | -2.14 | -2.15 | -2.14 | 0 |
| Syria | 1.5 | 1.34 | 1.65 | 0 | 2.24 | 2.22 | 2.26 | 0 |
| Tajikistan | 0.15 | -0.18 | 0.48 | 0.37 | 2.03 | 2.02 | 2.04 | 0 |
| Tanzania | 0.82 | 0.71 | 0.93 | 0 | 0.66 | 0.66 | 0.67 | 0 |
| Thailand | 0.21 | -0.03 | 0.45 | 0.08 | 2.12 | 2.1 | 2.13 | 0 |
| Timor-Leste | 1.84 | 1.78 | 1.91 | 0 | 1.75 | 1.71 | 1.79 | 0 |
| Togo | 2.34 | 2.28 | 2.39 | 0 | 1.25 | 1.2 | 1.3 | 0 |
| Trinidad and Tobago | -2.16 | -2.56 | -1.76 | 0 | 0.67 | 0.66 | 0.67 | 0 |
| Tunisia | 0.79 | 0.73 | 0.84 | 0 | 0.14 | 0.1 | 0.18 | 0 |
| Turkey | 0.17 | 0.01 | 0.33 | 0.041 | -1.69 | -1.73 | -1.66 | 0 |
| Turkmenistan | 0.73 | 0.67 | 0.79 | 0 | -0.14 | -0.14 | -0.13 | 0 |
| Uganda | 1.73 | 1.58 | 1.89 | 0 | -1.32 | -1.32 | -1.31 | 0 |
| UK | 0.93 | 0.84 | 1.02 | 0 | 0.75 | 0.72 | 0.78 | 0 |
| Ukraine | 0.75 | 0.45 | 1.06 | 0 | -6.62 | -6.65 | -6.58 | 0 |
| United Arab Emirates | 1.45 | 1.29 | 1.61 | 0 | 3.68 | 3.63 | 3.73 | 0 |
| Uruguay | 0.2 | 0.04 | 0.35 | 0.016 | 0.7 | 0.68 | 0.72 | 0 |
| USA | -0.14 | -0.23 | -0.05 | 0.004 | 1.26 | 1.25 | 1.27 | 0 |
| Uzbekistan | 1.5 | 1.39 | 1.61 | 0 | 0.63 | 0.61 | 0.65 | 0 |
| Venezuela | 0.25 | -0.26 | 0.76 | 0.323 | 0.72 | 0.7 | 0.75 | 0 |
| Vietnam | 0.75 | 0.7 | 0.8 | 0 | 0.41 | 0.41 | 0.41 | 0 |
| Yemen | 1.73 | 1.57 | 1.88 | 0 | 1.05 | 1.04 | 1.06 | 0 |
| Zambia | 0.15 | -0.05 | 0.36 | 0.133 | 0.79 | 0.77 | 0.81 | 0 |
| Zimbabwe | 1.44 | 0.87 | 2.01 | 0 | -1.59 | -1.61 | -1.57 | 0 |
