# Supplementary material for: Trends and projections of kidney cancer incidence at the global and national levels, 1990–2030: a Bayesian age-period-cohort modeling study
Source: Biomark Res. 2020 May 13;8:16. doi: 10.1186/s40364-020-00195-3 (PMC7222434; doi:10.1186/s40364-020-00195-3)
Supplement: Supplementary file 3 — Additional file 3: Table S2. The predicting kidney cancer cases at the national level from 2018 to 2030. [file 40364_2020_195_MOESM3_ESM.docx]

S-Table 2. The predicting kidney cancer cases at the national level from 2018 to 2030.

| Region | Sex | Cases | 95% HDI | | Year |
| --- | --- | --- | --- | --- | --- |
| Afghanistan | Both | 719.7 | 640.2 | 799.2 | 2018 |
| Afghanistan | Both | 746.5 | 660.4 | 832.5 | 2019 |
| Afghanistan | Both | 775 | 680.3 | 869.8 | 2020 |
| Afghanistan | Both | 805.8 | 699.7 | 912 | 2021 |
| Afghanistan | Both | 839.6 | 719.1 | 960.2 | 2022 |
| Afghanistan | Both | 876.8 | 738.2 | 1015.3 | 2023 |
| Afghanistan | Both | 916.7 | 756 | 1077.4 | 2024 |
| Afghanistan | Both | 959.8 | 772.1 | 1147.6 | 2025 |
| Afghanistan | Both | 1006.6 | 785.8 | 1227.3 | 2026 |
| Afghanistan | Both | 1059.5 | 797.7 | 1321.4 | 2027 |
| Afghanistan | Both | 1120.1 | 806.6 | 1433.7 | 2028 |
| Afghanistan | Both | 1188.2 | 809.4 | 1567 | 2029 |
| Afghanistan | Both | 1264.5 | 802.4 | 1726.5 | 2030 |
| Albania | Both | 196 | 160.4 | 231.6 | 2018 |
| Albania | Both | 198.6 | 162.1 | 235.2 | 2019 |
| Albania | Both | 201.4 | 163.5 | 239.3 | 2020 |
| Albania | Both | 204.2 | 164.5 | 244 | 2021 |
| Albania | Both | 207 | 164.9 | 249.2 | 2022 |
| Albania | Both | 210 | 164.9 | 255.2 | 2023 |
| Albania | Both | 213.3 | 164.6 | 262.1 | 2024 |
| Albania | Both | 217.1 | 163.9 | 270.3 | 2025 |
| Albania | Both | 220.8 | 162.5 | 279.2 | 2026 |
| Albania | Both | 225 | 160.5 | 289.6 | 2027 |
| Albania | Both | 229.7 | 157.8 | 301.6 | 2028 |
| Albania | Both | 234.7 | 154.2 | 315.4 | 2029 |
| Albania | Both | 240.2 | 149.4 | 331.2 | 2030 |
| Algeria | Both | 723.5 | 648 | 799 | 2018 |
| Algeria | Both | 748.9 | 670.4 | 827.3 | 2019 |
| Algeria | Both | 774.9 | 692.7 | 857.2 | 2020 |
| Algeria | Both | 802.2 | 715.1 | 889.2 | 2021 |
| Algeria | Both | 829.5 | 736.5 | 922.6 | 2022 |
| Algeria | Both | 857.6 | 757.2 | 958.1 | 2023 |
| Algeria | Both | 887.3 | 777.9 | 996.6 | 2024 |
| Algeria | Both | 918.8 | 798.9 | 1038.7 | 2025 |
| Algeria | Both | 950.1 | 818 | 1082.1 | 2026 |
| Algeria | Both | 983.4 | 837.3 | 1129.6 | 2027 |
| Algeria | Both | 1018.8 | 856.5 | 1181.1 | 2028 |
| Algeria | Both | 1055.8 | 875.2 | 1236.5 | 2029 |
| Algeria | Both | 1094.4 | 893.1 | 1295.6 | 2030 |
| Angola | Both | 647.1 | 577.3 | 717 | 2018 |
| Angola | Both | 661.3 | 588.1 | 734.6 | 2019 |
| Angola | Both | 677.2 | 599.7 | 754.7 | 2020 |
| Angola | Both | 693.8 | 611.2 | 776.4 | 2021 |
| Angola | Both | 712 | 623.4 | 800.5 | 2022 |
| Angola | Both | 731.9 | 636.4 | 827.4 | 2023 |
| Angola | Both | 753.7 | 650.2 | 857.3 | 2024 |
| Angola | Both | 777.7 | 664.8 | 890.6 | 2025 |
| Angola | Both | 802.4 | 678.9 | 925.8 | 2026 |
| Angola | Both | 829.1 | 693.5 | 964.6 | 2027 |
| Angola | Both | 857.9 | 708.7 | 1007.2 | 2028 |
| Angola | Both | 889.2 | 724.4 | 1053.9 | 2029 |
| Angola | Both | 922.9 | 740.7 | 1105.2 | 2030 |
| Argentina | Both | 6239.6 | 6004.7 | 6474.5 | 2018 |
| Argentina | Both | 6385.6 | 6084.9 | 6686.3 | 2019 |
| Argentina | Both | 6538.5 | 6136.7 | 6940.3 | 2020 |
| Argentina | Both | 6690.9 | 6157.7 | 7224.2 | 2021 |
| Argentina | Both | 6850 | 6157.1 | 7542.8 | 2022 |
| Argentina | Both | 7019 | 6138.4 | 7899.6 | 2023 |
| Argentina | Both | 7203.1 | 6104.9 | 8301.2 | 2024 |
| Argentina | Both | 7405.6 | 6056.9 | 8754.3 | 2025 |
| Argentina | Both | 7617.1 | 5982.7 | 9251.4 | 2026 |
| Argentina | Both | 7849.7 | 5887 | 9812.4 | 2027 |
| Argentina | Both | 8109.4 | 5766.6 | 10452.2 | 2028 |
| Argentina | Both | 8404.5 | 5617.4 | 11191.7 | 2029 |
| Argentina | Both | 8741.9 | 5429.9 | 12053.9 | 2030 |
| Armenia | Both | 268.1 | 222.8 | 313.4 | 2018 |
| Armenia | Both | 272.7 | 220.7 | 324.7 | 2019 |
| Armenia | Both | 280 | 217.5 | 342.5 | 2020 |
| Armenia | Both | 290.8 | 213.4 | 368.2 | 2021 |
| Armenia | Both | 305.2 | 207.5 | 402.9 | 2022 |
| Armenia | Both | 323.9 | 198.3 | 449.5 | 2023 |
| Armenia | Both | 347.8 | 182.7 | 513 | 2024 |
| Armenia | Both | 378.8 | 154.6 | 603 | 2025 |
| Armenia | Both | 419.3 | 100.7 | 738.1 | 2026 |
| Armenia | Both | 473.5 | 0 | 956.8 | 2027 |
| Armenia | Both | 547.9 | 0 | 1342.6 | 2028 |
| Armenia | Both | 653.3 | 0 | 2087.5 | 2029 |
| Armenia | Both | 808.3 | 0 | 3646.9 | 2030 |
| Australia | Both | 3288.1 | 3140.8 | 3435.5 | 2018 |
| Australia | Both | 3349.2 | 3191.4 | 3507.1 | 2019 |
| Australia | Both | 3413.7 | 3240.2 | 3587.2 | 2020 |
| Australia | Both | 3478.7 | 3284.1 | 3673.3 | 2021 |
| Australia | Both | 3546.6 | 3325.3 | 3767.9 | 2022 |
| Australia | Both | 3617.6 | 3364.1 | 3871.1 | 2023 |
| Australia | Both | 3691.3 | 3400.2 | 3982.4 | 2024 |
| Australia | Both | 3767.8 | 3433.8 | 4101.7 | 2025 |
| Australia | Both | 3843.2 | 3461.4 | 4225 | 2026 |
| Australia | Both | 3920.9 | 3485.8 | 4355.9 | 2027 |
| Australia | Both | 4001.8 | 3507.9 | 4495.6 | 2028 |
| Australia | Both | 4086.9 | 3528.3 | 4645.4 | 2029 |
| Australia | Both | 4177.2 | 3547.5 | 4806.8 | 2030 |
| Austria | Both | 1116.7 | 1036 | 1197.5 | 2018 |
| Austria | Both | 1116.3 | 1033.2 | 1199.4 | 2019 |
| Austria | Both | 1116.7 | 1030 | 1203.4 | 2020 |
| Austria | Both | 1117.2 | 1025.6 | 1208.8 | 2021 |
| Austria | Both | 1117.9 | 1020 | 1215.7 | 2022 |
| Austria | Both | 1118.5 | 1013.2 | 1223.7 | 2023 |
| Austria | Both | 1118.9 | 1005.1 | 1232.8 | 2024 |
| Austria | Both | 1119.5 | 995.9 | 1243.2 | 2025 |
| Austria | Both | 1119.7 | 985.2 | 1254.2 | 2026 |
| Austria | Both | 1120.9 | 974.3 | 1267.5 | 2027 |
| Austria | Both | 1123.4 | 963.5 | 1283.3 | 2028 |
| Austria | Both | 1127.1 | 952.7 | 1301.6 | 2029 |
| Austria | Both | 1131.6 | 941.4 | 1321.8 | 2030 |
| Azerbaijan | Both | 909.7 | 827.8 | 991.7 | 2018 |
| Azerbaijan | Both | 947 | 857.2 | 1036.8 | 2019 |
| Azerbaijan | Both | 985.4 | 883.6 | 1087.1 | 2020 |
| Azerbaijan | Both | 1025.4 | 907.2 | 1143.6 | 2021 |
| Azerbaijan | Both | 1066.1 | 926.6 | 1205.5 | 2022 |
| Azerbaijan | Both | 1108.2 | 942.6 | 1273.9 | 2023 |
| Azerbaijan | Both | 1153.6 | 956.1 | 1351.1 | 2024 |
| Azerbaijan | Both | 1203.4 | 967.5 | 1439.2 | 2025 |
| Azerbaijan | Both | 1254.4 | 973.5 | 1535.3 | 2026 |
| Azerbaijan | Both | 1310.8 | 976.1 | 1645.5 | 2027 |
| Azerbaijan | Both | 1373.5 | 974.4 | 1772.7 | 2028 |
| Azerbaijan | Both | 1443.3 | 966.3 | 1920.2 | 2029 |
| Azerbaijan | Both | 1521 | 949.3 | 2092.6 | 2030 |
| Bahrain | Both | 39 | 23.1 | 55.2 | 2018 |
| Bahrain | Both | 41 | 24.6 | 57.7 | 2019 |
| Bahrain | Both | 42.8 | 25.8 | 60.1 | 2020 |
| Bahrain | Both | 44.9 | 27.3 | 62.9 | 2021 |
| Bahrain | Both | 46.9 | 28.6 | 65.5 | 2022 |
| Bahrain | Both | 48.6 | 29.6 | 68 | 2023 |
| Bahrain | Both | 50.4 | 30.5 | 70.7 | 2024 |
| Bahrain | Both | 52.3 | 31.3 | 73.7 | 2025 |
| Bahrain | Both | 54.4 | 32.1 | 77.1 | 2026 |
| Bahrain | Both | 56.7 | 32.8 | 80.9 | 2027 |
| Bahrain | Both | 59.1 | 33.3 | 85.2 | 2028 |
| Bahrain | Both | 61.6 | 33.5 | 90 | 2029 |
| Bahrain | Both | 64.2 | 33.4 | 95.4 | 2030 |
| Bangladesh | Both | 2034.4 | 1902.6 | 2166.2 | 2018 |
| Bangladesh | Both | 2093 | 1952.6 | 2233.4 | 2019 |
| Bangladesh | Both | 2155.8 | 2003.3 | 2308.3 | 2020 |
| Bangladesh | Both | 2223.4 | 2054.6 | 2392.2 | 2021 |
| Bangladesh | Both | 2295.1 | 2105.2 | 2485 | 2022 |
| Bangladesh | Both | 2370.4 | 2154.4 | 2586.4 | 2023 |
| Bangladesh | Both | 2449.5 | 2202.3 | 2696.7 | 2024 |
| Bangladesh | Both | 2533.9 | 2250.2 | 2817.7 | 2025 |
| Bangladesh | Both | 2622.9 | 2296.9 | 2948.9 | 2026 |
| Bangladesh | Both | 2716.6 | 2342.2 | 3091 | 2027 |
| Bangladesh | Both | 2814.7 | 2385.3 | 3244.1 | 2028 |
| Bangladesh | Both | 2917.8 | 2426.4 | 3409.3 | 2029 |
| Bangladesh | Both | 3027.9 | 2466.6 | 3589.2 | 2030 |
| Barbados | Both | 27.6 | 15.2 | 40.6 | 2018 |
| Barbados | Both | 28.1 | 15.6 | 41.3 | 2019 |
| Barbados | Both | 28.6 | 15.9 | 42 | 2020 |
| Barbados | Both | 29.2 | 16.2 | 42.7 | 2021 |
| Barbados | Both | 29.7 | 16.4 | 43.6 | 2022 |
| Barbados | Both | 30.3 | 16.6 | 44.5 | 2023 |
| Barbados | Both | 30.8 | 16.8 | 45.5 | 2024 |
| Barbados | Both | 31.4 | 16.9 | 46.6 | 2025 |
| Barbados | Both | 32 | 16.9 | 47.8 | 2026 |
| Barbados | Both | 32.7 | 16.8 | 49.2 | 2027 |
| Barbados | Both | 33.4 | 16.6 | 50.8 | 2028 |
| Barbados | Both | 34.1 | 16.3 | 52.6 | 2029 |
| Barbados | Both | 34.9 | 15.8 | 54.8 | 2030 |
| Belarus | Both | 1468.9 | 1364.4 | 1573.5 | 2018 |
| Belarus | Both | 1480.8 | 1352.9 | 1608.7 | 2019 |
| Belarus | Both | 1496.3 | 1332.2 | 1660.5 | 2020 |
| Belarus | Both | 1517.2 | 1304.8 | 1729.5 | 2021 |
| Belarus | Both | 1542.2 | 1270 | 1814.5 | 2022 |
| Belarus | Both | 1572.9 | 1228 | 1917.8 | 2023 |
| Belarus | Both | 1610.8 | 1177.9 | 2043.8 | 2024 |
| Belarus | Both | 1658.2 | 1117.4 | 2199 | 2025 |
| Belarus | Both | 1716.2 | 1041.1 | 2391.3 | 2026 |
| Belarus | Both | 1789.3 | 942.1 | 2636.5 | 2027 |
| Belarus | Both | 1881.9 | 807.3 | 2956.4 | 2028 |
| Belarus | Both | 1998.7 | 613.9 | 3383.4 | 2029 |
| Belarus | Both | 2145.9 | 323.1 | 3968.8 | 2030 |
| Belgium | Both | 1491.5 | 1393.9 | 1589.2 | 2018 |
| Belgium | Both | 1494.1 | 1387.2 | 1600.9 | 2019 |
| Belgium | Both | 1497.6 | 1376.4 | 1618.8 | 2020 |
| Belgium | Both | 1503.7 | 1363.1 | 1644.4 | 2021 |
| Belgium | Both | 1511 | 1346 | 1675.9 | 2022 |
| Belgium | Both | 1519.6 | 1325.9 | 1713.4 | 2023 |
| Belgium | Both | 1530 | 1302.8 | 1757.2 | 2024 |
| Belgium | Both | 1542.4 | 1276.8 | 1807.9 | 2025 |
| Belgium | Both | 1556.8 | 1247.5 | 1866 | 2026 |
| Belgium | Both | 1573.6 | 1214.4 | 1932.7 | 2027 |
| Belgium | Both | 1593.2 | 1176.8 | 2009.7 | 2028 |
| Belgium | Both | 1616.4 | 1133.5 | 2099.3 | 2029 |
| Belgium | Both | 1643.9 | 1083 | 2204.7 | 2030 |
| Belize | Both | 19.4 | 8 | 31.3 | 2018 |
| Belize | Both | 20.5 | 8.7 | 32.9 | 2019 |
| Belize | Both | 21.7 | 9.3 | 34.6 | 2020 |
| Belize | Both | 23 | 10 | 36.5 | 2021 |
| Belize | Both | 24.4 | 10.6 | 38.8 | 2022 |
| Belize | Both | 26 | 11.2 | 41.4 | 2023 |
| Belize | Both | 27.8 | 11.7 | 44.5 | 2024 |
| Belize | Both | 29.8 | 12 | 48.2 | 2025 |
| Belize | Both | 32 | 11.9 | 52.8 | 2026 |
| Belize | Both | 34.5 | 11.1 | 58.6 | 2027 |
| Belize | Both | 37.4 | 9.3 | 66.4 | 2028 |
| Belize | Both | 40.7 | 5.6 | 77.4 | 2029 |
| Belize | Both | 44.6 | 0.2 | 93.2 | 2030 |
| Benin | Both | 316.3 | 268.8 | 363.8 | 2018 |
| Benin | Both | 330.6 | 280.5 | 380.6 | 2019 |
| Benin | Both | 345.7 | 292.5 | 399 | 2020 |
| Benin | Both | 361.5 | 304.4 | 418.5 | 2021 |
| Benin | Both | 378 | 316.4 | 439.7 | 2022 |
| Benin | Both | 395.6 | 328.5 | 462.7 | 2023 |
| Benin | Both | 414.3 | 340.7 | 487.8 | 2024 |
| Benin | Both | 434.3 | 353.2 | 515.3 | 2025 |
| Benin | Both | 455.2 | 365.5 | 545 | 2026 |
| Benin | Both | 477.4 | 377.6 | 577.2 | 2027 |
| Benin | Both | 501 | 389.7 | 612.3 | 2028 |
| Benin | Both | 526.2 | 401.7 | 650.8 | 2029 |
| Benin | Both | 553.3 | 413.5 | 693.2 | 2030 |
| Bhutan | Both | 14.7 | 5.2 | 24.5 | 2018 |
| Bhutan | Both | 15.6 | 5.7 | 25.9 | 2019 |
| Bhutan | Both | 16.7 | 6.2 | 27.6 | 2020 |
| Bhutan | Both | 17.9 | 6.8 | 29.5 | 2021 |
| Bhutan | Both | 19.4 | 7.3 | 31.8 | 2022 |
| Bhutan | Both | 21 | 7.8 | 34.6 | 2023 |
| Bhutan | Both | 22.9 | 8.4 | 38 | 2024 |
| Bhutan | Both | 25.1 | 8.9 | 42.1 | 2025 |
| Bhutan | Both | 27.6 | 9.2 | 47.1 | 2026 |
| Bhutan | Both | 30.5 | 9.2 | 53.3 | 2027 |
| Bhutan | Both | 33.8 | 8.7 | 61.1 | 2028 |
| Bhutan | Both | 37.6 | 7.6 | 71.3 | 2029 |
| Bhutan | Both | 41.9 | 5.9 | 85 | 2030 |
| Bolivia | Both | 547 | 482.4 | 611.7 | 2018 |
| Bolivia | Both | 563.6 | 496.9 | 630.4 | 2019 |
| Bolivia | Both | 581 | 511.6 | 650.4 | 2020 |
| Bolivia | Both | 597.3 | 524.6 | 669.9 | 2021 |
| Bolivia | Both | 614.3 | 537.6 | 691.1 | 2022 |
| Bolivia | Both | 632.2 | 550.5 | 713.9 | 2023 |
| Bolivia | Both | 651 | 563.3 | 738.6 | 2024 |
| Bolivia | Both | 670.7 | 576 | 765.4 | 2025 |
| Bolivia | Both | 689.8 | 587 | 792.6 | 2026 |
| Bolivia | Both | 709.9 | 597.8 | 822 | 2027 |
| Bolivia | Both | 730.8 | 608.1 | 853.5 | 2028 |
| Bolivia | Both | 752.7 | 618 | 887.5 | 2029 |
| Bolivia | Both | 775.6 | 627.4 | 923.9 | 2030 |
| Bosnia and Herzegovina | Both | 445.6 | 394.7 | 496.5 | 2018 |
| Bosnia and Herzegovina | Both | 449.9 | 397.7 | 502 | 2019 |
| Bosnia and Herzegovina | Both | 454.8 | 400.9 | 508.8 | 2020 |
| Bosnia and Herzegovina | Both | 459.2 | 402.9 | 515.4 | 2021 |
| Bosnia and Herzegovina | Both | 463.9 | 404.7 | 523.1 | 2022 |
| Bosnia and Herzegovina | Both | 468.6 | 405.8 | 531.4 | 2023 |
| Bosnia and Herzegovina | Both | 473.1 | 406.1 | 540.1 | 2024 |
| Bosnia and Herzegovina | Both | 477.1 | 405.4 | 548.9 | 2025 |
| Bosnia and Herzegovina | Both | 480.1 | 403 | 557.2 | 2026 |
| Bosnia and Herzegovina | Both | 482.8 | 399.8 | 565.8 | 2027 |
| Bosnia and Herzegovina | Both | 485.4 | 395.9 | 574.9 | 2028 |
| Bosnia and Herzegovina | Both | 488 | 391.4 | 584.7 | 2029 |
| Bosnia and Herzegovina | Both | 490.8 | 386.3 | 595.3 | 2030 |
| Botswana | Both | 50.5 | 30.8 | 70.2 | 2018 |
| Botswana | Both | 52.9 | 32.6 | 73.2 | 2019 |
| Botswana | Both | 55.5 | 34.5 | 76.5 | 2020 |
| Botswana | Both | 58.3 | 36.5 | 80.1 | 2021 |
| Botswana | Both | 61.2 | 38.5 | 83.9 | 2022 |
| Botswana | Both | 64.4 | 40.6 | 88.1 | 2023 |
| Botswana | Both | 67.8 | 42.9 | 92.7 | 2024 |
| Botswana | Both | 71.4 | 45.1 | 97.8 | 2025 |
| Botswana | Both | 75.3 | 47.4 | 103.3 | 2026 |
| Botswana | Both | 79.5 | 49.7 | 109.4 | 2027 |
| Botswana | Both | 84.1 | 52 | 116.1 | 2028 |
| Botswana | Both | 88.9 | 54.3 | 123.6 | 2029 |
| Botswana | Both | 94.2 | 56.5 | 131.9 | 2030 |
| Brazil | Both | 11847.8 | 11530.8 | 12164.7 | 2018 |
| Brazil | Both | 12313.4 | 11962.4 | 12664.5 | 2019 |
| Brazil | Both | 12800.1 | 12397 | 13203.2 | 2020 |
| Brazil | Both | 13299.7 | 12825 | 13774.4 | 2021 |
| Brazil | Both | 13818.8 | 13252.2 | 14385.5 | 2022 |
| Brazil | Both | 14356.5 | 13677.1 | 15035.8 | 2023 |
| Brazil | Both | 14914.1 | 14101.7 | 15726.6 | 2024 |
| Brazil | Both | 15495.3 | 14529 | 16461.6 | 2025 |
| Brazil | Both | 16081.6 | 14941.1 | 17222.1 | 2026 |
| Brazil | Both | 16688.3 | 15351.3 | 18025.2 | 2027 |
| Brazil | Both | 17315.3 | 15758.3 | 18872.3 | 2028 |
| Brazil | Both | 17965 | 16163.3 | 19766.7 | 2029 |
| Brazil | Both | 18641 | 16568.3 | 20713.8 | 2030 |
| Bulgaria | Both | 684 | 615 | 753 | 2018 |
| Bulgaria | Both | 667.2 | 589.3 | 745.1 | 2019 |
| Bulgaria | Both | 652.7 | 560.9 | 744.6 | 2020 |
| Bulgaria | Both | 639.9 | 529.6 | 750.1 | 2021 |
| Bulgaria | Both | 630.2 | 497.4 | 763 | 2022 |
| Bulgaria | Both | 624 | 464.2 | 783.8 | 2023 |
| Bulgaria | Both | 621.5 | 429.2 | 813.7 | 2024 |
| Bulgaria | Both | 623 | 391.4 | 854.5 | 2025 |
| Bulgaria | Both | 628.8 | 348.5 | 909.1 | 2026 |
| Bulgaria | Both | 640.3 | 297.4 | 983.2 | 2027 |
| Bulgaria | Both | 658.9 | 232.6 | 1085.1 | 2028 |
| Bulgaria | Both | 686.2 | 144.7 | 1228 | 2029 |
| Bulgaria | Both | 724.7 | 17.7 | 1433.4 | 2030 |
| Burkina Faso | Both | 533.5 | 473 | 594 | 2018 |
| Burkina Faso | Both | 570.2 | 505.1 | 635.4 | 2019 |
| Burkina Faso | Both | 609.8 | 538.7 | 680.9 | 2020 |
| Burkina Faso | Both | 652.7 | 574 | 731.3 | 2021 |
| Burkina Faso | Both | 698.5 | 610.5 | 786.5 | 2022 |
| Burkina Faso | Both | 747.7 | 648.3 | 847.1 | 2023 |
| Burkina Faso | Both | 801 | 687.8 | 914.1 | 2024 |
| Burkina Faso | Both | 858.9 | 729.2 | 988.6 | 2025 |
| Burkina Faso | Both | 921.9 | 772.4 | 1071.4 | 2026 |
| Burkina Faso | Both | 989.9 | 816.9 | 1162.9 | 2027 |
| Burkina Faso | Both | 1063.6 | 862.8 | 1264.3 | 2028 |
| Burkina Faso | Both | 1143.5 | 910.1 | 1376.8 | 2029 |
| Burkina Faso | Both | 1230.5 | 958.8 | 1502.3 | 2030 |
| Burundi | Both | 169.1 | 137.2 | 201 | 2018 |
| Burundi | Both | 171.9 | 139 | 204.8 | 2019 |
| Burundi | Both | 174.6 | 140.4 | 208.8 | 2020 |
| Burundi | Both | 177.1 | 141.5 | 212.8 | 2021 |
| Burundi | Both | 179.5 | 142.2 | 216.8 | 2022 |
| Burundi | Both | 181.7 | 142.6 | 220.9 | 2023 |
| Burundi | Both | 184.1 | 142.8 | 225.3 | 2024 |
| Burundi | Both | 186.5 | 142.9 | 230.2 | 2025 |
| Burundi | Both | 189 | 142.8 | 235.3 | 2026 |
| Burundi | Both | 191.7 | 142.5 | 240.8 | 2027 |
| Burundi | Both | 194.6 | 142.2 | 246.9 | 2028 |
| Burundi | Both | 197.8 | 141.8 | 253.7 | 2029 |
| Burundi | Both | 201.4 | 141.3 | 261.4 | 2030 |
| Cambodia | Both | 415.2 | 359.5 | 471 | 2018 |
| Cambodia | Both | 429.8 | 372.2 | 487.3 | 2019 |
| Cambodia | Both | 444.5 | 384.7 | 504.3 | 2020 |
| Cambodia | Both | 459.1 | 396.4 | 521.7 | 2021 |
| Cambodia | Both | 473.8 | 407.8 | 539.8 | 2022 |
| Cambodia | Both | 489 | 418.9 | 559 | 2023 |
| Cambodia | Both | 505.2 | 430.3 | 580.1 | 2024 |
| Cambodia | Both | 522.9 | 442.1 | 603.6 | 2025 |
| Cambodia | Both | 541.5 | 453.9 | 629.1 | 2026 |
| Cambodia | Both | 561.4 | 465.8 | 657 | 2027 |
| Cambodia | Both | 582.3 | 477.5 | 687.1 | 2028 |
| Cambodia | Both | 604.1 | 488.8 | 719.4 | 2029 |
| Cambodia | Both | 626.6 | 499.4 | 753.8 | 2030 |
| Cameroon | Both | 830.8 | 750.3 | 911.3 | 2018 |
| Cameroon | Both | 856.3 | 771.4 | 941.2 | 2019 |
| Cameroon | Both | 883.3 | 792.8 | 973.8 | 2020 |
| Cameroon | Both | 911.4 | 813.8 | 1008.9 | 2021 |
| Cameroon | Both | 941 | 834.9 | 1047.1 | 2022 |
| Cameroon | Both | 972.4 | 856 | 1088.7 | 2023 |
| Cameroon | Both | 1005.8 | 877.3 | 1134.3 | 2024 |
| Cameroon | Both | 1041.5 | 898.8 | 1184.2 | 2025 |
| Cameroon | Both | 1078.8 | 919.7 | 1237.9 | 2026 |
| Cameroon | Both | 1118.3 | 940.5 | 1296.1 | 2027 |
| Cameroon | Both | 1160.5 | 961.3 | 1359.7 | 2028 |
| Cameroon | Both | 1205.6 | 982.2 | 1429.1 | 2029 |
| Cameroon | Both | 1254.3 | 1003.2 | 1505.3 | 2030 |
| Canada | Both | 4423.8 | 4242.5 | 4605.2 | 2018 |
| Canada | Both | 4478.9 | 4259.5 | 4698.3 | 2019 |
| Canada | Both | 4538.1 | 4260.4 | 4815.9 | 2020 |
| Canada | Both | 4593.7 | 4240.4 | 4947 | 2021 |
| Canada | Both | 4654.6 | 4210.2 | 5099 | 2022 |
| Canada | Both | 4722.3 | 4171.9 | 5272.7 | 2023 |
| Canada | Both | 4797 | 4125.6 | 5468.5 | 2024 |
| Canada | Both | 4879.6 | 4071.2 | 5687.9 | 2025 |
| Canada | Both | 4961.7 | 4000.6 | 5922.7 | 2026 |
| Canada | Both | 5054 | 3920.8 | 6187.1 | 2027 |
| Canada | Both | 5158.8 | 3830.7 | 6487 | 2028 |
| Canada | Both | 5278.7 | 3728 | 6829.4 | 2029 |
| Canada | Both | 5415.6 | 3608.9 | 7222.2 | 2030 |
| Central African Republic | Both | 102.1 | 76 | 128.3 | 2018 |
| Central African Republic | Both | 104.1 | 77.3 | 130.9 | 2019 |
| Central African Republic | Both | 106.4 | 78.8 | 134 | 2020 |
| Central African Republic | Both | 108.8 | 80.3 | 137.4 | 2021 |
| Central African Republic | Both | 111.7 | 82.1 | 141.3 | 2022 |
| Central African Republic | Both | 114.9 | 84 | 145.9 | 2023 |
| Central African Republic | Both | 118.5 | 86 | 150.9 | 2024 |
| Central African Republic | Both | 122.2 | 88 | 156.5 | 2025 |
| Central African Republic | Both | 126.2 | 90 | 162.5 | 2026 |
| Central African Republic | Both | 130.3 | 91.8 | 168.9 | 2027 |
| Central African Republic | Both | 134.6 | 93.4 | 175.7 | 2028 |
| Central African Republic | Both | 139.1 | 95 | 183.1 | 2029 |
| Central African Republic | Both | 143.9 | 96.5 | 191.4 | 2030 |
| Chad | Both | 321.6 | 275.9 | 367.3 | 2018 |
| Chad | Both | 336.5 | 288.2 | 384.8 | 2019 |
| Chad | Both | 352.3 | 300.7 | 403.8 | 2020 |
| Chad | Both | 369.1 | 313.6 | 424.7 | 2021 |
| Chad | Both | 386.7 | 326.4 | 447 | 2022 |
| Chad | Both | 405.1 | 339.2 | 471 | 2023 |
| Chad | Both | 424.6 | 352.1 | 497.2 | 2024 |
| Chad | Both | 445.4 | 365.2 | 525.7 | 2025 |
| Chad | Both | 467.7 | 378.5 | 557 | 2026 |
| Chad | Both | 491.1 | 391.5 | 590.7 | 2027 |
| Chad | Both | 515.8 | 404.4 | 627.3 | 2028 |
| Chad | Both | 542.1 | 417.1 | 667.2 | 2029 |
| Chad | Both | 570.4 | 429.7 | 711.2 | 2030 |
| Chile | Both | 2344.1 | 2214.6 | 2473.7 | 2018 |
| Chile | Both | 2418.7 | 2279.2 | 2558.1 | 2019 |
| Chile | Both | 2496.4 | 2342.1 | 2650.6 | 2020 |
| Chile | Both | 2573.1 | 2398.7 | 2747.5 | 2021 |
| Chile | Both | 2652.7 | 2452.5 | 2852.9 | 2022 |
| Chile | Both | 2735.9 | 2503.9 | 2967.9 | 2023 |
| Chile | Both | 2822.9 | 2553.2 | 3092.5 | 2024 |
| Chile | Both | 2913.8 | 2600.3 | 3227.3 | 2025 |
| Chile | Both | 3004.5 | 2641.2 | 3367.7 | 2026 |
| Chile | Both | 3098.9 | 2679.2 | 3518.6 | 2027 |
| Chile | Both | 3197.4 | 2714 | 3680.7 | 2028 |
| Chile | Both | 3299.7 | 2745 | 3854.4 | 2029 |
| Chile | Both | 3405.8 | 2771.3 | 4040.2 | 2030 |
| China | Both | 48453.4 | 47607 | 49299.8 | 2018 |
| China | Both | 48593.8 | 47303.8 | 49883.8 | 2019 |
| China | Both | 48879.5 | 46974.5 | 50784.6 | 2020 |
| China | Both | 49275.6 | 46626.3 | 51924.8 | 2021 |
| China | Both | 49489.3 | 46000.4 | 52978.3 | 2022 |
| China | Both | 49656.6 | 45219.7 | 54093.4 | 2023 |
| China | Both | 50002.7 | 44507.3 | 55498.2 | 2024 |
| China | Both | 50599 | 43918.9 | 57279.1 | 2025 |
| China | Both | 51323.5 | 43333.8 | 59313.3 | 2026 |
| China | Both | 51885.3 | 42496.5 | 61274.1 | 2027 |
| China | Both | 52383.9 | 41476 | 63291.8 | 2028 |
| China | Both | 53050.7 | 40455.4 | 65645.9 | 2029 |
| China | Both | 53956.8 | 39462 | 68451.5 | 2030 |
| Colombia | Both | 1936.5 | 1811.3 | 2061.6 | 2018 |
| Colombia | Both | 1998.4 | 1866.6 | 2130.2 | 2019 |
| Colombia | Both | 2062.5 | 1921.4 | 2203.7 | 2020 |
| Colombia | Both | 2126.4 | 1973 | 2279.9 | 2021 |
| Colombia | Both | 2192.6 | 2023.3 | 2361.8 | 2022 |
| Colombia | Both | 2261.1 | 2072.3 | 2449.9 | 2023 |
| Colombia | Both | 2332.3 | 2120.2 | 2544.4 | 2024 |
| Colombia | Both | 2406.4 | 2166.9 | 2646 | 2025 |
| Colombia | Both | 2480.4 | 2209.5 | 2751.2 | 2026 |
| Colombia | Both | 2557.3 | 2250.8 | 2863.7 | 2027 |
| Colombia | Both | 2637.3 | 2290.6 | 2984 | 2028 |
| Colombia | Both | 2720.7 | 2328.9 | 3112.5 | 2029 |
| Colombia | Both | 2807.6 | 2365.5 | 3249.7 | 2030 |
| Comoros | Both | 14.1 | 5.7 | 23.1 | 2018 |
| Comoros | Both | 14.5 | 5.9 | 23.7 | 2019 |
| Comoros | Both | 15 | 6.1 | 24.5 | 2020 |
| Comoros | Both | 15.5 | 6.3 | 25.3 | 2021 |
| Comoros | Both | 16 | 6.5 | 26.2 | 2022 |
| Comoros | Both | 16.7 | 6.6 | 27.3 | 2023 |
| Comoros | Both | 17.4 | 6.8 | 28.6 | 2024 |
| Comoros | Both | 18.2 | 7 | 30 | 2025 |
| Comoros | Both | 19 | 7.1 | 31.6 | 2026 |
| Comoros | Both | 20 | 7.1 | 33.5 | 2027 |
| Comoros | Both | 21.1 | 7.1 | 35.6 | 2028 |
| Comoros | Both | 22.3 | 6.9 | 38.2 | 2029 |
| Comoros | Both | 23.6 | 6.6 | 41.2 | 2030 |
| Congo | Both | 137.4 | 105.5 | 169.4 | 2018 |
| Congo | Both | 141.8 | 108.9 | 174.7 | 2019 |
| Congo | Both | 146.5 | 112.5 | 180.5 | 2020 |
| Congo | Both | 151.4 | 116 | 186.7 | 2021 |
| Congo | Both | 156.7 | 119.8 | 193.6 | 2022 |
| Congo | Both | 162.4 | 123.6 | 201.1 | 2023 |
| Congo | Both | 168.5 | 127.6 | 209.4 | 2024 |
| Congo | Both | 175.1 | 131.7 | 218.5 | 2025 |
| Congo | Both | 181.9 | 135.5 | 228.2 | 2026 |
| Congo | Both | 189.1 | 139.3 | 238.9 | 2027 |
| Congo | Both | 196.9 | 143.1 | 250.7 | 2028 |
| Congo | Both | 205.2 | 146.8 | 263.6 | 2029 |
| Congo | Both | 214 | 150.3 | 277.8 | 2030 |
| Costa Rica | Both | 264.3 | 220 | 308.6 | 2018 |
| Costa Rica | Both | 277.3 | 231.2 | 323.4 | 2019 |
| Costa Rica | Both | 291.1 | 242.7 | 339.5 | 2020 |
| Costa Rica | Both | 305.2 | 254 | 356.3 | 2021 |
| Costa Rica | Both | 320.1 | 265.5 | 374.6 | 2022 |
| Costa Rica | Both | 335.9 | 277.2 | 394.6 | 2023 |
| Costa Rica | Both | 352.6 | 288.9 | 416.4 | 2024 |
| Costa Rica | Both | 370.4 | 300.6 | 440.1 | 2025 |
| Costa Rica | Both | 388.7 | 311.9 | 465.5 | 2026 |
| Costa Rica | Both | 408.1 | 323 | 493.1 | 2027 |
| Costa Rica | Both | 428.7 | 334 | 523.4 | 2028 |
| Costa Rica | Both | 450.6 | 344.7 | 556.6 | 2029 |
| Costa Rica | Both | 474.1 | 355.1 | 593.2 | 2030 |
| Cote d'Ivoire | Both | 345.9 | 294.1 | 397.7 | 2018 |
| Cote d'Ivoire | Both | 360.1 | 306.1 | 414.1 | 2019 |
| Cote d'Ivoire | Both | 374.9 | 318.2 | 431.7 | 2020 |
| Cote d'Ivoire | Both | 390.8 | 330.6 | 450.9 | 2021 |
| Cote d'Ivoire | Both | 407.3 | 343 | 471.6 | 2022 |
| Cote d'Ivoire | Both | 424.7 | 355.4 | 494 | 2023 |
| Cote d'Ivoire | Both | 443 | 367.7 | 518.2 | 2024 |
| Cote d'Ivoire | Both | 462.2 | 380 | 544.5 | 2025 |
| Cote d'Ivoire | Both | 482.7 | 392.2 | 573.2 | 2026 |
| Cote d'Ivoire | Both | 504.3 | 404.1 | 604.4 | 2027 |
| Cote d'Ivoire | Both | 527.1 | 415.8 | 638.3 | 2028 |
| Cote d'Ivoire | Both | 551.2 | 427.2 | 675.3 | 2029 |
| Cote d'Ivoire | Both | 577 | 438.2 | 715.7 | 2030 |
| Croatia | Both | 976.7 | 895.7 | 1057.7 | 2018 |
| Croatia | Both | 939.2 | 845.1 | 1033.2 | 2019 |
| Croatia | Both | 906.8 | 793.6 | 1020.1 | 2020 |
| Croatia | Both | 879.1 | 741.7 | 1016.5 | 2021 |
| Croatia | Both | 856.2 | 690.2 | 1022.1 | 2022 |
| Croatia | Both | 837.6 | 638.8 | 1036.5 | 2023 |
| Croatia | Both | 823 | 586.2 | 1059.8 | 2024 |
| Croatia | Both | 812.7 | 531.5 | 1093.9 | 2025 |
| Croatia | Both | 807.3 | 472.5 | 1142.2 | 2026 |
| Croatia | Both | 808.1 | 406.2 | 1210.1 | 2027 |
| Croatia | Both | 816.5 | 327.1 | 1306 | 2028 |
| Croatia | Both | 834.1 | 225.7 | 1443 | 2029 |
| Croatia | Both | 863 | 85.7 | 1641.8 | 2030 |
| Cuba | Both | 868.7 | 789.1 | 948.3 | 2018 |
| Cuba | Both | 891.3 | 805.4 | 977.1 | 2019 |
| Cuba | Both | 915 | 819.6 | 1010.4 | 2020 |
| Cuba | Both | 939 | 830.3 | 1047.7 | 2021 |
| Cuba | Both | 964.1 | 838.3 | 1089.9 | 2022 |
| Cuba | Both | 990.7 | 843.8 | 1137.6 | 2023 |
| Cuba | Both | 1019.2 | 846.9 | 1191.4 | 2024 |
| Cuba | Both | 1049.9 | 847.7 | 1252.1 | 2025 |
| Cuba | Both | 1080.9 | 844 | 1317.9 | 2026 |
| Cuba | Both | 1114.6 | 837.1 | 1392.1 | 2027 |
| Cuba | Both | 1151.3 | 826.5 | 1476.2 | 2028 |
| Cuba | Both | 1191.7 | 811.1 | 1572.2 | 2029 |
| Cuba | Both | 1236.2 | 789.6 | 1682.8 | 2030 |
| Cyprus | Both | 81.9 | 60.1 | 104.1 | 2018 |
| Cyprus | Both | 85.7 | 62.9 | 108.8 | 2019 |
| Cyprus | Both | 89.6 | 65.9 | 113.8 | 2020 |
| Cyprus | Both | 93.7 | 68.7 | 119.1 | 2021 |
| Cyprus | Both | 98.1 | 71.7 | 124.9 | 2022 |
| Cyprus | Both | 102.8 | 74.7 | 131.4 | 2023 |
| Cyprus | Both | 107.9 | 77.7 | 138.6 | 2024 |
| Cyprus | Both | 113.5 | 80.7 | 146.6 | 2025 |
| Cyprus | Both | 119.2 | 83.5 | 155.3 | 2026 |
| Cyprus | Both | 125.4 | 86.2 | 165 | 2027 |
| Cyprus | Both | 132.2 | 88.8 | 176 | 2028 |
| Cyprus | Both | 139.6 | 91.2 | 188.4 | 2029 |
| Cyprus | Both | 147.7 | 93.3 | 202.5 | 2030 |
| Czech Republic | Both | 2584.4 | 2456.8 | 2712.1 | 2018 |
| Czech Republic | Both | 2578.2 | 2430.5 | 2725.9 | 2019 |
| Czech Republic | Both | 2571.8 | 2393.7 | 2749.9 | 2020 |
| Czech Republic | Both | 2564.9 | 2347.6 | 2782.2 | 2021 |
| Czech Republic | Both | 2559.2 | 2295 | 2823.3 | 2022 |
| Czech Republic | Both | 2554.9 | 2236.9 | 2872.9 | 2023 |
| Czech Republic | Both | 2552.1 | 2173.8 | 2930.4 | 2024 |
| Czech Republic | Both | 2551.4 | 2106.2 | 2996.7 | 2025 |
| Czech Republic | Both | 2553.6 | 2034.2 | 3072.9 | 2026 |
| Czech Republic | Both | 2558.9 | 1957.5 | 3160.4 | 2027 |
| Czech Republic | Both | 2567.9 | 1874.9 | 3260.8 | 2028 |
| Czech Republic | Both | 2581.7 | 1786 | 3377.4 | 2029 |
| Czech Republic | Both | 2602 | 1689.6 | 3514.4 | 2030 |
| Democratic Republic of the Congo | Both | 1292.1 | 1193.3 | 1390.9 | 2018 |
| Democratic Republic of the Congo | Both | 1328.1 | 1222 | 1434.2 | 2019 |
| Democratic Republic of the Congo | Both | 1366.3 | 1250.7 | 1481.8 | 2020 |
| Democratic Republic of the Congo | Both | 1404.8 | 1277.5 | 1532 | 2021 |
| Democratic Republic of the Congo | Both | 1445.1 | 1303.9 | 1586.4 | 2022 |
| Democratic Republic of the Congo | Both | 1488 | 1330.1 | 1645.8 | 2023 |
| Democratic Republic of the Congo | Both | 1533.6 | 1356.5 | 1710.8 | 2024 |
| Democratic Republic of the Congo | Both | 1582.6 | 1383.2 | 1782.1 | 2025 |
| Democratic Republic of the Congo | Both | 1632.7 | 1408 | 1857.4 | 2026 |
| Democratic Republic of the Congo | Both | 1685.6 | 1432.4 | 1938.8 | 2027 |
| Democratic Republic of the Congo | Both | 1741.9 | 1456.6 | 2027.3 | 2028 |
| Democratic Republic of the Congo | Both | 1802.3 | 1480.9 | 2123.7 | 2029 |
| Democratic Republic of the Congo | Both | 1867.3 | 1505.3 | 2229.2 | 2030 |
| Denmark | Both | 888.3 | 817.3 | 959.3 | 2018 |
| Denmark | Both | 895 | 820.8 | 969.3 | 2019 |
| Denmark | Both | 902.2 | 823.3 | 981.2 | 2020 |
| Denmark | Both | 907.5 | 822.2 | 992.7 | 2021 |
| Denmark | Both | 913.4 | 820.3 | 1006.6 | 2022 |
| Denmark | Both | 919.9 | 817.2 | 1022.6 | 2023 |
| Denmark | Both | 926.4 | 812.6 | 1040.1 | 2024 |
| Denmark | Both | 932.9 | 806.6 | 1059.2 | 2025 |
| Denmark | Both | 938.2 | 798 | 1078.4 | 2026 |
| Denmark | Both | 943.7 | 788.2 | 1099.1 | 2027 |
| Denmark | Both | 949.4 | 777.1 | 1121.7 | 2028 |
| Denmark | Both | 955.9 | 765.2 | 1146.5 | 2029 |
| Denmark | Both | 963.4 | 752.5 | 1174.3 | 2030 |
| Djibouti | Both | 26.1 | 13.9 | 39 | 2018 |
| Djibouti | Both | 27.4 | 14.6 | 40.7 | 2019 |
| Djibouti | Both | 28.8 | 15.3 | 42.8 | 2020 |
| Djibouti | Both | 30.2 | 16.1 | 45 | 2021 |
| Djibouti | Both | 31.9 | 16.9 | 47.5 | 2022 |
| Djibouti | Both | 33.8 | 17.7 | 50.4 | 2023 |
| Djibouti | Both | 35.9 | 18.5 | 53.7 | 2024 |
| Djibouti | Both | 38.2 | 19.3 | 57.6 | 2025 |
| Djibouti | Both | 40.7 | 20 | 62 | 2026 |
| Djibouti | Both | 43.6 | 20.5 | 67.2 | 2027 |
| Djibouti | Both | 46.8 | 20.8 | 73.3 | 2028 |
| Djibouti | Both | 50.4 | 20.8 | 80.5 | 2029 |
| Djibouti | Both | 54.4 | 20.1 | 89.3 | 2030 |
| Ecuador | Both | 782.1 | 699.8 | 864.4 | 2018 |
| Ecuador | Both | 809.3 | 719 | 899.6 | 2019 |
| Ecuador | Both | 838.5 | 735.4 | 941.7 | 2020 |
| Ecuador | Both | 869.3 | 748 | 990.6 | 2021 |
| Ecuador | Both | 902.9 | 757.5 | 1048.3 | 2022 |
| Ecuador | Both | 940.1 | 764 | 1116.2 | 2023 |
| Ecuador | Both | 981.6 | 767.4 | 1195.9 | 2024 |
| Ecuador | Both | 1028.5 | 767.2 | 1289.8 | 2025 |
| Ecuador | Both | 1080.1 | 761.3 | 1399 | 2026 |
| Ecuador | Both | 1139.1 | 748.9 | 1529.3 | 2027 |
| Ecuador | Both | 1206.7 | 727.2 | 1686.2 | 2028 |
| Ecuador | Both | 1284.8 | 692 | 1877.7 | 2029 |
| Ecuador | Both | 1375.7 | 636.4 | 2115 | 2030 |
| Egypt | Both | 2208.7 | 2071.7 | 2345.8 | 2018 |
| Egypt | Both | 2293.3 | 2147.9 | 2438.8 | 2019 |
| Egypt | Both | 2380.1 | 2223.3 | 2536.9 | 2020 |
| Egypt | Both | 2470.2 | 2298.4 | 2641.9 | 2021 |
| Egypt | Both | 2562.1 | 2371.3 | 2752.9 | 2022 |
| Egypt | Both | 2657 | 2442.6 | 2871.5 | 2023 |
| Egypt | Both | 2757.2 | 2514.3 | 3000 | 2024 |
| Egypt | Both | 2863.9 | 2587.5 | 3140.2 | 2025 |
| Egypt | Both | 2969.9 | 2655.2 | 3284.6 | 2026 |
| Egypt | Both | 3082.4 | 2723.7 | 3441.1 | 2027 |
| Egypt | Both | 3202.3 | 2793.4 | 3611.1 | 2028 |
| Egypt | Both | 3329.4 | 2863.8 | 3795 | 2029 |
| Egypt | Both | 3463.7 | 2934.2 | 3993.3 | 2030 |
| El Salvador | Both | 215.2 | 174.9 | 255.5 | 2018 |
| El Salvador | Both | 220.2 | 179.1 | 261.3 | 2019 |
| El Salvador | Both | 225.3 | 183.2 | 267.5 | 2020 |
| El Salvador | Both | 230.6 | 187.3 | 274 | 2021 |
| El Salvador | Both | 236.2 | 191.3 | 281.1 | 2022 |
| El Salvador | Both | 242 | 195.2 | 288.8 | 2023 |
| El Salvador | Both | 248 | 199 | 297 | 2024 |
| El Salvador | Both | 254.3 | 202.7 | 305.9 | 2025 |
| El Salvador | Both | 260.8 | 206.1 | 315.5 | 2026 |
| El Salvador | Both | 267.5 | 209.2 | 325.8 | 2027 |
| El Salvador | Both | 274.5 | 212 | 336.9 | 2028 |
| El Salvador | Both | 281.7 | 214.5 | 348.9 | 2029 |
| El Salvador | Both | 289.3 | 216.7 | 361.8 | 2030 |
| Equatorial Guinea | Both | 33 | 17.7 | 48.5 | 2018 |
| Equatorial Guinea | Both | 34.5 | 18.7 | 50.6 | 2019 |
| Equatorial Guinea | Both | 36.2 | 19.6 | 53 | 2020 |
| Equatorial Guinea | Both | 38 | 20.7 | 55.6 | 2021 |
| Equatorial Guinea | Both | 39.9 | 21.7 | 58.4 | 2022 |
| Equatorial Guinea | Both | 42 | 22.8 | 61.5 | 2023 |
| Equatorial Guinea | Both | 44.2 | 23.8 | 64.9 | 2024 |
| Equatorial Guinea | Both | 46.7 | 24.9 | 68.7 | 2025 |
| Equatorial Guinea | Both | 49.8 | 26.3 | 73.7 | 2026 |
| Equatorial Guinea | Both | 53.3 | 27.6 | 79.4 | 2027 |
| Equatorial Guinea | Both | 57.2 | 28.9 | 85.8 | 2028 |
| Equatorial Guinea | Both | 61.5 | 30.1 | 93.2 | 2029 |
| Equatorial Guinea | Both | 66.3 | 31.1 | 101.7 | 2030 |
| Eritrea | Both | 136 | 105.7 | 166.2 | 2018 |
| Eritrea | Both | 138.4 | 107.1 | 169.7 | 2019 |
| Eritrea | Both | 141 | 108.5 | 173.5 | 2020 |
| Eritrea | Both | 143.7 | 109.7 | 177.8 | 2021 |
| Eritrea | Both | 146.7 | 110.8 | 182.6 | 2022 |
| Eritrea | Both | 149.8 | 111.7 | 187.9 | 2023 |
| Eritrea | Both | 153.4 | 112.6 | 194.1 | 2024 |
| Eritrea | Both | 157.3 | 113.5 | 201.1 | 2025 |
| Eritrea | Both | 161.7 | 114.2 | 209.1 | 2026 |
| Eritrea | Both | 166.4 | 114.7 | 218.2 | 2027 |
| Eritrea | Both | 171.7 | 115 | 228.5 | 2028 |
| Eritrea | Both | 177.6 | 114.8 | 240.3 | 2029 |
| Eritrea | Both | 184.1 | 114.3 | 253.9 | 2030 |
| Estonia | Both | 296.1 | 255.9 | 336.7 | 2018 |
| Estonia | Both | 291.9 | 249.6 | 334.5 | 2019 |
| Estonia | Both | 288.3 | 242.8 | 334.2 | 2020 |
| Estonia | Both | 284.8 | 234.9 | 335.1 | 2021 |
| Estonia | Both | 282 | 226.5 | 337.8 | 2022 |
| Estonia | Both | 279.8 | 217.6 | 342.4 | 2023 |
| Estonia | Both | 278.1 | 208 | 348.6 | 2024 |
| Estonia | Both | 276.9 | 197.6 | 356.6 | 2025 |
| Estonia | Both | 276.3 | 186.3 | 366.7 | 2026 |
| Estonia | Both | 276.5 | 173.9 | 379.6 | 2027 |
| Estonia | Both | 277.8 | 160 | 396 | 2028 |
| Estonia | Both | 280.3 | 144 | 417.1 | 2029 |
| Estonia | Both | 284.3 | 125 | 444.2 | 2030 |
| Ethiopia | Both | 1843.2 | 1723.9 | 1962.5 | 2018 |
| Ethiopia | Both | 1860 | 1730.7 | 1989.3 | 2019 |
| Ethiopia | Both | 1880 | 1737.2 | 2022.8 | 2020 |
| Ethiopia | Both | 1900.9 | 1741.1 | 2060.7 | 2021 |
| Ethiopia | Both | 1925.1 | 1744.6 | 2105.6 | 2022 |
| Ethiopia | Both | 1952.6 | 1747.7 | 2157.6 | 2023 |
| Ethiopia | Both | 1983.6 | 1750.2 | 2217.1 | 2024 |
| Ethiopia | Both | 2018.4 | 1752.4 | 2284.4 | 2025 |
| Ethiopia | Both | 2055.4 | 1752.6 | 2358.2 | 2026 |
| Ethiopia | Both | 2096.4 | 1752.1 | 2440.7 | 2027 |
| Ethiopia | Both | 2142.3 | 1751.3 | 2533.3 | 2028 |
| Ethiopia | Both | 2193.8 | 1750.3 | 2637.4 | 2029 |
| Ethiopia | Both | 2251.9 | 1749 | 2754.9 | 2030 |
| Fiji | Both | 19 | 8.5 | 29.9 | 2018 |
| Fiji | Both | 19.4 | 8.8 | 30.4 | 2019 |
| Fiji | Both | 19.8 | 9 | 31 | 2020 |
| Fiji | Both | 20.1 | 9.1 | 31.6 | 2021 |
| Fiji | Both | 20.6 | 9.3 | 32.3 | 2022 |
| Fiji | Both | 21 | 9.4 | 33 | 2023 |
| Fiji | Both | 21.4 | 9.6 | 33.7 | 2024 |
| Fiji | Both | 21.9 | 9.7 | 34.6 | 2025 |
| Fiji | Both | 22.4 | 9.8 | 35.5 | 2026 |
| Fiji | Both | 23 | 9.9 | 36.5 | 2027 |
| Fiji | Both | 23.6 | 9.9 | 37.6 | 2028 |
| Fiji | Both | 24.2 | 9.9 | 38.9 | 2029 |
| Fiji | Both | 24.9 | 9.9 | 40.4 | 2030 |
| Finland | Both | 1023.7 | 947.8 | 1099.7 | 2018 |
| Finland | Both | 1031.7 | 953.1 | 1110.2 | 2019 |
| Finland | Both | 1039.9 | 957.6 | 1122.2 | 2020 |
| Finland | Both | 1047.6 | 960.4 | 1134.8 | 2021 |
| Finland | Both | 1055.4 | 962 | 1148.7 | 2022 |
| Finland | Both | 1063.1 | 962.3 | 1163.8 | 2023 |
| Finland | Both | 1070.5 | 961.2 | 1179.8 | 2024 |
| Finland | Both | 1077.6 | 958.6 | 1196.6 | 2025 |
| Finland | Both | 1083.5 | 953.8 | 1213.2 | 2026 |
| Finland | Both | 1088.9 | 947.4 | 1230.3 | 2027 |
| Finland | Both | 1094.1 | 939.9 | 1248.2 | 2028 |
| Finland | Both | 1099.4 | 931.4 | 1267.4 | 2029 |
| Finland | Both | 1105.1 | 922.2 | 1287.9 | 2030 |
| France | Both | 9126.6 | 8872.1 | 9381.2 | 2018 |
| France | Both | 9205.7 | 8920.2 | 9491.3 | 2019 |
| France | Both | 9297.7 | 8965.3 | 9630.1 | 2020 |
| France | Both | 9393.7 | 8999.5 | 9788 | 2021 |
| France | Both | 9499.7 | 9029.1 | 9970.3 | 2022 |
| France | Both | 9613.2 | 9052.5 | 10173.9 | 2023 |
| France | Both | 9724.5 | 9062.3 | 10386.7 | 2024 |
| France | Both | 9830.2 | 9056.4 | 10604 | 2025 |
| France | Both | 9917.1 | 9023 | 10811.2 | 2026 |
| France | Both | 10000.9 | 8976.7 | 11025.1 | 2027 |
| France | Both | 10089.5 | 8924.3 | 11254.8 | 2028 |
| France | Both | 10186.5 | 8869.3 | 11503.8 | 2029 |
| France | Both | 10294.2 | 8813.4 | 11775.1 | 2030 |
| Gabon | Both | 56 | 35.9 | 76.2 | 2018 |
| Gabon | Both | 57.4 | 36.9 | 78 | 2019 |
| Gabon | Both | 58.8 | 37.8 | 80 | 2020 |
| Gabon | Both | 60.4 | 38.8 | 82.1 | 2021 |
| Gabon | Both | 62 | 39.7 | 84.4 | 2022 |
| Gabon | Both | 63.8 | 40.7 | 87 | 2023 |
| Gabon | Both | 65.7 | 41.6 | 89.9 | 2024 |
| Gabon | Both | 67.7 | 42.6 | 93 | 2025 |
| Gabon | Both | 69.9 | 43.4 | 96.4 | 2026 |
| Gabon | Both | 72.1 | 44.2 | 100.1 | 2027 |
| Gabon | Both | 74.6 | 44.9 | 104.3 | 2028 |
| Gabon | Both | 77.3 | 45.5 | 109 | 2029 |
| Gabon | Both | 80.1 | 46 | 114.3 | 2030 |
| Georgia | Both | 392 | 340.4 | 443.6 | 2018 |
| Georgia | Both | 402.3 | 347.2 | 457.4 | 2019 |
| Georgia | Both | 413.3 | 353 | 473.5 | 2020 |
| Georgia | Both | 424.8 | 357.6 | 492.1 | 2021 |
| Georgia | Both | 436.9 | 360.7 | 513.2 | 2022 |
| Georgia | Both | 449.9 | 362.6 | 537.1 | 2023 |
| Georgia | Both | 463.9 | 363.2 | 564.6 | 2024 |
| Georgia | Both | 479.4 | 362.7 | 596.2 | 2025 |
| Georgia | Both | 495.6 | 359.8 | 631.4 | 2026 |
| Georgia | Both | 513.7 | 355.2 | 672.2 | 2027 |
| Georgia | Both | 534.1 | 348.2 | 720 | 2028 |
| Georgia | Both | 557.1 | 337.8 | 776.5 | 2029 |
| Georgia | Both | 583.2 | 322.6 | 843.9 | 2030 |
| Germany | Both | 17952.4 | 17592.5 | 18312.3 | 2018 |
| Germany | Both | 18125.8 | 17700.2 | 18551.3 | 2019 |
| Germany | Both | 18347.9 | 17827.4 | 18868.4 | 2020 |
| Germany | Both | 18615.1 | 17971.6 | 19258.5 | 2021 |
| Germany | Both | 18852 | 18060.3 | 19643.8 | 2022 |
| Germany | Both | 19079.4 | 18111.2 | 20047.6 | 2023 |
| Germany | Both | 19244.3 | 18084 | 20404.6 | 2024 |
| Germany | Both | 19385.4 | 18019.2 | 20751.6 | 2025 |
| Germany | Both | 19517.2 | 17930.6 | 21103.9 | 2026 |
| Germany | Both | 19672.5 | 17846.2 | 21498.8 | 2027 |
| Germany | Both | 19866.4 | 17775 | 21957.8 | 2028 |
| Germany | Both | 20152.7 | 17768 | 22537.4 | 2029 |
| Germany | Both | 20602.9 | 17884.9 | 23321 | 2030 |
| Ghana | Both | 762.6 | 679.8 | 845.3 | 2018 |
| Ghana | Both | 808.5 | 713.1 | 903.8 | 2019 |
| Ghana | Both | 859.2 | 744.6 | 973.7 | 2020 |
| Ghana | Both | 915.1 | 773.6 | 1056.6 | 2021 |
| Ghana | Both | 978.3 | 800.8 | 1155.9 | 2022 |
| Ghana | Both | 1050.4 | 826.1 | 1274.6 | 2023 |
| Ghana | Both | 1132.6 | 848.5 | 1416.7 | 2024 |
| Ghana | Both | 1226.9 | 866.6 | 1587.2 | 2025 |
| Ghana | Both | 1335.4 | 877.8 | 1793.1 | 2026 |
| Ghana | Both | 1461.6 | 878.4 | 2044.8 | 2027 |
| Ghana | Both | 1610 | 862.6 | 2357.5 | 2028 |
| Ghana | Both | 1786.4 | 820.1 | 2752.6 | 2029 |
| Ghana | Both | 1997.9 | 734.3 | 3261.5 | 2030 |
| Global | Both | 399538.8 | 396799.1 | 402278.5 | 2018 |
| Global | Both | 405393.2 | 401014.5 | 409771.9 | 2019 |
| Global | Both | 411758.9 | 405124.1 | 418393.8 | 2020 |
| Global | Both | 417903.7 | 408561.4 | 427246.1 | 2021 |
| Global | Both | 423881.3 | 411434 | 436328.5 | 2022 |
| Global | Both | 430028.8 | 414015.1 | 446042.4 | 2023 |
| Global | Both | 435861.3 | 415913.3 | 455809.3 | 2024 |
| Global | Both | 442156.2 | 417895 | 466417.4 | 2025 |
| Global | Both | 448073.4 | 419179.6 | 476967.1 | 2026 |
| Global | Both | 454097.2 | 420224.9 | 487969.5 | 2027 |
| Global | Both | 460449.3 | 421166.3 | 499732.4 | 2028 |
| Global | Both | 467349.7 | 422226.2 | 512473.2 | 2029 |
| Global | Both | 475387.6 | 423917.9 | 526857.3 | 2030 |
| Greece | Both | 1360.5 | 1269.2 | 1451.9 | 2018 |
| Greece | Both | 1371.4 | 1276.5 | 1466.2 | 2019 |
| Greece | Both | 1384 | 1284 | 1483.9 | 2020 |
| Greece | Both | 1396.9 | 1290.2 | 1503.6 | 2021 |
| Greece | Both | 1411.3 | 1295.9 | 1526.7 | 2022 |
| Greece | Both | 1427 | 1301 | 1552.9 | 2023 |
| Greece | Both | 1443.2 | 1304.9 | 1581.6 | 2024 |
| Greece | Both | 1460 | 1307.4 | 1612.5 | 2025 |
| Greece | Both | 1476.6 | 1308.2 | 1645.1 | 2026 |
| Greece | Both | 1493.5 | 1307.4 | 1679.6 | 2027 |
| Greece | Both | 1510.7 | 1305.2 | 1716.1 | 2028 |
| Greece | Both | 1528.5 | 1301.9 | 1755.1 | 2029 |
| Greece | Both | 1547.5 | 1297.9 | 1797.2 | 2030 |
| Guatemala | Both | 503.1 | 434.2 | 572 | 2018 |
| Guatemala | Both | 530.6 | 450.8 | 610.5 | 2019 |
| Guatemala | Both | 562 | 463.6 | 660.4 | 2020 |
| Guatemala | Both | 597.7 | 471.5 | 724 | 2021 |
| Guatemala | Both | 640.1 | 474.5 | 805.7 | 2022 |
| Guatemala | Both | 691.1 | 471 | 911.3 | 2023 |
| Guatemala | Both | 753.6 | 457.3 | 1050 | 2024 |
| Guatemala | Both | 831.4 | 426 | 1236.7 | 2025 |
| Guatemala | Both | 928.9 | 362.3 | 1495.6 | 2026 |
| Guatemala | Both | 1054.2 | 237.2 | 1871.3 | 2027 |
| Guatemala | Both | 1218.4 | 0 | 2445.4 | 2028 |
| Guatemala | Both | 1438 | 0 | 3376.5 | 2029 |
| Guatemala | Both | 1739.7 | 0 | 4987.9 | 2030 |
| Guinea | Both | 214.4 | 175.3 | 253.5 | 2018 |
| Guinea | Both | 222.2 | 181.6 | 262.7 | 2019 |
| Guinea | Both | 230.4 | 188.1 | 272.6 | 2020 |
| Guinea | Both | 239.3 | 195 | 283.7 | 2021 |
| Guinea | Both | 248.7 | 201.8 | 295.6 | 2022 |
| Guinea | Both | 258.5 | 208.7 | 308.3 | 2023 |
| Guinea | Both | 268.9 | 215.6 | 322.1 | 2024 |
| Guinea | Both | 279.9 | 222.7 | 337.1 | 2025 |
| Guinea | Both | 291.8 | 229.9 | 353.7 | 2026 |
| Guinea | Both | 304.3 | 237 | 371.7 | 2027 |
| Guinea | Both | 317.5 | 244 | 391 | 2028 |
| Guinea | Both | 331.4 | 250.8 | 412 | 2029 |
| Guinea | Both | 346.2 | 257.4 | 434.9 | 2030 |
| Guinea-Bissau | Both | 55.7 | 36.1 | 75.5 | 2018 |
| Guinea-Bissau | Both | 57.8 | 37.5 | 78.3 | 2019 |
| Guinea-Bissau | Both | 60.1 | 39 | 81.3 | 2020 |
| Guinea-Bissau | Both | 62.5 | 40.5 | 84.6 | 2021 |
| Guinea-Bissau | Both | 65.1 | 42 | 88.2 | 2022 |
| Guinea-Bissau | Both | 67.9 | 43.6 | 92.3 | 2023 |
| Guinea-Bissau | Both | 71 | 45.3 | 96.8 | 2024 |
| Guinea-Bissau | Both | 74.4 | 47.1 | 101.9 | 2025 |
| Guinea-Bissau | Both | 78.1 | 48.9 | 107.4 | 2026 |
| Guinea-Bissau | Both | 82 | 50.6 | 113.6 | 2027 |
| Guinea-Bissau | Both | 86.4 | 52.3 | 120.6 | 2028 |
| Guinea-Bissau | Both | 91.2 | 54.1 | 128.5 | 2029 |
| Guinea-Bissau | Both | 96.6 | 55.7 | 137.5 | 2030 |
| Guyana | Both | 38.6 | 21.5 | 55.7 | 2018 |
| Guyana | Both | 39.4 | 22.1 | 56.8 | 2019 |
| Guyana | Both | 40.2 | 22.6 | 57.8 | 2020 |
| Guyana | Both | 41.1 | 23.1 | 59 | 2021 |
| Guyana | Both | 41.9 | 23.6 | 60.2 | 2022 |
| Guyana | Both | 42.8 | 24.1 | 61.5 | 2023 |
| Guyana | Both | 43.7 | 24.5 | 62.9 | 2024 |
| Guyana | Both | 44.6 | 24.9 | 64.4 | 2025 |
| Guyana | Both | 45.6 | 25.2 | 66 | 2026 |
| Guyana | Both | 46.6 | 25.4 | 67.7 | 2027 |
| Guyana | Both | 47.6 | 25.6 | 69.6 | 2028 |
| Guyana | Both | 48.7 | 25.8 | 71.6 | 2029 |
| Guyana | Both | 49.8 | 25.8 | 73.9 | 2030 |
| Haiti | Both | 465.7 | 404.4 | 527 | 2018 |
| Haiti | Both | 474.5 | 411.4 | 537.6 | 2019 |
| Haiti | Both | 483.8 | 418.3 | 549.3 | 2020 |
| Haiti | Both | 493.3 | 424.8 | 561.8 | 2021 |
| Haiti | Both | 503.3 | 431.2 | 575.5 | 2022 |
| Haiti | Both | 513.9 | 437.3 | 590.6 | 2023 |
| Haiti | Both | 525.1 | 443.1 | 607.2 | 2024 |
| Haiti | Both | 537 | 448.7 | 625.3 | 2025 |
| Haiti | Both | 548.9 | 453.3 | 644.6 | 2026 |
| Haiti | Both | 561.6 | 457.6 | 665.5 | 2027 |
| Haiti | Both | 574.9 | 461.4 | 688.4 | 2028 |
| Haiti | Both | 588.9 | 464.6 | 713.2 | 2029 |
| Haiti | Both | 603.7 | 467.3 | 740.2 | 2030 |
| Honduras | Both | 253.3 | 208.1 | 298.4 | 2018 |
| Honduras | Both | 264.1 | 217.5 | 310.8 | 2019 |
| Honduras | Both | 275.7 | 227.2 | 324.2 | 2020 |
| Honduras | Both | 287.4 | 236.8 | 338.1 | 2021 |
| Honduras | Both | 299.9 | 246.7 | 353.1 | 2022 |
| Honduras | Both | 313.1 | 256.8 | 369.4 | 2023 |
| Honduras | Both | 327 | 267.1 | 387 | 2024 |
| Honduras | Both | 341.7 | 277.4 | 406 | 2025 |
| Honduras | Both | 356.6 | 287.2 | 426 | 2026 |
| Honduras | Both | 372.3 | 297 | 447.6 | 2027 |
| Honduras | Both | 388.8 | 306.7 | 471 | 2028 |
| Honduras | Both | 406.3 | 316.2 | 496.3 | 2029 |
| Honduras | Both | 424.7 | 325.6 | 523.9 | 2030 |
| Hungary | Both | 1720.7 | 1620.4 | 1820.9 | 2018 |
| Hungary | Both | 1678.4 | 1573.8 | 1783 | 2019 |
| Hungary | Both | 1636.7 | 1525.2 | 1748.2 | 2020 |
| Hungary | Both | 1595 | 1474.4 | 1715.7 | 2021 |
| Hungary | Both | 1554.1 | 1422.4 | 1685.8 | 2022 |
| Hungary | Both | 1513.9 | 1369.6 | 1658.2 | 2023 |
| Hungary | Both | 1474.3 | 1316.2 | 1632.4 | 2024 |
| Hungary | Both | 1435.2 | 1262.5 | 1607.9 | 2025 |
| Hungary | Both | 1396.8 | 1208.8 | 1584.8 | 2026 |
| Hungary | Both | 1358.9 | 1155.1 | 1562.7 | 2027 |
| Hungary | Both | 1321.6 | 1101.5 | 1541.6 | 2028 |
| Hungary | Both | 1284.7 | 1048.1 | 1521.2 | 2029 |
| Hungary | Both | 1248.3 | 995 | 1501.6 | 2030 |
| Iceland | Both | 66.3 | 47.3 | 85.7 | 2018 |
| Iceland | Both | 67.7 | 48.4 | 87.6 | 2019 |
| Iceland | Both | 69.3 | 49.5 | 89.6 | 2020 |
| Iceland | Both | 70.8 | 50.5 | 91.6 | 2021 |
| Iceland | Both | 72.3 | 51.4 | 93.8 | 2022 |
| Iceland | Both | 74 | 52.4 | 96.1 | 2023 |
| Iceland | Both | 75.7 | 53.3 | 98.6 | 2024 |
| Iceland | Both | 77.5 | 54.1 | 101.4 | 2025 |
| Iceland | Both | 79.3 | 54.8 | 104.3 | 2026 |
| Iceland | Both | 81.1 | 55.3 | 107.4 | 2027 |
| Iceland | Both | 83 | 55.8 | 110.7 | 2028 |
| Iceland | Both | 85 | 56.2 | 114.4 | 2029 |
| Iceland | Both | 87.2 | 56.4 | 118.5 | 2030 |
| India | Both | 23289 | 22810 | 23767.9 | 2018 |
| India | Both | 24301.6 | 23724.1 | 24879.1 | 2019 |
| India | Both | 25395 | 24664.9 | 26125.2 | 2020 |
| India | Both | 26495.6 | 25560.5 | 27430.7 | 2021 |
| India | Both | 27642.6 | 26450.5 | 28834.7 | 2022 |
| India | Both | 28853.1 | 27349.5 | 30356.7 | 2023 |
| India | Both | 30149.3 | 28280 | 32018.5 | 2024 |
| India | Both | 31532.5 | 29241.3 | 33823.6 | 2025 |
| India | Both | 32936.1 | 30168.3 | 35704 | 2026 |
| India | Both | 34374.2 | 31069.8 | 37678.6 | 2027 |
| India | Both | 35872.6 | 31963.4 | 39781.9 | 2028 |
| India | Both | 37473.6 | 32883.7 | 42063.5 | 2029 |
| India | Both | 39186.6 | 33832.6 | 44540.6 | 2030 |
| Indonesia | Both | 7618.2 | 7364 | 7872.4 | 2018 |
| Indonesia | Both | 7828 | 7549.5 | 8106.4 | 2019 |
| Indonesia | Both | 8044 | 7728.7 | 8359.4 | 2020 |
| Indonesia | Both | 8268.7 | 7902.7 | 8634.7 | 2021 |
| Indonesia | Both | 8498.7 | 8068.2 | 8929.1 | 2022 |
| Indonesia | Both | 8736.3 | 8227.5 | 9245.1 | 2023 |
| Indonesia | Both | 8983 | 8382.6 | 9583.5 | 2024 |
| Indonesia | Both | 9239.8 | 8534.7 | 9945 | 2025 |
| Indonesia | Both | 9496.5 | 8674 | 10319.1 | 2026 |
| Indonesia | Both | 9762 | 8808.4 | 10715.7 | 2027 |
| Indonesia | Both | 10037.5 | 8938.1 | 11136.8 | 2028 |
| Indonesia | Both | 10320.2 | 9060.5 | 11579.9 | 2029 |
| Indonesia | Both | 10609.9 | 9174.6 | 12045.2 | 2030 |
| Iran | Both | 2414.7 | 2265.9 | 2563.6 | 2018 |
| Iran | Both | 2426.7 | 2264.7 | 2588.7 | 2019 |
| Iran | Both | 2442.7 | 2260.2 | 2625.2 | 2020 |
| Iran | Both | 2463.9 | 2253.2 | 2674.6 | 2021 |
| Iran | Both | 2488.6 | 2242.2 | 2734.9 | 2022 |
| Iran | Both | 2518.1 | 2228.9 | 2807.3 | 2023 |
| Iran | Both | 2553.7 | 2214.6 | 2892.8 | 2024 |
| Iran | Both | 2595.7 | 2199.5 | 2992 | 2025 |
| Iran | Both | 2642.1 | 2181.3 | 3102.9 | 2026 |
| Iran | Both | 2694.2 | 2160.6 | 3227.9 | 2027 |
| Iran | Both | 2752.9 | 2137 | 3368.7 | 2028 |
| Iran | Both | 2818.2 | 2109.5 | 3526.8 | 2029 |
| Iran | Both | 2890.6 | 2076.9 | 3704.2 | 2030 |
| Iraq | Both | 943.2 | 853.8 | 1032.7 | 2018 |
| Iraq | Both | 966.4 | 872.8 | 1059.9 | 2019 |
| Iraq | Both | 990.1 | 891.2 | 1089.1 | 2020 |
| Iraq | Both | 1013.7 | 907.9 | 1119.4 | 2021 |
| Iraq | Both | 1037.9 | 923.8 | 1152.1 | 2022 |
| Iraq | Both | 1062.9 | 938.6 | 1187.2 | 2023 |
| Iraq | Both | 1089.1 | 952.7 | 1225.5 | 2024 |
| Iraq | Both | 1117.1 | 966.6 | 1267.6 | 2025 |
| Iraq | Both | 1146.2 | 979.5 | 1312.9 | 2026 |
| Iraq | Both | 1177.3 | 992 | 1362.5 | 2027 |
| Iraq | Both | 1210.2 | 1003.9 | 1416.5 | 2028 |
| Iraq | Both | 1244.9 | 1014.9 | 1474.8 | 2029 |
| Iraq | Both | 1281.6 | 1025 | 1538.2 | 2030 |
| Ireland | Both | 561.7 | 501.1 | 622.2 | 2018 |
| Ireland | Both | 568.3 | 505.6 | 631 | 2019 |
| Ireland | Both | 575.4 | 509.8 | 641 | 2020 |
| Ireland | Both | 581.8 | 512.2 | 651.4 | 2021 |
| Ireland | Both | 588.8 | 514.1 | 663.4 | 2022 |
| Ireland | Both | 596.2 | 515.4 | 677.1 | 2023 |
| Ireland | Both | 604.3 | 516 | 692.5 | 2024 |
| Ireland | Both | 612.8 | 515.9 | 709.7 | 2025 |
| Ireland | Both | 620.9 | 514.1 | 727.6 | 2026 |
| Ireland | Both | 629.5 | 511.6 | 747.3 | 2027 |
| Ireland | Both | 638.7 | 508.4 | 769.1 | 2028 |
| Ireland | Both | 648.8 | 504.4 | 793.2 | 2029 |
| Ireland | Both | 659.9 | 499.7 | 820.1 | 2030 |
| Israel | Both | 679.2 | 611.9 | 746.5 | 2018 |
| Israel | Both | 689.6 | 620.1 | 759.2 | 2019 |
| Israel | Both | 700.2 | 627.6 | 772.8 | 2020 |
| Israel | Both | 710.9 | 634.2 | 787.6 | 2021 |
| Israel | Both | 721.4 | 639.5 | 803.2 | 2022 |
| Israel | Both | 732 | 643.9 | 820.1 | 2023 |
| Israel | Both | 743.3 | 647.7 | 838.8 | 2024 |
| Israel | Both | 755.5 | 651.2 | 859.8 | 2025 |
| Israel | Both | 766.6 | 652.4 | 880.8 | 2026 |
| Israel | Both | 778.5 | 653.2 | 903.9 | 2027 |
| Israel | Both | 791.3 | 653.4 | 929.3 | 2028 |
| Israel | Both | 805 | 652.9 | 957.1 | 2029 |
| Israel | Both | 819.6 | 651.8 | 987.5 | 2030 |
| Italy | Both | 10703.8 | 10431.3 | 10976.3 | 2018 |
| Italy | Both | 10662.2 | 10355.2 | 10969.1 | 2019 |
| Italy | Both | 10641.2 | 10282 | 11000.4 | 2020 |
| Italy | Both | 10619.8 | 10192.5 | 11047 | 2021 |
| Italy | Both | 10606 | 10096.7 | 11115.4 | 2022 |
| Italy | Both | 10587.1 | 9983.8 | 11190.5 | 2023 |
| Italy | Both | 10551.2 | 9845.4 | 11257 | 2024 |
| Italy | Both | 10503.7 | 9688.3 | 11319 | 2025 |
| Italy | Both | 10437.6 | 9507.1 | 11368.2 | 2026 |
| Italy | Both | 10380.9 | 9327.7 | 11434 | 2027 |
| Italy | Both | 10340.4 | 9156 | 11524.7 | 2028 |
| Italy | Both | 10318 | 8993.8 | 11642.3 | 2029 |
| Italy | Both | 10312.6 | 8839.6 | 11785.7 | 2030 |
| Jamaica | Both | 119.1 | 88.9 | 149.4 | 2018 |
| Jamaica | Both | 123.8 | 92.6 | 155 | 2019 |
| Jamaica | Both | 128.8 | 96.4 | 161.2 | 2020 |
| Jamaica | Both | 133.9 | 100 | 167.8 | 2021 |
| Jamaica | Both | 139.5 | 103.7 | 175.3 | 2022 |
| Jamaica | Both | 145.6 | 107.4 | 183.7 | 2023 |
| Jamaica | Both | 152 | 111 | 193 | 2024 |
| Jamaica | Both | 158.8 | 114.4 | 203.3 | 2025 |
| Jamaica | Both | 166.2 | 117.5 | 214.9 | 2026 |
| Jamaica | Both | 174 | 120.2 | 227.8 | 2027 |
| Jamaica | Both | 182.4 | 122.5 | 242.3 | 2028 |
| Jamaica | Both | 191.4 | 124.1 | 258.8 | 2029 |
| Jamaica | Both | 201.4 | 125.1 | 277.7 | 2030 |
| Japan | Both | 13120.5 | 12822.4 | 13418.5 | 2018 |
| Japan | Both | 13125.4 | 12790.4 | 13460.5 | 2019 |
| Japan | Both | 13137.8 | 12750.3 | 13525.2 | 2020 |
| Japan | Both | 13156.7 | 12701.5 | 13611.8 | 2021 |
| Japan | Both | 13187.8 | 12649.7 | 13726 | 2022 |
| Japan | Both | 13229.1 | 12592 | 13866.2 | 2023 |
| Japan | Both | 13261.1 | 12515 | 14007.1 | 2024 |
| Japan | Both | 13243.7 | 12383.4 | 14104 | 2025 |
| Japan | Both | 13175.8 | 12197.2 | 14154.5 | 2026 |
| Japan | Both | 13131.9 | 12025.5 | 14238.4 | 2027 |
| Japan | Both | 13153.5 | 11904 | 14402.9 | 2028 |
| Japan | Both | 13234.5 | 11828.1 | 14640.9 | 2029 |
| Japan | Both | 13390.9 | 11810.5 | 14971.4 | 2030 |
| Jordan | Both | 200.7 | 160.6 | 240.8 | 2018 |
| Jordan | Both | 210.3 | 168.6 | 251.9 | 2019 |
| Jordan | Both | 219.8 | 176.5 | 263.1 | 2020 |
| Jordan | Both | 229.5 | 184.2 | 274.8 | 2021 |
| Jordan | Both | 239 | 191.4 | 286.7 | 2022 |
| Jordan | Both | 248.6 | 198.3 | 299 | 2023 |
| Jordan | Both | 258.7 | 205.2 | 312.2 | 2024 |
| Jordan | Both | 269.5 | 212.2 | 326.7 | 2025 |
| Jordan | Both | 281.2 | 219.4 | 343 | 2026 |
| Jordan | Both | 293.9 | 226.7 | 361 | 2027 |
| Jordan | Both | 307.6 | 234.2 | 381.1 | 2028 |
| Jordan | Both | 322.4 | 241.6 | 403.2 | 2029 |
| Jordan | Both | 338.4 | 249 | 427.7 | 2030 |
| Kazakhstan | Both | 1373.4 | 1267.5 | 1479.4 | 2018 |
| Kazakhstan | Both | 1381 | 1262.1 | 1499.9 | 2019 |
| Kazakhstan | Both | 1395.9 | 1257.7 | 1534.2 | 2020 |
| Kazakhstan | Both | 1416.8 | 1252.4 | 1581.1 | 2021 |
| Kazakhstan | Both | 1435.6 | 1238.3 | 1632.8 | 2022 |
| Kazakhstan | Both | 1453.2 | 1215.8 | 1690.5 | 2023 |
| Kazakhstan | Both | 1474.6 | 1189.7 | 1759.5 | 2024 |
| Kazakhstan | Both | 1505.4 | 1163.7 | 1847.1 | 2025 |
| Kazakhstan | Both | 1539.9 | 1130.9 | 1948.8 | 2026 |
| Kazakhstan | Both | 1579 | 1089.4 | 2068.6 | 2027 |
| Kazakhstan | Both | 1623.3 | 1035.8 | 2210.8 | 2028 |
| Kazakhstan | Both | 1676.2 | 970 | 2384.4 | 2029 |
| Kazakhstan | Both | 1742.5 | 895.6 | 2603.5 | 2030 |
| Kenya | Both | 462.6 | 402.4 | 522.8 | 2018 |
| Kenya | Both | 478.4 | 415.8 | 540.9 | 2019 |
| Kenya | Both | 495 | 429.6 | 560.4 | 2020 |
| Kenya | Both | 512.1 | 443.1 | 581 | 2021 |
| Kenya | Both | 529.9 | 456.7 | 603.2 | 2022 |
| Kenya | Both | 548.8 | 470.4 | 627.2 | 2023 |
| Kenya | Both | 568.6 | 484.1 | 653.2 | 2024 |
| Kenya | Both | 589.7 | 497.9 | 681.4 | 2025 |
| Kenya | Both | 611.2 | 511.1 | 711.3 | 2026 |
| Kenya | Both | 633.9 | 524.2 | 743.7 | 2027 |
| Kenya | Both | 658 | 537.2 | 778.9 | 2028 |
| Kenya | Both | 683.6 | 550.2 | 817.1 | 2029 |
| Kenya | Both | 710.8 | 562.9 | 858.6 | 2030 |
| Kuwait | Both | 81.2 | 57.2 | 105.4 | 2018 |
| Kuwait | Both | 83.6 | 58.9 | 108.4 | 2019 |
| Kuwait | Both | 86.1 | 60.6 | 111.8 | 2020 |
| Kuwait | Both | 89.1 | 62.6 | 115.9 | 2021 |
| Kuwait | Both | 92.4 | 64.6 | 120.5 | 2022 |
| Kuwait | Both | 95.9 | 66.4 | 125.6 | 2023 |
| Kuwait | Both | 99.5 | 68.1 | 131.2 | 2024 |
| Kuwait | Both | 103.3 | 69.4 | 137.4 | 2025 |
| Kuwait | Both | 107.5 | 70.5 | 144.7 | 2026 |
| Kuwait | Both | 112 | 71.3 | 153 | 2027 |
| Kuwait | Both | 116.8 | 71.4 | 162.4 | 2028 |
| Kuwait | Both | 122 | 70.8 | 173.3 | 2029 |
| Kuwait | Both | 127.6 | 69.3 | 186.2 | 2030 |
| Kyrgyzstan | Both | 238.2 | 196 | 280.4 | 2018 |
| Kyrgyzstan | Both | 243.4 | 199.2 | 287.6 | 2019 |
| Kyrgyzstan | Both | 248.8 | 201.6 | 296 | 2020 |
| Kyrgyzstan | Both | 254.7 | 203.3 | 306 | 2021 |
| Kyrgyzstan | Both | 260.8 | 204 | 317.7 | 2022 |
| Kyrgyzstan | Both | 267.6 | 203.8 | 331.3 | 2023 |
| Kyrgyzstan | Both | 275.1 | 202.6 | 347.7 | 2024 |
| Kyrgyzstan | Both | 283.7 | 200.2 | 367.2 | 2025 |
| Kyrgyzstan | Both | 293 | 196 | 390 | 2026 |
| Kyrgyzstan | Both | 303.7 | 189.7 | 417.6 | 2027 |
| Kyrgyzstan | Both | 316 | 180.5 | 451.5 | 2028 |
| Kyrgyzstan | Both | 330.3 | 167 | 493.6 | 2029 |
| Kyrgyzstan | Both | 346.8 | 146.9 | 546.7 | 2030 |
| Laos | Both | 197.1 | 158 | 236.1 | 2018 |
| Laos | Both | 201.5 | 161.5 | 241.5 | 2019 |
| Laos | Both | 206.1 | 165.1 | 247.2 | 2020 |
| Laos | Both | 210.8 | 168.4 | 253.2 | 2021 |
| Laos | Both | 215.7 | 171.8 | 259.6 | 2022 |
| Laos | Both | 220.8 | 175.1 | 266.5 | 2023 |
| Laos | Both | 226.2 | 178.3 | 274.1 | 2024 |
| Laos | Both | 232 | 181.6 | 282.3 | 2025 |
| Laos | Both | 237.9 | 184.6 | 291.1 | 2026 |
| Laos | Both | 244.1 | 187.5 | 300.7 | 2027 |
| Laos | Both | 250.7 | 190.2 | 311.2 | 2028 |
| Laos | Both | 257.7 | 192.8 | 322.6 | 2029 |
| Laos | Both | 265.1 | 195.2 | 335 | 2030 |
| Latvia | Both | 376.9 | 330.7 | 423.4 | 2018 |
| Latvia | Both | 370 | 322.5 | 417.9 | 2019 |
| Latvia | Both | 363.7 | 313.9 | 413.8 | 2020 |
| Latvia | Both | 357.4 | 304.5 | 410.6 | 2021 |
| Latvia | Both | 351.8 | 294.9 | 408.9 | 2022 |
| Latvia | Both | 346.8 | 285.1 | 408.7 | 2023 |
| Latvia | Both | 342.3 | 275.1 | 409.8 | 2024 |
| Latvia | Both | 338.5 | 264.9 | 412.3 | 2025 |
| Latvia | Both | 335.1 | 254.4 | 416.2 | 2026 |
| Latvia | Both | 332.4 | 243.6 | 421.6 | 2027 |
| Latvia | Both | 330.4 | 232.5 | 428.8 | 2028 |
| Latvia | Both | 329.1 | 220.8 | 437.7 | 2029 |
| Latvia | Both | 328.3 | 208.2 | 448.7 | 2030 |
| Lebanon | Both | 223.6 | 182 | 265.3 | 2018 |
| Lebanon | Both | 226.8 | 184.3 | 269.3 | 2019 |
| Lebanon | Both | 229.6 | 186.2 | 273.1 | 2020 |
| Lebanon | Both | 233.4 | 188.6 | 278.2 | 2021 |
| Lebanon | Both | 236.5 | 190.2 | 282.9 | 2022 |
| Lebanon | Both | 239.5 | 191.3 | 287.6 | 2023 |
| Lebanon | Both | 242.7 | 192.4 | 293 | 2024 |
| Lebanon | Both | 246.7 | 193.8 | 299.6 | 2025 |
| Lebanon | Both | 250.3 | 194.4 | 306.2 | 2026 |
| Lebanon | Both | 255 | 195.5 | 314.4 | 2027 |
| Lebanon | Both | 260.6 | 196.9 | 324.3 | 2028 |
| Lebanon | Both | 267 | 198.4 | 335.7 | 2029 |
| Lebanon | Both | 274.1 | 199.7 | 348.6 | 2030 |
| Lesotho | Both | 56.2 | 35.3 | 77.1 | 2018 |
| Lesotho | Both | 57.3 | 36 | 78.6 | 2019 |
| Lesotho | Both | 58.5 | 36.7 | 80.2 | 2020 |
| Lesotho | Both | 59.8 | 37.5 | 82.1 | 2021 |
| Lesotho | Both | 61.3 | 38.3 | 84.3 | 2022 |
| Lesotho | Both | 62.9 | 39.1 | 86.6 | 2023 |
| Lesotho | Both | 64.6 | 39.9 | 89.2 | 2024 |
| Lesotho | Both | 66.4 | 40.7 | 92.1 | 2025 |
| Lesotho | Both | 68.5 | 41.5 | 95.6 | 2026 |
| Lesotho | Both | 70.9 | 42.3 | 99.5 | 2027 |
| Lesotho | Both | 73.4 | 43 | 103.8 | 2028 |
| Lesotho | Both | 76.1 | 43.5 | 108.7 | 2029 |
| Lesotho | Both | 79.1 | 43.9 | 114.3 | 2030 |
| Liberia | Both | 107.4 | 79.3 | 135.6 | 2018 |
| Liberia | Both | 112.9 | 83.4 | 142.4 | 2019 |
| Liberia | Both | 118.8 | 87.6 | 149.9 | 2020 |
| Liberia | Both | 125.2 | 92 | 158.4 | 2021 |
| Liberia | Both | 132.1 | 96.4 | 167.7 | 2022 |
| Liberia | Both | 139.6 | 100.9 | 178.2 | 2023 |
| Liberia | Both | 147.7 | 105.4 | 189.9 | 2024 |
| Liberia | Both | 156.6 | 110 | 203.2 | 2025 |
| Liberia | Both | 166.3 | 114.4 | 218.2 | 2026 |
| Liberia | Both | 177 | 118.7 | 235.3 | 2027 |
| Liberia | Both | 188.6 | 122.6 | 254.7 | 2028 |
| Liberia | Both | 201.5 | 126.1 | 276.9 | 2029 |
| Liberia | Both | 215.7 | 128.8 | 302.6 | 2030 |
| Libya | Both | 262.1 | 216.9 | 307.2 | 2018 |
| Libya | Both | 276 | 228.9 | 323 | 2019 |
| Libya | Both | 290.6 | 241.2 | 340 | 2020 |
| Libya | Both | 306.1 | 253.9 | 358.4 | 2021 |
| Libya | Both | 322.4 | 266.7 | 378.2 | 2022 |
| Libya | Both | 339.8 | 279.9 | 399.7 | 2023 |
| Libya | Both | 358.4 | 293.5 | 423.4 | 2024 |
| Libya | Both | 378.6 | 307.6 | 449.6 | 2025 |
| Libya | Both | 400.2 | 321.9 | 478.4 | 2026 |
| Libya | Both | 423.3 | 336.6 | 510.1 | 2027 |
| Libya | Both | 448.3 | 351.5 | 545 | 2028 |
| Libya | Both | 475.1 | 366.7 | 583.4 | 2029 |
| Libya | Both | 503.8 | 381.8 | 625.8 | 2030 |
| Lithuania | Both | 569.7 | 510.7 | 628.7 | 2018 |
| Lithuania | Both | 565 | 502.4 | 627.7 | 2019 |
| Lithuania | Both | 560.7 | 492.1 | 629.3 | 2020 |
| Lithuania | Both | 557 | 480.3 | 633.6 | 2021 |
| Lithuania | Both | 553.5 | 466.6 | 640.3 | 2022 |
| Lithuania | Both | 550.5 | 451.4 | 649.7 | 2023 |
| Lithuania | Both | 548.5 | 434.8 | 662.1 | 2024 |
| Lithuania | Both | 547.7 | 417.1 | 678.4 | 2025 |
| Lithuania | Both | 548.4 | 397.7 | 699 | 2026 |
| Lithuania | Both | 550.5 | 376.2 | 724.9 | 2027 |
| Lithuania | Both | 554.4 | 351.5 | 757.3 | 2028 |
| Lithuania | Both | 560 | 322.2 | 797.9 | 2029 |
| Lithuania | Both | 567.7 | 286.2 | 849.4 | 2030 |
| Luxembourg | Both | 38.4 | 24.5 | 270 | 2018 |
| Luxembourg | Both | 39.2 | 25 | 275.5 | 2019 |
| Luxembourg | Both | 40 | 25.5 | 280.8 | 2020 |
| Luxembourg | Both | 40.9 | 26.1 | 286.9 | 2021 |
| Luxembourg | Both | 41.8 | 26.6 | 292.1 | 2022 |
| Luxembourg | Both | 42.7 | 27.1 | 296.8 | 2023 |
| Luxembourg | Both | 43.7 | 27.6 | 301.5 | 2024 |
| Luxembourg | Both | 44.7 | 28 | 306.3 | 2025 |
| Luxembourg | Both | 45.7 | 28.4 | 310.4 | 2026 |
| Luxembourg | Both | 46.8 | 28.7 | 316 | 2027 |
| Luxembourg | Both | 47.9 | 28.9 | 324.1 | 2028 |
| Luxembourg | Both | 49.1 | 29 | 332.9 | 2029 |
| Luxembourg | Both | 50.4 | 29 | 341.8 | 2030 |
| Macedonia | Both | 142.8 | 111.7 | 174 | 2018 |
| Macedonia | Both | 146.1 | 113.7 | 178.5 | 2019 |
| Macedonia | Both | 149.6 | 115.4 | 183.7 | 2020 |
| Macedonia | Both | 153.2 | 116.7 | 189.7 | 2021 |
| Macedonia | Both | 157.2 | 117.6 | 196.8 | 2022 |
| Macedonia | Both | 161.7 | 118.1 | 205.2 | 2023 |
| Macedonia | Both | 166.6 | 118.1 | 215.1 | 2024 |
| Macedonia | Both | 172.2 | 117.5 | 227 | 2025 |
| Macedonia | Both | 178.3 | 115.8 | 240.8 | 2026 |
| Macedonia | Both | 185.2 | 112.9 | 257.5 | 2027 |
| Macedonia | Both | 193.1 | 108.3 | 277.9 | 2028 |
| Macedonia | Both | 202.2 | 101.2 | 303.2 | 2029 |
| Macedonia | Both | 212.7 | 90.4 | 335.1 | 2030 |
| Madagascar | Both | 365.2 | 315.5 | 414.9 | 2018 |
| Madagascar | Both | 378.7 | 326.5 | 430.8 | 2019 |
| Madagascar | Both | 392.7 | 337.6 | 447.9 | 2020 |
| Madagascar | Both | 407.3 | 348.5 | 466.1 | 2021 |
| Madagascar | Both | 422.2 | 359 | 485.4 | 2022 |
| Madagascar | Both | 437.4 | 369.1 | 505.8 | 2023 |
| Madagascar | Both | 453.3 | 378.9 | 527.7 | 2024 |
| Madagascar | Both | 470 | 388.6 | 551.3 | 2025 |
| Madagascar | Both | 486.9 | 397.7 | 576.2 | 2026 |
| Madagascar | Both | 504.7 | 406.4 | 603 | 2027 |
| Madagascar | Both | 523.3 | 414.7 | 631.9 | 2028 |
| Madagascar | Both | 542.7 | 422.6 | 662.9 | 2029 |
| Madagascar | Both | 563.1 | 429.9 | 696.4 | 2030 |
| Malawi | Both | 401.7 | 350.8 | 452.7 | 2018 |
| Malawi | Both | 412.5 | 358.4 | 466.6 | 2019 |
| Malawi | Both | 423.2 | 365.1 | 481.3 | 2020 |
| Malawi | Both | 435.2 | 372.1 | 498.3 | 2021 |
| Malawi | Both | 446.4 | 377.3 | 515.5 | 2022 |
| Malawi | Both | 457.4 | 381.4 | 533.4 | 2023 |
| Malawi | Both | 469 | 385.1 | 553 | 2024 |
| Malawi | Both | 482 | 388.8 | 575.3 | 2025 |
| Malawi | Both | 496 | 392 | 599.9 | 2026 |
| Malawi | Both | 511.2 | 395 | 627.5 | 2027 |
| Malawi | Both | 528 | 397.5 | 658.4 | 2028 |
| Malawi | Both | 546.2 | 399.4 | 693 | 2029 |
| Malawi | Both | 566 | 400.4 | 731.6 | 2030 |
| Malaysia | Both | 928.3 | 846.8 | 1009.8 | 2018 |
| Malaysia | Both | 961.2 | 876.2 | 1046.2 | 2019 |
| Malaysia | Both | 995.3 | 905.6 | 1084.9 | 2020 |
| Malaysia | Both | 1029.5 | 933.9 | 1125.1 | 2021 |
| Malaysia | Both | 1064.9 | 961.8 | 1168 | 2022 |
| Malaysia | Both | 1101.7 | 989.4 | 1214 | 2023 |
| Malaysia | Both | 1139.7 | 1016.4 | 1263 | 2024 |
| Malaysia | Both | 1179.1 | 1042.9 | 1315.4 | 2025 |
| Malaysia | Both | 1218.4 | 1067.4 | 1369.4 | 2026 |
| Malaysia | Both | 1258.9 | 1091.1 | 1426.8 | 2027 |
| Malaysia | Both | 1301 | 1114.1 | 1487.9 | 2028 |
| Malaysia | Both | 1344.8 | 1136.5 | 1553.1 | 2029 |
| Malaysia | Both | 1390.4 | 1158.2 | 1622.6 | 2030 |
| Mali | Both | 389.2 | 338.9 | 439.6 | 2018 |
| Mali | Both | 411.2 | 357.4 | 465 | 2019 |
| Mali | Both | 434.7 | 376.5 | 492.8 | 2020 |
| Mali | Both | 460.1 | 396.3 | 523.8 | 2021 |
| Mali | Both | 486.9 | 416.4 | 557.3 | 2022 |
| Mali | Both | 515.3 | 436.8 | 593.9 | 2023 |
| Mali | Both | 545.9 | 457.7 | 634.1 | 2024 |
| Mali | Both | 578.9 | 479.2 | 678.7 | 2025 |
| Mali | Both | 615.1 | 501.6 | 728.7 | 2026 |
| Mali | Both | 653.6 | 523.9 | 783.3 | 2027 |
| Mali | Both | 694.6 | 546 | 843.3 | 2028 |
| Mali | Both | 738.7 | 568.1 | 909.4 | 2029 |
| Mali | Both | 786.5 | 590 | 983 | 2030 |
| Malta | Both | 58.2 | 41.4 | 75.4 | 2018 |
| Malta | Both | 60.2 | 42.8 | 77.8 | 2019 |
| Malta | Both | 62.2 | 44.3 | 80.4 | 2020 |
| Malta | Both | 64.2 | 45.7 | 83.1 | 2021 |
| Malta | Both | 66.3 | 47.1 | 85.9 | 2022 |
| Malta | Both | 68.5 | 48.5 | 89 | 2023 |
| Malta | Both | 70.9 | 49.8 | 92.3 | 2024 |
| Malta | Both | 73.3 | 51.1 | 95.9 | 2025 |
| Malta | Both | 75.7 | 52.2 | 99.6 | 2026 |
| Malta | Both | 78.3 | 53.3 | 103.7 | 2027 |
| Malta | Both | 81 | 54.2 | 108.2 | 2028 |
| Malta | Both | 83.9 | 55.1 | 113.1 | 2029 |
| Malta | Both | 87 | 55.8 | 118.6 | 2030 |
| Mauritania | Both | 107 | 78.6 | 135.4 | 2018 |
| Mauritania | Both | 110.9 | 81.6 | 140.1 | 2019 |
| Mauritania | Both | 114.9 | 84.6 | 145.2 | 2020 |
| Mauritania | Both | 119.1 | 87.6 | 150.6 | 2021 |
| Mauritania | Both | 123.5 | 90.6 | 156.4 | 2022 |
| Mauritania | Both | 128.1 | 93.7 | 162.6 | 2023 |
| Mauritania | Both | 133.1 | 96.7 | 169.4 | 2024 |
| Mauritania | Both | 138.3 | 99.8 | 176.7 | 2025 |
| Mauritania | Both | 143.7 | 102.8 | 184.6 | 2026 |
| Mauritania | Both | 149.5 | 105.7 | 193.2 | 2027 |
| Mauritania | Both | 155.6 | 108.6 | 202.6 | 2028 |
| Mauritania | Both | 162.1 | 111.4 | 212.8 | 2029 |
| Mauritania | Both | 169.1 | 114 | 224.2 | 2030 |
| Mauritius | Both | 57.4 | 38.1 | 77 | 2018 |
| Mauritius | Both | 58.5 | 38.9 | 78.4 | 2019 |
| Mauritius | Both | 59.8 | 39.8 | 80.1 | 2020 |
| Mauritius | Both | 61 | 40.6 | 81.7 | 2021 |
| Mauritius | Both | 62.3 | 41.3 | 83.6 | 2022 |
| Mauritius | Both | 63.7 | 42.1 | 85.6 | 2023 |
| Mauritius | Both | 65.1 | 42.8 | 87.8 | 2024 |
| Mauritius | Both | 66.6 | 43.4 | 90.1 | 2025 |
| Mauritius | Both | 68 | 43.9 | 92.5 | 2026 |
| Mauritius | Both | 69.5 | 44.2 | 95.2 | 2027 |
| Mauritius | Both | 71 | 44.4 | 98.1 | 2028 |
| Mauritius | Both | 72.6 | 44.4 | 101.2 | 2029 |
| Mauritius | Both | 74.2 | 44.2 | 104.6 | 2030 |
| Mexico | Both | 8590.1 | 8325.3 | 8854.8 | 2018 |
| Mexico | Both | 8901.9 | 8613.1 | 9190.7 | 2019 |
| Mexico | Both | 9220.5 | 8896.4 | 9544.5 | 2020 |
| Mexico | Both | 9538 | 9166.3 | 9909.8 | 2021 |
| Mexico | Both | 9865 | 9432 | 10298 | 2022 |
| Mexico | Both | 10203.7 | 9695.4 | 10712 | 2023 |
| Mexico | Both | 10554.1 | 9957 | 11151.2 | 2024 |
| Mexico | Both | 10918.2 | 10219.1 | 11617.3 | 2025 |
| Mexico | Both | 11276.5 | 10462.8 | 12090.2 | 2026 |
| Mexico | Both | 11649.7 | 10707 | 12592.4 | 2027 |
| Mexico | Both | 12037.2 | 10950.3 | 13124.1 | 2028 |
| Mexico | Both | 12434.3 | 11188.3 | 13680.3 | 2029 |
| Mexico | Both | 12839.6 | 11419.2 | 14260 | 2030 |
| Moldova | Both | 371.5 | 322.7 | 420.4 | 2018 |
| Moldova | Both | 377.4 | 327.1 | 427.7 | 2019 |
| Moldova | Both | 382.8 | 330.4 | 435.3 | 2020 |
| Moldova | Both | 387.4 | 332.2 | 442.6 | 2021 |
| Moldova | Both | 391.6 | 333 | 450.2 | 2022 |
| Moldova | Both | 395.7 | 333 | 458.5 | 2023 |
| Moldova | Both | 400 | 332.5 | 467.6 | 2024 |
| Moldova | Both | 404.7 | 331.4 | 477.9 | 2025 |
| Moldova | Both | 408.6 | 328.9 | 488.3 | 2026 |
| Moldova | Both | 413 | 326 | 500 | 2027 |
| Moldova | Both | 417.8 | 322.5 | 513 | 2028 |
| Moldova | Both | 422.4 | 317.9 | 526.9 | 2029 |
| Moldova | Both | 426.7 | 312 | 541.5 | 2030 |
| Mongolia | Both | 131.3 | 100.9 | 161.8 | 2018 |
| Mongolia | Both | 136.2 | 104.9 | 167.6 | 2019 |
| Mongolia | Both | 141.4 | 108.9 | 173.9 | 2020 |
| Mongolia | Both | 147.1 | 113.1 | 181.1 | 2021 |
| Mongolia | Both | 152.9 | 117.1 | 188.7 | 2022 |
| Mongolia | Both | 158.9 | 120.9 | 196.9 | 2023 |
| Mongolia | Both | 165.3 | 124.7 | 206 | 2024 |
| Mongolia | Both | 172.3 | 128.5 | 216.3 | 2025 |
| Mongolia | Both | 179.4 | 131.7 | 227.2 | 2026 |
| Mongolia | Both | 187.1 | 134.9 | 239.3 | 2027 |
| Mongolia | Both | 195.3 | 137.7 | 252.9 | 2028 |
| Mongolia | Both | 203.9 | 140.1 | 267.8 | 2029 |
| Mongolia | Both | 213 | 141.9 | 284.2 | 2030 |
| Montenegro | Both | 52.7 | 37.2 | 68.6 | 2018 |
| Montenegro | Both | 53.2 | 37.6 | 69.3 | 2019 |
| Montenegro | Both | 53.7 | 38 | 70 | 2020 |
| Montenegro | Both | 54.2 | 38.2 | 70.7 | 2021 |
| Montenegro | Both | 54.7 | 38.4 | 71.4 | 2022 |
| Montenegro | Both | 55.1 | 38.5 | 72.2 | 2023 |
| Montenegro | Both | 55.5 | 38.6 | 73 | 2024 |
| Montenegro | Both | 55.9 | 38.4 | 73.8 | 2025 |
| Montenegro | Both | 56.1 | 38.1 | 74.5 | 2026 |
| Montenegro | Both | 56.3 | 37.7 | 75.3 | 2027 |
| Montenegro | Both | 56.4 | 37.2 | 76.1 | 2028 |
| Montenegro | Both | 56.5 | 36.5 | 77 | 2029 |
| Montenegro | Both | 56.5 | 35.7 | 77.9 | 2030 |
| Morocco | Both | 655.5 | 584.4 | 726.7 | 2018 |
| Morocco | Both | 680 | 606.1 | 753.9 | 2019 |
| Morocco | Both | 705.2 | 627.9 | 782.5 | 2020 |
| Morocco | Both | 731.4 | 649.7 | 813.1 | 2021 |
| Morocco | Both | 758 | 670.9 | 845.1 | 2022 |
| Morocco | Both | 785.5 | 691.8 | 879.1 | 2023 |
| Morocco | Both | 814.1 | 712.6 | 915.6 | 2024 |
| Morocco | Both | 844.2 | 733.4 | 955.1 | 2025 |
| Morocco | Both | 874.7 | 753.1 | 996.4 | 2026 |
| Morocco | Both | 906.7 | 772.6 | 1040.8 | 2027 |
| Morocco | Both | 940.1 | 791.9 | 1088.4 | 2028 |
| Morocco | Both | 974.9 | 810.5 | 1139.3 | 2029 |
| Morocco | Both | 1011 | 828.5 | 1193.4 | 2030 |
| Mozambique | Both | 472.4 | 413.6 | 531.3 | 2018 |
| Mozambique | Both | 491.9 | 429.4 | 554.4 | 2019 |
| Mozambique | Both | 512.7 | 445.4 | 580 | 2020 |
| Mozambique | Both | 534.7 | 461.3 | 608.1 | 2021 |
| Mozambique | Both | 558.1 | 477.1 | 639 | 2022 |
| Mozambique | Both | 583.1 | 492.8 | 673.3 | 2023 |
| Mozambique | Both | 609.8 | 508.3 | 711.3 | 2024 |
| Mozambique | Both | 638.6 | 523.7 | 753.5 | 2025 |
| Mozambique | Both | 669.4 | 538.6 | 800.2 | 2026 |
| Mozambique | Both | 702.2 | 552.8 | 851.6 | 2027 |
| Mozambique | Both | 737.4 | 566.2 | 908.5 | 2028 |
| Mozambique | Both | 775.4 | 578.7 | 972 | 2029 |
| Mozambique | Both | 816.7 | 590.3 | 1043.2 | 2030 |
| Myanmar | Both | 2162.2 | 2031.1 | 2293.3 | 2018 |
| Myanmar | Both | 2202.6 | 2065.2 | 2339.9 | 2019 |
| Myanmar | Both | 2244.5 | 2098.2 | 2390.7 | 2020 |
| Myanmar | Both | 2285.7 | 2127.3 | 2444 | 2021 |
| Myanmar | Both | 2327.7 | 2154 | 2501.3 | 2022 |
| Myanmar | Both | 2370.5 | 2178.1 | 2562.8 | 2023 |
| Myanmar | Both | 2414.3 | 2199.9 | 2628.7 | 2024 |
| Myanmar | Both | 2459.5 | 2219.7 | 2699.3 | 2025 |
| Myanmar | Both | 2504.2 | 2235.7 | 2772.7 | 2026 |
| Myanmar | Both | 2549.8 | 2249.2 | 2850.4 | 2027 |
| Myanmar | Both | 2596.1 | 2260 | 2932.2 | 2028 |
| Myanmar | Both | 2643.1 | 2268 | 3018.1 | 2029 |
| Myanmar | Both | 2691 | 2273.4 | 3108.7 | 2030 |
| Namibia | Both | 40.9 | 23.7 | 58.3 | 2018 |
| Namibia | Both | 41.9 | 24.3 | 59.7 | 2019 |
| Namibia | Both | 42.9 | 24.9 | 61.1 | 2020 |
| Namibia | Both | 44 | 25.5 | 62.7 | 2021 |
| Namibia | Both | 45.1 | 26 | 64.4 | 2022 |
| Namibia | Both | 46.4 | 26.6 | 66.3 | 2023 |
| Namibia | Both | 47.7 | 27.1 | 68.4 | 2024 |
| Namibia | Both | 49.1 | 27.6 | 70.8 | 2025 |
| Namibia | Both | 50.7 | 28.1 | 73.5 | 2026 |
| Namibia | Both | 52.4 | 28.4 | 76.6 | 2027 |
| Namibia | Both | 54.2 | 28.6 | 80 | 2028 |
| Namibia | Both | 56.3 | 28.7 | 84 | 2029 |
| Namibia | Both | 58.5 | 28.6 | 88.5 | 2030 |
| Nepal | Both | 448.6 | 390.4 | 506.8 | 2018 |
| Nepal | Both | 472.3 | 411.1 | 533.5 | 2019 |
| Nepal | Both | 497.7 | 432.5 | 563 | 2020 |
| Nepal | Both | 524.1 | 453.7 | 594.5 | 2021 |
| Nepal | Both | 552.6 | 475.5 | 629.6 | 2022 |
| Nepal | Both | 583.2 | 497.9 | 668.5 | 2023 |
| Nepal | Both | 615.7 | 520.3 | 711.2 | 2024 |
| Nepal | Both | 650.4 | 542.8 | 758 | 2025 |
| Nepal | Both | 687.3 | 565.1 | 809.4 | 2026 |
| Nepal | Both | 726.6 | 587.3 | 866 | 2027 |
| Nepal | Both | 768.7 | 609.2 | 928.2 | 2028 |
| Nepal | Both | 813.9 | 630.8 | 997 | 2029 |
| Nepal | Both | 862.8 | 652.2 | 1073.4 | 2030 |
| Netherlands | Both | 2819.5 | 2689.3 | 2949.7 | 2018 |
| Netherlands | Both | 2852.7 | 2714.3 | 2991.2 | 2019 |
| Netherlands | Both | 2887 | 2736.1 | 3037.9 | 2020 |
| Netherlands | Both | 2917.9 | 2750.4 | 3085.5 | 2021 |
| Netherlands | Both | 2950.1 | 2761.5 | 3138.7 | 2022 |
| Netherlands | Both | 2982.2 | 2768.5 | 3196 | 2023 |
| Netherlands | Both | 3013.2 | 2770.5 | 3255.9 | 2024 |
| Netherlands | Both | 3043.1 | 2767.9 | 3318.3 | 2025 |
| Netherlands | Both | 3068.5 | 2757.7 | 3379.3 | 2026 |
| Netherlands | Both | 3093.3 | 2743.6 | 3443 | 2027 |
| Netherlands | Both | 3117.9 | 2725.9 | 3509.8 | 2028 |
| Netherlands | Both | 3142.9 | 2705.3 | 3580.6 | 2029 |
| Netherlands | Both | 3169.7 | 2682.7 | 3656.7 | 2030 |
| New Zealand | Both | 635.5 | 572.3 | 698.7 | 2018 |
| New Zealand | Both | 646 | 580.7 | 711.2 | 2019 |
| New Zealand | Both | 657 | 588.8 | 725.2 | 2020 |
| New Zealand | Both | 667.6 | 595.6 | 739.5 | 2021 |
| New Zealand | Both | 678.6 | 601.8 | 755.3 | 2022 |
| New Zealand | Both | 690 | 607.4 | 772.6 | 2023 |
| New Zealand | Both | 701.7 | 612.2 | 791.2 | 2024 |
| New Zealand | Both | 713.7 | 616.2 | 811.2 | 2025 |
| New Zealand | Both | 724.8 | 618.3 | 831.3 | 2026 |
| New Zealand | Both | 736.2 | 619.6 | 852.9 | 2027 |
| New Zealand | Both | 748.2 | 620.2 | 876.2 | 2028 |
| New Zealand | Both | 761 | 620.4 | 901.6 | 2029 |
| New Zealand | Both | 774.7 | 620 | 929.3 | 2030 |
| Nicaragua | Both | 172.8 | 136.2 | 209.5 | 2018 |
| Nicaragua | Both | 177.8 | 140.3 | 215.3 | 2019 |
| Nicaragua | Both | 183 | 144.5 | 221.6 | 2020 |
| Nicaragua | Both | 188.3 | 148.6 | 228.1 | 2021 |
| Nicaragua | Both | 193.9 | 152.6 | 235.2 | 2022 |
| Nicaragua | Both | 199.8 | 156.7 | 243 | 2023 |
| Nicaragua | Both | 206.1 | 160.8 | 251.5 | 2024 |
| Nicaragua | Both | 212.8 | 164.9 | 260.8 | 2025 |
| Nicaragua | Both | 219.6 | 168.5 | 270.7 | 2026 |
| Nicaragua | Both | 226.8 | 172.1 | 281.4 | 2027 |
| Nicaragua | Both | 234.3 | 175.4 | 293.2 | 2028 |
| Nicaragua | Both | 242.2 | 178.4 | 306.1 | 2029 |
| Nicaragua | Both | 250.6 | 181.1 | 320.1 | 2030 |
| Niger | Both | 389.5 | 340.3 | 438.8 | 2018 |
| Niger | Both | 407.3 | 354.9 | 459.6 | 2019 |
| Niger | Both | 426.1 | 369.9 | 482.4 | 2020 |
| Niger | Both | 446.1 | 385 | 507.3 | 2021 |
| Niger | Both | 467 | 399.9 | 534 | 2022 |
| Niger | Both | 488.9 | 414.8 | 562.9 | 2023 |
| Niger | Both | 511.9 | 429.6 | 594.1 | 2024 |
| Niger | Both | 536.2 | 444.4 | 628.1 | 2025 |
| Niger | Both | 561.9 | 458.9 | 665 | 2026 |
| Niger | Both | 588.7 | 472.7 | 704.6 | 2027 |
| Niger | Both | 616.8 | 486.1 | 747.5 | 2028 |
| Niger | Both | 646.7 | 499 | 794.5 | 2029 |
| Niger | Both | 679 | 511.7 | 846.4 | 2030 |
| Nigeria | Both | 4328.2 | 4142.7 | 4513.7 | 2018 |
| Nigeria | Both | 4531.2 | 4320.2 | 4742.2 | 2019 |
| Nigeria | Both | 4745.3 | 4498.8 | 4991.9 | 2020 |
| Nigeria | Both | 4978.2 | 4684.9 | 5271.6 | 2021 |
| Nigeria | Both | 5222.6 | 4871.1 | 5574.2 | 2022 |
| Nigeria | Both | 5480.3 | 5058.1 | 5902.4 | 2023 |
| Nigeria | Both | 5752.1 | 5246.1 | 6258.1 | 2024 |
| Nigeria | Both | 6039.6 | 5435.5 | 6643.6 | 2025 |
| Nigeria | Both | 6355 | 5636.1 | 7074 | 2026 |
| Nigeria | Both | 6685.2 | 5834.7 | 7535.7 | 2027 |
| Nigeria | Both | 7033.2 | 6032.4 | 8033.9 | 2028 |
| Nigeria | Both | 7402.8 | 6230.8 | 8574.7 | 2029 |
| Nigeria | Both | 7798.1 | 6431.2 | 9164.9 | 2030 |
| North Korea | Both | 1153.3 | 1057.9 | 1248.6 | 2018 |
| North Korea | Both | 1165 | 1066.2 | 1263.9 | 2019 |
| North Korea | Both | 1179 | 1075.6 | 1282.5 | 2020 |
| North Korea | Both | 1195 | 1085.7 | 1304.3 | 2021 |
| North Korea | Both | 1211.9 | 1095.1 | 1328.6 | 2022 |
| North Korea | Both | 1226.2 | 1100.4 | 1352 | 2023 |
| North Korea | Both | 1239.4 | 1103 | 1375.7 | 2024 |
| North Korea | Both | 1255.6 | 1107.1 | 1404.1 | 2025 |
| North Korea | Both | 1274.4 | 1111.9 | 1436.9 | 2026 |
| North Korea | Both | 1294.8 | 1116.3 | 1473.4 | 2027 |
| North Korea | Both | 1313.9 | 1117.2 | 1510.5 | 2028 |
| North Korea | Both | 1332.4 | 1115.7 | 1549 | 2029 |
| North Korea | Both | 1354.1 | 1114.9 | 1593.3 | 2030 |
| Norway | Both | 980.6 | 904.4 | 1056.7 | 2018 |
| Norway | Both | 982.7 | 903.2 | 1062.2 | 2019 |
| Norway | Both | 985.5 | 900.9 | 1070.2 | 2020 |
| Norway | Both | 987.2 | 895.7 | 1078.8 | 2021 |
| Norway | Both | 989.5 | 889.3 | 1089.8 | 2022 |
| Norway | Both | 992.4 | 881.6 | 1103.1 | 2023 |
| Norway | Both | 995.2 | 872.4 | 1117.9 | 2024 |
| Norway | Both | 997.8 | 861.4 | 1134.2 | 2025 |
| Norway | Both | 998.9 | 847.6 | 1150.2 | 2026 |
| Norway | Both | 999.9 | 832.4 | 1167.4 | 2027 |
| Norway | Both | 1001.1 | 815.8 | 1186.4 | 2028 |
| Norway | Both | 1002.9 | 798.3 | 1207.5 | 2029 |
| Norway | Both | 1005.8 | 780.1 | 1231.5 | 2030 |
| Oman | Both | 79.4 | 55 | 103.9 | 2018 |
| Oman | Both | 84.7 | 59.2 | 110.3 | 2019 |
| Oman | Both | 90 | 63.4 | 116.8 | 2020 |
| Oman | Both | 96.6 | 68.5 | 124.8 | 2021 |
| Oman | Both | 103 | 73.3 | 132.8 | 2022 |
| Oman | Both | 109.6 | 78.1 | 141.2 | 2023 |
| Oman | Both | 116.5 | 82.9 | 150.1 | 2024 |
| Oman | Both | 123.8 | 87.8 | 159.9 | 2025 |
| Oman | Both | 132.3 | 93.3 | 171.3 | 2026 |
| Oman | Both | 141.5 | 99 | 184 | 2027 |
| Oman | Both | 151.3 | 104.7 | 198 | 2028 |
| Oman | Both | 161.9 | 110.3 | 213.4 | 2029 |
| Oman | Both | 173.1 | 115.8 | 230.3 | 2030 |
| Pakistan | Both | 3457.2 | 3281.1 | 3633.4 | 2018 |
| Pakistan | Both | 3559.7 | 3371.2 | 3748.2 | 2019 |
| Pakistan | Both | 3666.7 | 3461.1 | 3872.3 | 2020 |
| Pakistan | Both | 3774.4 | 3546.5 | 4002.4 | 2021 |
| Pakistan | Both | 3887.5 | 3631.3 | 4143.7 | 2022 |
| Pakistan | Both | 4006.8 | 3716.1 | 4297.6 | 2023 |
| Pakistan | Both | 4133 | 3801.2 | 4464.8 | 2024 |
| Pakistan | Both | 4266.8 | 3887.4 | 4646.3 | 2025 |
| Pakistan | Both | 4402.7 | 3969 | 4836.3 | 2026 |
| Pakistan | Both | 4547.1 | 4051.9 | 5042.2 | 2027 |
| Pakistan | Both | 4700.9 | 4136.5 | 5265.3 | 2028 |
| Pakistan | Both | 4864.2 | 4222.3 | 5506.1 | 2029 |
| Pakistan | Both | 5037.6 | 4309.3 | 5765.9 | 2030 |
| Palestine | Both | 149.7 | 114.7 | 184.6 | 2018 |
| Palestine | Both | 156.2 | 120.1 | 192.4 | 2019 |
| Palestine | Both | 163.1 | 125.5 | 200.7 | 2020 |
| Palestine | Both | 170.3 | 131.1 | 209.6 | 2021 |
| Palestine | Both | 178 | 136.8 | 219.2 | 2022 |
| Palestine | Both | 186 | 142.5 | 229.5 | 2023 |
| Palestine | Both | 194.5 | 148.3 | 240.7 | 2024 |
| Palestine | Both | 203.5 | 154.1 | 252.9 | 2025 |
| Palestine | Both | 212.9 | 159.8 | 266 | 2026 |
| Palestine | Both | 222.8 | 165.3 | 280.3 | 2027 |
| Palestine | Both | 233.3 | 170.8 | 295.9 | 2028 |
| Palestine | Both | 244.5 | 176 | 313 | 2029 |
| Palestine | Both | 256.4 | 181.1 | 331.7 | 2030 |
| Panama | Both | 196.7 | 157.9 | 235.5 | 2018 |
| Panama | Both | 208.2 | 167.7 | 248.6 | 2019 |
| Panama | Both | 220.4 | 177.9 | 262.8 | 2020 |
| Panama | Both | 233.1 | 188.3 | 277.8 | 2021 |
| Panama | Both | 246.6 | 199 | 294.1 | 2022 |
| Panama | Both | 261 | 210.1 | 311.8 | 2023 |
| Panama | Both | 276.3 | 221.5 | 331.1 | 2024 |
| Panama | Both | 292.8 | 233.3 | 352.3 | 2025 |
| Panama | Both | 309.9 | 244.9 | 374.9 | 2026 |
| Panama | Both | 328.2 | 256.7 | 399.7 | 2027 |
| Panama | Both | 347.7 | 268.7 | 426.8 | 2028 |
| Panama | Both | 368.7 | 280.8 | 456.5 | 2029 |
| Panama | Both | 391.1 | 293 | 489.2 | 2030 |
| Papua New Guinea | Both | 190.5 | 153.3 | 227.8 | 2018 |
| Papua New Guinea | Both | 197.4 | 158.8 | 235.9 | 2019 |
| Papua New Guinea | Both | 204.5 | 164.5 | 244.6 | 2020 |
| Papua New Guinea | Both | 211.8 | 170 | 253.6 | 2021 |
| Papua New Guinea | Both | 219.4 | 175.5 | 263.4 | 2022 |
| Papua New Guinea | Both | 227.5 | 181 | 273.9 | 2023 |
| Papua New Guinea | Both | 236 | 186.6 | 285.3 | 2024 |
| Papua New Guinea | Both | 245 | 192.2 | 297.8 | 2025 |
| Papua New Guinea | Both | 254.2 | 197.4 | 310.9 | 2026 |
| Papua New Guinea | Both | 263.9 | 202.6 | 325.3 | 2027 |
| Papua New Guinea | Both | 274.3 | 207.6 | 340.9 | 2028 |
| Papua New Guinea | Both | 285.3 | 212.6 | 358 | 2029 |
| Papua New Guinea | Both | 297.1 | 217.4 | 376.8 | 2030 |
| Paraguay | Both | 263.3 | 219 | 307.5 | 2018 |
| Paraguay | Both | 271.2 | 225.8 | 316.6 | 2019 |
| Paraguay | Both | 279.5 | 232.7 | 326.3 | 2020 |
| Paraguay | Both | 287.8 | 239.3 | 336.3 | 2021 |
| Paraguay | Both | 296.4 | 245.8 | 347 | 2022 |
| Paraguay | Both | 305.5 | 252.4 | 358.6 | 2023 |
| Paraguay | Both | 314.9 | 258.7 | 371 | 2024 |
| Paraguay | Both | 324.6 | 264.9 | 384.3 | 2025 |
| Paraguay | Both | 334.4 | 270.6 | 398.3 | 2026 |
| Paraguay | Both | 344.6 | 275.9 | 413.3 | 2027 |
| Paraguay | Both | 355.2 | 280.9 | 429.5 | 2028 |
| Paraguay | Both | 366.2 | 285.5 | 446.9 | 2029 |
| Paraguay | Both | 377.7 | 289.7 | 465.7 | 2030 |
| Peru | Both | 1580.4 | 1465 | 1695.8 | 2018 |
| Peru | Both | 1624 | 1493.5 | 1754.5 | 2019 |
| Peru | Both | 1670.6 | 1515.7 | 1825.6 | 2020 |
| Peru | Both | 1719.7 | 1530.7 | 1908.7 | 2021 |
| Peru | Both | 1772.7 | 1540 | 2005.5 | 2022 |
| Peru | Both | 1830.9 | 1544.3 | 2117.5 | 2023 |
| Peru | Both | 1895.6 | 1544.1 | 2247 | 2024 |
| Peru | Both | 1968 | 1539 | 2397.1 | 2025 |
| Peru | Both | 2046.8 | 1525.8 | 2567.7 | 2026 |
| Peru | Both | 2135.7 | 1504.7 | 2766.6 | 2027 |
| Peru | Both | 2236.7 | 1473 | 3000.3 | 2028 |
| Peru | Both | 2351.7 | 1426.3 | 3277.1 | 2029 |
| Peru | Both | 2483.1 | 1358.1 | 3608.2 | 2030 |
| Philippines | Both | 3901.1 | 3695 | 4107.1 | 2018 |
| Philippines | Both | 3950.5 | 3680.2 | 4220.8 | 2019 |
| Philippines | Both | 4009.1 | 3639.7 | 4378.5 | 2020 |
| Philippines | Both | 4078.2 | 3579 | 4577.4 | 2021 |
| Philippines | Both | 4163 | 3503.7 | 4822.3 | 2022 |
| Philippines | Both | 4268 | 3414.9 | 5121.2 | 2023 |
| Philippines | Both | 4398.3 | 3310.3 | 5486.2 | 2024 |
| Philippines | Both | 4559.2 | 3183.4 | 5935 | 2025 |
| Philippines | Both | 4755.4 | 3021.1 | 6489.6 | 2026 |
| Philippines | Both | 4997.6 | 2805.5 | 7189.6 | 2027 |
| Philippines | Both | 5297.6 | 2504 | 8091.2 | 2028 |
| Philippines | Both | 5671 | 2061.8 | 9280.1 | 2029 |
| Philippines | Both | 6137.6 | 1384.4 | 10890.9 | 2030 |
| Poland | Both | 5315.5 | 5131 | 5500 | 2018 |
| Poland | Both | 5330.3 | 5130.3 | 5530.3 | 2019 |
| Poland | Both | 5351.8 | 5128.6 | 5575.1 | 2020 |
| Poland | Both | 5371.8 | 5117.6 | 5626 | 2021 |
| Poland | Both | 5387.6 | 5095.4 | 5679.9 | 2022 |
| Poland | Both | 5396.3 | 5059.7 | 5732.8 | 2023 |
| Poland | Both | 5399.9 | 5014 | 5785.7 | 2024 |
| Poland | Both | 5400.4 | 4960.8 | 5840 | 2025 |
| Poland | Both | 5395.1 | 4897.9 | 5892.4 | 2026 |
| Poland | Both | 5386.6 | 4827.9 | 5945.4 | 2027 |
| Poland | Both | 5376.7 | 4752.5 | 6000.9 | 2028 |
| Poland | Both | 5371 | 4677.2 | 6064.8 | 2029 |
| Poland | Both | 5371.1 | 4603.4 | 6138.8 | 2030 |
| Portugal | Both | 1257 | 1166 | 1348.1 | 2018 |
| Portugal | Both | 1259.8 | 1161.3 | 1358.2 | 2019 |
| Portugal | Both | 1263.7 | 1153.3 | 1374 | 2020 |
| Portugal | Both | 1266.6 | 1140.1 | 1393 | 2021 |
| Portugal | Both | 1271.1 | 1124.2 | 1418 | 2022 |
| Portugal | Both | 1277.4 | 1105.9 | 1448.9 | 2023 |
| Portugal | Both | 1285.3 | 1085.1 | 1485.5 | 2024 |
| Portugal | Both | 1295.3 | 1062 | 1528.5 | 2025 |
| Portugal | Both | 1305.3 | 1034.5 | 1576.1 | 2026 |
| Portugal | Both | 1317.6 | 1003.9 | 1631.3 | 2027 |
| Portugal | Both | 1332.4 | 969.4 | 1695.4 | 2028 |
| Portugal | Both | 1350.2 | 930.1 | 1770.2 | 2029 |
| Portugal | Both | 1371.6 | 884.6 | 1858.5 | 2030 |
| Puerto Rico | Both | 249.2 | 208.5 | 289.8 | 2018 |
| Puerto Rico | Both | 253.9 | 212.2 | 295.5 | 2019 |
| Puerto Rico | Both | 258.7 | 215.6 | 301.8 | 2020 |
| Puerto Rico | Both | 263.4 | 218.5 | 308.3 | 2021 |
| Puerto Rico | Both | 268.3 | 221 | 315.6 | 2022 |
| Puerto Rico | Both | 273.4 | 223.3 | 323.5 | 2023 |
| Puerto Rico | Both | 278.7 | 225.1 | 332.3 | 2024 |
| Puerto Rico | Both | 284.2 | 226.4 | 341.9 | 2025 |
| Puerto Rico | Both | 289.7 | 227.1 | 352.3 | 2026 |
| Puerto Rico | Both | 295.5 | 227.3 | 363.7 | 2027 |
| Puerto Rico | Both | 301.5 | 226.9 | 376.2 | 2028 |
| Puerto Rico | Both | 307.9 | 225.9 | 390 | 2029 |
| Puerto Rico | Both | 314.7 | 224.2 | 405.3 | 2030 |
| Qatar | Both | 42.5 | 25.6 | 59.7 | 2018 |
| Qatar | Both | 44.4 | 26.9 | 62.1 | 2019 |
| Qatar | Both | 46.4 | 28.3 | 64.8 | 2020 |
| Qatar | Both | 48.9 | 30 | 68.1 | 2021 |
| Qatar | Both | 51.6 | 31.7 | 71.7 | 2022 |
| Qatar | Both | 54.3 | 33.3 | 75.6 | 2023 |
| Qatar | Both | 57.2 | 34.9 | 79.8 | 2024 |
| Qatar | Both | 60.2 | 36.5 | 84.3 | 2025 |
| Qatar | Both | 63.8 | 38.2 | 89.6 | 2026 |
| Qatar | Both | 67.5 | 39.8 | 95.5 | 2027 |
| Qatar | Both | 71.4 | 41.2 | 101.9 | 2028 |
| Qatar | Both | 75.6 | 42.3 | 109 | 2029 |
| Qatar | Both | 79.9 | 43.2 | 116.9 | 2030 |
| Romania | Both | 2329.1 | 2205.5 | 2452.8 | 2018 |
| Romania | Both | 2358.4 | 2226.3 | 2490.6 | 2019 |
| Romania | Both | 2391.8 | 2246.6 | 2537 | 2020 |
| Romania | Both | 2422.9 | 2260.2 | 2585.7 | 2021 |
| Romania | Both | 2456.7 | 2271.6 | 2641.8 | 2022 |
| Romania | Both | 2491.5 | 2279.5 | 2703.4 | 2023 |
| Romania | Both | 2525.5 | 2282.7 | 2768.4 | 2024 |
| Romania | Both | 2558.1 | 2280.5 | 2835.7 | 2025 |
| Romania | Both | 2585.7 | 2269.9 | 2901.5 | 2026 |
| Romania | Both | 2612 | 2254.4 | 2969.6 | 2027 |
| Romania | Both | 2637.9 | 2234.7 | 3041.1 | 2028 |
| Romania | Both | 2664.7 | 2211.8 | 3117.6 | 2029 |
| Romania | Both | 2693.1 | 2186 | 3200.1 | 2030 |
| Russian Federation | Both | 22830.4 | 22057.4 | 23603.4 | 2018 |
| Russian Federation | Both | 23027.3 | 21554.9 | 24499.8 | 2019 |
| Russian Federation | Both | 23137.4 | 20761.7 | 25513.2 | 2020 |
| Russian Federation | Both | 23260.8 | 19801.5 | 26720.1 | 2021 |
| Russian Federation | Both | 23733 | 18943.1 | 28522.9 | 2022 |
| Russian Federation | Both | 24691 | 18189.3 | 31192.8 | 2023 |
| Russian Federation | Both | 25736.8 | 17127.8 | 34345.8 | 2024 |
| Russian Federation | Both | 26801.8 | 15598.1 | 38005.5 | 2025 |
| Russian Federation | Both | 28048.2 | 13508 | 42588.4 | 2026 |
| Russian Federation | Both | 30080.7 | 10800.3 | 49370 | 2027 |
| Russian Federation | Both | 33349 | 6832.9 | 59923 | 2028 |
| Russian Federation | Both | 37548.4 | 113.3 | 75142.4 | 2029 |
| Russian Federation | Both | 42710.1 | 0 | 97378.5 | 2030 |
| Rwanda | Both | 224.2 | 183.2 | 265.2 | 2018 |
| Rwanda | Both | 233.3 | 187.3 | 279.3 | 2019 |
| Rwanda | Both | 243.7 | 190.4 | 297 | 2020 |
| Rwanda | Both | 255.8 | 192.2 | 319.3 | 2021 |
| Rwanda | Both | 270.1 | 192.5 | 347.7 | 2022 |
| Rwanda | Both | 287.4 | 190.8 | 383.9 | 2023 |
| Rwanda | Both | 308.5 | 186 | 431 | 2024 |
| Rwanda | Both | 334.8 | 176.2 | 493.4 | 2025 |
| Rwanda | Both | 367.2 | 157.3 | 577 | 2026 |
| Rwanda | Both | 408.1 | 122.4 | 693.8 | 2027 |
| Rwanda | Both | 460.5 | 57.8 | 863.2 | 2028 |
| Rwanda | Both | 528.7 | 0 | 1120.4 | 2029 |
| Rwanda | Both | 619.2 | 0 | 1531.8 | 2030 |
| Saint Lucia | Both | 8.1 | 2.6 | 14.2 | 2018 |
| Saint Lucia | Both | 8.3 | 2.7 | 14.4 | 2019 |
| Saint Lucia | Both | 8.4 | 2.7 | 14.6 | 2020 |
| Saint Lucia | Both | 8.5 | 2.8 | 14.9 | 2021 |
| Saint Lucia | Both | 8.7 | 2.8 | 15.1 | 2022 |
| Saint Lucia | Both | 8.8 | 2.8 | 15.4 | 2023 |
| Saint Lucia | Both | 9 | 2.8 | 15.8 | 2024 |
| Saint Lucia | Both | 9.2 | 2.8 | 16.2 | 2025 |
| Saint Lucia | Both | 9.4 | 2.7 | 16.6 | 2026 |
| Saint Lucia | Both | 9.6 | 2.7 | 17.1 | 2027 |
| Saint Lucia | Both | 9.8 | 2.5 | 17.8 | 2028 |
| Saint Lucia | Both | 10.1 | 2.3 | 18.6 | 2029 |
| Saint Lucia | Both | 10.4 | 1.9 | 19.6 | 2030 |
| Saudi Arabia | Both | 730.3 | 654.7 | 805.9 | 2018 |
| Saudi Arabia | Both | 767 | 687 | 847 | 2019 |
| Saudi Arabia | Both | 806.4 | 720.4 | 892.4 | 2020 |
| Saudi Arabia | Both | 845.7 | 752 | 939.5 | 2021 |
| Saudi Arabia | Both | 887.2 | 783.5 | 990.9 | 2022 |
| Saudi Arabia | Both | 930.8 | 814.6 | 1046.9 | 2023 |
| Saudi Arabia | Both | 976.5 | 845.2 | 1107.7 | 2024 |
| Saudi Arabia | Both | 1024.4 | 875.2 | 1173.7 | 2025 |
| Saudi Arabia | Both | 1075.4 | 904.8 | 1245.9 | 2026 |
| Saudi Arabia | Both | 1128.4 | 933.1 | 1323.8 | 2027 |
| Saudi Arabia | Both | 1183.9 | 959.9 | 1408 | 2028 |
| Saudi Arabia | Both | 1242.4 | 985.3 | 1499.5 | 2029 |
| Saudi Arabia | Both | 1304.3 | 1009.2 | 1599.4 | 2030 |
| Senegal | Both | 342.8 | 291.8 | 393.8 | 2018 |
| Senegal | Both | 354 | 300.7 | 407.2 | 2019 |
| Senegal | Both | 365.9 | 309.9 | 421.9 | 2020 |
| Senegal | Both | 378.5 | 319 | 437.9 | 2021 |
| Senegal | Both | 392 | 328.4 | 455.6 | 2022 |
| Senegal | Both | 406.6 | 338 | 475.2 | 2023 |
| Senegal | Both | 422.3 | 347.8 | 496.8 | 2024 |
| Senegal | Both | 439.2 | 357.6 | 520.7 | 2025 |
| Senegal | Both | 457 | 367.2 | 546.8 | 2026 |
| Senegal | Both | 476.1 | 376.6 | 575.5 | 2027 |
| Senegal | Both | 496.5 | 385.9 | 607.1 | 2028 |
| Senegal | Both | 518.5 | 394.9 | 642.1 | 2029 |
| Senegal | Both | 542.2 | 403.4 | 681 | 2030 |
| Serbia | Both | 1172.5 | 1087.4 | 1257.6 | 2018 |
| Serbia | Both | 1165.3 | 1076.7 | 1253.9 | 2019 |
| Serbia | Both | 1159.3 | 1065.4 | 1253.2 | 2020 |
| Serbia | Both | 1154 | 1053 | 1255 | 2021 |
| Serbia | Both | 1148.6 | 1038.6 | 1258.6 | 2022 |
| Serbia | Both | 1142 | 1021.3 | 1262.7 | 2023 |
| Serbia | Both | 1134 | 1001.3 | 1266.7 | 2024 |
| Serbia | Both | 1124.8 | 979 | 1270.6 | 2025 |
| Serbia | Both | 1114.9 | 954.9 | 1274.9 | 2026 |
| Serbia | Both | 1104.9 | 929.7 | 1280.2 | 2027 |
| Serbia | Both | 1095.7 | 903.9 | 1287.6 | 2028 |
| Serbia | Both | 1087.9 | 878.3 | 1297.6 | 2029 |
| Serbia | Both | 1081.3 | 852.6 | 1310.1 | 2030 |
| Sierra Leone | Both | 180.7 | 144.8 | 216.7 | 2018 |
| Sierra Leone | Both | 188.9 | 151.4 | 226.5 | 2019 |
| Sierra Leone | Both | 197.4 | 157.9 | 236.8 | 2020 |
| Sierra Leone | Both | 207.1 | 165.3 | 248.9 | 2021 |
| Sierra Leone | Both | 217.1 | 172.5 | 261.7 | 2022 |
| Sierra Leone | Both | 227.4 | 179.7 | 275.1 | 2023 |
| Sierra Leone | Both | 238.1 | 186.8 | 289.5 | 2024 |
| Sierra Leone | Both | 249.4 | 193.9 | 305 | 2025 |
| Sierra Leone | Both | 262.4 | 201.8 | 323 | 2026 |
| Sierra Leone | Both | 276 | 209.6 | 342.3 | 2027 |
| Sierra Leone | Both | 290.2 | 217.3 | 363.2 | 2028 |
| Sierra Leone | Both | 305.3 | 224.7 | 385.8 | 2029 |
| Sierra Leone | Both | 321.2 | 232 | 410.4 | 2030 |
| Singapore | Both | 242.7 | 202.2 | 283.1 | 2018 |
| Singapore | Both | 250.2 | 208.5 | 291.8 | 2019 |
| Singapore | Both | 257.6 | 214.5 | 300.7 | 2020 |
| Singapore | Both | 264.6 | 219.8 | 309.4 | 2021 |
| Singapore | Both | 271.5 | 224.6 | 318.3 | 2022 |
| Singapore | Both | 278.3 | 229 | 327.6 | 2023 |
| Singapore | Both | 285.2 | 233 | 337.4 | 2024 |
| Singapore | Both | 292.2 | 236.7 | 347.7 | 2025 |
| Singapore | Both | 298.9 | 239.6 | 358.2 | 2026 |
| Singapore | Both | 305.7 | 242.1 | 369.3 | 2027 |
| Singapore | Both | 312.8 | 244.3 | 381.2 | 2028 |
| Singapore | Both | 320 | 246.1 | 394 | 2029 |
| Singapore | Both | 327.5 | 247.4 | 407.6 | 2030 |
| Slovakia | Both | 1225 | 1136.7 | 1313.2 | 2018 |
| Slovakia | Both | 1198.7 | 1103.3 | 1294.1 | 2019 |
| Slovakia | Both | 1173.8 | 1067.3 | 1280.3 | 2020 |
| Slovakia | Both | 1149.6 | 1028.7 | 1270.5 | 2021 |
| Slovakia | Both | 1126.7 | 988.5 | 1264.9 | 2022 |
| Slovakia | Both | 1105.3 | 947.5 | 1263.2 | 2023 |
| Slovakia | Both | 1085.4 | 905.8 | 1265.1 | 2024 |
| Slovakia | Both | 1067 | 863.5 | 1270.4 | 2025 |
| Slovakia | Both | 1049.4 | 820.2 | 1278.5 | 2026 |
| Slovakia | Both | 1033.8 | 776.6 | 1290.9 | 2027 |
| Slovakia | Both | 1020.2 | 732.5 | 1308 | 2028 |
| Slovakia | Both | 1008.8 | 687.3 | 1330.3 | 2029 |
| Slovakia | Both | 999.6 | 640.6 | 1358.6 | 2030 |
| Slovenia | Both | 323.5 | 282.6 | 364.8 | 2018 |
| Slovenia | Both | 323.3 | 281.5 | 365.4 | 2019 |
| Slovenia | Both | 323.1 | 280 | 366.5 | 2020 |
| Slovenia | Both | 322.6 | 277.8 | 367.8 | 2021 |
| Slovenia | Both | 322.1 | 275.1 | 369.5 | 2022 |
| Slovenia | Both | 321.7 | 272 | 371.7 | 2023 |
| Slovenia | Both | 321.3 | 268.6 | 374.5 | 2024 |
| Slovenia | Both | 321.1 | 264.7 | 377.9 | 2025 |
| Slovenia | Both | 320.7 | 260.3 | 381.5 | 2026 |
| Slovenia | Both | 320.5 | 255.5 | 385.8 | 2027 |
| Slovenia | Both | 320.3 | 250.3 | 390.6 | 2028 |
| Slovenia | Both | 320 | 244.6 | 395.9 | 2029 |
| Slovenia | Both | 319.7 | 238.3 | 401.5 | 2030 |
| Solomon Islands | Both | 13.7 | 5.1 | 23.1 | 2018 |
| Solomon Islands | Both | 14.2 | 5.4 | 23.8 | 2019 |
| Solomon Islands | Both | 14.8 | 5.6 | 24.6 | 2020 |
| Solomon Islands | Both | 15.3 | 5.8 | 25.5 | 2021 |
| Solomon Islands | Both | 15.9 | 6 | 26.5 | 2022 |
| Solomon Islands | Both | 16.6 | 6.2 | 27.6 | 2023 |
| Solomon Islands | Both | 17.3 | 6.4 | 28.9 | 2024 |
| Solomon Islands | Both | 18 | 6.5 | 30.4 | 2025 |
| Solomon Islands | Both | 18.9 | 6.4 | 32.1 | 2026 |
| Solomon Islands | Both | 19.9 | 6.6 | 34.3 | 2027 |
| Solomon Islands | Both | 20.9 | 6.7 | 37.1 | 2028 |
| Solomon Islands | Both | 22.2 | 6.8 | 40.8 | 2029 |
| Solomon Islands | Both | 23.6 | 6.7 | 46 | 2030 |
| Somalia | Both | 333.3 | 285 | 381.6 | 2018 |
| Somalia | Both | 339.3 | 287.5 | 391.1 | 2019 |
| Somalia | Both | 345.9 | 289.5 | 402.2 | 2020 |
| Somalia | Both | 353.1 | 291.1 | 415.1 | 2021 |
| Somalia | Both | 360.6 | 291.8 | 429.4 | 2022 |
| Somalia | Both | 368.9 | 292 | 445.8 | 2023 |
| Somalia | Both | 378.2 | 291.7 | 464.7 | 2024 |
| Somalia | Both | 388.9 | 291.1 | 486.8 | 2025 |
| Somalia | Both | 400.8 | 289.5 | 512.1 | 2026 |
| Somalia | Both | 414.3 | 287 | 541.6 | 2027 |
| Somalia | Both | 429.7 | 283.2 | 576.3 | 2028 |
| Somalia | Both | 447.4 | 277.5 | 617.3 | 2029 |
| Somalia | Both | 467.6 | 269.1 | 666.2 | 2030 |
| South Africa | Both | 1728 | 1604.7 | 1851.3 | 2018 |
| South Africa | Both | 1772.6 | 1634.4 | 1910.9 | 2019 |
| South Africa | Both | 1818.3 | 1656.7 | 1980 | 2020 |
| South Africa | Both | 1867.9 | 1673.9 | 2062 | 2021 |
| South Africa | Both | 1919.1 | 1683.8 | 2154.3 | 2022 |
| South Africa | Both | 1972.6 | 1687.3 | 2257.8 | 2023 |
| South Africa | Both | 2029.7 | 1685.1 | 2374.2 | 2024 |
| South Africa | Both | 2091.1 | 1677.2 | 2504.9 | 2025 |
| South Africa | Both | 2161.1 | 1666 | 2656.2 | 2026 |
| South Africa | Both | 2237.2 | 1647.6 | 2826.9 | 2027 |
| South Africa | Both | 2321 | 1620.8 | 3021.2 | 2028 |
| South Africa | Both | 2413.9 | 1583.7 | 3244.1 | 2029 |
| South Africa | Both | 2517.1 | 1532.9 | 3501.3 | 2030 |
| South Korea | Both | 3509.5 | 3351.1 | 3667.9 | 2018 |
| South Korea | Both | 3536.3 | 3360.2 | 3712.5 | 2019 |
| South Korea | Both | 3564.2 | 3360.1 | 3768.3 | 2020 |
| South Korea | Both | 3587.2 | 3345.7 | 3828.7 | 2021 |
| South Korea | Both | 3610.7 | 3323.1 | 3898.3 | 2022 |
| South Korea | Both | 3633.9 | 3292.5 | 3975.4 | 2023 |
| South Korea | Both | 3656.6 | 3254.2 | 4059 | 2024 |
| South Korea | Both | 3679.4 | 3209.4 | 4149.4 | 2025 |
| South Korea | Both | 3697.8 | 3154.1 | 4241.5 | 2026 |
| South Korea | Both | 3718.2 | 3093.8 | 4342.7 | 2027 |
| South Korea | Both | 3742.8 | 3029.7 | 4455.9 | 2028 |
| South Korea | Both | 3773.2 | 2962.6 | 4583.8 | 2029 |
| South Korea | Both | 3810.5 | 2892.1 | 4728.9 | 2030 |
| South Sudan | Both | 250.2 | 210.8 | 289.6 | 2018 |
| South Sudan | Both | 256 | 214.6 | 297.5 | 2019 |
| South Sudan | Both | 262.1 | 217.9 | 306.3 | 2020 |
| South Sudan | Both | 268.6 | 221 | 316.3 | 2021 |
| South Sudan | Both | 275.3 | 223.4 | 327.2 | 2022 |
| South Sudan | Both | 282.3 | 225.3 | 339.2 | 2023 |
| South Sudan | Both | 289.6 | 226.8 | 352.4 | 2024 |
| South Sudan | Both | 297.5 | 227.7 | 367.3 | 2025 |
| South Sudan | Both | 306.1 | 228 | 384.1 | 2026 |
| South Sudan | Both | 315.2 | 227.4 | 403 | 2027 |
| South Sudan | Both | 325 | 225.6 | 424.4 | 2028 |
| South Sudan | Both | 335.7 | 222.3 | 449.1 | 2029 |
| South Sudan | Both | 347.6 | 217 | 478.1 | 2030 |
| Spain | Both | 9894.3 | 9631.8 | 10156.7 | 2018 |
| Spain | Both | 9899.9 | 9603.3 | 10196.4 | 2019 |
| Spain | Both | 9899 | 9552.2 | 10245.8 | 2020 |
| Spain | Both | 9876.1 | 9465.2 | 10287 | 2021 |
| Spain | Both | 9865.1 | 9377 | 10353.1 | 2022 |
| Spain | Both | 9880.1 | 9302.4 | 10457.7 | 2023 |
| Spain | Both | 9915.6 | 9237.8 | 10593.3 | 2024 |
| Spain | Both | 9962.3 | 9174.9 | 10749.7 | 2025 |
| Spain | Both | 9995.6 | 9091.2 | 10900 | 2026 |
| Spain | Both | 10028.8 | 8999.1 | 11058.5 | 2027 |
| Spain | Both | 10062.1 | 8898.6 | 11225.6 | 2028 |
| Spain | Both | 10091.2 | 8786.6 | 11395.7 | 2029 |
| Spain | Both | 10117.5 | 8664.9 | 11570 | 2030 |
| Sri Lanka | Both | 1608.5 | 1490.7 | 1726.3 | 2018 |
| Sri Lanka | Both | 1625.5 | 1468.5 | 1782.4 | 2019 |
| Sri Lanka | Both | 1649.4 | 1433 | 1865.8 | 2020 |
| Sri Lanka | Both | 1678.6 | 1384.4 | 1972.8 | 2021 |
| Sri Lanka | Both | 1720.7 | 1328.2 | 2113.1 | 2022 |
| Sri Lanka | Both | 1778.6 | 1262.3 | 2294.9 | 2023 |
| Sri Lanka | Both | 1856.2 | 1181.1 | 2531.4 | 2024 |
| Sri Lanka | Both | 1959.2 | 1074.4 | 2843.9 | 2025 |
| Sri Lanka | Both | 2091.7 | 922 | 3261.4 | 2026 |
| Sri Lanka | Both | 2266.7 | 691 | 3842.4 | 2027 |
| Sri Lanka | Both | 2498.6 | 316.8 | 4680.5 | 2028 |
| Sri Lanka | Both | 2809.2 | 0 | 5943.5 | 2029 |
| Sri Lanka | Both | 3230.1 | 0 | 7942.1 | 2030 |
| Sudan | Both | 735.5 | 657.9 | 813.1 | 2018 |
| Sudan | Both | 756.7 | 674.8 | 838.6 | 2019 |
| Sudan | Both | 779.6 | 692 | 867.1 | 2020 |
| Sudan | Both | 803.8 | 709.1 | 898.5 | 2021 |
| Sudan | Both | 829.8 | 726.3 | 933.4 | 2022 |
| Sudan | Both | 857.9 | 743.6 | 972.2 | 2023 |
| Sudan | Both | 888.2 | 761 | 1015.4 | 2024 |
| Sudan | Both | 920.9 | 778.5 | 1063.4 | 2025 |
| Sudan | Both | 955.7 | 795.5 | 1115.9 | 2026 |
| Sudan | Both | 993.1 | 812.3 | 1173.9 | 2027 |
| Sudan | Both | 1033.4 | 828.8 | 1237.9 | 2028 |
| Sudan | Both | 1076.9 | 845 | 1308.7 | 2029 |
| Sudan | Both | 1124 | 860.9 | 1387.2 | 2030 |
| Suriname | Both | 30 | 15.5 | 44.8 | 2018 |
| Suriname | Both | 31 | 16.1 | 46 | 2019 |
| Suriname | Both | 31.9 | 16.7 | 47.3 | 2020 |
| Suriname | Both | 32.9 | 17.3 | 48.7 | 2021 |
| Suriname | Both | 34 | 17.9 | 50.2 | 2022 |
| Suriname | Both | 35.1 | 18.5 | 51.9 | 2023 |
| Suriname | Both | 36.3 | 19.1 | 53.7 | 2024 |
| Suriname | Both | 37.5 | 19.5 | 55.7 | 2025 |
| Suriname | Both | 38.8 | 19.9 | 57.9 | 2026 |
| Suriname | Both | 40.1 | 20.2 | 60.3 | 2027 |
| Suriname | Both | 41.6 | 20.3 | 63.1 | 2028 |
| Suriname | Both | 43.1 | 20.2 | 66.3 | 2029 |
| Suriname | Both | 44.8 | 19.8 | 70 | 2030 |
| Swaziland | Both | 33 | 17.2 | 48.9 | 2018 |
| Swaziland | Both | 33 | 17 | 49 | 2019 |
| Swaziland | Both | 33 | 16.8 | 49.3 | 2020 |
| Swaziland | Both | 33.1 | 16.6 | 49.7 | 2021 |
| Swaziland | Both | 33.4 | 16.4 | 50.4 | 2022 |
| Swaziland | Both | 33.7 | 16.2 | 51.2 | 2023 |
| Swaziland | Both | 34.1 | 16 | 52.4 | 2024 |
| Swaziland | Both | 34.7 | 15.6 | 53.9 | 2025 |
| Swaziland | Both | 35.4 | 15.2 | 55.8 | 2026 |
| Swaziland | Both | 36.3 | 14.5 | 58.3 | 2027 |
| Swaziland | Both | 37.4 | 13.6 | 61.4 | 2028 |
| Swaziland | Both | 38.7 | 12.1 | 65.4 | 2029 |
| Swaziland | Both | 40.3 | 10 | 70.8 | 2030 |
| Sweden | Both | 1426.4 | 1334 | 1518.7 | 2018 |
| Sweden | Both | 1436.7 | 1339.9 | 1533.5 | 2019 |
| Sweden | Both | 1446.7 | 1343.6 | 1549.9 | 2020 |
| Sweden | Both | 1454.8 | 1343.4 | 1566.1 | 2021 |
| Sweden | Both | 1463.1 | 1341.6 | 1584.6 | 2022 |
| Sweden | Both | 1472.1 | 1338.6 | 1605.6 | 2023 |
| Sweden | Both | 1481.6 | 1334.3 | 1628.9 | 2024 |
| Sweden | Both | 1491.8 | 1329.1 | 1654.5 | 2025 |
| Sweden | Both | 1500.4 | 1320.7 | 1680 | 2026 |
| Sweden | Both | 1509.8 | 1311.6 | 1708 | 2027 |
| Sweden | Both | 1520.5 | 1302 | 1738.9 | 2028 |
| Sweden | Both | 1533 | 1292.5 | 1773.4 | 2029 |
| Sweden | Both | 1547.8 | 1283.3 | 1812.2 | 2030 |
| Switzerland | Both | 935.1 | 861.3 | 1009 | 2018 |
| Switzerland | Both | 933.5 | 857.2 | 1009.9 | 2019 |
| Switzerland | Both | 932.3 | 852.3 | 1012.3 | 2020 |
| Switzerland | Both | 931 | 846 | 1015.9 | 2021 |
| Switzerland | Both | 929.9 | 838.8 | 1021.1 | 2022 |
| Switzerland | Both | 929.3 | 830.7 | 1028 | 2023 |
| Switzerland | Both | 929.2 | 821.9 | 1036.6 | 2024 |
| Switzerland | Both | 929.8 | 812.6 | 1047.1 | 2025 |
| Switzerland | Both | 929.7 | 801.5 | 1057.8 | 2026 |
| Switzerland | Both | 929.9 | 789.8 | 1070 | 2027 |
| Switzerland | Both | 930.7 | 777.6 | 1083.9 | 2028 |
| Switzerland | Both | 932.4 | 765 | 1099.8 | 2029 |
| Switzerland | Both | 935.1 | 752.2 | 1117.9 | 2030 |
| Syria | Both | 325.6 | 275.6 | 375.6 | 2018 |
| Syria | Both | 343 | 290.8 | 395.2 | 2019 |
| Syria | Both | 365 | 309.7 | 420.3 | 2020 |
| Syria | Both | 391.8 | 332.3 | 451.3 | 2021 |
| Syria | Both | 425.3 | 360 | 490.5 | 2022 |
| Syria | Both | 464 | 391.5 | 536.6 | 2023 |
| Syria | Both | 506.2 | 424.5 | 587.9 | 2024 |
| Syria | Both | 550.2 | 457.5 | 643 | 2025 |
| Syria | Both | 593.6 | 488 | 699.2 | 2026 |
| Syria | Both | 638 | 517.3 | 758.6 | 2027 |
| Syria | Both | 684 | 545.7 | 822.2 | 2028 |
| Syria | Both | 732.4 | 573.5 | 891.3 | 2029 |
| Syria | Both | 784.3 | 601.1 | 967.5 | 2030 |
| Tajikistan | Both | 413.6 | 355.7 | 471.5 | 2018 |
| Tajikistan | Both | 434.9 | 373.9 | 495.9 | 2019 |
| Tajikistan | Both | 457.3 | 392.3 | 522.3 | 2020 |
| Tajikistan | Both | 480.8 | 410.5 | 551.1 | 2021 |
| Tajikistan | Both | 505.5 | 428.5 | 582.6 | 2022 |
| Tajikistan | Both | 531.7 | 446.2 | 617.3 | 2023 |
| Tajikistan | Both | 559.9 | 463.8 | 655.9 | 2024 |
| Tajikistan | Both | 590.3 | 481.4 | 699.2 | 2025 |
| Tajikistan | Both | 622.5 | 498.1 | 746.9 | 2026 |
| Tajikistan | Both | 657.5 | 514.5 | 800.5 | 2027 |
| Tajikistan | Both | 695.6 | 530.2 | 861 | 2028 |
| Tajikistan | Both | 737.1 | 545 | 929.2 | 2029 |
| Tajikistan | Both | 782.4 | 558.3 | 1006.5 | 2030 |
| Tanzania | Both | 1360.7 | 1265.7 | 1455.6 | 2018 |
| Tanzania | Both | 1400.4 | 1297.8 | 1502.9 | 2019 |
| Tanzania | Both | 1442.5 | 1330.2 | 1554.7 | 2020 |
| Tanzania | Both | 1485.5 | 1361.2 | 1609.7 | 2021 |
| Tanzania | Both | 1531.5 | 1393 | 1670 | 2022 |
| Tanzania | Both | 1580.8 | 1425.5 | 1736.1 | 2023 |
| Tanzania | Both | 1633 | 1458.3 | 1807.6 | 2024 |
| Tanzania | Both | 1688 | 1491.2 | 1884.9 | 2025 |
| Tanzania | Both | 1746.2 | 1524.1 | 1968.3 | 2026 |
| Tanzania | Both | 1806.9 | 1556.4 | 2057.5 | 2027 |
| Tanzania | Both | 1870.7 | 1588.4 | 2153.1 | 2028 |
| Tanzania | Both | 1938.4 | 1620.4 | 2256.5 | 2029 |
| Tanzania | Both | 2010.8 | 1652.8 | 2368.7 | 2030 |
| Thailand | Both | 2474.3 | 2334.2 | 2614.3 | 2018 |
| Thailand | Both | 2581 | 2427.7 | 2734.3 | 2019 |
| Thailand | Both | 2692.5 | 2519 | 2866.1 | 2020 |
| Thailand | Both | 2807.8 | 2606.1 | 3009.5 | 2021 |
| Thailand | Both | 2927.7 | 2689.3 | 3166 | 2022 |
| Thailand | Both | 3052.9 | 2769.3 | 3336.6 | 2023 |
| Thailand | Both | 3184.5 | 2846.5 | 3522.5 | 2024 |
| Thailand | Both | 3323 | 2921 | 3724.9 | 2025 |
| Thailand | Both | 3464.8 | 2989.2 | 3940.4 | 2026 |
| Thailand | Both | 3614.1 | 3053.8 | 4174.3 | 2027 |
| Thailand | Both | 3771.5 | 3114.5 | 4428.5 | 2028 |
| Thailand | Both | 3937.8 | 3170.6 | 4704.9 | 2029 |
| Thailand | Both | 4113.4 | 3221.1 | 5005.8 | 2030 |
| The Bahamas | Both | 25.9 | 12.5 | 39.6 | 2018 |
| The Bahamas | Both | 26.5 | 12.9 | 40.4 | 2019 |
| The Bahamas | Both | 27.2 | 13.4 | 41.4 | 2020 |
| The Bahamas | Both | 27.9 | 13.7 | 42.3 | 2021 |
| The Bahamas | Both | 28.6 | 14.1 | 43.3 | 2022 |
| The Bahamas | Both | 29.3 | 14.5 | 44.5 | 2023 |
| The Bahamas | Both | 30.1 | 14.9 | 45.6 | 2024 |
| The Bahamas | Both | 30.9 | 15.2 | 46.9 | 2025 |
| The Bahamas | Both | 31.7 | 15.5 | 48.3 | 2026 |
| The Bahamas | Both | 32.6 | 15.7 | 49.8 | 2027 |
| The Bahamas | Both | 33.4 | 15.8 | 51.4 | 2028 |
| The Bahamas | Both | 34.4 | 15.9 | 53.2 | 2029 |
| The Bahamas | Both | 35.4 | 15.9 | 55.3 | 2030 |
| The Gambia | Both | 34.3 | 19.3 | 49.8 | 2018 |
| The Gambia | Both | 36.1 | 20.4 | 52.1 | 2019 |
| The Gambia | Both | 37.9 | 21.7 | 54.6 | 2020 |
| The Gambia | Both | 40 | 23 | 57.4 | 2021 |
| The Gambia | Both | 42.2 | 24.3 | 60.5 | 2022 |
| The Gambia | Both | 44.6 | 25.8 | 63.9 | 2023 |
| The Gambia | Both | 47.3 | 27.3 | 67.7 | 2024 |
| The Gambia | Both | 50.3 | 28.9 | 72 | 2025 |
| The Gambia | Both | 53.5 | 30.5 | 76.9 | 2026 |
| The Gambia | Both | 57.1 | 32.1 | 82.4 | 2027 |
| The Gambia | Both | 61 | 33.8 | 88.5 | 2028 |
| The Gambia | Both | 65.3 | 35.4 | 95.6 | 2029 |
| The Gambia | Both | 70 | 36.9 | 103.5 | 2030 |
| Timor-Leste | Both | 28.8 | 15.3 | 42.7 | 2018 |
| Timor-Leste | Both | 29.9 | 16.1 | 44.3 | 2019 |
| Timor-Leste | Both | 31.1 | 16.8 | 45.9 | 2020 |
| Timor-Leste | Both | 32.5 | 17.6 | 47.8 | 2021 |
| Timor-Leste | Both | 33.9 | 18.4 | 49.8 | 2022 |
| Timor-Leste | Both | 35.3 | 19.2 | 51.9 | 2023 |
| Timor-Leste | Both | 36.9 | 20 | 54.3 | 2024 |
| Timor-Leste | Both | 38.6 | 20.8 | 56.9 | 2025 |
| Timor-Leste | Both | 40.5 | 21.6 | 59.9 | 2026 |
| Timor-Leste | Both | 42.5 | 22.3 | 63.2 | 2027 |
| Timor-Leste | Both | 44.6 | 22.9 | 66.9 | 2028 |
| Timor-Leste | Both | 46.9 | 23.3 | 71 | 2029 |
| Timor-Leste | Both | 49.5 | 23.6 | 75.8 | 2030 |
| Togo | Both | 147.9 | 115 | 180.8 | 2018 |
| Togo | Both | 153.5 | 119.5 | 187.5 | 2019 |
| Togo | Both | 159.5 | 124.1 | 194.8 | 2020 |
| Togo | Both | 165.7 | 128.7 | 202.6 | 2021 |
| Togo | Both | 172.2 | 133.4 | 211 | 2022 |
| Togo | Both | 179.1 | 138.2 | 220 | 2023 |
| Togo | Both | 186.4 | 143.1 | 229.8 | 2024 |
| Togo | Both | 194.2 | 148 | 240.5 | 2025 |
| Togo | Both | 202.4 | 152.9 | 251.9 | 2026 |
| Togo | Both | 211 | 157.7 | 264.3 | 2027 |
| Togo | Both | 220.1 | 162.4 | 277.8 | 2028 |
| Togo | Both | 229.8 | 167.1 | 292.4 | 2029 |
| Togo | Both | 240.1 | 171.7 | 308.5 | 2030 |
| Trinidad and Tobago | Both | 93 | 66.5 | 119.5 | 2018 |
| Trinidad and Tobago | Both | 95.2 | 68 | 122.4 | 2019 |
| Trinidad and Tobago | Both | 97.5 | 69.4 | 125.6 | 2020 |
| Trinidad and Tobago | Both | 99.8 | 70.6 | 129 | 2021 |
| Trinidad and Tobago | Both | 102.2 | 71.6 | 132.8 | 2022 |
| Trinidad and Tobago | Both | 104.8 | 72.4 | 137.2 | 2023 |
| Trinidad and Tobago | Both | 107.7 | 72.9 | 142.4 | 2024 |
| Trinidad and Tobago | Both | 110.8 | 73.2 | 148.4 | 2025 |
| Trinidad and Tobago | Both | 114.1 | 73 | 155.3 | 2026 |
| Trinidad and Tobago | Both | 117.9 | 72.3 | 163.4 | 2027 |
| Trinidad and Tobago | Both | 122 | 70.9 | 173.1 | 2028 |
| Trinidad and Tobago | Both | 126.6 | 68.6 | 184.7 | 2029 |
| Trinidad and Tobago | Both | 131.8 | 64.8 | 198.7 | 2030 |
| Tunisia | Both | 283.3 | 238.3 | 328.3 | 2018 |
| Tunisia | Both | 292.4 | 246.2 | 338.6 | 2019 |
| Tunisia | Both | 301.7 | 254 | 349.4 | 2020 |
| Tunisia | Both | 311.6 | 262 | 361.2 | 2021 |
| Tunisia | Both | 321.6 | 269.7 | 373.5 | 2022 |
| Tunisia | Both | 331.8 | 277.1 | 386.4 | 2023 |
| Tunisia | Both | 342.3 | 284.4 | 400.3 | 2024 |
| Tunisia | Both | 353.4 | 291.6 | 415.3 | 2025 |
| Tunisia | Both | 364.6 | 298.3 | 431 | 2026 |
| Tunisia | Both | 376.3 | 304.7 | 447.9 | 2027 |
| Tunisia | Both | 388.4 | 310.8 | 466 | 2028 |
| Tunisia | Both | 400.9 | 316.4 | 485.3 | 2029 |
| Tunisia | Both | 413.7 | 321.6 | 505.9 | 2030 |
| Turkey | Both | 3995.4 | 3811.6 | 4179.2 | 2018 |
| Turkey | Both | 4055.9 | 3850.4 | 4261.5 | 2019 |
| Turkey | Both | 4112.5 | 3873.5 | 4351.5 | 2020 |
| Turkey | Both | 4167.1 | 3883.2 | 4451 | 2021 |
| Turkey | Both | 4215.9 | 3876.6 | 4555.2 | 2022 |
| Turkey | Both | 4263.1 | 3858.4 | 4667.8 | 2023 |
| Turkey | Both | 4312.5 | 3833.1 | 4791.8 | 2024 |
| Turkey | Both | 4366.7 | 3803.7 | 4929.7 | 2025 |
| Turkey | Both | 4418.5 | 3763.4 | 5073.7 | 2026 |
| Turkey | Both | 4479 | 3721.3 | 5236.7 | 2027 |
| Turkey | Both | 4549.7 | 3677.5 | 5421.9 | 2028 |
| Turkey | Both | 4627.4 | 3628.4 | 5626.4 | 2029 |
| Turkey | Both | 4709.5 | 3570.7 | 5848.3 | 2030 |
| Turkmenistan | Both | 377.5 | 326 | 428.9 | 2018 |
| Turkmenistan | Both | 389.1 | 335.9 | 442.3 | 2019 |
| Turkmenistan | Both | 400.8 | 345.4 | 456.2 | 2020 |
| Turkmenistan | Both | 412.9 | 354.7 | 471.1 | 2021 |
| Turkmenistan | Both | 424.7 | 363.1 | 486.4 | 2022 |
| Turkmenistan | Both | 436.6 | 370.8 | 502.4 | 2023 |
| Turkmenistan | Both | 449 | 378.2 | 519.7 | 2024 |
| Turkmenistan | Both | 462.1 | 385.6 | 538.7 | 2025 |
| Turkmenistan | Both | 474.5 | 391.4 | 557.7 | 2026 |
| Turkmenistan | Both | 487.7 | 396.9 | 578.4 | 2027 |
| Turkmenistan | Both | 501.6 | 402.1 | 601.1 | 2028 |
| Turkmenistan | Both | 516.3 | 406.9 | 625.7 | 2029 |
| Turkmenistan | Both | 531.6 | 411 | 652.2 | 2030 |
| Uganda | Both | 671 | 603.2 | 738.9 | 2018 |
| Uganda | Both | 673 | 599.8 | 746.3 | 2019 |
| Uganda | Both | 675.8 | 595.8 | 755.8 | 2020 |
| Uganda | Both | 679.6 | 591.6 | 767.6 | 2021 |
| Uganda | Both | 684 | 586.9 | 781.2 | 2022 |
| Uganda | Both | 689.5 | 581.9 | 797 | 2023 |
| Uganda | Both | 696.2 | 577 | 815.4 | 2024 |
| Uganda | Both | 704.6 | 572.3 | 836.9 | 2025 |
| Uganda | Both | 714.5 | 567.5 | 861.5 | 2026 |
| Uganda | Both | 725.9 | 562.5 | 889.3 | 2027 |
| Uganda | Both | 738.9 | 557.2 | 920.7 | 2028 |
| Uganda | Both | 753.9 | 551.5 | 956.3 | 2029 |
| Uganda | Both | 771.1 | 545.2 | 997 | 2030 |
| Ukraine | Both | 6115 | 5745.9 | 6484 | 2018 |
| Ukraine | Both | 5738.2 | 5086.3 | 6390.2 | 2019 |
| Ukraine | Both | 5476.6 | 4468.9 | 6484.4 | 2020 |
| Ukraine | Both | 5311.9 | 3869.4 | 6754.4 | 2021 |
| Ukraine | Both | 5272.6 | 3265.7 | 7279.5 | 2022 |
| Ukraine | Both | 5370.3 | 2571.1 | 8169.4 | 2023 |
| Ukraine | Both | 5638.7 | 1623.2 | 9654.1 | 2024 |
| Ukraine | Both | 6166.2 | 73.2 | 12268.3 | 2025 |
| Ukraine | Both | 7036.3 | 0 | 17066 | 2026 |
| Ukraine | Both | 8472.7 | 0 | 26742 | 2027 |
| Ukraine | Both | 10860.5 | 0 | 48152.5 | 2028 |
| Ukraine | Both | 14977.1 | 0 | 100110.7 | 2029 |
| Ukraine | Both | 22548.7 | 0 | 238057.3 | 2030 |
| United Arab Emirates | Both | 378.9 | 326.1 | 431.7 | 2018 |
| United Arab Emirates | Both | 409 | 351.7 | 466.3 | 2019 |
| United Arab Emirates | Both | 440.4 | 377.3 | 503.6 | 2020 |
| United Arab Emirates | Both | 478 | 406.9 | 549.1 | 2021 |
| United Arab Emirates | Both | 518.2 | 436.8 | 599.6 | 2022 |
| United Arab Emirates | Both | 561 | 466.4 | 655.5 | 2023 |
| United Arab Emirates | Both | 606.6 | 495.7 | 717.5 | 2024 |
| United Arab Emirates | Both | 655.1 | 524.1 | 786.1 | 2025 |
| United Arab Emirates | Both | 713.4 | 556.5 | 870.3 | 2026 |
| United Arab Emirates | Both | 776.3 | 587.5 | 965.1 | 2027 |
| United Arab Emirates | Both | 844.2 | 616.2 | 1072.1 | 2028 |
| United Arab Emirates | Both | 917.7 | 641.6 | 1193.7 | 2029 |
| United Arab Emirates | Both | 997.3 | 662.1 | 1332.4 | 2030 |
| United Kingdom | Both | 10803.3 | 10531.7 | 11074.9 | 2018 |
| United Kingdom | Both | 10998.5 | 10701.5 | 11295.5 | 2019 |
| United Kingdom | Both | 11206.2 | 10871.2 | 11541.2 | 2020 |
| United Kingdom | Both | 11417.6 | 11031.8 | 11803.5 | 2021 |
| United Kingdom | Both | 11637.3 | 11187.7 | 12087 | 2022 |
| United Kingdom | Both | 11865.8 | 11339.9 | 12391.7 | 2023 |
| United Kingdom | Both | 12100.4 | 11486.8 | 12713.9 | 2024 |
| United Kingdom | Both | 12344.7 | 11632.2 | 13057.1 | 2025 |
| United Kingdom | Both | 12585.5 | 11763.8 | 13407.3 | 2026 |
| United Kingdom | Both | 12835.1 | 11892.8 | 13777.4 | 2027 |
| United Kingdom | Both | 13097 | 12022.3 | 14171.7 | 2028 |
| United Kingdom | Both | 13373 | 12153.8 | 14592.3 | 2029 |
| United Kingdom | Both | 13668.5 | 12291.6 | 15045.3 | 2030 |
| United States | Both | 66443.7 | 65651.4 | 67236.1 | 2018 |
| United States | Both | 68488.9 | 67431.6 | 69546.2 | 2019 |
| United States | Both | 70716 | 69263.4 | 72168.6 | 2020 |
| United States | Both | 72950.1 | 70989.3 | 74910.9 | 2021 |
| United States | Both | 75228.4 | 72653.9 | 77802.9 | 2022 |
| United States | Both | 77554.5 | 74255.3 | 80853.6 | 2023 |
| United States | Both | 79922.6 | 75798 | 84047.1 | 2024 |
| United States | Both | 82401.1 | 77348.3 | 87454 | 2025 |
| United States | Both | 84859.5 | 78780.1 | 90938.8 | 2026 |
| United States | Both | 87247.8 | 80043.4 | 94452.2 | 2027 |
| United States | Both | 89583.2 | 81145.2 | 98021.3 | 2028 |
| United States | Both | 92103.9 | 82304.1 | 101903.7 | 2029 |
| United States | Both | 94973.6 | 83657.2 | 106289.9 | 2030 |
| Uruguay | Both | 750.4 | 678.6 | 822.2 | 2018 |
| Uruguay | Both | 766 | 691.3 | 840.7 | 2019 |
| Uruguay | Both | 782.1 | 703.4 | 860.9 | 2020 |
| Uruguay | Both | 797.9 | 713.8 | 882.1 | 2021 |
| Uruguay | Both | 814.2 | 723.3 | 905.1 | 2022 |
| Uruguay | Both | 831 | 731.7 | 930.3 | 2023 |
| Uruguay | Both | 848.6 | 739.3 | 957.8 | 2024 |
| Uruguay | Both | 866.9 | 746 | 987.9 | 2025 |
| Uruguay | Both | 885.2 | 751 | 1019.5 | 2026 |
| Uruguay | Both | 904.4 | 755 | 1053.7 | 2027 |
| Uruguay | Both | 924.6 | 758.1 | 1091 | 2028 |
| Uruguay | Both | 946.1 | 760.4 | 1131.8 | 2029 |
| Uruguay | Both | 969.1 | 761.8 | 1176.5 | 2030 |
| Uzbekistan | Both | 1244 | 1144.6 | 1343.3 | 2018 |
| Uzbekistan | Both | 1289.8 | 1185 | 1394.5 | 2019 |
| Uzbekistan | Both | 1337.5 | 1225.4 | 1449.7 | 2020 |
| Uzbekistan | Both | 1384.4 | 1262.6 | 1506.3 | 2021 |
| Uzbekistan | Both | 1433.2 | 1299.1 | 1567.3 | 2022 |
| Uzbekistan | Both | 1484 | 1334.7 | 1633.2 | 2023 |
| Uzbekistan | Both | 1536.8 | 1369.3 | 1704.3 | 2024 |
| Uzbekistan | Both | 1591.9 | 1403 | 1780.8 | 2025 |
| Uzbekistan | Both | 1645.9 | 1432.5 | 1859.2 | 2026 |
| Uzbekistan | Both | 1702.3 | 1461 | 1943.6 | 2027 |
| Uzbekistan | Both | 1761.8 | 1488.7 | 2034.9 | 2028 |
| Uzbekistan | Both | 1825.2 | 1516.1 | 2134.4 | 2029 |
| Uzbekistan | Both | 1893 | 1543.1 | 2243 | 2030 |
| Venezuela | Both | 2180.4 | 2041.5 | 2319.3 | 2018 |
| Venezuela | Both | 2262.2 | 2101.6 | 2422.8 | 2019 |
| Venezuela | Both | 2349.4 | 2154.2 | 2544.6 | 2020 |
| Venezuela | Both | 2439.1 | 2196.1 | 2682.1 | 2021 |
| Venezuela | Both | 2535.7 | 2231.2 | 2840.2 | 2022 |
| Venezuela | Both | 2639.9 | 2259.4 | 3020.4 | 2023 |
| Venezuela | Both | 2753.3 | 2280.9 | 3225.7 | 2024 |
| Venezuela | Both | 2878.4 | 2295.5 | 3461.3 | 2025 |
| Venezuela | Both | 3012.6 | 2297.9 | 3727.3 | 2026 |
| Venezuela | Both | 3162.3 | 2288.8 | 4035.9 | 2027 |
| Venezuela | Both | 3329.7 | 2263 | 4396.4 | 2028 |
| Venezuela | Both | 3518 | 2214 | 4822.1 | 2029 |
| Venezuela | Both | 3731.9 | 2131.7 | 5332.1 | 2030 |
| Vietnam | Both | 1975.3 | 1854.6 | 2096 | 2018 |
| Vietnam | Both | 2048.3 | 1921.1 | 2175.4 | 2019 |
| Vietnam | Both | 2124.4 | 1988.3 | 2260.5 | 2020 |
| Vietnam | Both | 2202 | 2054.1 | 2350 | 2021 |
| Vietnam | Both | 2281.8 | 2118.7 | 2444.9 | 2022 |
| Vietnam | Both | 2364.2 | 2182.4 | 2546.1 | 2023 |
| Vietnam | Both | 2449.9 | 2245.5 | 2654.3 | 2024 |
| Vietnam | Both | 2539 | 2308.3 | 2769.8 | 2025 |
| Vietnam | Both | 2626.6 | 2365.7 | 2887.4 | 2026 |
| Vietnam | Both | 2717.8 | 2422.7 | 3012.9 | 2027 |
| Vietnam | Both | 2812.1 | 2478.4 | 3145.8 | 2028 |
| Vietnam | Both | 2908.2 | 2531.6 | 3284.9 | 2029 |
| Vietnam | Both | 3005.8 | 2581.6 | 3429.9 | 2030 |
| Yemen | Both | 413.5 | 355 | 471.9 | 2018 |
| Yemen | Both | 427.7 | 366.7 | 488.7 | 2019 |
| Yemen | Both | 442.8 | 378.6 | 507 | 2020 |
| Yemen | Both | 458.7 | 390.5 | 527 | 2021 |
| Yemen | Both | 475.8 | 402.4 | 549.1 | 2022 |
| Yemen | Both | 494 | 414.5 | 573.5 | 2023 |
| Yemen | Both | 513.6 | 426.5 | 600.6 | 2024 |
| Yemen | Both | 534.7 | 438.6 | 630.7 | 2025 |
| Yemen | Both | 557 | 450.3 | 663.8 | 2026 |
| Yemen | Both | 581.1 | 461.9 | 700.4 | 2027 |
| Yemen | Both | 607.1 | 473.1 | 741.1 | 2028 |
| Yemen | Both | 635.3 | 484.1 | 786.5 | 2029 |
| Yemen | Both | 665.8 | 494.5 | 837.1 | 2030 |
| Zambia | Both | 379 | 328.6 | 429.3 | 2018 |
| Zambia | Both | 391.5 | 338.4 | 444.7 | 2019 |
| Zambia | Both | 404.9 | 348.2 | 461.7 | 2020 |
| Zambia | Both | 419.3 | 358.1 | 480.5 | 2021 |
| Zambia | Both | 434.3 | 367.7 | 501 | 2022 |
| Zambia | Both | 450.2 | 377.1 | 523.3 | 2023 |
| Zambia | Both | 467.1 | 386.4 | 547.8 | 2024 |
| Zambia | Both | 485.2 | 395.6 | 574.9 | 2025 |
| Zambia | Both | 504.6 | 404.4 | 604.8 | 2026 |
| Zambia | Both | 525.2 | 412.8 | 637.7 | 2027 |
| Zambia | Both | 547.4 | 420.7 | 674 | 2028 |
| Zambia | Both | 571.2 | 428.1 | 714.3 | 2029 |
| Zambia | Both | 596.9 | 434.5 | 759.3 | 2030 |
| Zimbabwe | Both | 211.5 | 170.8 | 252.1 | 2018 |
| Zimbabwe | Both | 214.4 | 172.4 | 256.5 | 2019 |
| Zimbabwe | Both | 217.7 | 173.8 | 261.6 | 2020 |
| Zimbabwe | Both | 221.1 | 174.7 | 267.5 | 2021 |
| Zimbabwe | Both | 224.9 | 175.4 | 274.5 | 2022 |
| Zimbabwe | Both | 229.3 | 175.8 | 282.9 | 2023 |
| Zimbabwe | Both | 234.3 | 175.8 | 292.8 | 2024 |
| Zimbabwe | Both | 240 | 175.4 | 304.5 | 2025 |
| Zimbabwe | Both | 246.3 | 174.5 | 318.1 | 2026 |
| Zimbabwe | Both | 253.5 | 172.9 | 334.2 | 2027 |
| Zimbabwe | Both | 261.7 | 170.4 | 352.9 | 2028 |
| Zimbabwe | Both | 270.9 | 166.8 | 375.1 | 2029 |
| Zimbabwe | Both | 281.5 | 161.6 | 401.3 | 2030 |
