# Supplementary material for: Trends and projections of kidney cancer incidence at the global and national levels, 1990–2030: a Bayesian age-period-cohort modeling study
Source: Biomark Res. 2020 May 13;8:16. doi: 10.1186/s40364-020-00195-3 (PMC7222434; doi:10.1186/s40364-020-00195-3)
Supplement: Supplementary file 4 — Additional file 4: Table S3. The age-standardized incidence rate of kidney cancer at the national level from 1990 to 2030. [file 40364_2020_195_MOESM4_ESM.docx]

S-Table 3. The age-standardized incidence rate of kidney cancer at the national level from 1990 to 2030.

| Region | Incidence estimate | 95 % CI | | Year |
| --- | --- | --- | --- | --- |
| Afghanistan | 4.49 | 4.36 | 4.63 | 1990 |
| Afghanistan | 4.28 | 4.18 | 4.38 | 1991 |
| Afghanistan | 4.09 | 4.01 | 4.17 | 1992 |
| Afghanistan | 3.91 | 3.85 | 3.98 | 1993 |
| Afghanistan | 3.76 | 3.7 | 3.82 | 1994 |
| Afghanistan | 3.63 | 3.57 | 3.69 | 1995 |
| Afghanistan | 3.51 | 3.46 | 3.56 | 1996 |
| Afghanistan | 3.41 | 3.35 | 3.46 | 1997 |
| Afghanistan | 3.32 | 3.27 | 3.37 | 1998 |
| Afghanistan | 3.24 | 3.2 | 3.29 | 1999 |
| Afghanistan | 3.19 | 3.14 | 3.24 | 2000 |
| Afghanistan | 3.16 | 3.11 | 3.2 | 2001 |
| Afghanistan | 3.15 | 3.1 | 3.19 | 2002 |
| Afghanistan | 3.15 | 3.11 | 3.2 | 2003 |
| Afghanistan | 3.16 | 3.12 | 3.21 | 2004 |
| Afghanistan | 3.17 | 3.12 | 3.21 | 2005 |
| Afghanistan | 3.15 | 3.11 | 3.2 | 2006 |
| Afghanistan | 3.14 | 3.09 | 3.18 | 2007 |
| Afghanistan | 3.11 | 3.07 | 3.15 | 2008 |
| Afghanistan | 3.09 | 3.05 | 3.13 | 2009 |
| Afghanistan | 3.07 | 3.03 | 3.11 | 2010 |
| Afghanistan | 3.06 | 3.02 | 3.1 | 2011 |
| Afghanistan | 3.06 | 3.02 | 3.1 | 2012 |
| Afghanistan | 3.06 | 3.03 | 3.1 | 2013 |
| Afghanistan | 3.07 | 3.03 | 3.11 | 2014 |
| Afghanistan | 3.07 | 3.03 | 3.12 | 2015 |
| Afghanistan | 3.08 | 3.04 | 3.13 | 2016 |
| Afghanistan | 3.09 | 3.04 | 3.15 | 2017 |
| Afghanistan | 3.11 | 3.03 | 3.19 | 2018 |
| Afghanistan | 3.12 | 3.01 | 3.23 | 2019 |
| Afghanistan | 3.14 | 2.99 | 3.28 | 2020 |
| Afghanistan | 3.15 | 2.97 | 3.34 | 2021 |
| Afghanistan | 3.17 | 2.94 | 3.4 | 2022 |
| Afghanistan | 3.19 | 2.91 | 3.47 | 2023 |
| Afghanistan | 3.21 | 2.88 | 3.54 | 2024 |
| Afghanistan | 3.23 | 2.85 | 3.62 | 2025 |
| Afghanistan | 3.25 | 2.81 | 3.7 | 2026 |
| Afghanistan | 3.28 | 2.77 | 3.79 | 2027 |
| Afghanistan | 3.31 | 2.73 | 3.89 | 2028 |
| Afghanistan | 3.34 | 2.7 | 3.99 | 2029 |
| Afghanistan | 3.38 | 2.65 | 4.1 | 2030 |
| Albania | 3.3 | 3.15 | 3.44 | 1990 |
| Albania | 3.34 | 3.22 | 3.45 | 1991 |
| Albania | 3.37 | 3.27 | 3.47 | 1992 |
| Albania | 3.41 | 3.32 | 3.5 | 1993 |
| Albania | 3.46 | 3.37 | 3.55 | 1994 |
| Albania | 3.51 | 3.43 | 3.6 | 1995 |
| Albania | 3.58 | 3.5 | 3.67 | 1996 |
| Albania | 3.67 | 3.58 | 3.76 | 1997 |
| Albania | 3.77 | 3.68 | 3.86 | 1998 |
| Albania | 3.89 | 3.8 | 3.98 | 1999 |
| Albania | 4.02 | 3.93 | 4.11 | 2000 |
| Albania | 4.17 | 4.08 | 4.26 | 2001 |
| Albania | 4.32 | 4.23 | 4.41 | 2002 |
| Albania | 4.48 | 4.38 | 4.57 | 2003 |
| Albania | 4.62 | 4.53 | 4.72 | 2004 |
| Albania | 4.75 | 4.66 | 4.85 | 2005 |
| Albania | 4.86 | 4.76 | 4.96 | 2006 |
| Albania | 4.93 | 4.83 | 5.03 | 2007 |
| Albania | 4.97 | 4.87 | 5.07 | 2008 |
| Albania | 4.98 | 4.88 | 5.07 | 2009 |
| Albania | 4.96 | 4.87 | 5.06 | 2010 |
| Albania | 4.93 | 4.84 | 5.03 | 2011 |
| Albania | 4.9 | 4.8 | 4.99 | 2012 |
| Albania | 4.86 | 4.76 | 4.95 | 2013 |
| Albania | 4.81 | 4.72 | 4.91 | 2014 |
| Albania | 4.77 | 4.67 | 4.87 | 2015 |
| Albania | 4.73 | 4.61 | 4.85 | 2016 |
| Albania | 4.69 | 4.54 | 4.84 | 2017 |
| Albania | 4.65 | 4.45 | 4.84 | 2018 |
| Albania | 4.6 | 4.35 | 4.86 | 2019 |
| Albania | 4.56 | 4.24 | 4.88 | 2020 |
| Albania | 4.52 | 4.13 | 4.91 | 2021 |
| Albania | 4.48 | 4.01 | 4.95 | 2022 |
| Albania | 4.44 | 3.89 | 4.99 | 2023 |
| Albania | 4.4 | 3.76 | 5.04 | 2024 |
| Albania | 4.36 | 3.63 | 5.08 | 2025 |
| Albania | 4.32 | 3.5 | 5.14 | 2026 |
| Albania | 4.28 | 3.36 | 5.19 | 2027 |
| Albania | 4.24 | 3.22 | 5.25 | 2028 |
| Albania | 4.19 | 3.08 | 5.3 | 2029 |
| Albania | 4.15 | 2.94 | 5.36 | 2030 |
| Algeria | 1.41 | 1.37 | 1.45 | 1990 |
| Algeria | 1.43 | 1.4 | 1.46 | 1991 |
| Algeria | 1.45 | 1.42 | 1.48 | 1992 |
| Algeria | 1.47 | 1.45 | 1.49 | 1993 |
| Algeria | 1.49 | 1.47 | 1.52 | 1994 |
| Algeria | 1.52 | 1.49 | 1.54 | 1995 |
| Algeria | 1.54 | 1.52 | 1.56 | 1996 |
| Algeria | 1.57 | 1.55 | 1.59 | 1997 |
| Algeria | 1.59 | 1.57 | 1.62 | 1998 |
| Algeria | 1.62 | 1.6 | 1.64 | 1999 |
| Algeria | 1.65 | 1.63 | 1.67 | 2000 |
| Algeria | 1.68 | 1.66 | 1.7 | 2001 |
| Algeria | 1.71 | 1.69 | 1.73 | 2002 |
| Algeria | 1.74 | 1.72 | 1.76 | 2003 |
| Algeria | 1.77 | 1.74 | 1.79 | 2004 |
| Algeria | 1.79 | 1.77 | 1.81 | 2005 |
| Algeria | 1.81 | 1.79 | 1.84 | 2006 |
| Algeria | 1.83 | 1.81 | 1.85 | 2007 |
| Algeria | 1.85 | 1.83 | 1.87 | 2008 |
| Algeria | 1.86 | 1.84 | 1.89 | 2009 |
| Algeria | 1.88 | 1.86 | 1.9 | 2010 |
| Algeria | 1.89 | 1.87 | 1.91 | 2011 |
| Algeria | 1.89 | 1.87 | 1.92 | 2012 |
| Algeria | 1.9 | 1.88 | 1.92 | 2013 |
| Algeria | 1.91 | 1.89 | 1.93 | 2014 |
| Algeria | 1.91 | 1.89 | 1.94 | 2015 |
| Algeria | 1.92 | 1.89 | 1.94 | 2016 |
| Algeria | 1.92 | 1.89 | 1.96 | 2017 |
| Algeria | 1.93 | 1.89 | 1.97 | 2018 |
| Algeria | 1.93 | 1.88 | 1.99 | 2019 |
| Algeria | 1.94 | 1.87 | 2.01 | 2020 |
| Algeria | 1.94 | 1.85 | 2.03 | 2021 |
| Algeria | 1.95 | 1.84 | 2.06 | 2022 |
| Algeria | 1.96 | 1.83 | 2.09 | 2023 |
| Algeria | 1.96 | 1.81 | 2.11 | 2024 |
| Algeria | 1.97 | 1.79 | 2.14 | 2025 |
| Algeria | 1.97 | 1.77 | 2.17 | 2026 |
| Algeria | 1.98 | 1.75 | 2.21 | 2027 |
| Algeria | 1.99 | 1.73 | 2.24 | 2028 |
| Algeria | 1.99 | 1.71 | 2.27 | 2029 |
| Algeria | 2 | 1.69 | 2.31 | 2030 |
| Angola | 2.37 | 2.3 | 2.44 | 1990 |
| Angola | 2.41 | 2.35 | 2.47 | 1991 |
| Angola | 2.45 | 2.4 | 2.5 | 1992 |
| Angola | 2.49 | 2.45 | 2.54 | 1993 |
| Angola | 2.53 | 2.49 | 2.58 | 1994 |
| Angola | 2.58 | 2.53 | 2.62 | 1995 |
| Angola | 2.62 | 2.58 | 2.66 | 1996 |
| Angola | 2.66 | 2.62 | 2.7 | 1997 |
| Angola | 2.71 | 2.67 | 2.75 | 1998 |
| Angola | 2.75 | 2.71 | 2.79 | 1999 |
| Angola | 2.8 | 2.76 | 2.84 | 2000 |
| Angola | 2.84 | 2.81 | 2.88 | 2001 |
| Angola | 2.89 | 2.85 | 2.93 | 2002 |
| Angola | 2.93 | 2.89 | 2.97 | 2003 |
| Angola | 2.98 | 2.94 | 3.01 | 2004 |
| Angola | 3.01 | 2.98 | 3.05 | 2005 |
| Angola | 3.05 | 3.01 | 3.09 | 2006 |
| Angola | 3.08 | 3.04 | 3.11 | 2007 |
| Angola | 3.1 | 3.06 | 3.14 | 2008 |
| Angola | 3.12 | 3.08 | 3.16 | 2009 |
| Angola | 3.14 | 3.1 | 3.18 | 2010 |
| Angola | 3.16 | 3.12 | 3.2 | 2011 |
| Angola | 3.18 | 3.14 | 3.21 | 2012 |
| Angola | 3.19 | 3.16 | 3.23 | 2013 |
| Angola | 3.21 | 3.17 | 3.25 | 2014 |
| Angola | 3.23 | 3.19 | 3.27 | 2015 |
| Angola | 3.25 | 3.2 | 3.3 | 2016 |
| Angola | 3.27 | 3.2 | 3.33 | 2017 |
| Angola | 3.29 | 3.21 | 3.37 | 2018 |
| Angola | 3.31 | 3.2 | 3.41 | 2019 |
| Angola | 3.33 | 3.2 | 3.45 | 2020 |
| Angola | 3.35 | 3.19 | 3.5 | 2021 |
| Angola | 3.37 | 3.18 | 3.55 | 2022 |
| Angola | 3.39 | 3.17 | 3.61 | 2023 |
| Angola | 3.41 | 3.15 | 3.67 | 2024 |
| Angola | 3.43 | 3.14 | 3.73 | 2025 |
| Angola | 3.45 | 3.12 | 3.79 | 2026 |
| Angola | 3.48 | 3.1 | 3.86 | 2027 |
| Angola | 3.5 | 3.07 | 3.92 | 2028 |
| Angola | 3.52 | 3.05 | 3.99 | 2029 |
| Angola | 3.54 | 3.02 | 4.07 | 2030 |
| Argentina | 13.38 | 13.26 | 13.5 | 1990 |
| Argentina | 13.61 | 13.52 | 13.7 | 1991 |
| Argentina | 13.79 | 13.7 | 13.88 | 1992 |
| Argentina | 13.78 | 13.69 | 13.87 | 1993 |
| Argentina | 13.55 | 13.47 | 13.64 | 1994 |
| Argentina | 13.08 | 12.99 | 13.16 | 1995 |
| Argentina | 12.38 | 12.3 | 12.46 | 1996 |
| Argentina | 11.58 | 11.5 | 11.65 | 1997 |
| Argentina | 10.98 | 10.91 | 11.06 | 1998 |
| Argentina | 10.66 | 10.59 | 10.73 | 1999 |
| Argentina | 10.55 | 10.48 | 10.62 | 2000 |
| Argentina | 10.62 | 10.55 | 10.69 | 2001 |
| Argentina | 10.78 | 10.71 | 10.85 | 2002 |
| Argentina | 10.91 | 10.83 | 10.98 | 2003 |
| Argentina | 11 | 10.93 | 11.07 | 2004 |
| Argentina | 11.13 | 11.06 | 11.2 | 2005 |
| Argentina | 11.31 | 11.23 | 11.38 | 2006 |
| Argentina | 11.46 | 11.39 | 11.53 | 2007 |
| Argentina | 11.54 | 11.47 | 11.61 | 2008 |
| Argentina | 11.62 | 11.55 | 11.69 | 2009 |
| Argentina | 11.71 | 11.64 | 11.78 | 2010 |
| Argentina | 11.83 | 11.75 | 11.9 | 2011 |
| Argentina | 11.94 | 11.87 | 12.01 | 2012 |
| Argentina | 12 | 11.93 | 12.07 | 2013 |
| Argentina | 12.01 | 11.94 | 12.08 | 2014 |
| Argentina | 12.03 | 11.96 | 12.11 | 2015 |
| Argentina | 12.1 | 12.02 | 12.17 | 2016 |
| Argentina | 12.14 | 12.04 | 12.23 | 2017 |
| Argentina | 12.17 | 11.95 | 12.39 | 2018 |
| Argentina | 12.2 | 11.8 | 12.6 | 2019 |
| Argentina | 12.22 | 11.59 | 12.84 | 2020 |
| Argentina | 12.22 | 11.35 | 13.1 | 2021 |
| Argentina | 12.22 | 11.07 | 13.37 | 2022 |
| Argentina | 12.2 | 10.75 | 13.65 | 2023 |
| Argentina | 12.18 | 10.41 | 13.95 | 2024 |
| Argentina | 12.15 | 10.04 | 14.26 | 2025 |
| Argentina | 12.11 | 9.64 | 14.57 | 2026 |
| Argentina | 12.06 | 9.22 | 14.89 | 2027 |
| Argentina | 12 | 8.78 | 15.21 | 2028 |
| Argentina | 11.93 | 8.32 | 15.54 | 2029 |
| Argentina | 11.86 | 7.85 | 15.87 | 2030 |
| Armenia | 1.57 | 1.46 | 1.68 | 1990 |
| Armenia | 1.59 | 1.51 | 1.67 | 1991 |
| Armenia | 1.61 | 1.54 | 1.68 | 1992 |
| Armenia | 1.62 | 1.55 | 1.69 | 1993 |
| Armenia | 1.62 | 1.55 | 1.69 | 1994 |
| Armenia | 1.62 | 1.55 | 1.69 | 1995 |
| Armenia | 1.61 | 1.54 | 1.67 | 1996 |
| Armenia | 1.6 | 1.53 | 1.67 | 1997 |
| Armenia | 1.61 | 1.54 | 1.67 | 1998 |
| Armenia | 1.63 | 1.56 | 1.7 | 1999 |
| Armenia | 1.68 | 1.61 | 1.75 | 2000 |
| Armenia | 1.75 | 1.68 | 1.82 | 2001 |
| Armenia | 1.84 | 1.76 | 1.91 | 2002 |
| Armenia | 1.93 | 1.86 | 2.01 | 2003 |
| Armenia | 2.03 | 1.95 | 2.11 | 2004 |
| Armenia | 2.15 | 2.07 | 2.23 | 2005 |
| Armenia | 2.28 | 2.2 | 2.36 | 2006 |
| Armenia | 2.45 | 2.36 | 2.54 | 2007 |
| Armenia | 2.67 | 2.58 | 2.76 | 2008 |
| Armenia | 2.98 | 2.88 | 3.08 | 2009 |
| Armenia | 3.43 | 3.32 | 3.54 | 2010 |
| Armenia | 4.06 | 3.94 | 4.18 | 2011 |
| Armenia | 4.87 | 4.73 | 5 | 2012 |
| Armenia | 5.69 | 5.54 | 5.85 | 2013 |
| Armenia | 6.37 | 6.2 | 6.54 | 2014 |
| Armenia | 6.81 | 6.64 | 6.98 | 2015 |
| Armenia | 6.97 | 6.79 | 7.14 | 2016 |
| Armenia | 6.95 | 6.69 | 7.2 | 2017 |
| Armenia | 6.92 | 6.45 | 7.4 | 2018 |
| Armenia | 6.89 | 6.12 | 7.66 | 2019 |
| Armenia | 6.85 | 5.73 | 7.97 | 2020 |
| Armenia | 6.81 | 5.31 | 8.31 | 2021 |
| Armenia | 6.76 | 4.85 | 8.68 | 2022 |
| Armenia | 6.71 | 4.36 | 9.07 | 2023 |
| Armenia | 6.66 | 3.84 | 9.47 | 2024 |
| Armenia | 6.6 | 3.3 | 9.89 | 2025 |
| Armenia | 6.53 | 2.75 | 10.32 | 2026 |
| Armenia | 6.47 | 2.17 | 10.76 | 2027 |
| Armenia | 6.4 | 1.58 | 11.21 | 2028 |
| Armenia | 6.33 | 0.99 | 11.67 | 2029 |
| Armenia | 6.25 | 0.38 | 12.12 | 2030 |
| Australia | 6.63 | 6.54 | 6.71 | 1990 |
| Australia | 6.85 | 6.79 | 6.92 | 1991 |
| Australia | 7.08 | 7.02 | 7.14 | 1992 |
| Australia | 7.31 | 7.25 | 7.36 | 1993 |
| Australia | 7.53 | 7.47 | 7.58 | 1994 |
| Australia | 7.74 | 7.68 | 7.79 | 1995 |
| Australia | 7.94 | 7.88 | 7.99 | 1996 |
| Australia | 8.12 | 8.07 | 8.18 | 1997 |
| Australia | 8.29 | 8.24 | 8.35 | 1998 |
| Australia | 8.45 | 8.4 | 8.51 | 1999 |
| Australia | 8.6 | 8.54 | 8.65 | 2000 |
| Australia | 8.73 | 8.67 | 8.78 | 2001 |
| Australia | 8.84 | 8.79 | 8.9 | 2002 |
| Australia | 8.94 | 8.89 | 9 | 2003 |
| Australia | 9.02 | 8.97 | 9.08 | 2004 |
| Australia | 9.09 | 9.03 | 9.14 | 2005 |
| Australia | 9.14 | 9.08 | 9.19 | 2006 |
| Australia | 9.17 | 9.11 | 9.22 | 2007 |
| Australia | 9.17 | 9.11 | 9.22 | 2008 |
| Australia | 9.14 | 9.08 | 9.19 | 2009 |
| Australia | 9.08 | 9.03 | 9.13 | 2010 |
| Australia | 9.01 | 8.95 | 9.06 | 2011 |
| Australia | 8.93 | 8.88 | 8.98 | 2012 |
| Australia | 8.86 | 8.81 | 8.91 | 2013 |
| Australia | 8.8 | 8.75 | 8.85 | 2014 |
| Australia | 8.75 | 8.7 | 8.8 | 2015 |
| Australia | 8.71 | 8.65 | 8.77 | 2016 |
| Australia | 8.69 | 8.61 | 8.77 | 2017 |
| Australia | 8.66 | 8.54 | 8.78 | 2018 |
| Australia | 8.64 | 8.47 | 8.82 | 2019 |
| Australia | 8.63 | 8.39 | 8.86 | 2020 |
| Australia | 8.61 | 8.31 | 8.92 | 2021 |
| Australia | 8.6 | 8.22 | 8.98 | 2022 |
| Australia | 8.59 | 8.12 | 9.06 | 2023 |
| Australia | 8.58 | 8.03 | 9.14 | 2024 |
| Australia | 8.58 | 7.93 | 9.23 | 2025 |
| Australia | 8.57 | 7.82 | 9.32 | 2026 |
| Australia | 8.57 | 7.71 | 9.42 | 2027 |
| Australia | 8.56 | 7.6 | 9.52 | 2028 |
| Australia | 8.56 | 7.49 | 9.63 | 2029 |
| Australia | 8.56 | 7.37 | 9.75 | 2030 |
| Austria | 9.48 | 9.35 | 9.62 | 1990 |
| Austria | 9.35 | 9.25 | 9.45 | 1991 |
| Austria | 9.21 | 9.13 | 9.3 | 1992 |
| Austria | 9.08 | 8.99 | 9.16 | 1993 |
| Austria | 8.94 | 8.86 | 9.01 | 1994 |
| Austria | 8.8 | 8.72 | 8.87 | 1995 |
| Austria | 8.67 | 8.59 | 8.74 | 1996 |
| Austria | 8.55 | 8.47 | 8.62 | 1997 |
| Austria | 8.45 | 8.38 | 8.53 | 1998 |
| Austria | 8.38 | 8.3 | 8.45 | 1999 |
| Austria | 8.32 | 8.25 | 8.39 | 2000 |
| Austria | 8.28 | 8.21 | 8.35 | 2001 |
| Austria | 8.25 | 8.18 | 8.32 | 2002 |
| Austria | 8.22 | 8.15 | 8.29 | 2003 |
| Austria | 8.18 | 8.11 | 8.25 | 2004 |
| Austria | 8.12 | 8.05 | 8.19 | 2005 |
| Austria | 8.05 | 7.98 | 8.12 | 2006 |
| Austria | 7.97 | 7.9 | 8.04 | 2007 |
| Austria | 7.88 | 7.81 | 7.95 | 2008 |
| Austria | 7.78 | 7.71 | 7.85 | 2009 |
| Austria | 7.67 | 7.6 | 7.73 | 2010 |
| Austria | 7.55 | 7.48 | 7.61 | 2011 |
| Austria | 7.42 | 7.36 | 7.49 | 2012 |
| Austria | 7.3 | 7.24 | 7.36 | 2013 |
| Austria | 7.18 | 7.12 | 7.24 | 2014 |
| Austria | 7.07 | 7 | 7.13 | 2015 |
| Austria | 6.96 | 6.88 | 7.03 | 2016 |
| Austria | 6.85 | 6.75 | 6.95 | 2017 |
| Austria | 6.74 | 6.61 | 6.87 | 2018 |
| Austria | 6.64 | 6.47 | 6.81 | 2019 |
| Austria | 6.54 | 6.33 | 6.76 | 2020 |
| Austria | 6.44 | 6.17 | 6.71 | 2021 |
| Austria | 6.34 | 6.02 | 6.66 | 2022 |
| Austria | 6.24 | 5.86 | 6.61 | 2023 |
| Austria | 6.14 | 5.71 | 6.57 | 2024 |
| Austria | 6.04 | 5.55 | 6.53 | 2025 |
| Austria | 5.93 | 5.38 | 6.49 | 2026 |
| Austria | 5.84 | 5.22 | 6.45 | 2027 |
| Austria | 5.74 | 5.07 | 6.41 | 2028 |
| Austria | 5.65 | 4.91 | 6.38 | 2029 |
| Austria | 5.55 | 4.75 | 6.35 | 2030 |
| Azerbaijan | 9.11 | 8.91 | 9.32 | 1990 |
| Azerbaijan | 9.44 | 9.29 | 9.59 | 1991 |
| Azerbaijan | 9.74 | 9.61 | 9.88 | 1992 |
| Azerbaijan | 9.97 | 9.83 | 10.1 | 1993 |
| Azerbaijan | 10.08 | 9.95 | 10.22 | 1994 |
| Azerbaijan | 10.07 | 9.94 | 10.2 | 1995 |
| Azerbaijan | 9.97 | 9.85 | 10.1 | 1996 |
| Azerbaijan | 9.83 | 9.71 | 9.96 | 1997 |
| Azerbaijan | 9.67 | 9.54 | 9.79 | 1998 |
| Azerbaijan | 9.5 | 9.38 | 9.62 | 1999 |
| Azerbaijan | 9.33 | 9.21 | 9.45 | 2000 |
| Azerbaijan | 9.15 | 9.03 | 9.27 | 2001 |
| Azerbaijan | 8.95 | 8.83 | 9.06 | 2002 |
| Azerbaijan | 8.7 | 8.59 | 8.81 | 2003 |
| Azerbaijan | 8.42 | 8.31 | 8.53 | 2004 |
| Azerbaijan | 8.15 | 8.04 | 8.25 | 2005 |
| Azerbaijan | 7.91 | 7.81 | 8.02 | 2006 |
| Azerbaijan | 7.76 | 7.66 | 7.87 | 2007 |
| Azerbaijan | 7.73 | 7.63 | 7.83 | 2008 |
| Azerbaijan | 7.79 | 7.69 | 7.89 | 2009 |
| Azerbaijan | 7.9 | 7.81 | 8 | 2010 |
| Azerbaijan | 8.04 | 7.94 | 8.14 | 2011 |
| Azerbaijan | 8.17 | 8.07 | 8.27 | 2012 |
| Azerbaijan | 8.29 | 8.19 | 8.39 | 2013 |
| Azerbaijan | 8.39 | 8.29 | 8.49 | 2014 |
| Azerbaijan | 8.49 | 8.39 | 8.59 | 2015 |
| Azerbaijan | 8.58 | 8.46 | 8.69 | 2016 |
| Azerbaijan | 8.66 | 8.51 | 8.81 | 2017 |
| Azerbaijan | 8.75 | 8.5 | 8.99 | 2018 |
| Azerbaijan | 8.83 | 8.46 | 9.21 | 2019 |
| Azerbaijan | 8.92 | 8.39 | 9.45 | 2020 |
| Azerbaijan | 9.02 | 8.31 | 9.73 | 2021 |
| Azerbaijan | 9.11 | 8.21 | 10.02 | 2022 |
| Azerbaijan | 9.21 | 8.09 | 10.34 | 2023 |
| Azerbaijan | 9.31 | 7.95 | 10.68 | 2024 |
| Azerbaijan | 9.42 | 7.8 | 11.04 | 2025 |
| Azerbaijan | 9.53 | 7.63 | 11.43 | 2026 |
| Azerbaijan | 9.64 | 7.44 | 11.84 | 2027 |
| Azerbaijan | 9.75 | 7.24 | 12.27 | 2028 |
| Azerbaijan | 9.87 | 7.02 | 12.72 | 2029 |
| Azerbaijan | 9.99 | 6.79 | 13.2 | 2030 |
| Bahrain | 4.74 | 4.28 | 5.21 | 1990 |
| Bahrain | 4.77 | 4.36 | 5.18 | 1991 |
| Bahrain | 4.79 | 4.42 | 5.15 | 1992 |
| Bahrain | 4.81 | 4.47 | 5.14 | 1993 |
| Bahrain | 4.82 | 4.52 | 5.13 | 1994 |
| Bahrain | 4.83 | 4.55 | 5.11 | 1995 |
| Bahrain | 4.84 | 4.57 | 5.1 | 1996 |
| Bahrain | 4.84 | 4.59 | 5.09 | 1997 |
| Bahrain | 4.83 | 4.59 | 5.08 | 1998 |
| Bahrain | 4.82 | 4.58 | 5.05 | 1999 |
| Bahrain | 4.79 | 4.57 | 5.02 | 2000 |
| Bahrain | 4.76 | 4.54 | 4.98 | 2001 |
| Bahrain | 4.71 | 4.5 | 4.93 | 2002 |
| Bahrain | 4.65 | 4.45 | 4.86 | 2003 |
| Bahrain | 4.59 | 4.39 | 4.79 | 2004 |
| Bahrain | 4.51 | 4.32 | 4.71 | 2005 |
| Bahrain | 4.43 | 4.24 | 4.62 | 2006 |
| Bahrain | 4.35 | 4.17 | 4.54 | 2007 |
| Bahrain | 4.27 | 4.09 | 4.45 | 2008 |
| Bahrain | 4.18 | 4.01 | 4.36 | 2009 |
| Bahrain | 4.1 | 3.93 | 4.27 | 2010 |
| Bahrain | 4.03 | 3.86 | 4.19 | 2011 |
| Bahrain | 3.95 | 3.78 | 4.12 | 2012 |
| Bahrain | 3.89 | 3.71 | 4.06 | 2013 |
| Bahrain | 3.82 | 3.64 | 4 | 2014 |
| Bahrain | 3.76 | 3.57 | 3.95 | 2015 |
| Bahrain | 3.71 | 3.5 | 3.91 | 2016 |
| Bahrain | 3.65 | 3.42 | 3.88 | 2017 |
| Bahrain | 3.6 | 3.34 | 3.86 | 2018 |
| Bahrain | 3.55 | 3.25 | 3.84 | 2019 |
| Bahrain | 3.49 | 3.16 | 3.83 | 2020 |
| Bahrain | 3.44 | 3.06 | 3.83 | 2021 |
| Bahrain | 3.4 | 2.97 | 3.82 | 2022 |
| Bahrain | 3.35 | 2.87 | 3.82 | 2023 |
| Bahrain | 3.3 | 2.77 | 3.83 | 2024 |
| Bahrain | 3.26 | 2.68 | 3.83 | 2025 |
| Bahrain | 3.21 | 2.58 | 3.84 | 2026 |
| Bahrain | 3.17 | 2.48 | 3.85 | 2027 |
| Bahrain | 3.12 | 2.38 | 3.86 | 2028 |
| Bahrain | 3.08 | 2.28 | 3.88 | 2029 |
| Bahrain | 3.04 | 2.19 | 3.89 | 2030 |
| Bangladesh | 1.51 | 1.48 | 1.53 | 1990 |
| Bangladesh | 1.46 | 1.45 | 1.48 | 1991 |
| Bangladesh | 1.43 | 1.41 | 1.44 | 1992 |
| Bangladesh | 1.39 | 1.38 | 1.41 | 1993 |
| Bangladesh | 1.37 | 1.35 | 1.38 | 1994 |
| Bangladesh | 1.34 | 1.33 | 1.36 | 1995 |
| Bangladesh | 1.33 | 1.32 | 1.34 | 1996 |
| Bangladesh | 1.31 | 1.3 | 1.33 | 1997 |
| Bangladesh | 1.31 | 1.3 | 1.32 | 1998 |
| Bangladesh | 1.31 | 1.3 | 1.32 | 1999 |
| Bangladesh | 1.32 | 1.3 | 1.33 | 2000 |
| Bangladesh | 1.33 | 1.32 | 1.34 | 2001 |
| Bangladesh | 1.34 | 1.33 | 1.36 | 2002 |
| Bangladesh | 1.36 | 1.35 | 1.38 | 2003 |
| Bangladesh | 1.39 | 1.38 | 1.4 | 2004 |
| Bangladesh | 1.41 | 1.4 | 1.42 | 2005 |
| Bangladesh | 1.43 | 1.42 | 1.44 | 2006 |
| Bangladesh | 1.45 | 1.44 | 1.46 | 2007 |
| Bangladesh | 1.46 | 1.45 | 1.48 | 2008 |
| Bangladesh | 1.47 | 1.46 | 1.49 | 2009 |
| Bangladesh | 1.48 | 1.47 | 1.49 | 2010 |
| Bangladesh | 1.48 | 1.47 | 1.49 | 2011 |
| Bangladesh | 1.48 | 1.47 | 1.49 | 2012 |
| Bangladesh | 1.49 | 1.47 | 1.5 | 2013 |
| Bangladesh | 1.49 | 1.48 | 1.5 | 2014 |
| Bangladesh | 1.5 | 1.49 | 1.51 | 2015 |
| Bangladesh | 1.51 | 1.49 | 1.52 | 2016 |
| Bangladesh | 1.51 | 1.5 | 1.53 | 2017 |
| Bangladesh | 1.52 | 1.5 | 1.55 | 2018 |
| Bangladesh | 1.53 | 1.49 | 1.56 | 2019 |
| Bangladesh | 1.53 | 1.48 | 1.58 | 2020 |
| Bangladesh | 1.54 | 1.47 | 1.6 | 2021 |
| Bangladesh | 1.54 | 1.46 | 1.63 | 2022 |
| Bangladesh | 1.55 | 1.45 | 1.65 | 2023 |
| Bangladesh | 1.55 | 1.43 | 1.67 | 2024 |
| Bangladesh | 1.56 | 1.42 | 1.69 | 2025 |
| Bangladesh | 1.56 | 1.4 | 1.72 | 2026 |
| Bangladesh | 1.56 | 1.38 | 1.74 | 2027 |
| Bangladesh | 1.56 | 1.36 | 1.77 | 2028 |
| Bangladesh | 1.56 | 1.34 | 1.79 | 2029 |
| Bangladesh | 1.57 | 1.31 | 1.82 | 2030 |
| Barbados | 9.8 | 9.1 | 10.51 | 1990 |
| Barbados | 9.48 | 8.89 | 10.07 | 1991 |
| Barbados | 9.17 | 8.67 | 9.68 | 1992 |
| Barbados | 8.87 | 8.43 | 9.31 | 1993 |
| Barbados | 8.57 | 8.18 | 8.97 | 1994 |
| Barbados | 8.29 | 7.93 | 8.66 | 1995 |
| Barbados | 8.03 | 7.69 | 8.37 | 1996 |
| Barbados | 7.78 | 7.45 | 8.1 | 1997 |
| Barbados | 7.55 | 7.23 | 7.86 | 1998 |
| Barbados | 7.34 | 7.04 | 7.65 | 1999 |
| Barbados | 7.17 | 6.87 | 7.46 | 2000 |
| Barbados | 7.01 | 6.72 | 7.31 | 2001 |
| Barbados | 6.89 | 6.6 | 7.17 | 2002 |
| Barbados | 6.78 | 6.5 | 7.07 | 2003 |
| Barbados | 6.7 | 6.42 | 6.98 | 2004 |
| Barbados | 6.64 | 6.37 | 6.92 | 2005 |
| Barbados | 6.6 | 6.33 | 6.87 | 2006 |
| Barbados | 6.57 | 6.3 | 6.84 | 2007 |
| Barbados | 6.55 | 6.29 | 6.82 | 2008 |
| Barbados | 6.54 | 6.28 | 6.81 | 2009 |
| Barbados | 6.54 | 6.27 | 6.81 | 2010 |
| Barbados | 6.54 | 6.27 | 6.81 | 2011 |
| Barbados | 6.54 | 6.26 | 6.82 | 2012 |
| Barbados | 6.54 | 6.25 | 6.83 | 2013 |
| Barbados | 6.54 | 6.23 | 6.86 | 2014 |
| Barbados | 6.54 | 6.19 | 6.89 | 2015 |
| Barbados | 6.53 | 6.14 | 6.93 | 2016 |
| Barbados | 6.52 | 6.07 | 6.98 | 2017 |
| Barbados | 6.51 | 5.98 | 7.04 | 2018 |
| Barbados | 6.5 | 5.88 | 7.11 | 2019 |
| Barbados | 6.48 | 5.77 | 7.19 | 2020 |
| Barbados | 6.46 | 5.65 | 7.28 | 2021 |
| Barbados | 6.44 | 5.51 | 7.36 | 2022 |
| Barbados | 6.41 | 5.37 | 7.45 | 2023 |
| Barbados | 6.38 | 5.22 | 7.54 | 2024 |
| Barbados | 6.35 | 5.06 | 7.63 | 2025 |
| Barbados | 6.31 | 4.9 | 7.72 | 2026 |
| Barbados | 6.27 | 4.72 | 7.81 | 2027 |
| Barbados | 6.22 | 4.54 | 7.9 | 2028 |
| Barbados | 6.17 | 4.36 | 7.98 | 2029 |
| Barbados | 6.12 | 4.17 | 8.06 | 2030 |
| Belarus | 3 | 2.92 | 3.08 | 1990 |
| Belarus | 3.31 | 3.24 | 3.37 | 1991 |
| Belarus | 3.64 | 3.57 | 3.7 | 1992 |
| Belarus | 3.98 | 3.91 | 4.04 | 1993 |
| Belarus | 4.32 | 4.25 | 4.39 | 1994 |
| Belarus | 4.75 | 4.68 | 4.83 | 1995 |
| Belarus | 5.4 | 5.32 | 5.49 | 1996 |
| Belarus | 6.28 | 6.19 | 6.37 | 1997 |
| Belarus | 7.31 | 7.21 | 7.41 | 1998 |
| Belarus | 8.31 | 8.2 | 8.42 | 1999 |
| Belarus | 9.14 | 9.03 | 9.25 | 2000 |
| Belarus | 9.86 | 9.75 | 9.98 | 2001 |
| Belarus | 10.45 | 10.33 | 10.58 | 2002 |
| Belarus | 10.8 | 10.68 | 10.93 | 2003 |
| Belarus | 11.02 | 10.9 | 11.15 | 2004 |
| Belarus | 11.18 | 11.06 | 11.31 | 2005 |
| Belarus | 11.23 | 11.1 | 11.36 | 2006 |
| Belarus | 11.28 | 11.15 | 11.41 | 2007 |
| Belarus | 11.46 | 11.33 | 11.59 | 2008 |
| Belarus | 11.69 | 11.56 | 11.82 | 2009 |
| Belarus | 11.73 | 11.6 | 11.85 | 2010 |
| Belarus | 11.47 | 11.35 | 11.6 | 2011 |
| Belarus | 11 | 10.88 | 11.12 | 2012 |
| Belarus | 10.61 | 10.48 | 10.73 | 2013 |
| Belarus | 10.33 | 10.21 | 10.45 | 2014 |
| Belarus | 10.15 | 10.03 | 10.27 | 2015 |
| Belarus | 10.07 | 9.96 | 10.19 | 2016 |
| Belarus | 10.04 | 9.88 | 10.2 | 2017 |
| Belarus | 10.01 | 9.68 | 10.34 | 2018 |
| Belarus | 9.97 | 9.4 | 10.54 | 2019 |
| Belarus | 9.93 | 9.07 | 10.79 | 2020 |
| Belarus | 9.88 | 8.69 | 11.07 | 2021 |
| Belarus | 9.82 | 8.28 | 11.37 | 2022 |
| Belarus | 9.76 | 7.83 | 11.69 | 2023 |
| Belarus | 9.69 | 7.35 | 12.03 | 2024 |
| Belarus | 9.61 | 6.85 | 12.38 | 2025 |
| Belarus | 9.53 | 6.33 | 12.73 | 2026 |
| Belarus | 9.44 | 5.78 | 13.1 | 2027 |
| Belarus | 9.35 | 5.22 | 13.47 | 2028 |
| Belarus | 9.25 | 4.65 | 13.85 | 2029 |
| Belarus | 9.15 | 4.07 | 14.23 | 2030 |
| Belgium | 7.04 | 6.93 | 7.16 | 1990 |
| Belgium | 7.07 | 6.98 | 7.15 | 1991 |
| Belgium | 7.09 | 7.01 | 7.16 | 1992 |
| Belgium | 7.09 | 7.01 | 7.16 | 1993 |
| Belgium | 7.06 | 6.99 | 7.13 | 1994 |
| Belgium | 7.02 | 6.95 | 7.1 | 1995 |
| Belgium | 7.04 | 6.96 | 7.12 | 1996 |
| Belgium | 7.22 | 7.14 | 7.29 | 1997 |
| Belgium | 7.47 | 7.4 | 7.55 | 1998 |
| Belgium | 7.75 | 7.67 | 7.82 | 1999 |
| Belgium | 7.99 | 7.91 | 8.07 | 2000 |
| Belgium | 8.18 | 8.1 | 8.26 | 2001 |
| Belgium | 8.3 | 8.23 | 8.38 | 2002 |
| Belgium | 8.37 | 8.29 | 8.45 | 2003 |
| Belgium | 8.4 | 8.32 | 8.48 | 2004 |
| Belgium | 8.41 | 8.34 | 8.49 | 2005 |
| Belgium | 8.41 | 8.34 | 8.49 | 2006 |
| Belgium | 8.41 | 8.34 | 8.49 | 2007 |
| Belgium | 8.39 | 8.31 | 8.46 | 2008 |
| Belgium | 8.32 | 8.25 | 8.4 | 2009 |
| Belgium | 8.22 | 8.14 | 8.29 | 2010 |
| Belgium | 8.1 | 8.02 | 8.17 | 2011 |
| Belgium | 7.97 | 7.9 | 8.05 | 2012 |
| Belgium | 7.85 | 7.78 | 7.92 | 2013 |
| Belgium | 7.73 | 7.66 | 7.8 | 2014 |
| Belgium | 7.62 | 7.55 | 7.69 | 2015 |
| Belgium | 7.52 | 7.44 | 7.59 | 2016 |
| Belgium | 7.42 | 7.32 | 7.53 | 2017 |
| Belgium | 7.33 | 7.16 | 7.5 | 2018 |
| Belgium | 7.23 | 6.98 | 7.49 | 2019 |
| Belgium | 7.14 | 6.78 | 7.5 | 2020 |
| Belgium | 7.05 | 6.57 | 7.52 | 2021 |
| Belgium | 6.96 | 6.36 | 7.56 | 2022 |
| Belgium | 6.87 | 6.14 | 7.6 | 2023 |
| Belgium | 6.77 | 5.91 | 7.64 | 2024 |
| Belgium | 6.68 | 5.67 | 7.69 | 2025 |
| Belgium | 6.59 | 5.44 | 7.75 | 2026 |
| Belgium | 6.5 | 5.19 | 7.8 | 2027 |
| Belgium | 6.41 | 4.95 | 7.86 | 2028 |
| Belgium | 6.31 | 4.7 | 7.93 | 2029 |
| Belgium | 6.22 | 4.46 | 7.99 | 2030 |
| Belize | 2.33 | 1.92 | 2.74 | 1990 |
| Belize | 2.53 | 2.14 | 2.92 | 1991 |
| Belize | 2.74 | 2.37 | 3.12 | 1992 |
| Belize | 2.97 | 2.61 | 3.32 | 1993 |
| Belize | 3.2 | 2.86 | 3.55 | 1994 |
| Belize | 3.45 | 3.11 | 3.79 | 1995 |
| Belize | 3.7 | 3.37 | 4.03 | 1996 |
| Belize | 3.95 | 3.62 | 4.28 | 1997 |
| Belize | 4.2 | 3.86 | 4.53 | 1998 |
| Belize | 4.43 | 4.09 | 4.77 | 1999 |
| Belize | 4.65 | 4.31 | 4.99 | 2000 |
| Belize | 4.85 | 4.5 | 5.19 | 2001 |
| Belize | 5.02 | 4.68 | 5.37 | 2002 |
| Belize | 5.18 | 4.84 | 5.52 | 2003 |
| Belize | 5.31 | 4.97 | 5.66 | 2004 |
| Belize | 5.43 | 5.09 | 5.77 | 2005 |
| Belize | 5.52 | 5.18 | 5.86 | 2006 |
| Belize | 5.59 | 5.26 | 5.93 | 2007 |
| Belize | 5.66 | 5.33 | 5.99 | 2008 |
| Belize | 5.71 | 5.38 | 6.04 | 2009 |
| Belize | 5.76 | 5.43 | 6.09 | 2010 |
| Belize | 5.81 | 5.48 | 6.15 | 2011 |
| Belize | 5.87 | 5.53 | 6.21 | 2012 |
| Belize | 5.93 | 5.58 | 6.28 | 2013 |
| Belize | 5.99 | 5.62 | 6.37 | 2014 |
| Belize | 6.06 | 5.65 | 6.48 | 2015 |
| Belize | 6.13 | 5.66 | 6.61 | 2016 |
| Belize | 6.21 | 5.65 | 6.76 | 2017 |
| Belize | 6.28 | 5.62 | 6.94 | 2018 |
| Belize | 6.36 | 5.57 | 7.14 | 2019 |
| Belize | 6.44 | 5.51 | 7.37 | 2020 |
| Belize | 6.52 | 5.43 | 7.61 | 2021 |
| Belize | 6.61 | 5.34 | 7.88 | 2022 |
| Belize | 6.7 | 5.23 | 8.17 | 2023 |
| Belize | 6.8 | 5.12 | 8.49 | 2024 |
| Belize | 6.91 | 4.99 | 8.82 | 2025 |
| Belize | 7.01 | 4.85 | 9.18 | 2026 |
| Belize | 7.13 | 4.7 | 9.56 | 2027 |
| Belize | 7.24 | 4.53 | 9.96 | 2028 |
| Belize | 7.36 | 4.35 | 10.38 | 2029 |
| Belize | 7.49 | 4.16 | 10.82 | 2030 |
| Benin | 1.87 | 1.78 | 1.95 | 1990 |
| Benin | 1.9 | 1.82 | 1.97 | 1991 |
| Benin | 1.93 | 1.86 | 1.99 | 1992 |
| Benin | 1.96 | 1.9 | 2.02 | 1993 |
| Benin | 2 | 1.94 | 2.05 | 1994 |
| Benin | 2.04 | 1.99 | 2.09 | 1995 |
| Benin | 2.08 | 2.03 | 2.13 | 1996 |
| Benin | 2.13 | 2.08 | 2.18 | 1997 |
| Benin | 2.19 | 2.14 | 2.24 | 1998 |
| Benin | 2.25 | 2.2 | 2.3 | 1999 |
| Benin | 2.31 | 2.26 | 2.36 | 2000 |
| Benin | 2.38 | 2.33 | 2.43 | 2001 |
| Benin | 2.45 | 2.4 | 2.5 | 2002 |
| Benin | 2.53 | 2.48 | 2.58 | 2003 |
| Benin | 2.61 | 2.56 | 2.66 | 2004 |
| Benin | 2.69 | 2.64 | 2.74 | 2005 |
| Benin | 2.78 | 2.73 | 2.83 | 2006 |
| Benin | 2.87 | 2.81 | 2.92 | 2007 |
| Benin | 2.95 | 2.9 | 3.01 | 2008 |
| Benin | 3.05 | 2.99 | 3.1 | 2009 |
| Benin | 3.14 | 3.08 | 3.19 | 2010 |
| Benin | 3.23 | 3.17 | 3.29 | 2011 |
| Benin | 3.32 | 3.26 | 3.38 | 2012 |
| Benin | 3.4 | 3.34 | 3.46 | 2013 |
| Benin | 3.47 | 3.41 | 3.53 | 2014 |
| Benin | 3.54 | 3.47 | 3.6 | 2015 |
| Benin | 3.6 | 3.52 | 3.68 | 2016 |
| Benin | 3.66 | 3.56 | 3.75 | 2017 |
| Benin | 3.71 | 3.59 | 3.84 | 2018 |
| Benin | 3.77 | 3.61 | 3.93 | 2019 |
| Benin | 3.83 | 3.63 | 4.04 | 2020 |
| Benin | 3.89 | 3.64 | 4.14 | 2021 |
| Benin | 3.96 | 3.65 | 4.26 | 2022 |
| Benin | 4.02 | 3.66 | 4.38 | 2023 |
| Benin | 4.08 | 3.66 | 4.5 | 2024 |
| Benin | 4.14 | 3.65 | 4.63 | 2025 |
| Benin | 4.21 | 3.64 | 4.77 | 2026 |
| Benin | 4.27 | 3.63 | 4.91 | 2027 |
| Benin | 4.33 | 3.61 | 5.05 | 2028 |
| Benin | 4.39 | 3.59 | 5.2 | 2029 |
| Benin | 4.45 | 3.56 | 5.35 | 2030 |
| Bhutan | 1.28 | 1.09 | 1.47 | 1990 |
| Bhutan | 1.37 | 1.19 | 1.55 | 1991 |
| Bhutan | 1.46 | 1.29 | 1.63 | 1992 |
| Bhutan | 1.54 | 1.38 | 1.7 | 1993 |
| Bhutan | 1.63 | 1.47 | 1.78 | 1994 |
| Bhutan | 1.71 | 1.56 | 1.86 | 1995 |
| Bhutan | 1.79 | 1.65 | 1.94 | 1996 |
| Bhutan | 1.87 | 1.73 | 2.02 | 1997 |
| Bhutan | 1.94 | 1.8 | 2.08 | 1998 |
| Bhutan | 2 | 1.86 | 2.14 | 1999 |
| Bhutan | 2.05 | 1.91 | 2.2 | 2000 |
| Bhutan | 2.1 | 1.96 | 2.24 | 2001 |
| Bhutan | 2.13 | 1.99 | 2.27 | 2002 |
| Bhutan | 2.16 | 2.02 | 2.3 | 2003 |
| Bhutan | 2.18 | 2.04 | 2.31 | 2004 |
| Bhutan | 2.19 | 2.05 | 2.32 | 2005 |
| Bhutan | 2.19 | 2.06 | 2.32 | 2006 |
| Bhutan | 2.19 | 2.06 | 2.32 | 2007 |
| Bhutan | 2.18 | 2.05 | 2.31 | 2008 |
| Bhutan | 2.17 | 2.05 | 2.3 | 2009 |
| Bhutan | 2.17 | 2.04 | 2.3 | 2010 |
| Bhutan | 2.16 | 2.03 | 2.29 | 2011 |
| Bhutan | 2.15 | 2.02 | 2.29 | 2012 |
| Bhutan | 2.15 | 2.01 | 2.29 | 2013 |
| Bhutan | 2.15 | 2 | 2.3 | 2014 |
| Bhutan | 2.15 | 1.99 | 2.31 | 2015 |
| Bhutan | 2.15 | 1.97 | 2.34 | 2016 |
| Bhutan | 2.16 | 1.95 | 2.37 | 2017 |
| Bhutan | 2.18 | 1.94 | 2.42 | 2018 |
| Bhutan | 2.2 | 1.91 | 2.48 | 2019 |
| Bhutan | 2.23 | 1.89 | 2.56 | 2020 |
| Bhutan | 2.26 | 1.87 | 2.65 | 2021 |
| Bhutan | 2.3 | 1.85 | 2.76 | 2022 |
| Bhutan | 2.35 | 1.83 | 2.88 | 2023 |
| Bhutan | 2.41 | 1.8 | 3.02 | 2024 |
| Bhutan | 2.47 | 1.77 | 3.17 | 2025 |
| Bhutan | 2.54 | 1.75 | 3.34 | 2026 |
| Bhutan | 2.62 | 1.71 | 3.53 | 2027 |
| Bhutan | 2.7 | 1.68 | 3.73 | 2028 |
| Bhutan | 2.79 | 1.64 | 3.95 | 2029 |
| Bhutan | 2.88 | 1.59 | 4.18 | 2030 |
| Bolivia | 3.97 | 3.86 | 4.07 | 1990 |
| Bolivia | 4.04 | 3.95 | 4.13 | 1991 |
| Bolivia | 4.13 | 4.05 | 4.2 | 1992 |
| Bolivia | 4.21 | 4.14 | 4.28 | 1993 |
| Bolivia | 4.29 | 4.23 | 4.36 | 1994 |
| Bolivia | 4.38 | 4.31 | 4.44 | 1995 |
| Bolivia | 4.47 | 4.4 | 4.53 | 1996 |
| Bolivia | 4.56 | 4.5 | 4.63 | 1997 |
| Bolivia | 4.66 | 4.6 | 4.72 | 1998 |
| Bolivia | 4.76 | 4.69 | 4.82 | 1999 |
| Bolivia | 4.86 | 4.79 | 4.92 | 2000 |
| Bolivia | 4.96 | 4.89 | 5.02 | 2001 |
| Bolivia | 5.06 | 4.99 | 5.12 | 2002 |
| Bolivia | 5.15 | 5.08 | 5.21 | 2003 |
| Bolivia | 5.23 | 5.17 | 5.3 | 2004 |
| Bolivia | 5.32 | 5.25 | 5.38 | 2005 |
| Bolivia | 5.39 | 5.32 | 5.45 | 2006 |
| Bolivia | 5.45 | 5.39 | 5.52 | 2007 |
| Bolivia | 5.51 | 5.45 | 5.58 | 2008 |
| Bolivia | 5.57 | 5.5 | 5.63 | 2009 |
| Bolivia | 5.62 | 5.55 | 5.68 | 2010 |
| Bolivia | 5.66 | 5.6 | 5.73 | 2011 |
| Bolivia | 5.71 | 5.64 | 5.77 | 2012 |
| Bolivia | 5.75 | 5.68 | 5.81 | 2013 |
| Bolivia | 5.79 | 5.72 | 5.86 | 2014 |
| Bolivia | 5.82 | 5.75 | 5.9 | 2015 |
| Bolivia | 5.86 | 5.77 | 5.95 | 2016 |
| Bolivia | 5.89 | 5.78 | 6 | 2017 |
| Bolivia | 5.92 | 5.78 | 6.07 | 2018 |
| Bolivia | 5.95 | 5.77 | 6.14 | 2019 |
| Bolivia | 5.98 | 5.74 | 6.21 | 2020 |
| Bolivia | 6 | 5.71 | 6.28 | 2021 |
| Bolivia | 6.02 | 5.67 | 6.36 | 2022 |
| Bolivia | 6.03 | 5.63 | 6.44 | 2023 |
| Bolivia | 6.05 | 5.58 | 6.52 | 2024 |
| Bolivia | 6.06 | 5.52 | 6.6 | 2025 |
| Bolivia | 6.07 | 5.45 | 6.68 | 2026 |
| Bolivia | 6.07 | 5.38 | 6.76 | 2027 |
| Bolivia | 6.07 | 5.3 | 6.84 | 2028 |
| Bolivia | 6.07 | 5.22 | 6.92 | 2029 |
| Bolivia | 6.06 | 5.13 | 6.99 | 2030 |
| Bosnia and Herzegovina | 5.82 | 5.67 | 5.96 | 1990 |
| Bosnia and Herzegovina | 5.95 | 5.83 | 6.07 | 1991 |
| Bosnia and Herzegovina | 6.08 | 5.97 | 6.18 | 1992 |
| Bosnia and Herzegovina | 6.2 | 6.1 | 6.3 | 1993 |
| Bosnia and Herzegovina | 6.32 | 6.23 | 6.41 | 1994 |
| Bosnia and Herzegovina | 6.43 | 6.34 | 6.52 | 1995 |
| Bosnia and Herzegovina | 6.54 | 6.45 | 6.63 | 1996 |
| Bosnia and Herzegovina | 6.65 | 6.56 | 6.74 | 1997 |
| Bosnia and Herzegovina | 6.77 | 6.68 | 6.86 | 1998 |
| Bosnia and Herzegovina | 6.89 | 6.8 | 6.99 | 1999 |
| Bosnia and Herzegovina | 7.03 | 6.93 | 7.12 | 2000 |
| Bosnia and Herzegovina | 7.16 | 7.07 | 7.25 | 2001 |
| Bosnia and Herzegovina | 7.3 | 7.2 | 7.39 | 2002 |
| Bosnia and Herzegovina | 7.43 | 7.33 | 7.52 | 2003 |
| Bosnia and Herzegovina | 7.54 | 7.45 | 7.64 | 2004 |
| Bosnia and Herzegovina | 7.65 | 7.55 | 7.74 | 2005 |
| Bosnia and Herzegovina | 7.73 | 7.64 | 7.83 | 2006 |
| Bosnia and Herzegovina | 7.79 | 7.7 | 7.89 | 2007 |
| Bosnia and Herzegovina | 7.83 | 7.73 | 7.93 | 2008 |
| Bosnia and Herzegovina | 7.84 | 7.75 | 7.94 | 2009 |
| Bosnia and Herzegovina | 7.84 | 7.74 | 7.94 | 2010 |
| Bosnia and Herzegovina | 7.83 | 7.73 | 7.92 | 2011 |
| Bosnia and Herzegovina | 7.81 | 7.71 | 7.9 | 2012 |
| Bosnia and Herzegovina | 7.78 | 7.69 | 7.88 | 2013 |
| Bosnia and Herzegovina | 7.76 | 7.66 | 7.85 | 2014 |
| Bosnia and Herzegovina | 7.73 | 7.62 | 7.83 | 2015 |
| Bosnia and Herzegovina | 7.69 | 7.57 | 7.82 | 2016 |
| Bosnia and Herzegovina | 7.66 | 7.5 | 7.82 | 2017 |
| Bosnia and Herzegovina | 7.63 | 7.42 | 7.83 | 2018 |
| Bosnia and Herzegovina | 7.59 | 7.33 | 7.85 | 2019 |
| Bosnia and Herzegovina | 7.56 | 7.23 | 7.88 | 2020 |
| Bosnia and Herzegovina | 7.52 | 7.12 | 7.92 | 2021 |
| Bosnia and Herzegovina | 7.48 | 7.01 | 7.96 | 2022 |
| Bosnia and Herzegovina | 7.44 | 6.88 | 8 | 2023 |
| Bosnia and Herzegovina | 7.4 | 6.76 | 8.04 | 2024 |
| Bosnia and Herzegovina | 7.36 | 6.63 | 8.09 | 2025 |
| Bosnia and Herzegovina | 7.32 | 6.49 | 8.15 | 2026 |
| Bosnia and Herzegovina | 7.28 | 6.35 | 8.2 | 2027 |
| Bosnia and Herzegovina | 7.23 | 6.21 | 8.26 | 2028 |
| Bosnia and Herzegovina | 7.19 | 6.07 | 8.32 | 2029 |
| Bosnia and Herzegovina | 7.15 | 5.92 | 8.38 | 2030 |
| Botswana | 2 | 1.83 | 2.17 | 1990 |
| Botswana | 2.04 | 1.89 | 2.2 | 1991 |
| Botswana | 2.08 | 1.95 | 2.22 | 1992 |
| Botswana | 2.13 | 2 | 2.25 | 1993 |
| Botswana | 2.17 | 2.05 | 2.28 | 1994 |
| Botswana | 2.21 | 2.1 | 2.32 | 1995 |
| Botswana | 2.25 | 2.14 | 2.35 | 1996 |
| Botswana | 2.28 | 2.18 | 2.38 | 1997 |
| Botswana | 2.32 | 2.22 | 2.42 | 1998 |
| Botswana | 2.36 | 2.26 | 2.45 | 1999 |
| Botswana | 2.39 | 2.29 | 2.48 | 2000 |
| Botswana | 2.42 | 2.33 | 2.52 | 2001 |
| Botswana | 2.45 | 2.36 | 2.55 | 2002 |
| Botswana | 2.49 | 2.39 | 2.58 | 2003 |
| Botswana | 2.52 | 2.43 | 2.61 | 2004 |
| Botswana | 2.55 | 2.46 | 2.64 | 2005 |
| Botswana | 2.59 | 2.49 | 2.68 | 2006 |
| Botswana | 2.62 | 2.53 | 2.71 | 2007 |
| Botswana | 2.65 | 2.56 | 2.75 | 2008 |
| Botswana | 2.69 | 2.6 | 2.78 | 2009 |
| Botswana | 2.72 | 2.63 | 2.81 | 2010 |
| Botswana | 2.75 | 2.66 | 2.85 | 2011 |
| Botswana | 2.79 | 2.69 | 2.88 | 2012 |
| Botswana | 2.82 | 2.72 | 2.92 | 2013 |
| Botswana | 2.85 | 2.74 | 2.96 | 2014 |
| Botswana | 2.88 | 2.77 | 3 | 2015 |
| Botswana | 2.92 | 2.78 | 3.05 | 2016 |
| Botswana | 2.95 | 2.79 | 3.11 | 2017 |
| Botswana | 2.98 | 2.8 | 3.17 | 2018 |
| Botswana | 3.02 | 2.8 | 3.23 | 2019 |
| Botswana | 3.05 | 2.8 | 3.31 | 2020 |
| Botswana | 3.09 | 2.8 | 3.39 | 2021 |
| Botswana | 3.13 | 2.79 | 3.47 | 2022 |
| Botswana | 3.17 | 2.78 | 3.56 | 2023 |
| Botswana | 3.22 | 2.77 | 3.66 | 2024 |
| Botswana | 3.26 | 2.76 | 3.76 | 2025 |
| Botswana | 3.31 | 2.74 | 3.87 | 2026 |
| Botswana | 3.36 | 2.72 | 3.99 | 2027 |
| Botswana | 3.41 | 2.7 | 4.11 | 2028 |
| Botswana | 3.46 | 2.68 | 4.23 | 2029 |
| Botswana | 3.51 | 2.66 | 4.37 | 2030 |
| Brazil | 3.95 | 3.92 | 3.98 | 1990 |
| Brazil | 3.98 | 3.96 | 4 | 1991 |
| Brazil | 4.01 | 3.99 | 4.03 | 1992 |
| Brazil | 4.03 | 4.02 | 4.05 | 1993 |
| Brazil | 4.06 | 4.04 | 4.08 | 1994 |
| Brazil | 4.08 | 4.06 | 4.1 | 1995 |
| Brazil | 4.11 | 4.09 | 4.13 | 1996 |
| Brazil | 4.14 | 4.13 | 4.16 | 1997 |
| Brazil | 4.19 | 4.17 | 4.21 | 1998 |
| Brazil | 4.25 | 4.23 | 4.27 | 1999 |
| Brazil | 4.32 | 4.3 | 4.34 | 2000 |
| Brazil | 4.39 | 4.37 | 4.41 | 2001 |
| Brazil | 4.46 | 4.44 | 4.48 | 2002 |
| Brazil | 4.53 | 4.51 | 4.55 | 2003 |
| Brazil | 4.59 | 4.57 | 4.61 | 2004 |
| Brazil | 4.64 | 4.62 | 4.66 | 2005 |
| Brazil | 4.7 | 4.68 | 4.72 | 2006 |
| Brazil | 4.75 | 4.73 | 4.77 | 2007 |
| Brazil | 4.81 | 4.79 | 4.82 | 2008 |
| Brazil | 4.86 | 4.84 | 4.87 | 2009 |
| Brazil | 4.9 | 4.88 | 4.92 | 2010 |
| Brazil | 4.94 | 4.92 | 4.96 | 2011 |
| Brazil | 4.98 | 4.96 | 4.99 | 2012 |
| Brazil | 5.01 | 4.99 | 5.02 | 2013 |
| Brazil | 5.04 | 5.02 | 5.06 | 2014 |
| Brazil | 5.08 | 5.06 | 5.1 | 2015 |
| Brazil | 5.13 | 5.11 | 5.15 | 2016 |
| Brazil | 5.19 | 5.16 | 5.22 | 2017 |
| Brazil | 5.25 | 5.2 | 5.29 | 2018 |
| Brazil | 5.31 | 5.24 | 5.38 | 2019 |
| Brazil | 5.36 | 5.26 | 5.47 | 2020 |
| Brazil | 5.42 | 5.29 | 5.56 | 2021 |
| Brazil | 5.48 | 5.31 | 5.66 | 2022 |
| Brazil | 5.55 | 5.33 | 5.77 | 2023 |
| Brazil | 5.61 | 5.34 | 5.87 | 2024 |
| Brazil | 5.67 | 5.35 | 5.99 | 2025 |
| Brazil | 5.73 | 5.35 | 6.1 | 2026 |
| Brazil | 5.79 | 5.36 | 6.22 | 2027 |
| Brazil | 5.85 | 5.35 | 6.34 | 2028 |
| Brazil | 5.91 | 5.35 | 6.47 | 2029 |
| Brazil | 5.97 | 5.34 | 6.6 | 2030 |
| Bulgaria | 2.83 | 2.74 | 2.91 | 1990 |
| Bulgaria | 2.64 | 2.58 | 2.7 | 1991 |
| Bulgaria | 2.49 | 2.43 | 2.54 | 1992 |
| Bulgaria | 2.36 | 2.31 | 2.41 | 1993 |
| Bulgaria | 2.28 | 2.23 | 2.33 | 1994 |
| Bulgaria | 2.23 | 2.18 | 2.27 | 1995 |
| Bulgaria | 2.2 | 2.16 | 2.25 | 1996 |
| Bulgaria | 2.21 | 2.16 | 2.25 | 1997 |
| Bulgaria | 2.24 | 2.19 | 2.28 | 1998 |
| Bulgaria | 2.3 | 2.25 | 2.35 | 1999 |
| Bulgaria | 2.41 | 2.36 | 2.46 | 2000 |
| Bulgaria | 2.58 | 2.53 | 2.63 | 2001 |
| Bulgaria | 2.83 | 2.77 | 2.88 | 2002 |
| Bulgaria | 3.17 | 3.12 | 3.23 | 2003 |
| Bulgaria | 3.64 | 3.57 | 3.7 | 2004 |
| Bulgaria | 4.19 | 4.12 | 4.26 | 2005 |
| Bulgaria | 4.72 | 4.65 | 4.8 | 2006 |
| Bulgaria | 5.18 | 5.1 | 5.26 | 2007 |
| Bulgaria | 5.54 | 5.45 | 5.62 | 2008 |
| Bulgaria | 5.8 | 5.71 | 5.89 | 2009 |
| Bulgaria | 6 | 5.91 | 6.09 | 2010 |
| Bulgaria | 6.14 | 6.05 | 6.23 | 2011 |
| Bulgaria | 6.22 | 6.13 | 6.31 | 2012 |
| Bulgaria | 6.23 | 6.14 | 6.32 | 2013 |
| Bulgaria | 6.17 | 6.08 | 6.26 | 2014 |
| Bulgaria | 6.01 | 5.92 | 6.1 | 2015 |
| Bulgaria | 5.84 | 5.75 | 5.93 | 2016 |
| Bulgaria | 5.67 | 5.54 | 5.79 | 2017 |
| Bulgaria | 5.5 | 5.27 | 5.72 | 2018 |
| Bulgaria | 5.33 | 4.97 | 5.7 | 2019 |
| Bulgaria | 5.17 | 4.65 | 5.69 | 2020 |
| Bulgaria | 5.01 | 4.32 | 5.71 | 2021 |
| Bulgaria | 4.86 | 3.99 | 5.73 | 2022 |
| Bulgaria | 4.71 | 3.66 | 5.76 | 2023 |
| Bulgaria | 4.56 | 3.33 | 5.8 | 2024 |
| Bulgaria | 4.42 | 3 | 5.84 | 2025 |
| Bulgaria | 4.28 | 2.68 | 5.89 | 2026 |
| Bulgaria | 4.15 | 2.36 | 5.94 | 2027 |
| Bulgaria | 4.01 | 2.05 | 5.98 | 2028 |
| Bulgaria | 3.88 | 1.74 | 6.03 | 2029 |
| Bulgaria | 3.76 | 1.45 | 6.07 | 2030 |
| Burkina Faso | 2.11 | 2.03 | 2.18 | 1990 |
| Burkina Faso | 2.12 | 2.06 | 2.19 | 1991 |
| Burkina Faso | 2.14 | 2.09 | 2.2 | 1992 |
| Burkina Faso | 2.17 | 2.12 | 2.22 | 1993 |
| Burkina Faso | 2.19 | 2.14 | 2.23 | 1994 |
| Burkina Faso | 2.21 | 2.17 | 2.26 | 1995 |
| Burkina Faso | 2.24 | 2.2 | 2.28 | 1996 |
| Burkina Faso | 2.27 | 2.23 | 2.32 | 1997 |
| Burkina Faso | 2.31 | 2.27 | 2.35 | 1998 |
| Burkina Faso | 2.35 | 2.3 | 2.39 | 1999 |
| Burkina Faso | 2.39 | 2.34 | 2.43 | 2000 |
| Burkina Faso | 2.43 | 2.39 | 2.47 | 2001 |
| Burkina Faso | 2.47 | 2.43 | 2.51 | 2002 |
| Burkina Faso | 2.51 | 2.47 | 2.56 | 2003 |
| Burkina Faso | 2.56 | 2.52 | 2.6 | 2004 |
| Burkina Faso | 2.6 | 2.56 | 2.65 | 2005 |
| Burkina Faso | 2.65 | 2.61 | 2.69 | 2006 |
| Burkina Faso | 2.71 | 2.66 | 2.75 | 2007 |
| Burkina Faso | 2.77 | 2.72 | 2.81 | 2008 |
| Burkina Faso | 2.84 | 2.79 | 2.88 | 2009 |
| Burkina Faso | 2.92 | 2.87 | 2.96 | 2010 |
| Burkina Faso | 3.01 | 2.96 | 3.05 | 2011 |
| Burkina Faso | 3.11 | 3.06 | 3.15 | 2012 |
| Burkina Faso | 3.21 | 3.17 | 3.26 | 2013 |
| Burkina Faso | 3.33 | 3.28 | 3.38 | 2014 |
| Burkina Faso | 3.45 | 3.39 | 3.5 | 2015 |
| Burkina Faso | 3.56 | 3.5 | 3.63 | 2016 |
| Burkina Faso | 3.69 | 3.6 | 3.77 | 2017 |
| Burkina Faso | 3.81 | 3.7 | 3.92 | 2018 |
| Burkina Faso | 3.94 | 3.8 | 4.08 | 2019 |
| Burkina Faso | 4.08 | 3.9 | 4.26 | 2020 |
| Burkina Faso | 4.22 | 3.99 | 4.44 | 2021 |
| Burkina Faso | 4.36 | 4.08 | 4.64 | 2022 |
| Burkina Faso | 4.51 | 4.17 | 4.86 | 2023 |
| Burkina Faso | 4.67 | 4.26 | 5.08 | 2024 |
| Burkina Faso | 4.83 | 4.35 | 5.32 | 2025 |
| Burkina Faso | 5 | 4.43 | 5.57 | 2026 |
| Burkina Faso | 5.17 | 4.51 | 5.83 | 2027 |
| Burkina Faso | 5.35 | 4.59 | 6.11 | 2028 |
| Burkina Faso | 5.54 | 4.67 | 6.41 | 2029 |
| Burkina Faso | 5.73 | 4.74 | 6.72 | 2030 |
| Burundi | 2.23 | 2.14 | 2.32 | 1990 |
| Burundi | 2.22 | 2.14 | 2.3 | 1991 |
| Burundi | 2.21 | 2.15 | 2.28 | 1992 |
| Burundi | 2.21 | 2.14 | 2.27 | 1993 |
| Burundi | 2.2 | 2.14 | 2.25 | 1994 |
| Burundi | 2.19 | 2.14 | 2.24 | 1995 |
| Burundi | 2.18 | 2.13 | 2.23 | 1996 |
| Burundi | 2.17 | 2.12 | 2.22 | 1997 |
| Burundi | 2.16 | 2.11 | 2.2 | 1998 |
| Burundi | 2.14 | 2.09 | 2.19 | 1999 |
| Burundi | 2.13 | 2.08 | 2.17 | 2000 |
| Burundi | 2.11 | 2.07 | 2.16 | 2001 |
| Burundi | 2.1 | 2.06 | 2.15 | 2002 |
| Burundi | 2.09 | 2.04 | 2.13 | 2003 |
| Burundi | 2.08 | 2.03 | 2.12 | 2004 |
| Burundi | 2.06 | 2.02 | 2.11 | 2005 |
| Burundi | 2.05 | 2.01 | 2.09 | 2006 |
| Burundi | 2.03 | 1.99 | 2.07 | 2007 |
| Burundi | 2.02 | 1.97 | 2.06 | 2008 |
| Burundi | 2 | 1.96 | 2.04 | 2009 |
| Burundi | 1.98 | 1.94 | 2.02 | 2010 |
| Burundi | 1.96 | 1.92 | 2 | 2011 |
| Burundi | 1.94 | 1.9 | 1.99 | 2012 |
| Burundi | 1.92 | 1.88 | 1.97 | 2013 |
| Burundi | 1.9 | 1.86 | 1.95 | 2014 |
| Burundi | 1.88 | 1.83 | 1.93 | 2015 |
| Burundi | 1.86 | 1.81 | 1.91 | 2016 |
| Burundi | 1.84 | 1.78 | 1.9 | 2017 |
| Burundi | 1.81 | 1.74 | 1.89 | 2018 |
| Burundi | 1.79 | 1.7 | 1.88 | 2019 |
| Burundi | 1.77 | 1.67 | 1.87 | 2020 |
| Burundi | 1.74 | 1.63 | 1.86 | 2021 |
| Burundi | 1.72 | 1.59 | 1.86 | 2022 |
| Burundi | 1.7 | 1.55 | 1.85 | 2023 |
| Burundi | 1.67 | 1.5 | 1.84 | 2024 |
| Burundi | 1.65 | 1.46 | 1.84 | 2025 |
| Burundi | 1.63 | 1.42 | 1.84 | 2026 |
| Burundi | 1.61 | 1.38 | 1.83 | 2027 |
| Burundi | 1.58 | 1.33 | 1.83 | 2028 |
| Burundi | 1.56 | 1.29 | 1.83 | 2029 |
| Burundi | 1.54 | 1.25 | 1.83 | 2030 |
| Cambodia | 3.11 | 3.01 | 3.2 | 1990 |
| Cambodia | 3.11 | 3.03 | 3.19 | 1991 |
| Cambodia | 3.12 | 3.05 | 3.18 | 1992 |
| Cambodia | 3.12 | 3.06 | 3.18 | 1993 |
| Cambodia | 3.13 | 3.07 | 3.18 | 1994 |
| Cambodia | 3.13 | 3.08 | 3.18 | 1995 |
| Cambodia | 3.14 | 3.09 | 3.19 | 1996 |
| Cambodia | 3.15 | 3.1 | 3.2 | 1997 |
| Cambodia | 3.16 | 3.11 | 3.21 | 1998 |
| Cambodia | 3.18 | 3.13 | 3.23 | 1999 |
| Cambodia | 3.19 | 3.15 | 3.24 | 2000 |
| Cambodia | 3.21 | 3.16 | 3.26 | 2001 |
| Cambodia | 3.23 | 3.18 | 3.27 | 2002 |
| Cambodia | 3.24 | 3.19 | 3.29 | 2003 |
| Cambodia | 3.25 | 3.21 | 3.3 | 2004 |
| Cambodia | 3.27 | 3.22 | 3.31 | 2005 |
| Cambodia | 3.28 | 3.23 | 3.32 | 2006 |
| Cambodia | 3.29 | 3.24 | 3.33 | 2007 |
| Cambodia | 3.3 | 3.25 | 3.34 | 2008 |
| Cambodia | 3.31 | 3.27 | 3.36 | 2009 |
| Cambodia | 3.33 | 3.28 | 3.37 | 2010 |
| Cambodia | 3.34 | 3.3 | 3.39 | 2011 |
| Cambodia | 3.36 | 3.32 | 3.4 | 2012 |
| Cambodia | 3.38 | 3.33 | 3.42 | 2013 |
| Cambodia | 3.4 | 3.35 | 3.45 | 2014 |
| Cambodia | 3.42 | 3.37 | 3.47 | 2015 |
| Cambodia | 3.44 | 3.38 | 3.5 | 2016 |
| Cambodia | 3.46 | 3.38 | 3.53 | 2017 |
| Cambodia | 3.48 | 3.38 | 3.57 | 2018 |
| Cambodia | 3.49 | 3.37 | 3.61 | 2019 |
| Cambodia | 3.51 | 3.36 | 3.66 | 2020 |
| Cambodia | 3.53 | 3.35 | 3.7 | 2021 |
| Cambodia | 3.54 | 3.33 | 3.75 | 2022 |
| Cambodia | 3.56 | 3.3 | 3.81 | 2023 |
| Cambodia | 3.57 | 3.28 | 3.86 | 2024 |
| Cambodia | 3.58 | 3.25 | 3.91 | 2025 |
| Cambodia | 3.59 | 3.21 | 3.97 | 2026 |
| Cambodia | 3.6 | 3.18 | 4.03 | 2027 |
| Cambodia | 3.61 | 3.14 | 4.08 | 2028 |
| Cambodia | 3.62 | 3.1 | 4.14 | 2029 |
| Cambodia | 3.63 | 3.05 | 4.2 | 2030 |
| Cameroon | 2.5 | 2.43 | 2.57 | 1990 |
| Cameroon | 2.58 | 2.52 | 2.64 | 1991 |
| Cameroon | 2.67 | 2.62 | 2.72 | 1992 |
| Cameroon | 2.76 | 2.71 | 2.81 | 1993 |
| Cameroon | 2.85 | 2.81 | 2.9 | 1994 |
| Cameroon | 2.95 | 2.91 | 3 | 1995 |
| Cameroon | 3.06 | 3.01 | 3.1 | 1996 |
| Cameroon | 3.17 | 3.13 | 3.22 | 1997 |
| Cameroon | 3.29 | 3.24 | 3.34 | 1998 |
| Cameroon | 3.41 | 3.37 | 3.46 | 1999 |
| Cameroon | 3.54 | 3.49 | 3.58 | 2000 |
| Cameroon | 3.66 | 3.61 | 3.71 | 2001 |
| Cameroon | 3.79 | 3.74 | 3.84 | 2002 |
| Cameroon | 3.91 | 3.86 | 3.96 | 2003 |
| Cameroon | 4.04 | 3.99 | 4.09 | 2004 |
| Cameroon | 4.16 | 4.11 | 4.21 | 2005 |
| Cameroon | 4.29 | 4.24 | 4.34 | 2006 |
| Cameroon | 4.42 | 4.36 | 4.47 | 2007 |
| Cameroon | 4.54 | 4.49 | 4.59 | 2008 |
| Cameroon | 4.66 | 4.61 | 4.71 | 2009 |
| Cameroon | 4.77 | 4.71 | 4.82 | 2010 |
| Cameroon | 4.86 | 4.81 | 4.91 | 2011 |
| Cameroon | 4.94 | 4.88 | 4.99 | 2012 |
| Cameroon | 5 | 4.94 | 5.05 | 2013 |
| Cameroon | 5.05 | 4.99 | 5.11 | 2014 |
| Cameroon | 5.09 | 5.03 | 5.15 | 2015 |
| Cameroon | 5.14 | 5.07 | 5.21 | 2016 |
| Cameroon | 5.17 | 5.08 | 5.26 | 2017 |
| Cameroon | 5.21 | 5.09 | 5.33 | 2018 |
| Cameroon | 5.25 | 5.09 | 5.41 | 2019 |
| Cameroon | 5.28 | 5.08 | 5.49 | 2020 |
| Cameroon | 5.31 | 5.06 | 5.57 | 2021 |
| Cameroon | 5.35 | 5.03 | 5.66 | 2022 |
| Cameroon | 5.38 | 5 | 5.75 | 2023 |
| Cameroon | 5.41 | 4.97 | 5.85 | 2024 |
| Cameroon | 5.44 | 4.93 | 5.95 | 2025 |
| Cameroon | 5.47 | 4.88 | 6.05 | 2026 |
| Cameroon | 5.49 | 4.83 | 6.16 | 2027 |
| Cameroon | 5.52 | 4.77 | 6.26 | 2028 |
| Cameroon | 5.54 | 4.71 | 6.37 | 2029 |
| Cameroon | 5.57 | 4.65 | 6.49 | 2030 |
| Canada | 5.33 | 5.26 | 5.4 | 1990 |
| Canada | 5.4 | 5.35 | 5.45 | 1991 |
| Canada | 5.47 | 5.43 | 5.52 | 1992 |
| Canada | 5.54 | 5.5 | 5.59 | 1993 |
| Canada | 5.61 | 5.56 | 5.65 | 1994 |
| Canada | 5.66 | 5.61 | 5.71 | 1995 |
| Canada | 5.72 | 5.67 | 5.77 | 1996 |
| Canada | 5.8 | 5.75 | 5.84 | 1997 |
| Canada | 5.92 | 5.87 | 5.96 | 1998 |
| Canada | 6.1 | 6.05 | 6.15 | 1999 |
| Canada | 6.34 | 6.29 | 6.39 | 2000 |
| Canada | 6.61 | 6.56 | 6.66 | 2001 |
| Canada | 6.98 | 6.93 | 7.03 | 2002 |
| Canada | 7.43 | 7.38 | 7.48 | 2003 |
| Canada | 7.82 | 7.77 | 7.88 | 2004 |
| Canada | 8.08 | 8.02 | 8.13 | 2005 |
| Canada | 8.16 | 8.1 | 8.21 | 2006 |
| Canada | 8.13 | 8.07 | 8.18 | 2007 |
| Canada | 8.04 | 7.99 | 8.1 | 2008 |
| Canada | 7.92 | 7.86 | 7.97 | 2009 |
| Canada | 7.76 | 7.71 | 7.81 | 2010 |
| Canada | 7.58 | 7.54 | 7.63 | 2011 |
| Canada | 7.46 | 7.41 | 7.51 | 2012 |
| Canada | 7.38 | 7.34 | 7.43 | 2013 |
| Canada | 7.35 | 7.3 | 7.4 | 2014 |
| Canada | 7.32 | 7.27 | 7.37 | 2015 |
| Canada | 7.25 | 7.2 | 7.29 | 2016 |
| Canada | 7.17 | 7.1 | 7.23 | 2017 |
| Canada | 7.09 | 6.96 | 7.22 | 2018 |
| Canada | 7.01 | 6.78 | 7.23 | 2019 |
| Canada | 6.93 | 6.6 | 7.26 | 2020 |
| Canada | 6.85 | 6.4 | 7.3 | 2021 |
| Canada | 6.77 | 6.19 | 7.36 | 2022 |
| Canada | 6.7 | 5.97 | 7.42 | 2023 |
| Canada | 6.62 | 5.74 | 7.5 | 2024 |
| Canada | 6.54 | 5.51 | 7.57 | 2025 |
| Canada | 6.47 | 5.28 | 7.66 | 2026 |
| Canada | 6.39 | 5.04 | 7.75 | 2027 |
| Canada | 6.32 | 4.79 | 7.85 | 2028 |
| Canada | 6.25 | 4.55 | 7.95 | 2029 |
| Canada | 6.18 | 4.3 | 8.05 | 2030 |
| Central African Republic | 1.89 | 1.79 | 1.98 | 1990 |
| Central African Republic | 1.92 | 1.84 | 2.01 | 1991 |
| Central African Republic | 1.96 | 1.88 | 2.03 | 1992 |
| Central African Republic | 2 | 1.93 | 2.06 | 1993 |
| Central African Republic | 2.03 | 1.97 | 2.1 | 1994 |
| Central African Republic | 2.07 | 2.01 | 2.13 | 1995 |
| Central African Republic | 2.11 | 2.05 | 2.17 | 1996 |
| Central African Republic | 2.15 | 2.09 | 2.21 | 1997 |
| Central African Republic | 2.19 | 2.13 | 2.25 | 1998 |
| Central African Republic | 2.23 | 2.17 | 2.29 | 1999 |
| Central African Republic | 2.27 | 2.21 | 2.33 | 2000 |
| Central African Republic | 2.31 | 2.25 | 2.37 | 2001 |
| Central African Republic | 2.35 | 2.29 | 2.41 | 2002 |
| Central African Republic | 2.39 | 2.33 | 2.45 | 2003 |
| Central African Republic | 2.43 | 2.37 | 2.49 | 2004 |
| Central African Republic | 2.47 | 2.41 | 2.53 | 2005 |
| Central African Republic | 2.52 | 2.46 | 2.57 | 2006 |
| Central African Republic | 2.56 | 2.5 | 2.62 | 2007 |
| Central African Republic | 2.6 | 2.54 | 2.66 | 2008 |
| Central African Republic | 2.64 | 2.58 | 2.7 | 2009 |
| Central African Republic | 2.67 | 2.61 | 2.74 | 2010 |
| Central African Republic | 2.71 | 2.64 | 2.77 | 2011 |
| Central African Republic | 2.74 | 2.67 | 2.81 | 2012 |
| Central African Republic | 2.77 | 2.7 | 2.84 | 2013 |
| Central African Republic | 2.8 | 2.72 | 2.87 | 2014 |
| Central African Republic | 2.82 | 2.74 | 2.91 | 2015 |
| Central African Republic | 2.85 | 2.75 | 2.94 | 2016 |
| Central African Republic | 2.87 | 2.76 | 2.98 | 2017 |
| Central African Republic | 2.89 | 2.76 | 3.03 | 2018 |
| Central African Republic | 2.92 | 2.75 | 3.08 | 2019 |
| Central African Republic | 2.94 | 2.75 | 3.13 | 2020 |
| Central African Republic | 2.96 | 2.73 | 3.18 | 2021 |
| Central African Republic | 2.98 | 2.72 | 3.24 | 2022 |
| Central African Republic | 3 | 2.7 | 3.3 | 2023 |
| Central African Republic | 3.01 | 2.67 | 3.35 | 2024 |
| Central African Republic | 3.03 | 2.65 | 3.42 | 2025 |
| Central African Republic | 3.05 | 2.62 | 3.48 | 2026 |
| Central African Republic | 3.06 | 2.58 | 3.54 | 2027 |
| Central African Republic | 3.07 | 2.55 | 3.6 | 2028 |
| Central African Republic | 3.09 | 2.51 | 3.67 | 2029 |
| Central African Republic | 3.1 | 2.46 | 3.73 | 2030 |
| Chad | 1.63 | 1.55 | 1.7 | 1990 |
| Chad | 1.65 | 1.58 | 1.71 | 1991 |
| Chad | 1.67 | 1.61 | 1.73 | 1992 |
| Chad | 1.69 | 1.64 | 1.75 | 1993 |
| Chad | 1.72 | 1.67 | 1.77 | 1994 |
| Chad | 1.75 | 1.7 | 1.8 | 1995 |
| Chad | 1.78 | 1.74 | 1.83 | 1996 |
| Chad | 1.82 | 1.77 | 1.86 | 1997 |
| Chad | 1.86 | 1.81 | 1.9 | 1998 |
| Chad | 1.9 | 1.86 | 1.94 | 1999 |
| Chad | 1.94 | 1.9 | 1.99 | 2000 |
| Chad | 1.99 | 1.95 | 2.03 | 2001 |
| Chad | 2.04 | 2 | 2.08 | 2002 |
| Chad | 2.09 | 2.05 | 2.13 | 2003 |
| Chad | 2.14 | 2.1 | 2.18 | 2004 |
| Chad | 2.19 | 2.15 | 2.23 | 2005 |
| Chad | 2.24 | 2.2 | 2.28 | 2006 |
| Chad | 2.29 | 2.25 | 2.33 | 2007 |
| Chad | 2.34 | 2.3 | 2.38 | 2008 |
| Chad | 2.39 | 2.34 | 2.43 | 2009 |
| Chad | 2.43 | 2.39 | 2.48 | 2010 |
| Chad | 2.48 | 2.43 | 2.52 | 2011 |
| Chad | 2.52 | 2.47 | 2.56 | 2012 |
| Chad | 2.56 | 2.51 | 2.61 | 2013 |
| Chad | 2.6 | 2.55 | 2.65 | 2014 |
| Chad | 2.64 | 2.59 | 2.7 | 2015 |
| Chad | 2.68 | 2.62 | 2.75 | 2016 |
| Chad | 2.73 | 2.65 | 2.8 | 2017 |
| Chad | 2.77 | 2.67 | 2.86 | 2018 |
| Chad | 2.81 | 2.7 | 2.93 | 2019 |
| Chad | 2.86 | 2.71 | 3 | 2020 |
| Chad | 2.9 | 2.73 | 3.08 | 2021 |
| Chad | 2.95 | 2.74 | 3.16 | 2022 |
| Chad | 2.99 | 2.75 | 3.24 | 2023 |
| Chad | 3.04 | 2.75 | 3.33 | 2024 |
| Chad | 3.09 | 2.76 | 3.42 | 2025 |
| Chad | 3.14 | 2.76 | 3.52 | 2026 |
| Chad | 3.19 | 2.75 | 3.62 | 2027 |
| Chad | 3.24 | 2.75 | 3.72 | 2028 |
| Chad | 3.29 | 2.74 | 3.83 | 2029 |
| Chad | 3.34 | 2.73 | 3.95 | 2030 |
| Chile | 8.82 | 8.67 | 8.96 | 1990 |
| Chile | 8.62 | 8.52 | 8.73 | 1991 |
| Chile | 8.46 | 8.38 | 8.55 | 1992 |
| Chile | 8.36 | 8.28 | 8.44 | 1993 |
| Chile | 8.31 | 8.23 | 8.39 | 1994 |
| Chile | 8.31 | 8.23 | 8.39 | 1995 |
| Chile | 8.35 | 8.27 | 8.42 | 1996 |
| Chile | 8.41 | 8.33 | 8.48 | 1997 |
| Chile | 8.49 | 8.42 | 8.56 | 1998 |
| Chile | 8.57 | 8.5 | 8.65 | 1999 |
| Chile | 8.67 | 8.59 | 8.74 | 2000 |
| Chile | 8.77 | 8.7 | 8.84 | 2001 |
| Chile | 8.88 | 8.8 | 8.95 | 2002 |
| Chile | 8.99 | 8.92 | 9.06 | 2003 |
| Chile | 9.1 | 9.03 | 9.17 | 2004 |
| Chile | 9.2 | 9.13 | 9.27 | 2005 |
| Chile | 9.29 | 9.22 | 9.36 | 2006 |
| Chile | 9.38 | 9.31 | 9.45 | 2007 |
| Chile | 9.47 | 9.4 | 9.54 | 2008 |
| Chile | 9.57 | 9.5 | 9.64 | 2009 |
| Chile | 9.66 | 9.59 | 9.73 | 2010 |
| Chile | 9.74 | 9.67 | 9.81 | 2011 |
| Chile | 9.81 | 9.74 | 9.88 | 2012 |
| Chile | 9.88 | 9.81 | 9.95 | 2013 |
| Chile | 9.94 | 9.87 | 10.01 | 2014 |
| Chile | 9.99 | 9.92 | 10.07 | 2015 |
| Chile | 10.04 | 9.96 | 10.12 | 2016 |
| Chile | 10.09 | 9.98 | 10.2 | 2017 |
| Chile | 10.14 | 9.97 | 10.3 | 2018 |
| Chile | 10.18 | 9.94 | 10.43 | 2019 |
| Chile | 10.23 | 9.89 | 10.57 | 2020 |
| Chile | 10.27 | 9.83 | 10.71 | 2021 |
| Chile | 10.31 | 9.75 | 10.87 | 2022 |
| Chile | 10.35 | 9.66 | 11.04 | 2023 |
| Chile | 10.39 | 9.56 | 11.21 | 2024 |
| Chile | 10.43 | 9.46 | 11.4 | 2025 |
| Chile | 10.46 | 9.34 | 11.59 | 2026 |
| Chile | 10.49 | 9.21 | 11.78 | 2027 |
| Chile | 10.52 | 9.07 | 11.98 | 2028 |
| Chile | 10.55 | 8.92 | 12.18 | 2029 |
| Chile | 10.58 | 8.76 | 12.39 | 2030 |
| China | 2.29 | 2.28 | 2.3 | 1990 |
| China | 2.31 | 2.3 | 2.31 | 1991 |
| China | 2.3 | 2.29 | 2.3 | 1992 |
| China | 2.25 | 2.25 | 2.26 | 1993 |
| China | 2.19 | 2.18 | 2.19 | 1994 |
| China | 2.15 | 2.14 | 2.15 | 1995 |
| China | 2.13 | 2.12 | 2.14 | 1996 |
| China | 2.12 | 2.11 | 2.13 | 1997 |
| China | 2.14 | 2.13 | 2.14 | 1998 |
| China | 2.19 | 2.19 | 2.2 | 1999 |
| China | 2.28 | 2.27 | 2.28 | 2000 |
| China | 2.36 | 2.36 | 2.37 | 2001 |
| China | 2.42 | 2.41 | 2.42 | 2002 |
| China | 2.45 | 2.44 | 2.46 | 2003 |
| China | 2.49 | 2.48 | 2.5 | 2004 |
| China | 2.5 | 2.5 | 2.51 | 2005 |
| China | 2.51 | 2.5 | 2.52 | 2006 |
| China | 2.53 | 2.52 | 2.53 | 2007 |
| China | 2.57 | 2.56 | 2.58 | 2008 |
| China | 2.63 | 2.62 | 2.63 | 2009 |
| China | 2.67 | 2.66 | 2.68 | 2010 |
| China | 2.71 | 2.7 | 2.71 | 2011 |
| China | 2.73 | 2.73 | 2.74 | 2012 |
| China | 2.75 | 2.75 | 2.76 | 2013 |
| China | 2.79 | 2.78 | 2.8 | 2014 |
| China | 2.82 | 2.82 | 2.83 | 2015 |
| China | 2.82 | 2.81 | 2.83 | 2016 |
| China | 2.76 | 2.75 | 2.77 | 2017 |
| China | 2.7 | 2.67 | 2.73 | 2018 |
| China | 2.64 | 2.58 | 2.7 | 2019 |
| China | 2.58 | 2.49 | 2.68 | 2020 |
| China | 2.53 | 2.4 | 2.66 | 2021 |
| China | 2.48 | 2.31 | 2.65 | 2022 |
| China | 2.42 | 2.21 | 2.63 | 2023 |
| China | 2.36 | 2.11 | 2.62 | 2024 |
| China | 2.32 | 2.02 | 2.62 | 2025 |
| China | 2.27 | 1.93 | 2.62 | 2026 |
| China | 2.22 | 1.83 | 2.61 | 2027 |
| China | 2.17 | 1.73 | 2.6 | 2028 |
| China | 2.12 | 1.64 | 2.6 | 2029 |
| China | 2.07 | 1.54 | 2.6 | 2030 |
| Colombia | 2.78 | 2.73 | 2.82 | 1990 |
| Colombia | 2.82 | 2.78 | 2.86 | 1991 |
| Colombia | 2.87 | 2.83 | 2.9 | 1992 |
| Colombia | 2.91 | 2.88 | 2.94 | 1993 |
| Colombia | 2.95 | 2.92 | 2.98 | 1994 |
| Colombia | 2.99 | 2.96 | 3.02 | 1995 |
| Colombia | 3.03 | 3 | 3.06 | 1996 |
| Colombia | 3.07 | 3.05 | 3.1 | 1997 |
| Colombia | 3.12 | 3.09 | 3.15 | 1998 |
| Colombia | 3.17 | 3.15 | 3.2 | 1999 |
| Colombia | 3.24 | 3.21 | 3.26 | 2000 |
| Colombia | 3.31 | 3.28 | 3.34 | 2001 |
| Colombia | 3.38 | 3.36 | 3.41 | 2002 |
| Colombia | 3.45 | 3.43 | 3.48 | 2003 |
| Colombia | 3.52 | 3.49 | 3.54 | 2004 |
| Colombia | 3.57 | 3.54 | 3.6 | 2005 |
| Colombia | 3.61 | 3.59 | 3.64 | 2006 |
| Colombia | 3.65 | 3.63 | 3.68 | 2007 |
| Colombia | 3.69 | 3.66 | 3.72 | 2008 |
| Colombia | 3.72 | 3.69 | 3.75 | 2009 |
| Colombia | 3.74 | 3.71 | 3.77 | 2010 |
| Colombia | 3.76 | 3.73 | 3.78 | 2011 |
| Colombia | 3.77 | 3.74 | 3.8 | 2012 |
| Colombia | 3.78 | 3.75 | 3.81 | 2013 |
| Colombia | 3.79 | 3.76 | 3.82 | 2014 |
| Colombia | 3.8 | 3.78 | 3.83 | 2015 |
| Colombia | 3.82 | 3.79 | 3.85 | 2016 |
| Colombia | 3.83 | 3.79 | 3.88 | 2017 |
| Colombia | 3.85 | 3.79 | 3.91 | 2018 |
| Colombia | 3.86 | 3.78 | 3.95 | 2019 |
| Colombia | 3.88 | 3.76 | 3.99 | 2020 |
| Colombia | 3.89 | 3.75 | 4.04 | 2021 |
| Colombia | 3.91 | 3.72 | 4.09 | 2022 |
| Colombia | 3.92 | 3.7 | 4.14 | 2023 |
| Colombia | 3.94 | 3.67 | 4.2 | 2024 |
| Colombia | 3.95 | 3.65 | 4.26 | 2025 |
| Colombia | 3.97 | 3.62 | 4.32 | 2026 |
| Colombia | 3.98 | 3.58 | 4.39 | 2027 |
| Colombia | 4 | 3.55 | 4.45 | 2028 |
| Colombia | 4.02 | 3.51 | 4.53 | 2029 |
| Colombia | 4.04 | 3.48 | 4.6 | 2030 |
| Comoros | 1.64 | 1.41 | 1.86 | 1990 |
| Comoros | 1.7 | 1.49 | 1.92 | 1991 |
| Comoros | 1.77 | 1.58 | 1.97 | 1992 |
| Comoros | 1.84 | 1.65 | 2.03 | 1993 |
| Comoros | 1.91 | 1.73 | 2.09 | 1994 |
| Comoros | 1.97 | 1.8 | 2.15 | 1995 |
| Comoros | 2.04 | 1.87 | 2.2 | 1996 |
| Comoros | 2.1 | 1.94 | 2.25 | 1997 |
| Comoros | 2.15 | 2 | 2.31 | 1998 |
| Comoros | 2.2 | 2.05 | 2.36 | 1999 |
| Comoros | 2.26 | 2.11 | 2.41 | 2000 |
| Comoros | 2.3 | 2.15 | 2.45 | 2001 |
| Comoros | 2.35 | 2.2 | 2.49 | 2002 |
| Comoros | 2.38 | 2.24 | 2.53 | 2003 |
| Comoros | 2.42 | 2.27 | 2.56 | 2004 |
| Comoros | 2.45 | 2.3 | 2.59 | 2005 |
| Comoros | 2.47 | 2.33 | 2.62 | 2006 |
| Comoros | 2.49 | 2.35 | 2.64 | 2007 |
| Comoros | 2.51 | 2.36 | 2.66 | 2008 |
| Comoros | 2.52 | 2.37 | 2.67 | 2009 |
| Comoros | 2.52 | 2.37 | 2.68 | 2010 |
| Comoros | 2.53 | 2.37 | 2.68 | 2011 |
| Comoros | 2.52 | 2.36 | 2.69 | 2012 |
| Comoros | 2.52 | 2.35 | 2.69 | 2013 |
| Comoros | 2.51 | 2.33 | 2.69 | 2014 |
| Comoros | 2.51 | 2.31 | 2.7 | 2015 |
| Comoros | 2.5 | 2.28 | 2.72 | 2016 |
| Comoros | 2.49 | 2.25 | 2.74 | 2017 |
| Comoros | 2.49 | 2.22 | 2.76 | 2018 |
| Comoros | 2.49 | 2.18 | 2.8 | 2019 |
| Comoros | 2.49 | 2.14 | 2.83 | 2020 |
| Comoros | 2.49 | 2.1 | 2.88 | 2021 |
| Comoros | 2.49 | 2.05 | 2.93 | 2022 |
| Comoros | 2.49 | 2.01 | 2.98 | 2023 |
| Comoros | 2.5 | 1.96 | 3.04 | 2024 |
| Comoros | 2.51 | 1.91 | 3.1 | 2025 |
| Comoros | 2.52 | 1.86 | 3.17 | 2026 |
| Comoros | 2.53 | 1.81 | 3.24 | 2027 |
| Comoros | 2.54 | 1.76 | 3.32 | 2028 |
| Comoros | 2.55 | 1.71 | 3.4 | 2029 |
| Comoros | 2.56 | 1.65 | 3.48 | 2030 |
| Congo | 2.97 | 2.82 | 3.11 | 1990 |
| Congo | 3.01 | 2.89 | 3.14 | 1991 |
| Congo | 3.06 | 2.95 | 3.17 | 1992 |
| Congo | 3.11 | 3.01 | 3.21 | 1993 |
| Congo | 3.15 | 3.06 | 3.25 | 1994 |
| Congo | 3.2 | 3.11 | 3.29 | 1995 |
| Congo | 3.24 | 3.15 | 3.32 | 1996 |
| Congo | 3.28 | 3.19 | 3.36 | 1997 |
| Congo | 3.31 | 3.23 | 3.39 | 1998 |
| Congo | 3.34 | 3.25 | 3.42 | 1999 |
| Congo | 3.36 | 3.28 | 3.44 | 2000 |
| Congo | 3.38 | 3.3 | 3.46 | 2001 |
| Congo | 3.4 | 3.32 | 3.48 | 2002 |
| Congo | 3.42 | 3.34 | 3.5 | 2003 |
| Congo | 3.44 | 3.36 | 3.52 | 2004 |
| Congo | 3.46 | 3.38 | 3.54 | 2005 |
| Congo | 3.48 | 3.4 | 3.56 | 2006 |
| Congo | 3.5 | 3.43 | 3.58 | 2007 |
| Congo | 3.53 | 3.45 | 3.6 | 2008 |
| Congo | 3.55 | 3.47 | 3.62 | 2009 |
| Congo | 3.57 | 3.49 | 3.65 | 2010 |
| Congo | 3.6 | 3.52 | 3.68 | 2011 |
| Congo | 3.63 | 3.55 | 3.71 | 2012 |
| Congo | 3.66 | 3.57 | 3.74 | 2013 |
| Congo | 3.69 | 3.6 | 3.77 | 2014 |
| Congo | 3.72 | 3.62 | 3.81 | 2015 |
| Congo | 3.75 | 3.64 | 3.86 | 2016 |
| Congo | 3.78 | 3.64 | 3.91 | 2017 |
| Congo | 3.81 | 3.65 | 3.97 | 2018 |
| Congo | 3.83 | 3.64 | 4.03 | 2019 |
| Congo | 3.86 | 3.63 | 4.1 | 2020 |
| Congo | 3.89 | 3.62 | 4.17 | 2021 |
| Congo | 3.92 | 3.6 | 4.24 | 2022 |
| Congo | 3.95 | 3.57 | 4.32 | 2023 |
| Congo | 3.97 | 3.55 | 4.4 | 2024 |
| Congo | 4 | 3.51 | 4.49 | 2025 |
| Congo | 4.02 | 3.48 | 4.57 | 2026 |
| Congo | 4.05 | 3.44 | 4.66 | 2027 |
| Congo | 4.07 | 3.39 | 4.75 | 2028 |
| Congo | 4.09 | 3.35 | 4.84 | 2029 |
| Congo | 4.11 | 3.3 | 4.93 | 2030 |
| Costa Rica | 2.59 | 2.46 | 2.71 | 1990 |
| Costa Rica | 2.61 | 2.51 | 2.71 | 1991 |
| Costa Rica | 2.64 | 2.55 | 2.73 | 1992 |
| Costa Rica | 2.66 | 2.59 | 2.74 | 1993 |
| Costa Rica | 2.69 | 2.62 | 2.77 | 1994 |
| Costa Rica | 2.73 | 2.66 | 2.8 | 1995 |
| Costa Rica | 2.76 | 2.69 | 2.83 | 1996 |
| Costa Rica | 2.8 | 2.74 | 2.87 | 1997 |
| Costa Rica | 2.85 | 2.78 | 2.92 | 1998 |
| Costa Rica | 2.91 | 2.84 | 2.97 | 1999 |
| Costa Rica | 2.98 | 2.91 | 3.04 | 2000 |
| Costa Rica | 3.05 | 2.99 | 3.12 | 2001 |
| Costa Rica | 3.14 | 3.08 | 3.21 | 2002 |
| Costa Rica | 3.24 | 3.17 | 3.3 | 2003 |
| Costa Rica | 3.34 | 3.27 | 3.4 | 2004 |
| Costa Rica | 3.44 | 3.37 | 3.51 | 2005 |
| Costa Rica | 3.55 | 3.48 | 3.62 | 2006 |
| Costa Rica | 3.66 | 3.59 | 3.72 | 2007 |
| Costa Rica | 3.77 | 3.7 | 3.83 | 2008 |
| Costa Rica | 3.88 | 3.81 | 3.95 | 2009 |
| Costa Rica | 3.99 | 3.92 | 4.06 | 2010 |
| Costa Rica | 4.1 | 4.03 | 4.17 | 2011 |
| Costa Rica | 4.2 | 4.13 | 4.27 | 2012 |
| Costa Rica | 4.29 | 4.22 | 4.36 | 2013 |
| Costa Rica | 4.38 | 4.31 | 4.46 | 2014 |
| Costa Rica | 4.47 | 4.38 | 4.55 | 2015 |
| Costa Rica | 4.55 | 4.45 | 4.65 | 2016 |
| Costa Rica | 4.64 | 4.51 | 4.76 | 2017 |
| Costa Rica | 4.73 | 4.57 | 4.89 | 2018 |
| Costa Rica | 4.82 | 4.61 | 5.03 | 2019 |
| Costa Rica | 4.91 | 4.64 | 5.18 | 2020 |
| Costa Rica | 5.01 | 4.68 | 5.34 | 2021 |
| Costa Rica | 5.1 | 4.7 | 5.5 | 2022 |
| Costa Rica | 5.2 | 4.72 | 5.68 | 2023 |
| Costa Rica | 5.3 | 4.74 | 5.87 | 2024 |
| Costa Rica | 5.4 | 4.75 | 6.06 | 2025 |
| Costa Rica | 5.51 | 4.75 | 6.27 | 2026 |
| Costa Rica | 5.62 | 4.75 | 6.48 | 2027 |
| Costa Rica | 5.72 | 4.74 | 6.71 | 2028 |
| Costa Rica | 5.83 | 4.73 | 6.94 | 2029 |
| Costa Rica | 5.95 | 4.71 | 7.18 | 2030 |
| Cote d'Ivoire | 1.23 | 1.18 | 1.28 | 1990 |
| Cote d'Ivoire | 1.28 | 1.23 | 1.32 | 1991 |
| Cote d'Ivoire | 1.32 | 1.28 | 1.36 | 1992 |
| Cote d'Ivoire | 1.37 | 1.33 | 1.4 | 1993 |
| Cote d'Ivoire | 1.41 | 1.38 | 1.45 | 1994 |
| Cote d'Ivoire | 1.46 | 1.43 | 1.49 | 1995 |
| Cote d'Ivoire | 1.51 | 1.48 | 1.54 | 1996 |
| Cote d'Ivoire | 1.56 | 1.53 | 1.59 | 1997 |
| Cote d'Ivoire | 1.61 | 1.58 | 1.64 | 1998 |
| Cote d'Ivoire | 1.65 | 1.62 | 1.68 | 1999 |
| Cote d'Ivoire | 1.7 | 1.67 | 1.73 | 2000 |
| Cote d'Ivoire | 1.74 | 1.71 | 1.77 | 2001 |
| Cote d'Ivoire | 1.78 | 1.75 | 1.81 | 2002 |
| Cote d'Ivoire | 1.82 | 1.79 | 1.85 | 2003 |
| Cote d'Ivoire | 1.86 | 1.83 | 1.89 | 2004 |
| Cote d'Ivoire | 1.89 | 1.86 | 1.92 | 2005 |
| Cote d'Ivoire | 1.92 | 1.89 | 1.95 | 2006 |
| Cote d'Ivoire | 1.95 | 1.92 | 1.98 | 2007 |
| Cote d'Ivoire | 1.98 | 1.95 | 2.01 | 2008 |
| Cote d'Ivoire | 2.01 | 1.98 | 2.04 | 2009 |
| Cote d'Ivoire | 2.04 | 2.01 | 2.07 | 2010 |
| Cote d'Ivoire | 2.07 | 2.04 | 2.1 | 2011 |
| Cote d'Ivoire | 2.1 | 2.07 | 2.14 | 2012 |
| Cote d'Ivoire | 2.14 | 2.1 | 2.17 | 2013 |
| Cote d'Ivoire | 2.17 | 2.13 | 2.2 | 2014 |
| Cote d'Ivoire | 2.2 | 2.16 | 2.24 | 2015 |
| Cote d'Ivoire | 2.23 | 2.19 | 2.28 | 2016 |
| Cote d'Ivoire | 2.26 | 2.21 | 2.32 | 2017 |
| Cote d'Ivoire | 2.29 | 2.22 | 2.36 | 2018 |
| Cote d'Ivoire | 2.32 | 2.23 | 2.41 | 2019 |
| Cote d'Ivoire | 2.35 | 2.24 | 2.47 | 2020 |
| Cote d'Ivoire | 2.38 | 2.25 | 2.52 | 2021 |
| Cote d'Ivoire | 2.41 | 2.25 | 2.58 | 2022 |
| Cote d'Ivoire | 2.45 | 2.25 | 2.64 | 2023 |
| Cote d'Ivoire | 2.48 | 2.25 | 2.71 | 2024 |
| Cote d'Ivoire | 2.51 | 2.24 | 2.77 | 2025 |
| Cote d'Ivoire | 2.54 | 2.23 | 2.84 | 2026 |
| Cote d'Ivoire | 2.57 | 2.22 | 2.91 | 2027 |
| Cote d'Ivoire | 2.6 | 2.21 | 2.98 | 2028 |
| Cote d'Ivoire | 2.63 | 2.19 | 3.06 | 2029 |
| Cote d'Ivoire | 2.65 | 2.17 | 3.14 | 2030 |
| Croatia | 4.88 | 4.73 | 5.03 | 1990 |
| Croatia | 5.19 | 5.08 | 5.3 | 1991 |
| Croatia | 5.54 | 5.44 | 5.65 | 1992 |
| Croatia | 6.01 | 5.89 | 6.12 | 1993 |
| Croatia | 6.64 | 6.52 | 6.76 | 1994 |
| Croatia | 7.47 | 7.34 | 7.59 | 1995 |
| Croatia | 8.42 | 8.29 | 8.56 | 1996 |
| Croatia | 9.31 | 9.16 | 9.46 | 1997 |
| Croatia | 9.92 | 9.77 | 10.08 | 1998 |
| Croatia | 10.18 | 10.03 | 10.34 | 1999 |
| Croatia | 10.15 | 9.99 | 10.3 | 2000 |
| Croatia | 10.07 | 9.92 | 10.22 | 2001 |
| Croatia | 10.1 | 9.95 | 10.25 | 2002 |
| Croatia | 10.27 | 10.12 | 10.43 | 2003 |
| Croatia | 10.56 | 10.41 | 10.72 | 2004 |
| Croatia | 10.95 | 10.79 | 11.11 | 2005 |
| Croatia | 11.32 | 11.16 | 11.47 | 2006 |
| Croatia | 11.77 | 11.6 | 11.93 | 2007 |
| Croatia | 12.25 | 12.09 | 12.42 | 2008 |
| Croatia | 12.6 | 12.43 | 12.77 | 2009 |
| Croatia | 12.94 | 12.76 | 13.11 | 2010 |
| Croatia | 13.32 | 13.14 | 13.49 | 2011 |
| Croatia | 13.67 | 13.49 | 13.84 | 2012 |
| Croatia | 13.92 | 13.74 | 14.1 | 2013 |
| Croatia | 14.09 | 13.9 | 14.28 | 2014 |
| Croatia | 13.98 | 13.79 | 14.16 | 2015 |
| Croatia | 13.48 | 13.29 | 13.66 | 2016 |
| Croatia | 12.83 | 12.59 | 13.08 | 2017 |
| Croatia | 12.22 | 11.76 | 12.68 | 2018 |
| Croatia | 11.63 | 10.88 | 12.38 | 2019 |
| Croatia | 11.07 | 10 | 12.14 | 2020 |
| Croatia | 10.53 | 9.13 | 11.92 | 2021 |
| Croatia | 10.01 | 8.28 | 11.74 | 2022 |
| Croatia | 9.51 | 7.45 | 11.57 | 2023 |
| Croatia | 9.03 | 6.65 | 11.41 | 2024 |
| Croatia | 8.57 | 5.89 | 11.26 | 2025 |
| Croatia | 8.13 | 5.15 | 11.1 | 2026 |
| Croatia | 7.7 | 4.46 | 10.94 | 2027 |
| Croatia | 7.29 | 3.8 | 10.79 | 2028 |
| Croatia | 6.91 | 3.18 | 10.63 | 2029 |
| Croatia | 6.54 | 2.61 | 10.48 | 2030 |
| Cuba | 7.14 | 7 | 7.28 | 1990 |
| Cuba | 7.03 | 6.94 | 7.13 | 1991 |
| Cuba | 6.91 | 6.82 | 7 | 1992 |
| Cuba | 6.75 | 6.66 | 6.83 | 1993 |
| Cuba | 6.52 | 6.44 | 6.61 | 1994 |
| Cuba | 6.24 | 6.16 | 6.32 | 1995 |
| Cuba | 5.92 | 5.84 | 5.99 | 1996 |
| Cuba | 5.6 | 5.53 | 5.67 | 1997 |
| Cuba | 5.32 | 5.26 | 5.39 | 1998 |
| Cuba | 5.11 | 5.05 | 5.18 | 1999 |
| Cuba | 4.97 | 4.9 | 5.03 | 2000 |
| Cuba | 4.88 | 4.81 | 4.94 | 2001 |
| Cuba | 4.84 | 4.78 | 4.9 | 2002 |
| Cuba | 4.84 | 4.78 | 4.9 | 2003 |
| Cuba | 4.86 | 4.8 | 4.92 | 2004 |
| Cuba | 4.87 | 4.81 | 4.94 | 2005 |
| Cuba | 4.88 | 4.82 | 4.94 | 2006 |
| Cuba | 4.88 | 4.82 | 4.94 | 2007 |
| Cuba | 4.89 | 4.83 | 4.95 | 2008 |
| Cuba | 4.89 | 4.83 | 4.95 | 2009 |
| Cuba | 4.9 | 4.84 | 4.96 | 2010 |
| Cuba | 4.91 | 4.85 | 4.97 | 2011 |
| Cuba | 4.93 | 4.87 | 4.98 | 2012 |
| Cuba | 4.95 | 4.9 | 5.01 | 2013 |
| Cuba | 4.99 | 4.93 | 5.05 | 2014 |
| Cuba | 5.04 | 4.98 | 5.1 | 2015 |
| Cuba | 5.08 | 5.01 | 5.15 | 2016 |
| Cuba | 5.11 | 5.02 | 5.21 | 2017 |
| Cuba | 5.14 | 5 | 5.29 | 2018 |
| Cuba | 5.17 | 4.96 | 5.39 | 2019 |
| Cuba | 5.2 | 4.9 | 5.5 | 2020 |
| Cuba | 5.23 | 4.83 | 5.63 | 2021 |
| Cuba | 5.25 | 4.74 | 5.76 | 2022 |
| Cuba | 5.27 | 4.65 | 5.89 | 2023 |
| Cuba | 5.29 | 4.54 | 6.04 | 2024 |
| Cuba | 5.31 | 4.43 | 6.19 | 2025 |
| Cuba | 5.32 | 4.3 | 6.35 | 2026 |
| Cuba | 5.34 | 4.17 | 6.51 | 2027 |
| Cuba | 5.35 | 4.02 | 6.68 | 2028 |
| Cuba | 5.36 | 3.87 | 6.85 | 2029 |
| Cuba | 5.37 | 3.72 | 7.03 | 2030 |
| Cyprus | 1.87 | 1.72 | 2.02 | 1990 |
| Cyprus | 1.94 | 1.81 | 2.07 | 1991 |
| Cyprus | 2.01 | 1.89 | 2.13 | 1992 |
| Cyprus | 2.08 | 1.97 | 2.19 | 1993 |
| Cyprus | 2.16 | 2.06 | 2.26 | 1994 |
| Cyprus | 2.24 | 2.14 | 2.34 | 1995 |
| Cyprus | 2.33 | 2.24 | 2.43 | 1996 |
| Cyprus | 2.43 | 2.33 | 2.53 | 1997 |
| Cyprus | 2.54 | 2.44 | 2.63 | 1998 |
| Cyprus | 2.65 | 2.55 | 2.75 | 1999 |
| Cyprus | 2.77 | 2.67 | 2.87 | 2000 |
| Cyprus | 2.91 | 2.8 | 3.01 | 2001 |
| Cyprus | 3.04 | 2.94 | 3.15 | 2002 |
| Cyprus | 3.19 | 3.08 | 3.29 | 2003 |
| Cyprus | 3.34 | 3.23 | 3.44 | 2004 |
| Cyprus | 3.48 | 3.37 | 3.6 | 2005 |
| Cyprus | 3.63 | 3.52 | 3.74 | 2006 |
| Cyprus | 3.77 | 3.66 | 3.89 | 2007 |
| Cyprus | 3.91 | 3.79 | 4.03 | 2008 |
| Cyprus | 4.04 | 3.92 | 4.16 | 2009 |
| Cyprus | 4.16 | 4.04 | 4.28 | 2010 |
| Cyprus | 4.27 | 4.15 | 4.39 | 2011 |
| Cyprus | 4.38 | 4.26 | 4.5 | 2012 |
| Cyprus | 4.48 | 4.35 | 4.6 | 2013 |
| Cyprus | 4.57 | 4.43 | 4.7 | 2014 |
| Cyprus | 4.66 | 4.5 | 4.81 | 2015 |
| Cyprus | 4.74 | 4.56 | 4.92 | 2016 |
| Cyprus | 4.83 | 4.61 | 5.06 | 2017 |
| Cyprus | 4.92 | 4.64 | 5.21 | 2018 |
| Cyprus | 5.02 | 4.67 | 5.37 | 2019 |
| Cyprus | 5.12 | 4.69 | 5.55 | 2020 |
| Cyprus | 5.22 | 4.7 | 5.75 | 2021 |
| Cyprus | 5.33 | 4.7 | 5.97 | 2022 |
| Cyprus | 5.45 | 4.7 | 6.19 | 2023 |
| Cyprus | 5.57 | 4.7 | 6.44 | 2024 |
| Cyprus | 5.7 | 4.69 | 6.7 | 2025 |
| Cyprus | 5.83 | 4.68 | 6.98 | 2026 |
| Cyprus | 5.97 | 4.66 | 7.28 | 2027 |
| Cyprus | 6.12 | 4.63 | 7.6 | 2028 |
| Cyprus | 6.27 | 4.6 | 7.94 | 2029 |
| Cyprus | 6.43 | 4.56 | 8.31 | 2030 |
| Czech Republic | 11 | 10.84 | 11.16 | 1990 |
| Czech Republic | 11.47 | 11.35 | 11.58 | 1991 |
| Czech Republic | 11.96 | 11.85 | 12.07 | 1992 |
| Czech Republic | 12.48 | 12.36 | 12.59 | 1993 |
| Czech Republic | 13 | 12.88 | 13.11 | 1994 |
| Czech Republic | 13.41 | 13.29 | 13.53 | 1995 |
| Czech Republic | 13.66 | 13.54 | 13.78 | 1996 |
| Czech Republic | 13.78 | 13.67 | 13.9 | 1997 |
| Czech Republic | 13.86 | 13.75 | 13.98 | 1998 |
| Czech Republic | 14.01 | 13.89 | 14.12 | 1999 |
| Czech Republic | 14.29 | 14.17 | 14.41 | 2000 |
| Czech Republic | 14.73 | 14.61 | 14.85 | 2001 |
| Czech Republic | 15.36 | 15.23 | 15.48 | 2002 |
| Czech Republic | 16.08 | 15.95 | 16.2 | 2003 |
| Czech Republic | 16.6 | 16.47 | 16.73 | 2004 |
| Czech Republic | 16.85 | 16.72 | 16.98 | 2005 |
| Czech Republic | 16.86 | 16.73 | 16.98 | 2006 |
| Czech Republic | 16.72 | 16.59 | 16.84 | 2007 |
| Czech Republic | 16.48 | 16.36 | 16.6 | 2008 |
| Czech Republic | 16.14 | 16.02 | 16.26 | 2009 |
| Czech Republic | 15.74 | 15.63 | 15.86 | 2010 |
| Czech Republic | 15.28 | 15.17 | 15.4 | 2011 |
| Czech Republic | 14.8 | 14.69 | 14.91 | 2012 |
| Czech Republic | 14.33 | 14.22 | 14.44 | 2013 |
| Czech Republic | 13.9 | 13.79 | 14.01 | 2014 |
| Czech Republic | 13.55 | 13.44 | 13.65 | 2015 |
| Czech Republic | 13.25 | 13.15 | 13.36 | 2016 |
| Czech Republic | 13.01 | 12.86 | 13.16 | 2017 |
| Czech Republic | 12.76 | 12.5 | 13.03 | 2018 |
| Czech Republic | 12.52 | 12.09 | 12.95 | 2019 |
| Czech Republic | 12.27 | 11.65 | 12.89 | 2020 |
| Czech Republic | 12.02 | 11.19 | 12.85 | 2021 |
| Czech Republic | 11.77 | 10.72 | 12.82 | 2022 |
| Czech Republic | 11.52 | 10.24 | 12.81 | 2023 |
| Czech Republic | 11.28 | 9.76 | 12.8 | 2024 |
| Czech Republic | 11.04 | 9.27 | 12.81 | 2025 |
| Czech Republic | 10.81 | 8.79 | 12.82 | 2026 |
| Czech Republic | 10.58 | 8.31 | 12.85 | 2027 |
| Czech Republic | 10.35 | 7.83 | 12.87 | 2028 |
| Czech Republic | 10.13 | 7.35 | 12.9 | 2029 |
| Czech Republic | 9.91 | 6.88 | 12.94 | 2030 |
| Democratic Republic of the Congo | 2.26 | 2.22 | 2.3 | 1990 |
| Democratic Republic of the Congo | 2.24 | 2.2 | 2.27 | 1991 |
| Democratic Republic of the Congo | 2.21 | 2.18 | 2.24 | 1992 |
| Democratic Republic of the Congo | 2.19 | 2.16 | 2.21 | 1993 |
| Democratic Republic of the Congo | 2.16 | 2.14 | 2.19 | 1994 |
| Democratic Republic of the Congo | 2.13 | 2.11 | 2.16 | 1995 |
| Democratic Republic of the Congo | 2.11 | 2.08 | 2.13 | 1996 |
| Democratic Republic of the Congo | 2.08 | 2.06 | 2.1 | 1997 |
| Democratic Republic of the Congo | 2.06 | 2.03 | 2.08 | 1998 |
| Democratic Republic of the Congo | 2.03 | 2.01 | 2.05 | 1999 |
| Democratic Republic of the Congo | 2.01 | 1.99 | 2.03 | 2000 |
| Democratic Republic of the Congo | 1.99 | 1.97 | 2.01 | 2001 |
| Democratic Republic of the Congo | 1.97 | 1.95 | 1.99 | 2002 |
| Democratic Republic of the Congo | 1.95 | 1.93 | 1.97 | 2003 |
| Democratic Republic of the Congo | 1.93 | 1.91 | 1.95 | 2004 |
| Democratic Republic of the Congo | 1.92 | 1.91 | 1.94 | 2005 |
| Democratic Republic of the Congo | 1.92 | 1.9 | 1.94 | 2006 |
| Democratic Republic of the Congo | 1.93 | 1.91 | 1.95 | 2007 |
| Democratic Republic of the Congo | 1.94 | 1.92 | 1.96 | 2008 |
| Democratic Republic of the Congo | 1.96 | 1.94 | 1.98 | 2009 |
| Democratic Republic of the Congo | 1.99 | 1.97 | 2.01 | 2010 |
| Democratic Republic of the Congo | 2.01 | 2 | 2.03 | 2011 |
| Democratic Republic of the Congo | 2.04 | 2.02 | 2.06 | 2012 |
| Democratic Republic of the Congo | 2.07 | 2.05 | 2.09 | 2013 |
| Democratic Republic of the Congo | 2.1 | 2.08 | 2.12 | 2014 |
| Democratic Republic of the Congo | 2.12 | 2.1 | 2.14 | 2015 |
| Democratic Republic of the Congo | 2.14 | 2.12 | 2.17 | 2016 |
| Democratic Republic of the Congo | 2.16 | 2.13 | 2.19 | 2017 |
| Democratic Republic of the Congo | 2.18 | 2.14 | 2.23 | 2018 |
| Democratic Republic of the Congo | 2.2 | 2.14 | 2.26 | 2019 |
| Democratic Republic of the Congo | 2.22 | 2.14 | 2.3 | 2020 |
| Democratic Republic of the Congo | 2.24 | 2.14 | 2.34 | 2021 |
| Democratic Republic of the Congo | 2.26 | 2.14 | 2.38 | 2022 |
| Democratic Republic of the Congo | 2.28 | 2.13 | 2.43 | 2023 |
| Democratic Republic of the Congo | 2.3 | 2.12 | 2.47 | 2024 |
| Democratic Republic of the Congo | 2.32 | 2.11 | 2.52 | 2025 |
| Democratic Republic of the Congo | 2.33 | 2.1 | 2.57 | 2026 |
| Democratic Republic of the Congo | 2.35 | 2.08 | 2.62 | 2027 |
| Democratic Republic of the Congo | 2.37 | 2.06 | 2.67 | 2028 |
| Democratic Republic of the Congo | 2.38 | 2.04 | 2.72 | 2029 |
| Democratic Republic of the Congo | 2.4 | 2.02 | 2.77 | 2030 |
| Denmark | 5.46 | 5.34 | 5.58 | 1990 |
| Denmark | 5.72 | 5.63 | 5.82 | 1991 |
| Denmark | 6 | 5.92 | 6.08 | 1992 |
| Denmark | 6.28 | 6.2 | 6.36 | 1993 |
| Denmark | 6.55 | 6.47 | 6.62 | 1994 |
| Denmark | 6.79 | 6.71 | 6.88 | 1995 |
| Denmark | 7.02 | 6.94 | 7.1 | 1996 |
| Denmark | 7.22 | 7.13 | 7.3 | 1997 |
| Denmark | 7.4 | 7.31 | 7.48 | 1998 |
| Denmark | 7.56 | 7.47 | 7.64 | 1999 |
| Denmark | 7.7 | 7.61 | 7.78 | 2000 |
| Denmark | 7.82 | 7.74 | 7.91 | 2001 |
| Denmark | 7.94 | 7.86 | 8.03 | 2002 |
| Denmark | 8.06 | 7.98 | 8.15 | 2003 |
| Denmark | 8.18 | 8.1 | 8.27 | 2004 |
| Denmark | 8.31 | 8.22 | 8.39 | 2005 |
| Denmark | 8.43 | 8.34 | 8.51 | 2006 |
| Denmark | 8.54 | 8.45 | 8.62 | 2007 |
| Denmark | 8.63 | 8.54 | 8.72 | 2008 |
| Denmark | 8.7 | 8.61 | 8.79 | 2009 |
| Denmark | 8.74 | 8.65 | 8.83 | 2010 |
| Denmark | 8.75 | 8.66 | 8.84 | 2011 |
| Denmark | 8.73 | 8.64 | 8.82 | 2012 |
| Denmark | 8.69 | 8.6 | 8.78 | 2013 |
| Denmark | 8.63 | 8.55 | 8.72 | 2014 |
| Denmark | 8.57 | 8.48 | 8.66 | 2015 |
| Denmark | 8.5 | 8.4 | 8.61 | 2016 |
| Denmark | 8.43 | 8.3 | 8.57 | 2017 |
| Denmark | 8.37 | 8.17 | 8.56 | 2018 |
| Denmark | 8.3 | 8.04 | 8.56 | 2019 |
| Denmark | 8.23 | 7.89 | 8.58 | 2020 |
| Denmark | 8.16 | 7.73 | 8.6 | 2021 |
| Denmark | 8.1 | 7.57 | 8.63 | 2022 |
| Denmark | 8.03 | 7.4 | 8.66 | 2023 |
| Denmark | 7.96 | 7.22 | 8.7 | 2024 |
| Denmark | 7.89 | 7.04 | 8.74 | 2025 |
| Denmark | 7.81 | 6.85 | 8.78 | 2026 |
| Denmark | 7.74 | 6.65 | 8.83 | 2027 |
| Denmark | 7.66 | 6.46 | 8.87 | 2028 |
| Denmark | 7.59 | 6.26 | 8.91 | 2029 |
| Denmark | 7.51 | 6.05 | 8.96 | 2030 |
| Djibouti | 0.96 | 0.83 | 1.09 | 1990 |
| Djibouti | 1.05 | 0.93 | 1.18 | 1991 |
| Djibouti | 1.14 | 1.02 | 1.26 | 1992 |
| Djibouti | 1.24 | 1.13 | 1.36 | 1993 |
| Djibouti | 1.35 | 1.23 | 1.46 | 1994 |
| Djibouti | 1.45 | 1.34 | 1.56 | 1995 |
| Djibouti | 1.56 | 1.45 | 1.67 | 1996 |
| Djibouti | 1.67 | 1.55 | 1.78 | 1997 |
| Djibouti | 1.77 | 1.66 | 1.89 | 1998 |
| Djibouti | 1.88 | 1.76 | 1.99 | 1999 |
| Djibouti | 1.98 | 1.86 | 2.1 | 2000 |
| Djibouti | 2.07 | 1.95 | 2.19 | 2001 |
| Djibouti | 2.16 | 2.04 | 2.28 | 2002 |
| Djibouti | 2.25 | 2.13 | 2.37 | 2003 |
| Djibouti | 2.33 | 2.21 | 2.45 | 2004 |
| Djibouti | 2.41 | 2.29 | 2.53 | 2005 |
| Djibouti | 2.48 | 2.36 | 2.61 | 2006 |
| Djibouti | 2.55 | 2.43 | 2.68 | 2007 |
| Djibouti | 2.62 | 2.49 | 2.74 | 2008 |
| Djibouti | 2.68 | 2.56 | 2.81 | 2009 |
| Djibouti | 2.75 | 2.62 | 2.88 | 2010 |
| Djibouti | 2.82 | 2.68 | 2.95 | 2011 |
| Djibouti | 2.88 | 2.74 | 3.02 | 2012 |
| Djibouti | 2.94 | 2.8 | 3.09 | 2013 |
| Djibouti | 3.01 | 2.85 | 3.17 | 2014 |
| Djibouti | 3.07 | 2.89 | 3.25 | 2015 |
| Djibouti | 3.13 | 2.92 | 3.33 | 2016 |
| Djibouti | 3.19 | 2.95 | 3.43 | 2017 |
| Djibouti | 3.25 | 2.96 | 3.54 | 2018 |
| Djibouti | 3.32 | 2.97 | 3.67 | 2019 |
| Djibouti | 3.39 | 2.97 | 3.8 | 2020 |
| Djibouti | 3.46 | 2.97 | 3.95 | 2021 |
| Djibouti | 3.53 | 2.95 | 4.1 | 2022 |
| Djibouti | 3.6 | 2.94 | 4.27 | 2023 |
| Djibouti | 3.68 | 2.91 | 4.45 | 2024 |
| Djibouti | 3.76 | 2.88 | 4.65 | 2025 |
| Djibouti | 3.85 | 2.85 | 4.85 | 2026 |
| Djibouti | 3.94 | 2.8 | 5.07 | 2027 |
| Djibouti | 4.03 | 2.76 | 5.3 | 2028 |
| Djibouti | 4.12 | 2.7 | 5.54 | 2029 |
| Djibouti | 4.22 | 2.64 | 5.8 | 2030 |
| Dominica | 0.02 | 0.01 | 0.03 | 1990 |
| Dominica | 0.02 | 0.01 | 0.02 | 1991 |
| Dominica | 0.01 | 0.01 | 0.02 | 1992 |
| Dominica | 0.01 | 0.01 | 0.01 | 1993 |
| Dominica | 0.01 | 0.01 | 0.01 | 1994 |
| Dominica | 0.01 | 0 | 0.01 | 1995 |
| Dominica | 0 | 0 | 0.01 | 1996 |
| Dominica | 0 | 0 | 0 | 1997 |
| Dominica | 0 | 0 | 0 | 1998 |
| Dominica | 0 | 0 | 0 | 1999 |
| Dominica | 0 | 0 | 0 | 2000 |
| Dominica | 0 | 0 | 0 | 2001 |
| Dominica | 0 | 0 | 0 | 2002 |
| Dominica | 0 | 0 | 0 | 2003 |
| Dominica | 0 | 0 | 0 | 2004 |
| Dominica | 0 | 0 | 0 | 2005 |
| Dominica | 0 | 0 | 0 | 2006 |
| Dominica | 0 | 0 | 0 | 2007 |
| Dominica | 0 | 0 | 0 | 2008 |
| Dominica | 0 | 0 | 0 | 2009 |
| Dominica | 0 | 0 | 0 | 2010 |
| Dominica | 0 | 0 | 0.01 | 2011 |
| Dominica | 0.01 | 0 | 0.01 | 2012 |
| Dominica | 0.01 | 0.01 | 0.01 | 2013 |
| Dominica | 0.02 | 0.01 | 0.02 | 2014 |
| Dominica | 0.03 | 0.02 | 0.03 | 2015 |
| Dominica | 0.04 | 0.03 | 0.05 | 2016 |
| Dominica | 0.06 | 0.05 | 0.08 | 2017 |
| Dominica | 0.1 | 0.06 | 0.13 | 2018 |
| Dominica | 0.14 | 0.07 | 0.22 | 2019 |
| Dominica | 0.22 | 0.06 | 0.37 | 2020 |
| Dominica | 0.33 | 0.02 | 0.64 | 2021 |
| Dominica | 0.5 | -0.09 | 1.09 | 2022 |
| Dominica | 0.76 | -0.34 | 1.86 | 2023 |
| Dominica | 1.16 | -0.84 | 3.16 | 2024 |
| Dominica | 1.77 | -1.81 | 5.35 | 2025 |
| Dominica | 2.7 | -3.61 | 9 | 2026 |
| Dominica | 4.12 | -6.85 | 15.08 | 2027 |
| Dominica | 6.28 | -12.6 | 25.17 | 2028 |
| Dominica | 9.59 | -22.65 | 41.83 | 2029 |
| Dominica | 14.65 | -39.97 | 69.27 | 2030 |
| Ecuador | 3.94 | 3.82 | 4.05 | 1990 |
| Ecuador | 3.88 | 3.79 | 3.96 | 1991 |
| Ecuador | 3.8 | 3.73 | 3.88 | 1992 |
| Ecuador | 3.71 | 3.64 | 3.78 | 1993 |
| Ecuador | 3.6 | 3.53 | 3.66 | 1994 |
| Ecuador | 3.46 | 3.4 | 3.53 | 1995 |
| Ecuador | 3.34 | 3.28 | 3.4 | 1996 |
| Ecuador | 3.24 | 3.18 | 3.3 | 1997 |
| Ecuador | 3.19 | 3.13 | 3.25 | 1998 |
| Ecuador | 3.19 | 3.13 | 3.25 | 1999 |
| Ecuador | 3.26 | 3.2 | 3.32 | 2000 |
| Ecuador | 3.39 | 3.33 | 3.45 | 2001 |
| Ecuador | 3.58 | 3.52 | 3.63 | 2002 |
| Ecuador | 3.79 | 3.73 | 3.85 | 2003 |
| Ecuador | 4.01 | 3.95 | 4.07 | 2004 |
| Ecuador | 4.23 | 4.17 | 4.3 | 2005 |
| Ecuador | 4.45 | 4.38 | 4.51 | 2006 |
| Ecuador | 4.63 | 4.56 | 4.7 | 2007 |
| Ecuador | 4.77 | 4.71 | 4.84 | 2008 |
| Ecuador | 4.86 | 4.79 | 4.93 | 2009 |
| Ecuador | 4.91 | 4.84 | 4.98 | 2010 |
| Ecuador | 4.92 | 4.86 | 4.99 | 2011 |
| Ecuador | 4.92 | 4.86 | 4.99 | 2012 |
| Ecuador | 4.91 | 4.84 | 4.98 | 2013 |
| Ecuador | 4.9 | 4.84 | 4.97 | 2014 |
| Ecuador | 4.91 | 4.85 | 4.98 | 2015 |
| Ecuador | 4.93 | 4.86 | 5 | 2016 |
| Ecuador | 4.96 | 4.86 | 5.06 | 2017 |
| Ecuador | 4.98 | 4.82 | 5.14 | 2018 |
| Ecuador | 5 | 4.74 | 5.26 | 2019 |
| Ecuador | 5.02 | 4.65 | 5.39 | 2020 |
| Ecuador | 5.04 | 4.55 | 5.54 | 2021 |
| Ecuador | 5.06 | 4.43 | 5.7 | 2022 |
| Ecuador | 5.08 | 4.29 | 5.88 | 2023 |
| Ecuador | 5.1 | 4.15 | 6.06 | 2024 |
| Ecuador | 5.12 | 3.99 | 6.26 | 2025 |
| Ecuador | 5.14 | 3.82 | 6.46 | 2026 |
| Ecuador | 5.16 | 3.64 | 6.68 | 2027 |
| Ecuador | 5.18 | 3.46 | 6.9 | 2028 |
| Ecuador | 5.2 | 3.26 | 7.14 | 2029 |
| Ecuador | 5.21 | 3.05 | 7.38 | 2030 |
| Egypt | 1.73 | 1.7 | 1.76 | 1990 |
| Egypt | 1.75 | 1.73 | 1.77 | 1991 |
| Egypt | 1.77 | 1.75 | 1.78 | 1992 |
| Egypt | 1.79 | 1.77 | 1.8 | 1993 |
| Egypt | 1.81 | 1.79 | 1.82 | 1994 |
| Egypt | 1.83 | 1.81 | 1.85 | 1995 |
| Egypt | 1.85 | 1.84 | 1.87 | 1996 |
| Egypt | 1.88 | 1.86 | 1.9 | 1997 |
| Egypt | 1.91 | 1.89 | 1.93 | 1998 |
| Egypt | 1.95 | 1.93 | 1.96 | 1999 |
| Egypt | 1.98 | 1.97 | 2 | 2000 |
| Egypt | 2.03 | 2.01 | 2.04 | 2001 |
| Egypt | 2.07 | 2.05 | 2.08 | 2002 |
| Egypt | 2.11 | 2.09 | 2.13 | 2003 |
| Egypt | 2.15 | 2.13 | 2.16 | 2004 |
| Egypt | 2.18 | 2.16 | 2.2 | 2005 |
| Egypt | 2.21 | 2.19 | 2.22 | 2006 |
| Egypt | 2.23 | 2.22 | 2.25 | 2007 |
| Egypt | 2.26 | 2.24 | 2.27 | 2008 |
| Egypt | 2.28 | 2.27 | 2.3 | 2009 |
| Egypt | 2.31 | 2.29 | 2.33 | 2010 |
| Egypt | 2.34 | 2.32 | 2.35 | 2011 |
| Egypt | 2.36 | 2.35 | 2.38 | 2012 |
| Egypt | 2.39 | 2.38 | 2.41 | 2013 |
| Egypt | 2.43 | 2.41 | 2.45 | 2014 |
| Egypt | 2.47 | 2.45 | 2.49 | 2015 |
| Egypt | 2.51 | 2.49 | 2.53 | 2016 |
| Egypt | 2.55 | 2.52 | 2.57 | 2017 |
| Egypt | 2.58 | 2.54 | 2.62 | 2018 |
| Egypt | 2.62 | 2.57 | 2.68 | 2019 |
| Egypt | 2.66 | 2.59 | 2.74 | 2020 |
| Egypt | 2.7 | 2.6 | 2.8 | 2021 |
| Egypt | 2.74 | 2.61 | 2.86 | 2022 |
| Egypt | 2.77 | 2.62 | 2.93 | 2023 |
| Egypt | 2.81 | 2.63 | 2.99 | 2024 |
| Egypt | 2.85 | 2.64 | 3.06 | 2025 |
| Egypt | 2.89 | 2.64 | 3.13 | 2026 |
| Egypt | 2.92 | 2.64 | 3.21 | 2027 |
| Egypt | 2.96 | 2.64 | 3.28 | 2028 |
| Egypt | 3 | 2.63 | 3.36 | 2029 |
| Egypt | 3.03 | 2.62 | 3.44 | 2030 |
| El Salvador | 2.71 | 2.61 | 2.81 | 1990 |
| El Salvador | 2.74 | 2.65 | 2.82 | 1991 |
| El Salvador | 2.76 | 2.69 | 2.83 | 1992 |
| El Salvador | 2.79 | 2.72 | 2.85 | 1993 |
| El Salvador | 2.81 | 2.75 | 2.87 | 1994 |
| El Salvador | 2.83 | 2.77 | 2.89 | 1995 |
| El Salvador | 2.85 | 2.8 | 2.91 | 1996 |
| El Salvador | 2.87 | 2.82 | 2.93 | 1997 |
| El Salvador | 2.89 | 2.84 | 2.95 | 1998 |
| El Salvador | 2.91 | 2.86 | 2.97 | 1999 |
| El Salvador | 2.94 | 2.89 | 2.99 | 2000 |
| El Salvador | 2.97 | 2.92 | 3.03 | 2001 |
| El Salvador | 3.01 | 2.95 | 3.06 | 2002 |
| El Salvador | 3.05 | 2.99 | 3.1 | 2003 |
| El Salvador | 3.09 | 3.04 | 3.15 | 2004 |
| El Salvador | 3.14 | 3.09 | 3.2 | 2005 |
| El Salvador | 3.19 | 3.14 | 3.25 | 2006 |
| El Salvador | 3.24 | 3.18 | 3.29 | 2007 |
| El Salvador | 3.28 | 3.22 | 3.34 | 2008 |
| El Salvador | 3.32 | 3.26 | 3.38 | 2009 |
| El Salvador | 3.35 | 3.3 | 3.41 | 2010 |
| El Salvador | 3.38 | 3.33 | 3.44 | 2011 |
| El Salvador | 3.41 | 3.35 | 3.47 | 2012 |
| El Salvador | 3.43 | 3.38 | 3.49 | 2013 |
| El Salvador | 3.46 | 3.39 | 3.52 | 2014 |
| El Salvador | 3.48 | 3.41 | 3.54 | 2015 |
| El Salvador | 3.49 | 3.41 | 3.57 | 2016 |
| El Salvador | 3.51 | 3.41 | 3.61 | 2017 |
| El Salvador | 3.53 | 3.4 | 3.65 | 2018 |
| El Salvador | 3.54 | 3.39 | 3.69 | 2019 |
| El Salvador | 3.56 | 3.37 | 3.74 | 2020 |
| El Salvador | 3.57 | 3.35 | 3.79 | 2021 |
| El Salvador | 3.58 | 3.32 | 3.84 | 2022 |
| El Salvador | 3.59 | 3.28 | 3.9 | 2023 |
| El Salvador | 3.6 | 3.25 | 3.95 | 2024 |
| El Salvador | 3.6 | 3.2 | 4 | 2025 |
| El Salvador | 3.61 | 3.16 | 4.06 | 2026 |
| El Salvador | 3.61 | 3.11 | 4.11 | 2027 |
| El Salvador | 3.61 | 3.05 | 4.17 | 2028 |
| El Salvador | 3.61 | 3 | 4.22 | 2029 |
| El Salvador | 3.61 | 2.94 | 4.28 | 2030 |
| Equatorial Guinea | 2.18 | 1.96 | 2.41 | 1990 |
| Equatorial Guinea | 2.26 | 2.05 | 2.47 | 1991 |
| Equatorial Guinea | 2.33 | 2.13 | 2.52 | 1992 |
| Equatorial Guinea | 2.39 | 2.21 | 2.58 | 1993 |
| Equatorial Guinea | 2.46 | 2.29 | 2.63 | 1994 |
| Equatorial Guinea | 2.52 | 2.36 | 2.68 | 1995 |
| Equatorial Guinea | 2.58 | 2.42 | 2.74 | 1996 |
| Equatorial Guinea | 2.64 | 2.49 | 2.79 | 1997 |
| Equatorial Guinea | 2.69 | 2.54 | 2.84 | 1998 |
| Equatorial Guinea | 2.74 | 2.6 | 2.89 | 1999 |
| Equatorial Guinea | 2.8 | 2.65 | 2.94 | 2000 |
| Equatorial Guinea | 2.85 | 2.71 | 2.99 | 2001 |
| Equatorial Guinea | 2.91 | 2.77 | 3.05 | 2002 |
| Equatorial Guinea | 2.97 | 2.83 | 3.11 | 2003 |
| Equatorial Guinea | 3.03 | 2.89 | 3.17 | 2004 |
| Equatorial Guinea | 3.09 | 2.95 | 3.23 | 2005 |
| Equatorial Guinea | 3.16 | 3.02 | 3.3 | 2006 |
| Equatorial Guinea | 3.23 | 3.09 | 3.38 | 2007 |
| Equatorial Guinea | 3.3 | 3.16 | 3.45 | 2008 |
| Equatorial Guinea | 3.38 | 3.23 | 3.52 | 2009 |
| Equatorial Guinea | 3.45 | 3.3 | 3.6 | 2010 |
| Equatorial Guinea | 3.52 | 3.37 | 3.68 | 2011 |
| Equatorial Guinea | 3.59 | 3.43 | 3.76 | 2012 |
| Equatorial Guinea | 3.67 | 3.5 | 3.84 | 2013 |
| Equatorial Guinea | 3.75 | 3.56 | 3.94 | 2014 |
| Equatorial Guinea | 3.83 | 3.62 | 4.04 | 2015 |
| Equatorial Guinea | 3.92 | 3.68 | 4.15 | 2016 |
| Equatorial Guinea | 4.01 | 3.73 | 4.28 | 2017 |
| Equatorial Guinea | 4.1 | 3.78 | 4.42 | 2018 |
| Equatorial Guinea | 4.2 | 3.82 | 4.58 | 2019 |
| Equatorial Guinea | 4.3 | 3.86 | 4.75 | 2020 |
| Equatorial Guinea | 4.41 | 3.9 | 4.93 | 2021 |
| Equatorial Guinea | 4.53 | 3.93 | 5.13 | 2022 |
| Equatorial Guinea | 4.65 | 3.96 | 5.35 | 2023 |
| Equatorial Guinea | 4.79 | 3.99 | 5.58 | 2024 |
| Equatorial Guinea | 4.92 | 4.01 | 5.84 | 2025 |
| Equatorial Guinea | 5.07 | 4.03 | 6.11 | 2026 |
| Equatorial Guinea | 5.23 | 4.05 | 6.4 | 2027 |
| Equatorial Guinea | 5.39 | 4.06 | 6.71 | 2028 |
| Equatorial Guinea | 5.56 | 4.07 | 7.05 | 2029 |
| Equatorial Guinea | 5.74 | 4.08 | 7.4 | 2030 |
| Eritrea | 1.74 | 1.65 | 1.84 | 1990 |
| Eritrea | 1.85 | 1.77 | 1.94 | 1991 |
| Eritrea | 1.97 | 1.89 | 2.05 | 1992 |
| Eritrea | 2.09 | 2.02 | 2.16 | 1993 |
| Eritrea | 2.22 | 2.14 | 2.29 | 1994 |
| Eritrea | 2.34 | 2.27 | 2.41 | 1995 |
| Eritrea | 2.47 | 2.4 | 2.54 | 1996 |
| Eritrea | 2.59 | 2.52 | 2.66 | 1997 |
| Eritrea | 2.7 | 2.63 | 2.78 | 1998 |
| Eritrea | 2.81 | 2.73 | 2.88 | 1999 |
| Eritrea | 2.9 | 2.83 | 2.98 | 2000 |
| Eritrea | 2.99 | 2.92 | 3.07 | 2001 |
| Eritrea | 3.07 | 3 | 3.15 | 2002 |
| Eritrea | 3.14 | 3.06 | 3.22 | 2003 |
| Eritrea | 3.2 | 3.12 | 3.27 | 2004 |
| Eritrea | 3.25 | 3.17 | 3.32 | 2005 |
| Eritrea | 3.28 | 3.21 | 3.36 | 2006 |
| Eritrea | 3.31 | 3.24 | 3.39 | 2007 |
| Eritrea | 3.34 | 3.26 | 3.41 | 2008 |
| Eritrea | 3.36 | 3.28 | 3.44 | 2009 |
| Eritrea | 3.38 | 3.3 | 3.45 | 2010 |
| Eritrea | 3.39 | 3.31 | 3.47 | 2011 |
| Eritrea | 3.4 | 3.32 | 3.48 | 2012 |
| Eritrea | 3.41 | 3.33 | 3.49 | 2013 |
| Eritrea | 3.42 | 3.33 | 3.5 | 2014 |
| Eritrea | 3.42 | 3.33 | 3.51 | 2015 |
| Eritrea | 3.42 | 3.31 | 3.53 | 2016 |
| Eritrea | 3.42 | 3.29 | 3.55 | 2017 |
| Eritrea | 3.41 | 3.25 | 3.58 | 2018 |
| Eritrea | 3.41 | 3.21 | 3.61 | 2019 |
| Eritrea | 3.4 | 3.15 | 3.65 | 2020 |
| Eritrea | 3.39 | 3.1 | 3.69 | 2021 |
| Eritrea | 3.38 | 3.03 | 3.73 | 2022 |
| Eritrea | 3.37 | 2.96 | 3.78 | 2023 |
| Eritrea | 3.36 | 2.89 | 3.83 | 2024 |
| Eritrea | 3.34 | 2.81 | 3.88 | 2025 |
| Eritrea | 3.33 | 2.73 | 3.93 | 2026 |
| Eritrea | 3.31 | 2.65 | 3.98 | 2027 |
| Eritrea | 3.3 | 2.56 | 4.03 | 2028 |
| Eritrea | 3.28 | 2.47 | 4.08 | 2029 |
| Eritrea | 3.26 | 2.38 | 4.13 | 2030 |
| Estonia | 4.09 | 3.89 | 4.29 | 1990 |
| Estonia | 4.53 | 4.36 | 4.69 | 1991 |
| Estonia | 5.01 | 4.86 | 5.16 | 1992 |
| Estonia | 5.55 | 5.4 | 5.7 | 1993 |
| Estonia | 6.16 | 6 | 6.32 | 1994 |
| Estonia | 6.85 | 6.68 | 7.01 | 1995 |
| Estonia | 7.64 | 7.46 | 7.82 | 1996 |
| Estonia | 8.55 | 8.36 | 8.74 | 1997 |
| Estonia | 9.54 | 9.34 | 9.74 | 1998 |
| Estonia | 10.55 | 10.33 | 10.76 | 1999 |
| Estonia | 11.53 | 11.3 | 11.75 | 2000 |
| Estonia | 12.43 | 12.2 | 12.67 | 2001 |
| Estonia | 13.26 | 13.01 | 13.51 | 2002 |
| Estonia | 13.95 | 13.7 | 14.21 | 2003 |
| Estonia | 14.45 | 14.19 | 14.71 | 2004 |
| Estonia | 14.73 | 14.46 | 14.99 | 2005 |
| Estonia | 14.76 | 14.5 | 15.02 | 2006 |
| Estonia | 14.59 | 14.33 | 14.84 | 2007 |
| Estonia | 14.27 | 14.02 | 14.52 | 2008 |
| Estonia | 13.93 | 13.68 | 14.18 | 2009 |
| Estonia | 13.65 | 13.4 | 13.9 | 2010 |
| Estonia | 13.47 | 13.22 | 13.71 | 2011 |
| Estonia | 13.36 | 13.12 | 13.6 | 2012 |
| Estonia | 13.25 | 13.01 | 13.49 | 2013 |
| Estonia | 13.09 | 12.85 | 13.33 | 2014 |
| Estonia | 12.86 | 12.62 | 13.11 | 2015 |
| Estonia | 12.57 | 12.3 | 12.85 | 2016 |
| Estonia | 12.27 | 11.91 | 12.63 | 2017 |
| Estonia | 11.96 | 11.43 | 12.48 | 2018 |
| Estonia | 11.64 | 10.91 | 12.38 | 2019 |
| Estonia | 11.33 | 10.36 | 12.31 | 2020 |
| Estonia | 11.02 | 9.79 | 12.25 | 2021 |
| Estonia | 10.71 | 9.21 | 12.21 | 2022 |
| Estonia | 10.4 | 8.63 | 12.17 | 2023 |
| Estonia | 10.09 | 8.05 | 12.14 | 2024 |
| Estonia | 9.79 | 7.47 | 12.11 | 2025 |
| Estonia | 9.49 | 6.89 | 12.08 | 2026 |
| Estonia | 9.19 | 6.33 | 12.05 | 2027 |
| Estonia | 8.9 | 5.78 | 12.02 | 2028 |
| Estonia | 8.62 | 5.24 | 11.99 | 2029 |
| Estonia | 8.34 | 4.72 | 11.96 | 2030 |
| Ethiopia | 3.08 | 3.04 | 3.12 | 1990 |
| Ethiopia | 3.09 | 3.06 | 3.13 | 1991 |
| Ethiopia | 3.1 | 3.07 | 3.13 | 1992 |
| Ethiopia | 3.09 | 3.06 | 3.12 | 1993 |
| Ethiopia | 3.06 | 3.03 | 3.09 | 1994 |
| Ethiopia | 3.01 | 2.98 | 3.03 | 1995 |
| Ethiopia | 2.94 | 2.91 | 2.96 | 1996 |
| Ethiopia | 2.85 | 2.83 | 2.88 | 1997 |
| Ethiopia | 2.77 | 2.75 | 2.79 | 1998 |
| Ethiopia | 2.69 | 2.67 | 2.71 | 1999 |
| Ethiopia | 2.63 | 2.61 | 2.65 | 2000 |
| Ethiopia | 2.58 | 2.56 | 2.6 | 2001 |
| Ethiopia | 2.55 | 2.53 | 2.57 | 2002 |
| Ethiopia | 2.52 | 2.5 | 2.54 | 2003 |
| Ethiopia | 2.5 | 2.48 | 2.52 | 2004 |
| Ethiopia | 2.48 | 2.46 | 2.5 | 2005 |
| Ethiopia | 2.47 | 2.45 | 2.49 | 2006 |
| Ethiopia | 2.45 | 2.43 | 2.47 | 2007 |
| Ethiopia | 2.44 | 2.42 | 2.46 | 2008 |
| Ethiopia | 2.42 | 2.4 | 2.44 | 2009 |
| Ethiopia | 2.39 | 2.37 | 2.41 | 2010 |
| Ethiopia | 2.37 | 2.35 | 2.38 | 2011 |
| Ethiopia | 2.34 | 2.32 | 2.36 | 2012 |
| Ethiopia | 2.31 | 2.29 | 2.33 | 2013 |
| Ethiopia | 2.29 | 2.27 | 2.31 | 2014 |
| Ethiopia | 2.27 | 2.25 | 2.29 | 2015 |
| Ethiopia | 2.26 | 2.24 | 2.28 | 2016 |
| Ethiopia | 2.25 | 2.22 | 2.28 | 2017 |
| Ethiopia | 2.24 | 2.2 | 2.28 | 2018 |
| Ethiopia | 2.23 | 2.17 | 2.29 | 2019 |
| Ethiopia | 2.22 | 2.14 | 2.3 | 2020 |
| Ethiopia | 2.21 | 2.1 | 2.31 | 2021 |
| Ethiopia | 2.2 | 2.07 | 2.33 | 2022 |
| Ethiopia | 2.19 | 2.03 | 2.35 | 2023 |
| Ethiopia | 2.18 | 1.99 | 2.37 | 2024 |
| Ethiopia | 2.17 | 1.95 | 2.39 | 2025 |
| Ethiopia | 2.16 | 1.91 | 2.42 | 2026 |
| Ethiopia | 2.15 | 1.87 | 2.44 | 2027 |
| Ethiopia | 2.15 | 1.82 | 2.47 | 2028 |
| Ethiopia | 2.14 | 1.78 | 2.5 | 2029 |
| Ethiopia | 2.13 | 1.73 | 2.53 | 2030 |
| Federated States of Micronesia | 0 | 0 | 0.01 | 1990 |
| Federated States of Micronesia | 0.01 | 0 | 0.01 | 1991 |
| Federated States of Micronesia | 0.01 | 0 | 0.02 | 1992 |
| Federated States of Micronesia | 0.01 | 0 | 0.02 | 1993 |
| Federated States of Micronesia | 0.02 | 0 | 0.03 | 1994 |
| Federated States of Micronesia | 0.02 | 0.01 | 0.04 | 1995 |
| Federated States of Micronesia | 0.03 | 0.01 | 0.06 | 1996 |
| Federated States of Micronesia | 0.04 | 0.01 | 0.07 | 1997 |
| Federated States of Micronesia | 0.06 | 0.02 | 0.1 | 1998 |
| Federated States of Micronesia | 0.08 | 0.03 | 0.13 | 1999 |
| Federated States of Micronesia | 0.11 | 0.05 | 0.16 | 2000 |
| Federated States of Micronesia | 0.14 | 0.07 | 0.21 | 2001 |
| Federated States of Micronesia | 0.18 | 0.1 | 0.26 | 2002 |
| Federated States of Micronesia | 0.24 | 0.14 | 0.33 | 2003 |
| Federated States of Micronesia | 0.3 | 0.19 | 0.41 | 2004 |
| Federated States of Micronesia | 0.37 | 0.25 | 0.5 | 2005 |
| Federated States of Micronesia | 0.46 | 0.32 | 0.61 | 2006 |
| Federated States of Micronesia | 0.56 | 0.4 | 0.72 | 2007 |
| Federated States of Micronesia | 0.67 | 0.49 | 0.84 | 2008 |
| Federated States of Micronesia | 0.78 | 0.59 | 0.97 | 2009 |
| Federated States of Micronesia | 0.9 | 0.69 | 1.11 | 2010 |
| Federated States of Micronesia | 1.02 | 0.79 | 1.25 | 2011 |
| Federated States of Micronesia | 1.14 | 0.88 | 1.4 | 2012 |
| Federated States of Micronesia | 1.27 | 0.97 | 1.57 | 2013 |
| Federated States of Micronesia | 1.39 | 1.04 | 1.74 | 2014 |
| Federated States of Micronesia | 1.49 | 1.08 | 1.91 | 2015 |
| Federated States of Micronesia | 1.57 | 1.09 | 2.05 | 2016 |
| Federated States of Micronesia | 1.62 | 1.06 | 2.17 | 2017 |
| Federated States of Micronesia | 1.64 | 1.01 | 2.28 | 2018 |
| Federated States of Micronesia | 1.65 | 0.94 | 2.36 | 2019 |
| Federated States of Micronesia | 1.64 | 0.85 | 2.43 | 2020 |
| Federated States of Micronesia | 1.61 | 0.75 | 2.47 | 2021 |
| Federated States of Micronesia | 1.56 | 0.64 | 2.48 | 2022 |
| Federated States of Micronesia | 1.51 | 0.52 | 2.49 | 2023 |
| Federated States of Micronesia | 1.44 | 0.41 | 2.48 | 2024 |
| Federated States of Micronesia | 1.38 | 0.3 | 2.45 | 2025 |
| Federated States of Micronesia | 1.3 | 0.19 | 2.41 | 2026 |
| Federated States of Micronesia | 1.23 | 0.09 | 2.36 | 2027 |
| Federated States of Micronesia | 1.15 | 0 | 2.3 | 2028 |
| Federated States of Micronesia | 1.07 | -0.08 | 2.23 | 2029 |
| Federated States of Micronesia | 1 | -0.16 | 2.15 | 2030 |
| Fiji | 2.45 | 2.21 | 2.68 | 1990 |
| Fiji | 2.43 | 2.23 | 2.64 | 1991 |
| Fiji | 2.42 | 2.24 | 2.61 | 1992 |
| Fiji | 2.41 | 2.24 | 2.58 | 1993 |
| Fiji | 2.39 | 2.24 | 2.55 | 1994 |
| Fiji | 2.38 | 2.24 | 2.52 | 1995 |
| Fiji | 2.37 | 2.23 | 2.5 | 1996 |
| Fiji | 2.35 | 2.22 | 2.48 | 1997 |
| Fiji | 2.33 | 2.21 | 2.45 | 1998 |
| Fiji | 2.32 | 2.2 | 2.43 | 1999 |
| Fiji | 2.3 | 2.19 | 2.42 | 2000 |
| Fiji | 2.29 | 2.18 | 2.4 | 2001 |
| Fiji | 2.27 | 2.17 | 2.38 | 2002 |
| Fiji | 2.26 | 2.15 | 2.37 | 2003 |
| Fiji | 2.25 | 2.14 | 2.35 | 2004 |
| Fiji | 2.24 | 2.13 | 2.34 | 2005 |
| Fiji | 2.23 | 2.13 | 2.33 | 2006 |
| Fiji | 2.22 | 2.12 | 2.32 | 2007 |
| Fiji | 2.21 | 2.11 | 2.31 | 2008 |
| Fiji | 2.2 | 2.1 | 2.3 | 2009 |
| Fiji | 2.19 | 2.09 | 2.29 | 2010 |
| Fiji | 2.19 | 2.08 | 2.29 | 2011 |
| Fiji | 2.18 | 2.07 | 2.28 | 2012 |
| Fiji | 2.17 | 2.06 | 2.28 | 2013 |
| Fiji | 2.16 | 2.04 | 2.28 | 2014 |
| Fiji | 2.16 | 2.03 | 2.29 | 2015 |
| Fiji | 2.15 | 2.01 | 2.3 | 2016 |
| Fiji | 2.14 | 1.98 | 2.31 | 2017 |
| Fiji | 2.14 | 1.95 | 2.32 | 2018 |
| Fiji | 2.13 | 1.93 | 2.34 | 2019 |
| Fiji | 2.13 | 1.89 | 2.36 | 2020 |
| Fiji | 2.12 | 1.86 | 2.38 | 2021 |
| Fiji | 2.12 | 1.83 | 2.41 | 2022 |
| Fiji | 2.11 | 1.79 | 2.44 | 2023 |
| Fiji | 2.11 | 1.75 | 2.47 | 2024 |
| Fiji | 2.11 | 1.71 | 2.5 | 2025 |
| Fiji | 2.1 | 1.67 | 2.53 | 2026 |
| Fiji | 2.1 | 1.63 | 2.57 | 2027 |
| Fiji | 2.1 | 1.59 | 2.61 | 2028 |
| Fiji | 2.1 | 1.55 | 2.65 | 2029 |
| Fiji | 2.1 | 1.51 | 2.69 | 2030 |
| Finland | 8.37 | 8.22 | 8.52 | 1990 |
| Finland | 8.51 | 8.39 | 8.63 | 1991 |
| Finland | 8.65 | 8.55 | 8.75 | 1992 |
| Finland | 8.79 | 8.7 | 8.89 | 1993 |
| Finland | 8.93 | 8.84 | 9.02 | 1994 |
| Finland | 9.06 | 8.97 | 9.15 | 1995 |
| Finland | 9.18 | 9.09 | 9.27 | 1996 |
| Finland | 9.3 | 9.21 | 9.39 | 1997 |
| Finland | 9.4 | 9.31 | 9.49 | 1998 |
| Finland | 9.48 | 9.4 | 9.57 | 1999 |
| Finland | 9.55 | 9.46 | 9.64 | 2000 |
| Finland | 9.6 | 9.51 | 9.69 | 2001 |
| Finland | 9.63 | 9.55 | 9.72 | 2002 |
| Finland | 9.66 | 9.57 | 9.75 | 2003 |
| Finland | 9.69 | 9.6 | 9.77 | 2004 |
| Finland | 9.71 | 9.62 | 9.8 | 2005 |
| Finland | 9.73 | 9.64 | 9.82 | 2006 |
| Finland | 9.76 | 9.67 | 9.84 | 2007 |
| Finland | 9.78 | 9.69 | 9.87 | 2008 |
| Finland | 9.8 | 9.71 | 9.88 | 2009 |
| Finland | 9.8 | 9.71 | 9.89 | 2010 |
| Finland | 9.78 | 9.7 | 9.87 | 2011 |
| Finland | 9.75 | 9.66 | 9.83 | 2012 |
| Finland | 9.69 | 9.6 | 9.77 | 2013 |
| Finland | 9.62 | 9.53 | 9.7 | 2014 |
| Finland | 9.54 | 9.44 | 9.63 | 2015 |
| Finland | 9.46 | 9.35 | 9.57 | 2016 |
| Finland | 9.39 | 9.25 | 9.53 | 2017 |
| Finland | 9.32 | 9.14 | 9.51 | 2018 |
| Finland | 9.25 | 9.01 | 9.5 | 2019 |
| Finland | 9.19 | 8.88 | 9.49 | 2020 |
| Finland | 9.12 | 8.74 | 9.5 | 2021 |
| Finland | 9.06 | 8.6 | 9.52 | 2022 |
| Finland | 8.99 | 8.45 | 9.54 | 2023 |
| Finland | 8.93 | 8.3 | 9.56 | 2024 |
| Finland | 8.87 | 8.15 | 9.59 | 2025 |
| Finland | 8.81 | 7.99 | 9.63 | 2026 |
| Finland | 8.75 | 7.83 | 9.67 | 2027 |
| Finland | 8.69 | 7.67 | 9.71 | 2028 |
| Finland | 8.64 | 7.51 | 9.76 | 2029 |
| Finland | 8.58 | 7.35 | 9.81 | 2030 |
| France | 7.27 | 7.22 | 7.32 | 1990 |
| France | 7.33 | 7.3 | 7.37 | 1991 |
| France | 7.4 | 7.37 | 7.44 | 1992 |
| France | 7.46 | 7.43 | 7.5 | 1993 |
| France | 7.51 | 7.48 | 7.55 | 1994 |
| France | 7.55 | 7.51 | 7.58 | 1995 |
| France | 7.58 | 7.54 | 7.61 | 1996 |
| France | 7.62 | 7.58 | 7.65 | 1997 |
| France | 7.65 | 7.62 | 7.68 | 1998 |
| France | 7.66 | 7.63 | 7.7 | 1999 |
| France | 7.66 | 7.62 | 7.69 | 2000 |
| France | 7.64 | 7.61 | 7.68 | 2001 |
| France | 7.62 | 7.59 | 7.66 | 2002 |
| France | 7.59 | 7.56 | 7.62 | 2003 |
| France | 7.56 | 7.52 | 7.59 | 2004 |
| France | 7.54 | 7.51 | 7.58 | 2005 |
| France | 7.56 | 7.52 | 7.59 | 2006 |
| France | 7.61 | 7.57 | 7.64 | 2007 |
| France | 7.71 | 7.68 | 7.74 | 2008 |
| France | 7.84 | 7.81 | 7.88 | 2009 |
| France | 7.98 | 7.95 | 8.01 | 2010 |
| France | 8.09 | 8.05 | 8.12 | 2011 |
| France | 8.15 | 8.12 | 8.18 | 2012 |
| France | 8.17 | 8.14 | 8.2 | 2013 |
| France | 8.16 | 8.13 | 8.2 | 2014 |
| France | 8.13 | 8.1 | 8.17 | 2015 |
| France | 8.09 | 8.06 | 8.13 | 2016 |
| France | 8.05 | 8.01 | 8.1 | 2017 |
| France | 8.02 | 7.93 | 8.1 | 2018 |
| France | 7.98 | 7.85 | 8.11 | 2019 |
| France | 7.95 | 7.76 | 8.14 | 2020 |
| France | 7.92 | 7.66 | 8.17 | 2021 |
| France | 7.89 | 7.56 | 8.21 | 2022 |
| France | 7.86 | 7.46 | 8.26 | 2023 |
| France | 7.83 | 7.35 | 8.31 | 2024 |
| France | 7.8 | 7.23 | 8.36 | 2025 |
| France | 7.76 | 7.11 | 8.42 | 2026 |
| France | 7.72 | 6.98 | 8.47 | 2027 |
| France | 7.69 | 6.84 | 8.53 | 2028 |
| France | 7.65 | 6.71 | 8.59 | 2029 |
| France | 7.61 | 6.57 | 8.66 | 2030 |
| Gabon | 2.49 | 2.31 | 2.67 | 1990 |
| Gabon | 2.55 | 2.39 | 2.71 | 1991 |
| Gabon | 2.61 | 2.47 | 2.76 | 1992 |
| Gabon | 2.68 | 2.55 | 2.81 | 1993 |
| Gabon | 2.75 | 2.62 | 2.87 | 1994 |
| Gabon | 2.82 | 2.7 | 2.94 | 1995 |
| Gabon | 2.9 | 2.78 | 3.01 | 1996 |
| Gabon | 2.97 | 2.86 | 3.09 | 1997 |
| Gabon | 3.05 | 2.94 | 3.17 | 1998 |
| Gabon | 3.14 | 3.02 | 3.25 | 1999 |
| Gabon | 3.22 | 3.1 | 3.33 | 2000 |
| Gabon | 3.3 | 3.19 | 3.42 | 2001 |
| Gabon | 3.39 | 3.27 | 3.51 | 2002 |
| Gabon | 3.47 | 3.35 | 3.59 | 2003 |
| Gabon | 3.55 | 3.43 | 3.67 | 2004 |
| Gabon | 3.62 | 3.5 | 3.75 | 2005 |
| Gabon | 3.69 | 3.57 | 3.81 | 2006 |
| Gabon | 3.75 | 3.63 | 3.87 | 2007 |
| Gabon | 3.8 | 3.68 | 3.92 | 2008 |
| Gabon | 3.83 | 3.71 | 3.95 | 2009 |
| Gabon | 3.86 | 3.74 | 3.98 | 2010 |
| Gabon | 3.88 | 3.76 | 4 | 2011 |
| Gabon | 3.89 | 3.76 | 4.01 | 2012 |
| Gabon | 3.89 | 3.76 | 4.02 | 2013 |
| Gabon | 3.89 | 3.75 | 4.03 | 2014 |
| Gabon | 3.88 | 3.73 | 4.04 | 2015 |
| Gabon | 3.88 | 3.7 | 4.05 | 2016 |
| Gabon | 3.87 | 3.66 | 4.07 | 2017 |
| Gabon | 3.85 | 3.61 | 4.09 | 2018 |
| Gabon | 3.84 | 3.56 | 4.12 | 2019 |
| Gabon | 3.82 | 3.49 | 4.15 | 2020 |
| Gabon | 3.8 | 3.42 | 4.18 | 2021 |
| Gabon | 3.78 | 3.35 | 4.22 | 2022 |
| Gabon | 3.76 | 3.27 | 4.25 | 2023 |
| Gabon | 3.73 | 3.18 | 4.29 | 2024 |
| Gabon | 3.71 | 3.09 | 4.32 | 2025 |
| Gabon | 3.68 | 3 | 4.36 | 2026 |
| Gabon | 3.65 | 2.91 | 4.39 | 2027 |
| Gabon | 3.62 | 2.81 | 4.43 | 2028 |
| Gabon | 3.59 | 2.72 | 4.46 | 2029 |
| Gabon | 3.56 | 2.62 | 4.5 | 2030 |
| Georgia | 5.4 | 5.25 | 5.55 | 1990 |
| Georgia | 5.32 | 5.21 | 5.43 | 1991 |
| Georgia | 5.24 | 5.14 | 5.33 | 1992 |
| Georgia | 5.13 | 5.04 | 5.22 | 1993 |
| Georgia | 5.01 | 4.92 | 5.1 | 1994 |
| Georgia | 4.87 | 4.78 | 4.95 | 1995 |
| Georgia | 4.72 | 4.64 | 4.81 | 1996 |
| Georgia | 4.58 | 4.5 | 4.67 | 1997 |
| Georgia | 4.47 | 4.39 | 4.55 | 1998 |
| Georgia | 4.38 | 4.3 | 4.46 | 1999 |
| Georgia | 4.32 | 4.24 | 4.39 | 2000 |
| Georgia | 4.27 | 4.2 | 4.35 | 2001 |
| Georgia | 4.25 | 4.17 | 4.33 | 2002 |
| Georgia | 4.24 | 4.16 | 4.32 | 2003 |
| Georgia | 4.26 | 4.18 | 4.34 | 2004 |
| Georgia | 4.31 | 4.23 | 4.39 | 2005 |
| Georgia | 4.41 | 4.33 | 4.49 | 2006 |
| Georgia | 4.57 | 4.49 | 4.66 | 2007 |
| Georgia | 4.81 | 4.72 | 4.9 | 2008 |
| Georgia | 5.1 | 5.01 | 5.19 | 2009 |
| Georgia | 5.43 | 5.34 | 5.52 | 2010 |
| Georgia | 5.76 | 5.66 | 5.86 | 2011 |
| Georgia | 6.06 | 5.95 | 6.16 | 2012 |
| Georgia | 6.3 | 6.2 | 6.41 | 2013 |
| Georgia | 6.51 | 6.4 | 6.62 | 2014 |
| Georgia | 6.68 | 6.57 | 6.79 | 2015 |
| Georgia | 6.82 | 6.69 | 6.95 | 2016 |
| Georgia | 6.94 | 6.76 | 7.12 | 2017 |
| Georgia | 7.06 | 6.78 | 7.34 | 2018 |
| Georgia | 7.18 | 6.78 | 7.59 | 2019 |
| Georgia | 7.3 | 6.74 | 7.86 | 2020 |
| Georgia | 7.42 | 6.68 | 8.15 | 2021 |
| Georgia | 7.54 | 6.6 | 8.47 | 2022 |
| Georgia | 7.65 | 6.5 | 8.8 | 2023 |
| Georgia | 7.77 | 6.38 | 9.16 | 2024 |
| Georgia | 7.88 | 6.24 | 9.53 | 2025 |
| Georgia | 7.99 | 6.08 | 9.91 | 2026 |
| Georgia | 8.1 | 5.89 | 10.32 | 2027 |
| Georgia | 8.21 | 5.69 | 10.74 | 2028 |
| Georgia | 8.32 | 5.47 | 11.17 | 2029 |
| Georgia | 8.43 | 5.22 | 11.63 | 2030 |
| Germany | 10.71 | 10.66 | 10.77 | 1990 |
| Germany | 10.85 | 10.81 | 10.89 | 1991 |
| Germany | 10.98 | 10.94 | 11.02 | 1992 |
| Germany | 11.12 | 11.08 | 11.16 | 1993 |
| Germany | 11.21 | 11.17 | 11.25 | 1994 |
| Germany | 11.23 | 11.19 | 11.27 | 1995 |
| Germany | 11.19 | 11.15 | 11.23 | 1996 |
| Germany | 11.09 | 11.05 | 11.13 | 1997 |
| Germany | 10.99 | 10.95 | 11.02 | 1998 |
| Germany | 10.86 | 10.82 | 10.9 | 1999 |
| Germany | 10.75 | 10.71 | 10.78 | 2000 |
| Germany | 10.66 | 10.63 | 10.7 | 2001 |
| Germany | 10.6 | 10.57 | 10.64 | 2002 |
| Germany | 10.59 | 10.55 | 10.63 | 2003 |
| Germany | 10.55 | 10.51 | 10.58 | 2004 |
| Germany | 10.52 | 10.49 | 10.56 | 2005 |
| Germany | 10.51 | 10.48 | 10.55 | 2006 |
| Germany | 10.53 | 10.49 | 10.56 | 2007 |
| Germany | 10.58 | 10.54 | 10.61 | 2008 |
| Germany | 10.59 | 10.56 | 10.63 | 2009 |
| Germany | 10.56 | 10.53 | 10.6 | 2010 |
| Germany | 10.5 | 10.47 | 10.54 | 2011 |
| Germany | 10.42 | 10.39 | 10.45 | 2012 |
| Germany | 10.37 | 10.33 | 10.4 | 2013 |
| Germany | 10.31 | 10.28 | 10.35 | 2014 |
| Germany | 10.33 | 10.29 | 10.36 | 2015 |
| Germany | 10.36 | 10.32 | 10.39 | 2016 |
| Germany | 10.35 | 10.3 | 10.39 | 2017 |
| Germany | 10.35 | 10.26 | 10.44 | 2018 |
| Germany | 10.34 | 10.19 | 10.49 | 2019 |
| Germany | 10.37 | 10.15 | 10.59 | 2020 |
| Germany | 10.4 | 10.11 | 10.7 | 2021 |
| Germany | 10.42 | 10.03 | 10.8 | 2022 |
| Germany | 10.42 | 9.94 | 10.9 | 2023 |
| Germany | 10.42 | 9.83 | 11 | 2024 |
| Germany | 10.43 | 9.73 | 11.12 | 2025 |
| Germany | 10.44 | 9.63 | 11.25 | 2026 |
| Germany | 10.44 | 9.51 | 11.37 | 2027 |
| Germany | 10.43 | 9.38 | 11.49 | 2028 |
| Germany | 10.45 | 9.26 | 11.64 | 2029 |
| Germany | 10.52 | 9.18 | 11.85 | 2030 |
| Ghana | 3.34 | 3.25 | 3.43 | 1990 |
| Ghana | 3.39 | 3.32 | 3.45 | 1991 |
| Ghana | 3.44 | 3.38 | 3.5 | 1992 |
| Ghana | 3.52 | 3.45 | 3.58 | 1993 |
| Ghana | 3.61 | 3.54 | 3.67 | 1994 |
| Ghana | 3.71 | 3.65 | 3.77 | 1995 |
| Ghana | 3.82 | 3.76 | 3.88 | 1996 |
| Ghana | 3.93 | 3.87 | 3.99 | 1997 |
| Ghana | 4.01 | 3.95 | 4.07 | 1998 |
| Ghana | 4.04 | 3.97 | 4.1 | 1999 |
| Ghana | 3.99 | 3.93 | 4.05 | 2000 |
| Ghana | 3.85 | 3.79 | 3.9 | 2001 |
| Ghana | 3.64 | 3.59 | 3.7 | 2002 |
| Ghana | 3.42 | 3.36 | 3.47 | 2003 |
| Ghana | 3.18 | 3.13 | 3.23 | 2004 |
| Ghana | 2.96 | 2.91 | 3 | 2005 |
| Ghana | 2.77 | 2.73 | 2.82 | 2006 |
| Ghana | 2.65 | 2.6 | 2.69 | 2007 |
| Ghana | 2.59 | 2.54 | 2.63 | 2008 |
| Ghana | 2.58 | 2.54 | 2.63 | 2009 |
| Ghana | 2.62 | 2.58 | 2.66 | 2010 |
| Ghana | 2.69 | 2.65 | 2.74 | 2011 |
| Ghana | 2.8 | 2.76 | 2.85 | 2012 |
| Ghana | 2.94 | 2.89 | 2.98 | 2013 |
| Ghana | 3.09 | 3.04 | 3.14 | 2014 |
| Ghana | 3.25 | 3.2 | 3.3 | 2015 |
| Ghana | 3.38 | 3.33 | 3.44 | 2016 |
| Ghana | 3.5 | 3.42 | 3.57 | 2017 |
| Ghana | 3.61 | 3.48 | 3.75 | 2018 |
| Ghana | 3.73 | 3.51 | 3.95 | 2019 |
| Ghana | 3.86 | 3.53 | 4.18 | 2020 |
| Ghana | 3.98 | 3.53 | 4.43 | 2021 |
| Ghana | 4.11 | 3.51 | 4.71 | 2022 |
| Ghana | 4.25 | 3.48 | 5.01 | 2023 |
| Ghana | 4.38 | 3.43 | 5.34 | 2024 |
| Ghana | 4.53 | 3.36 | 5.69 | 2025 |
| Ghana | 4.67 | 3.28 | 6.07 | 2026 |
| Ghana | 4.82 | 3.18 | 6.47 | 2027 |
| Ghana | 4.98 | 3.05 | 6.91 | 2028 |
| Ghana | 5.14 | 2.91 | 7.38 | 2029 |
| Ghana | 5.31 | 2.74 | 7.87 | 2030 |
| Global | 4.76 | 4.75 | 4.76 | 1990 |
| Global | 4.82 | 4.81 | 4.82 | 1991 |
| Global | 4.89 | 4.88 | 4.89 | 1992 |
| Global | 4.97 | 4.96 | 4.97 | 1993 |
| Global | 5.01 | 5 | 5.01 | 1994 |
| Global | 5.01 | 5.01 | 5.02 | 1995 |
| Global | 4.98 | 4.97 | 4.98 | 1996 |
| Global | 4.95 | 4.94 | 4.95 | 1997 |
| Global | 4.95 | 4.94 | 4.95 | 1998 |
| Global | 4.97 | 4.97 | 4.98 | 1999 |
| Global | 5 | 4.99 | 5 | 2000 |
| Global | 5.02 | 5.01 | 5.02 | 2001 |
| Global | 5.05 | 5.04 | 5.05 | 2002 |
| Global | 5.08 | 5.07 | 5.09 | 2003 |
| Global | 5.09 | 5.08 | 5.09 | 2004 |
| Global | 5.1 | 5.1 | 5.11 | 2005 |
| Global | 5.08 | 5.07 | 5.08 | 2006 |
| Global | 5.07 | 5.06 | 5.07 | 2007 |
| Global | 5.09 | 5.08 | 5.09 | 2008 |
| Global | 5.09 | 5.09 | 5.1 | 2009 |
| Global | 5.09 | 5.08 | 5.09 | 2010 |
| Global | 5.07 | 5.06 | 5.07 | 2011 |
| Global | 5.05 | 5.05 | 5.06 | 2012 |
| Global | 5.04 | 5.04 | 5.05 | 2013 |
| Global | 5.04 | 5.03 | 5.04 | 2014 |
| Global | 5.05 | 5.05 | 5.06 | 2015 |
| Global | 5.04 | 5.03 | 5.04 | 2016 |
| Global | 4.99 | 4.98 | 4.99 | 2017 |
| Global | 4.95 | 4.93 | 4.97 | 2018 |
| Global | 4.9 | 4.86 | 4.95 | 2019 |
| Global | 4.86 | 4.79 | 4.94 | 2020 |
| Global | 4.82 | 4.72 | 4.92 | 2021 |
| Global | 4.77 | 4.64 | 4.91 | 2022 |
| Global | 4.73 | 4.56 | 4.9 | 2023 |
| Global | 4.68 | 4.47 | 4.89 | 2024 |
| Global | 4.63 | 4.39 | 4.88 | 2025 |
| Global | 4.59 | 4.3 | 4.87 | 2026 |
| Global | 4.54 | 4.21 | 4.87 | 2027 |
| Global | 4.49 | 4.12 | 4.86 | 2028 |
| Global | 4.45 | 4.03 | 4.87 | 2029 |
| Global | 4.42 | 3.95 | 4.88 | 2030 |
| Greece | 4.73 | 4.65 | 4.81 | 1990 |
| Greece | 4.87 | 4.8 | 4.93 | 1991 |
| Greece | 5 | 4.94 | 5.05 | 1992 |
| Greece | 5.13 | 5.08 | 5.18 | 1993 |
| Greece | 5.26 | 5.21 | 5.31 | 1994 |
| Greece | 5.39 | 5.34 | 5.44 | 1995 |
| Greece | 5.51 | 5.46 | 5.56 | 1996 |
| Greece | 5.63 | 5.58 | 5.68 | 1997 |
| Greece | 5.74 | 5.69 | 5.79 | 1998 |
| Greece | 5.85 | 5.8 | 5.9 | 1999 |
| Greece | 5.96 | 5.9 | 6.01 | 2000 |
| Greece | 6.06 | 6.01 | 6.11 | 2001 |
| Greece | 6.17 | 6.12 | 6.22 | 2002 |
| Greece | 6.27 | 6.22 | 6.32 | 2003 |
| Greece | 6.37 | 6.31 | 6.42 | 2004 |
| Greece | 6.45 | 6.39 | 6.5 | 2005 |
| Greece | 6.51 | 6.46 | 6.56 | 2006 |
| Greece | 6.56 | 6.51 | 6.61 | 2007 |
| Greece | 6.59 | 6.54 | 6.64 | 2008 |
| Greece | 6.61 | 6.56 | 6.67 | 2009 |
| Greece | 6.63 | 6.58 | 6.68 | 2010 |
| Greece | 6.64 | 6.59 | 6.69 | 2011 |
| Greece | 6.65 | 6.6 | 6.7 | 2012 |
| Greece | 6.65 | 6.6 | 6.7 | 2013 |
| Greece | 6.64 | 6.59 | 6.7 | 2014 |
| Greece | 6.63 | 6.58 | 6.69 | 2015 |
| Greece | 6.62 | 6.55 | 6.69 | 2016 |
| Greece | 6.61 | 6.52 | 6.69 | 2017 |
| Greece | 6.59 | 6.47 | 6.71 | 2018 |
| Greece | 6.58 | 6.42 | 6.74 | 2019 |
| Greece | 6.56 | 6.35 | 6.77 | 2020 |
| Greece | 6.55 | 6.29 | 6.81 | 2021 |
| Greece | 6.54 | 6.21 | 6.86 | 2022 |
| Greece | 6.52 | 6.14 | 6.91 | 2023 |
| Greece | 6.51 | 6.05 | 6.96 | 2024 |
| Greece | 6.49 | 5.97 | 7.02 | 2025 |
| Greece | 6.47 | 5.87 | 7.07 | 2026 |
| Greece | 6.45 | 5.78 | 7.13 | 2027 |
| Greece | 6.43 | 5.68 | 7.18 | 2028 |
| Greece | 6.41 | 5.57 | 7.24 | 2029 |
| Greece | 6.38 | 5.46 | 7.3 | 2030 |
| Guam | 3.08 | 2.57 | 3.6 | 1990 |
| Guam | 3.22 | 2.74 | 3.71 | 1991 |
| Guam | 3.36 | 2.91 | 3.82 | 1992 |
| Guam | 3.5 | 3.07 | 3.93 | 1993 |
| Guam | 3.64 | 3.23 | 4.04 | 1994 |
| Guam | 3.78 | 3.39 | 4.16 | 1995 |
| Guam | 3.91 | 3.55 | 4.28 | 1996 |
| Guam | 4.05 | 3.7 | 4.41 | 1997 |
| Guam | 4.19 | 3.85 | 4.54 | 1998 |
| Guam | 4.33 | 3.99 | 4.67 | 1999 |
| Guam | 4.47 | 4.13 | 4.8 | 2000 |
| Guam | 4.6 | 4.27 | 4.93 | 2001 |
| Guam | 4.73 | 4.4 | 5.06 | 2002 |
| Guam | 4.86 | 4.52 | 5.19 | 2003 |
| Guam | 4.97 | 4.64 | 5.3 | 2004 |
| Guam | 5.06 | 4.73 | 5.4 | 2005 |
| Guam | 5.15 | 4.82 | 5.48 | 2006 |
| Guam | 5.22 | 4.89 | 5.55 | 2007 |
| Guam | 5.27 | 4.94 | 5.61 | 2008 |
| Guam | 5.32 | 4.98 | 5.65 | 2009 |
| Guam | 5.35 | 5.01 | 5.69 | 2010 |
| Guam | 5.37 | 5.03 | 5.72 | 2011 |
| Guam | 5.4 | 5.04 | 5.76 | 2012 |
| Guam | 5.41 | 5.03 | 5.79 | 2013 |
| Guam | 5.43 | 5.02 | 5.84 | 2014 |
| Guam | 5.45 | 4.99 | 5.91 | 2015 |
| Guam | 5.47 | 4.95 | 5.99 | 2016 |
| Guam | 5.49 | 4.9 | 6.08 | 2017 |
| Guam | 5.52 | 4.84 | 6.2 | 2018 |
| Guam | 5.55 | 4.76 | 6.34 | 2019 |
| Guam | 5.59 | 4.68 | 6.5 | 2020 |
| Guam | 5.64 | 4.6 | 6.68 | 2021 |
| Guam | 5.69 | 4.5 | 6.88 | 2022 |
| Guam | 5.74 | 4.4 | 7.09 | 2023 |
| Guam | 5.81 | 4.29 | 7.33 | 2024 |
| Guam | 5.88 | 4.17 | 7.59 | 2025 |
| Guam | 5.95 | 4.04 | 7.86 | 2026 |
| Guam | 6.03 | 3.91 | 8.16 | 2027 |
| Guam | 6.12 | 3.76 | 8.49 | 2028 |
| Guam | 6.22 | 3.6 | 8.83 | 2029 |
| Guam | 6.31 | 3.43 | 9.19 | 2030 |
| Guatemala | 3.05 | 2.93 | 3.17 | 1990 |
| Guatemala | 3.11 | 3.03 | 3.2 | 1991 |
| Guatemala | 3.17 | 3.09 | 3.25 | 1992 |
| Guatemala | 3.2 | 3.12 | 3.28 | 1993 |
| Guatemala | 3.2 | 3.12 | 3.28 | 1994 |
| Guatemala | 3.16 | 3.08 | 3.23 | 1995 |
| Guatemala | 3.08 | 3.01 | 3.16 | 1996 |
| Guatemala | 2.97 | 2.9 | 3.05 | 1997 |
| Guatemala | 2.83 | 2.76 | 2.9 | 1998 |
| Guatemala | 2.65 | 2.59 | 2.72 | 1999 |
| Guatemala | 2.49 | 2.43 | 2.55 | 2000 |
| Guatemala | 2.4 | 2.34 | 2.46 | 2001 |
| Guatemala | 2.41 | 2.34 | 2.47 | 2002 |
| Guatemala | 2.53 | 2.46 | 2.59 | 2003 |
| Guatemala | 2.75 | 2.69 | 2.82 | 2004 |
| Guatemala | 3.05 | 2.98 | 3.12 | 2005 |
| Guatemala | 3.33 | 3.26 | 3.41 | 2006 |
| Guatemala | 3.53 | 3.45 | 3.6 | 2007 |
| Guatemala | 3.64 | 3.57 | 3.72 | 2008 |
| Guatemala | 3.71 | 3.63 | 3.78 | 2009 |
| Guatemala | 3.73 | 3.66 | 3.8 | 2010 |
| Guatemala | 3.72 | 3.65 | 3.79 | 2011 |
| Guatemala | 3.71 | 3.64 | 3.78 | 2012 |
| Guatemala | 3.72 | 3.64 | 3.79 | 2013 |
| Guatemala | 3.75 | 3.68 | 3.82 | 2014 |
| Guatemala | 3.8 | 3.73 | 3.87 | 2015 |
| Guatemala | 3.87 | 3.8 | 3.95 | 2016 |
| Guatemala | 3.95 | 3.85 | 4.05 | 2017 |
| Guatemala | 4.02 | 3.82 | 4.23 | 2018 |
| Guatemala | 4.1 | 3.76 | 4.44 | 2019 |
| Guatemala | 4.17 | 3.66 | 4.69 | 2020 |
| Guatemala | 4.25 | 3.54 | 4.96 | 2021 |
| Guatemala | 4.32 | 3.38 | 5.27 | 2022 |
| Guatemala | 4.4 | 3.2 | 5.6 | 2023 |
| Guatemala | 4.47 | 2.99 | 5.95 | 2024 |
| Guatemala | 4.55 | 2.76 | 6.33 | 2025 |
| Guatemala | 4.62 | 2.5 | 6.74 | 2026 |
| Guatemala | 4.69 | 2.22 | 7.16 | 2027 |
| Guatemala | 4.77 | 1.91 | 7.62 | 2028 |
| Guatemala | 4.84 | 1.58 | 8.1 | 2029 |
| Guatemala | 4.91 | 1.22 | 8.6 | 2030 |
| Guinea | 2.19 | 2.1 | 2.28 | 1990 |
| Guinea | 2.18 | 2.11 | 2.26 | 1991 |
| Guinea | 2.18 | 2.11 | 2.24 | 1992 |
| Guinea | 2.17 | 2.12 | 2.23 | 1993 |
| Guinea | 2.17 | 2.12 | 2.22 | 1994 |
| Guinea | 2.17 | 2.12 | 2.22 | 1995 |
| Guinea | 2.17 | 2.12 | 2.21 | 1996 |
| Guinea | 2.17 | 2.12 | 2.22 | 1997 |
| Guinea | 2.18 | 2.13 | 2.22 | 1998 |
| Guinea | 2.18 | 2.14 | 2.23 | 1999 |
| Guinea | 2.19 | 2.15 | 2.24 | 2000 |
| Guinea | 2.21 | 2.16 | 2.25 | 2001 |
| Guinea | 2.22 | 2.18 | 2.27 | 2002 |
| Guinea | 2.24 | 2.2 | 2.29 | 2003 |
| Guinea | 2.26 | 2.22 | 2.31 | 2004 |
| Guinea | 2.29 | 2.24 | 2.33 | 2005 |
| Guinea | 2.31 | 2.27 | 2.36 | 2006 |
| Guinea | 2.34 | 2.3 | 2.39 | 2007 |
| Guinea | 2.37 | 2.33 | 2.41 | 2008 |
| Guinea | 2.4 | 2.36 | 2.44 | 2009 |
| Guinea | 2.43 | 2.39 | 2.48 | 2010 |
| Guinea | 2.46 | 2.42 | 2.51 | 2011 |
| Guinea | 2.49 | 2.45 | 2.54 | 2012 |
| Guinea | 2.52 | 2.48 | 2.57 | 2013 |
| Guinea | 2.56 | 2.5 | 2.61 | 2014 |
| Guinea | 2.59 | 2.53 | 2.64 | 2015 |
| Guinea | 2.62 | 2.55 | 2.68 | 2016 |
| Guinea | 2.65 | 2.57 | 2.73 | 2017 |
| Guinea | 2.68 | 2.58 | 2.78 | 2018 |
| Guinea | 2.71 | 2.59 | 2.83 | 2019 |
| Guinea | 2.75 | 2.6 | 2.89 | 2020 |
| Guinea | 2.78 | 2.61 | 2.95 | 2021 |
| Guinea | 2.82 | 2.61 | 3.02 | 2022 |
| Guinea | 2.85 | 2.61 | 3.09 | 2023 |
| Guinea | 2.89 | 2.61 | 3.17 | 2024 |
| Guinea | 2.92 | 2.6 | 3.24 | 2025 |
| Guinea | 2.96 | 2.59 | 3.32 | 2026 |
| Guinea | 3 | 2.58 | 3.41 | 2027 |
| Guinea | 3.03 | 2.57 | 3.49 | 2028 |
| Guinea | 3.07 | 2.56 | 3.58 | 2029 |
| Guinea | 3.1 | 2.54 | 3.67 | 2030 |
| Guinea-Bissau | 2.18 | 1.99 | 2.36 | 1990 |
| Guinea-Bissau | 2.22 | 2.06 | 2.39 | 1991 |
| Guinea-Bissau | 2.27 | 2.12 | 2.42 | 1992 |
| Guinea-Bissau | 2.32 | 2.18 | 2.46 | 1993 |
| Guinea-Bissau | 2.37 | 2.24 | 2.5 | 1994 |
| Guinea-Bissau | 2.43 | 2.3 | 2.55 | 1995 |
| Guinea-Bissau | 2.49 | 2.37 | 2.6 | 1996 |
| Guinea-Bissau | 2.55 | 2.44 | 2.67 | 1997 |
| Guinea-Bissau | 2.62 | 2.51 | 2.73 | 1998 |
| Guinea-Bissau | 2.7 | 2.58 | 2.81 | 1999 |
| Guinea-Bissau | 2.78 | 2.66 | 2.89 | 2000 |
| Guinea-Bissau | 2.86 | 2.75 | 2.97 | 2001 |
| Guinea-Bissau | 2.94 | 2.83 | 3.06 | 2002 |
| Guinea-Bissau | 3.03 | 2.92 | 3.15 | 2003 |
| Guinea-Bissau | 3.12 | 3 | 3.23 | 2004 |
| Guinea-Bissau | 3.2 | 3.09 | 3.32 | 2005 |
| Guinea-Bissau | 3.29 | 3.17 | 3.41 | 2006 |
| Guinea-Bissau | 3.37 | 3.25 | 3.49 | 2007 |
| Guinea-Bissau | 3.44 | 3.32 | 3.56 | 2008 |
| Guinea-Bissau | 3.51 | 3.39 | 3.63 | 2009 |
| Guinea-Bissau | 3.57 | 3.45 | 3.69 | 2010 |
| Guinea-Bissau | 3.63 | 3.5 | 3.75 | 2011 |
| Guinea-Bissau | 3.68 | 3.55 | 3.81 | 2012 |
| Guinea-Bissau | 3.73 | 3.59 | 3.86 | 2013 |
| Guinea-Bissau | 3.78 | 3.63 | 3.92 | 2014 |
| Guinea-Bissau | 3.83 | 3.66 | 3.99 | 2015 |
| Guinea-Bissau | 3.87 | 3.69 | 4.06 | 2016 |
| Guinea-Bissau | 3.92 | 3.71 | 4.14 | 2017 |
| Guinea-Bissau | 3.98 | 3.72 | 4.23 | 2018 |
| Guinea-Bissau | 4.03 | 3.73 | 4.34 | 2019 |
| Guinea-Bissau | 4.09 | 3.73 | 4.45 | 2020 |
| Guinea-Bissau | 4.15 | 3.72 | 4.58 | 2021 |
| Guinea-Bissau | 4.21 | 3.71 | 4.71 | 2022 |
| Guinea-Bissau | 4.28 | 3.7 | 4.86 | 2023 |
| Guinea-Bissau | 4.35 | 3.69 | 5.01 | 2024 |
| Guinea-Bissau | 4.42 | 3.67 | 5.18 | 2025 |
| Guinea-Bissau | 4.5 | 3.64 | 5.35 | 2026 |
| Guinea-Bissau | 4.58 | 3.62 | 5.53 | 2027 |
| Guinea-Bissau | 4.66 | 3.59 | 5.73 | 2028 |
| Guinea-Bissau | 4.74 | 3.55 | 5.93 | 2029 |
| Guinea-Bissau | 4.83 | 3.51 | 6.14 | 2030 |
| Guyana | 5.4 | 5.05 | 5.75 | 1990 |
| Guyana | 5.37 | 5.07 | 5.67 | 1991 |
| Guyana | 5.34 | 5.07 | 5.61 | 1992 |
| Guyana | 5.31 | 5.07 | 5.55 | 1993 |
| Guyana | 5.29 | 5.07 | 5.51 | 1994 |
| Guyana | 5.26 | 5.06 | 5.47 | 1995 |
| Guyana | 5.24 | 5.04 | 5.43 | 1996 |
| Guyana | 5.21 | 5.03 | 5.4 | 1997 |
| Guyana | 5.2 | 5.01 | 5.38 | 1998 |
| Guyana | 5.18 | 5 | 5.37 | 1999 |
| Guyana | 5.17 | 4.99 | 5.35 | 2000 |
| Guyana | 5.17 | 4.99 | 5.35 | 2001 |
| Guyana | 5.17 | 4.99 | 5.35 | 2002 |
| Guyana | 5.17 | 5 | 5.35 | 2003 |
| Guyana | 5.18 | 5.01 | 5.36 | 2004 |
| Guyana | 5.2 | 5.02 | 5.38 | 2005 |
| Guyana | 5.22 | 5.04 | 5.4 | 2006 |
| Guyana | 5.24 | 5.07 | 5.42 | 2007 |
| Guyana | 5.27 | 5.09 | 5.44 | 2008 |
| Guyana | 5.29 | 5.12 | 5.47 | 2009 |
| Guyana | 5.32 | 5.14 | 5.5 | 2010 |
| Guyana | 5.35 | 5.16 | 5.53 | 2011 |
| Guyana | 5.37 | 5.18 | 5.56 | 2012 |
| Guyana | 5.4 | 5.2 | 5.59 | 2013 |
| Guyana | 5.42 | 5.2 | 5.63 | 2014 |
| Guyana | 5.43 | 5.2 | 5.67 | 2015 |
| Guyana | 5.45 | 5.18 | 5.72 | 2016 |
| Guyana | 5.46 | 5.15 | 5.77 | 2017 |
| Guyana | 5.48 | 5.12 | 5.83 | 2018 |
| Guyana | 5.49 | 5.07 | 5.9 | 2019 |
| Guyana | 5.5 | 5.02 | 5.97 | 2020 |
| Guyana | 5.5 | 4.96 | 6.05 | 2021 |
| Guyana | 5.51 | 4.89 | 6.13 | 2022 |
| Guyana | 5.52 | 4.82 | 6.22 | 2023 |
| Guyana | 5.52 | 4.74 | 6.3 | 2024 |
| Guyana | 5.52 | 4.65 | 6.39 | 2025 |
| Guyana | 5.52 | 4.56 | 6.49 | 2026 |
| Guyana | 5.52 | 4.47 | 6.58 | 2027 |
| Guyana | 5.52 | 4.37 | 6.68 | 2028 |
| Guyana | 5.52 | 4.27 | 6.78 | 2029 |
| Guyana | 5.52 | 4.16 | 6.88 | 2030 |
| Haiti | 5.99 | 5.86 | 6.13 | 1990 |
| Haiti | 5.9 | 5.79 | 6.01 | 1991 |
| Haiti | 5.8 | 5.7 | 5.89 | 1992 |
| Haiti | 5.69 | 5.61 | 5.78 | 1993 |
| Haiti | 5.59 | 5.51 | 5.67 | 1994 |
| Haiti | 5.48 | 5.41 | 5.56 | 1995 |
| Haiti | 5.38 | 5.31 | 5.45 | 1996 |
| Haiti | 5.28 | 5.21 | 5.35 | 1997 |
| Haiti | 5.19 | 5.12 | 5.26 | 1998 |
| Haiti | 5.11 | 5.04 | 5.18 | 1999 |
| Haiti | 5.05 | 4.98 | 5.12 | 2000 |
| Haiti | 5 | 4.93 | 5.07 | 2001 |
| Haiti | 4.97 | 4.9 | 5.04 | 2002 |
| Haiti | 4.96 | 4.89 | 5.02 | 2003 |
| Haiti | 4.96 | 4.89 | 5.02 | 2004 |
| Haiti | 4.97 | 4.9 | 5.03 | 2005 |
| Haiti | 4.99 | 4.92 | 5.05 | 2006 |
| Haiti | 5.01 | 4.95 | 5.08 | 2007 |
| Haiti | 5.04 | 4.97 | 5.1 | 2008 |
| Haiti | 5.06 | 5 | 5.13 | 2009 |
| Haiti | 5.09 | 5.02 | 5.15 | 2010 |
| Haiti | 5.11 | 5.04 | 5.17 | 2011 |
| Haiti | 5.13 | 5.07 | 5.2 | 2012 |
| Haiti | 5.15 | 5.08 | 5.22 | 2013 |
| Haiti | 5.17 | 5.1 | 5.23 | 2014 |
| Haiti | 5.18 | 5.11 | 5.25 | 2015 |
| Haiti | 5.19 | 5.11 | 5.28 | 2016 |
| Haiti | 5.2 | 5.1 | 5.31 | 2017 |
| Haiti | 5.21 | 5.07 | 5.36 | 2018 |
| Haiti | 5.22 | 5.04 | 5.41 | 2019 |
| Haiti | 5.23 | 5 | 5.46 | 2020 |
| Haiti | 5.24 | 4.95 | 5.52 | 2021 |
| Haiti | 5.24 | 4.9 | 5.58 | 2022 |
| Haiti | 5.24 | 4.84 | 5.65 | 2023 |
| Haiti | 5.24 | 4.77 | 5.72 | 2024 |
| Haiti | 5.24 | 4.7 | 5.79 | 2025 |
| Haiti | 5.24 | 4.62 | 5.86 | 2026 |
| Haiti | 5.24 | 4.54 | 5.93 | 2027 |
| Haiti | 5.23 | 4.46 | 6 | 2028 |
| Haiti | 5.22 | 4.37 | 6.08 | 2029 |
| Haiti | 5.21 | 4.28 | 6.15 | 2030 |
| Honduras | 2.3 | 2.21 | 2.4 | 1990 |
| Honduras | 2.35 | 2.27 | 2.43 | 1991 |
| Honduras | 2.39 | 2.32 | 2.46 | 1992 |
| Honduras | 2.44 | 2.37 | 2.5 | 1993 |
| Honduras | 2.48 | 2.42 | 2.54 | 1994 |
| Honduras | 2.52 | 2.46 | 2.58 | 1995 |
| Honduras | 2.57 | 2.51 | 2.62 | 1996 |
| Honduras | 2.61 | 2.56 | 2.67 | 1997 |
| Honduras | 2.66 | 2.6 | 2.72 | 1998 |
| Honduras | 2.71 | 2.65 | 2.76 | 1999 |
| Honduras | 2.76 | 2.71 | 2.82 | 2000 |
| Honduras | 2.82 | 2.76 | 2.87 | 2001 |
| Honduras | 2.88 | 2.82 | 2.93 | 2002 |
| Honduras | 2.94 | 2.88 | 2.99 | 2003 |
| Honduras | 3 | 2.94 | 3.06 | 2004 |
| Honduras | 3.06 | 3.01 | 3.12 | 2005 |
| Honduras | 3.13 | 3.07 | 3.18 | 2006 |
| Honduras | 3.19 | 3.13 | 3.24 | 2007 |
| Honduras | 3.25 | 3.19 | 3.31 | 2008 |
| Honduras | 3.31 | 3.25 | 3.36 | 2009 |
| Honduras | 3.36 | 3.31 | 3.42 | 2010 |
| Honduras | 3.42 | 3.36 | 3.47 | 2011 |
| Honduras | 3.47 | 3.41 | 3.53 | 2012 |
| Honduras | 3.52 | 3.46 | 3.58 | 2013 |
| Honduras | 3.57 | 3.5 | 3.63 | 2014 |
| Honduras | 3.61 | 3.54 | 3.68 | 2015 |
| Honduras | 3.66 | 3.58 | 3.74 | 2016 |
| Honduras | 3.7 | 3.6 | 3.8 | 2017 |
| Honduras | 3.74 | 3.62 | 3.86 | 2018 |
| Honduras | 3.78 | 3.63 | 3.93 | 2019 |
| Honduras | 3.81 | 3.63 | 4 | 2020 |
| Honduras | 3.85 | 3.62 | 4.08 | 2021 |
| Honduras | 3.88 | 3.62 | 4.15 | 2022 |
| Honduras | 3.92 | 3.6 | 4.23 | 2023 |
| Honduras | 3.95 | 3.58 | 4.31 | 2024 |
| Honduras | 3.98 | 3.56 | 4.39 | 2025 |
| Honduras | 4 | 3.53 | 4.47 | 2026 |
| Honduras | 4.03 | 3.5 | 4.56 | 2027 |
| Honduras | 4.05 | 3.46 | 4.64 | 2028 |
| Honduras | 4.07 | 3.42 | 4.72 | 2029 |
| Honduras | 4.09 | 3.37 | 4.81 | 2030 |
| Hungary | 13.28 | 13.12 | 13.43 | 1990 |
| Hungary | 13.39 | 13.27 | 13.5 | 1991 |
| Hungary | 13.48 | 13.38 | 13.59 | 1992 |
| Hungary | 13.54 | 13.44 | 13.64 | 1993 |
| Hungary | 13.54 | 13.44 | 13.64 | 1994 |
| Hungary | 13.5 | 13.4 | 13.6 | 1995 |
| Hungary | 13.41 | 13.31 | 13.5 | 1996 |
| Hungary | 13.29 | 13.19 | 13.38 | 1997 |
| Hungary | 13.14 | 13.04 | 13.23 | 1998 |
| Hungary | 12.95 | 12.85 | 13.04 | 1999 |
| Hungary | 12.72 | 12.62 | 12.81 | 2000 |
| Hungary | 12.5 | 12.41 | 12.59 | 2001 |
| Hungary | 12.35 | 12.26 | 12.44 | 2002 |
| Hungary | 12.28 | 12.19 | 12.37 | 2003 |
| Hungary | 12.26 | 12.17 | 12.35 | 2004 |
| Hungary | 12.27 | 12.18 | 12.36 | 2005 |
| Hungary | 12.3 | 12.21 | 12.38 | 2006 |
| Hungary | 12.31 | 12.22 | 12.4 | 2007 |
| Hungary | 12.3 | 12.21 | 12.39 | 2008 |
| Hungary | 12.25 | 12.16 | 12.34 | 2009 |
| Hungary | 12.13 | 12.04 | 12.22 | 2010 |
| Hungary | 11.94 | 11.85 | 12.03 | 2011 |
| Hungary | 11.7 | 11.62 | 11.79 | 2012 |
| Hungary | 11.43 | 11.35 | 11.52 | 2013 |
| Hungary | 11.15 | 11.07 | 11.24 | 2014 |
| Hungary | 10.85 | 10.76 | 10.93 | 2015 |
| Hungary | 10.5 | 10.41 | 10.59 | 2016 |
| Hungary | 10.14 | 10.02 | 10.27 | 2017 |
| Hungary | 9.8 | 9.61 | 9.98 | 2018 |
| Hungary | 9.46 | 9.2 | 9.71 | 2019 |
| Hungary | 9.12 | 8.79 | 9.46 | 2020 |
| Hungary | 8.79 | 8.37 | 9.21 | 2021 |
| Hungary | 8.47 | 7.96 | 8.98 | 2022 |
| Hungary | 8.16 | 7.56 | 8.75 | 2023 |
| Hungary | 7.85 | 7.17 | 8.53 | 2024 |
| Hungary | 7.55 | 6.79 | 8.32 | 2025 |
| Hungary | 7.26 | 6.41 | 8.11 | 2026 |
| Hungary | 6.98 | 6.05 | 7.91 | 2027 |
| Hungary | 6.7 | 5.69 | 7.71 | 2028 |
| Hungary | 6.43 | 5.35 | 7.51 | 2029 |
| Hungary | 6.17 | 5.02 | 7.32 | 2030 |
| Iceland | 10.24 | 9.63 | 10.86 | 1990 |
| Iceland | 10.42 | 9.87 | 10.96 | 1991 |
| Iceland | 10.59 | 10.11 | 11.08 | 1992 |
| Iceland | 10.77 | 10.33 | 11.22 | 1993 |
| Iceland | 10.96 | 10.55 | 11.37 | 1994 |
| Iceland | 11.14 | 10.75 | 11.53 | 1995 |
| Iceland | 11.33 | 10.95 | 11.7 | 1996 |
| Iceland | 11.52 | 11.15 | 11.88 | 1997 |
| Iceland | 11.7 | 11.34 | 12.07 | 1998 |
| Iceland | 11.89 | 11.52 | 12.25 | 1999 |
| Iceland | 12.07 | 11.7 | 12.43 | 2000 |
| Iceland | 12.24 | 11.87 | 12.6 | 2001 |
| Iceland | 12.4 | 12.03 | 12.76 | 2002 |
| Iceland | 12.55 | 12.19 | 12.92 | 2003 |
| Iceland | 12.69 | 12.33 | 13.06 | 2004 |
| Iceland | 12.82 | 12.46 | 13.19 | 2005 |
| Iceland | 12.94 | 12.58 | 13.3 | 2006 |
| Iceland | 13.04 | 12.68 | 13.41 | 2007 |
| Iceland | 13.13 | 12.76 | 13.49 | 2008 |
| Iceland | 13.19 | 12.83 | 13.55 | 2009 |
| Iceland | 13.24 | 12.88 | 13.6 | 2010 |
| Iceland | 13.26 | 12.9 | 13.62 | 2011 |
| Iceland | 13.27 | 12.9 | 13.64 | 2012 |
| Iceland | 13.27 | 12.88 | 13.65 | 2013 |
| Iceland | 13.24 | 12.83 | 13.66 | 2014 |
| Iceland | 13.21 | 12.76 | 13.67 | 2015 |
| Iceland | 13.17 | 12.65 | 13.7 | 2016 |
| Iceland | 13.13 | 12.52 | 13.74 | 2017 |
| Iceland | 13.09 | 12.36 | 13.81 | 2018 |
| Iceland | 13.04 | 12.18 | 13.9 | 2019 |
| Iceland | 13 | 11.99 | 14 | 2020 |
| Iceland | 12.95 | 11.78 | 14.12 | 2021 |
| Iceland | 12.91 | 11.56 | 14.25 | 2022 |
| Iceland | 12.86 | 11.34 | 14.38 | 2023 |
| Iceland | 12.81 | 11.1 | 14.53 | 2024 |
| Iceland | 12.76 | 10.85 | 14.68 | 2025 |
| Iceland | 12.71 | 10.59 | 14.83 | 2026 |
| Iceland | 12.66 | 10.33 | 14.99 | 2027 |
| Iceland | 12.61 | 10.06 | 15.16 | 2028 |
| Iceland | 12.55 | 9.78 | 15.33 | 2029 |
| Iceland | 12.5 | 9.49 | 15.5 | 2030 |
| India | 1.21 | 1.2 | 1.22 | 1990 |
| India | 1.23 | 1.23 | 1.24 | 1991 |
| India | 1.26 | 1.25 | 1.26 | 1992 |
| India | 1.27 | 1.27 | 1.28 | 1993 |
| India | 1.29 | 1.28 | 1.3 | 1994 |
| India | 1.31 | 1.3 | 1.31 | 1995 |
| India | 1.33 | 1.33 | 1.34 | 1996 |
| India | 1.36 | 1.36 | 1.37 | 1997 |
| India | 1.39 | 1.38 | 1.4 | 1998 |
| India | 1.41 | 1.41 | 1.42 | 1999 |
| India | 1.43 | 1.42 | 1.43 | 2000 |
| India | 1.44 | 1.44 | 1.45 | 2001 |
| India | 1.45 | 1.44 | 1.45 | 2002 |
| India | 1.45 | 1.44 | 1.45 | 2003 |
| India | 1.46 | 1.45 | 1.46 | 2004 |
| India | 1.47 | 1.47 | 1.48 | 2005 |
| India | 1.49 | 1.49 | 1.5 | 2006 |
| India | 1.52 | 1.52 | 1.53 | 2007 |
| India | 1.55 | 1.55 | 1.56 | 2008 |
| India | 1.59 | 1.58 | 1.59 | 2009 |
| India | 1.63 | 1.62 | 1.63 | 2010 |
| India | 1.66 | 1.66 | 1.67 | 2011 |
| India | 1.7 | 1.7 | 1.71 | 2012 |
| India | 1.74 | 1.74 | 1.75 | 2013 |
| India | 1.79 | 1.78 | 1.79 | 2014 |
| India | 1.83 | 1.83 | 1.84 | 2015 |
| India | 1.88 | 1.87 | 1.88 | 2016 |
| India | 1.91 | 1.9 | 1.92 | 2017 |
| India | 1.94 | 1.93 | 1.96 | 2018 |
| India | 1.98 | 1.95 | 2.01 | 2019 |
| India | 2.02 | 1.97 | 2.06 | 2020 |
| India | 2.05 | 1.99 | 2.11 | 2021 |
| India | 2.09 | 2.01 | 2.17 | 2022 |
| India | 2.13 | 2.03 | 2.23 | 2023 |
| India | 2.17 | 2.04 | 2.29 | 2024 |
| India | 2.21 | 2.05 | 2.36 | 2025 |
| India | 2.25 | 2.07 | 2.43 | 2026 |
| India | 2.29 | 2.07 | 2.5 | 2027 |
| India | 2.33 | 2.08 | 2.57 | 2028 |
| India | 2.37 | 2.08 | 2.65 | 2029 |
| India | 2.41 | 2.09 | 2.73 | 2030 |
| Indonesia | 2.33 | 2.31 | 2.35 | 1990 |
| Indonesia | 2.35 | 2.34 | 2.37 | 1991 |
| Indonesia | 2.37 | 2.36 | 2.39 | 1992 |
| Indonesia | 2.4 | 2.38 | 2.41 | 1993 |
| Indonesia | 2.42 | 2.41 | 2.43 | 1994 |
| Indonesia | 2.46 | 2.44 | 2.47 | 1995 |
| Indonesia | 2.5 | 2.49 | 2.52 | 1996 |
| Indonesia | 2.56 | 2.55 | 2.58 | 1997 |
| Indonesia | 2.63 | 2.62 | 2.65 | 1998 |
| Indonesia | 2.7 | 2.69 | 2.72 | 1999 |
| Indonesia | 2.77 | 2.76 | 2.79 | 2000 |
| Indonesia | 2.84 | 2.83 | 2.86 | 2001 |
| Indonesia | 2.9 | 2.89 | 2.92 | 2002 |
| Indonesia | 2.96 | 2.95 | 2.97 | 2003 |
| Indonesia | 3.01 | 2.99 | 3.02 | 2004 |
| Indonesia | 3.05 | 3.03 | 3.06 | 2005 |
| Indonesia | 3.08 | 3.06 | 3.09 | 2006 |
| Indonesia | 3.11 | 3.09 | 3.12 | 2007 |
| Indonesia | 3.13 | 3.12 | 3.15 | 2008 |
| Indonesia | 3.15 | 3.14 | 3.17 | 2009 |
| Indonesia | 3.17 | 3.16 | 3.18 | 2010 |
| Indonesia | 3.18 | 3.17 | 3.19 | 2011 |
| Indonesia | 3.19 | 3.17 | 3.2 | 2012 |
| Indonesia | 3.19 | 3.18 | 3.21 | 2013 |
| Indonesia | 3.2 | 3.19 | 3.21 | 2014 |
| Indonesia | 3.21 | 3.19 | 3.22 | 2015 |
| Indonesia | 3.22 | 3.2 | 3.23 | 2016 |
| Indonesia | 3.22 | 3.2 | 3.24 | 2017 |
| Indonesia | 3.23 | 3.2 | 3.26 | 2018 |
| Indonesia | 3.23 | 3.18 | 3.29 | 2019 |
| Indonesia | 3.24 | 3.17 | 3.31 | 2020 |
| Indonesia | 3.24 | 3.15 | 3.34 | 2021 |
| Indonesia | 3.25 | 3.12 | 3.38 | 2022 |
| Indonesia | 3.26 | 3.1 | 3.41 | 2023 |
| Indonesia | 3.26 | 3.07 | 3.45 | 2024 |
| Indonesia | 3.26 | 3.04 | 3.49 | 2025 |
| Indonesia | 3.27 | 3.01 | 3.52 | 2026 |
| Indonesia | 3.27 | 2.97 | 3.56 | 2027 |
| Indonesia | 3.27 | 2.93 | 3.6 | 2028 |
| Indonesia | 3.27 | 2.89 | 3.65 | 2029 |
| Indonesia | 3.27 | 2.85 | 3.69 | 2030 |
| Iran | 2.33 | 2.29 | 2.37 | 1990 |
| Iran | 2.35 | 2.32 | 2.38 | 1991 |
| Iran | 2.36 | 2.34 | 2.39 | 1992 |
| Iran | 2.38 | 2.35 | 2.4 | 1993 |
| Iran | 2.39 | 2.37 | 2.42 | 1994 |
| Iran | 2.4 | 2.38 | 2.43 | 1995 |
| Iran | 2.4 | 2.38 | 2.43 | 1996 |
| Iran | 2.41 | 2.38 | 2.43 | 1997 |
| Iran | 2.42 | 2.4 | 2.44 | 1998 |
| Iran | 2.44 | 2.42 | 2.46 | 1999 |
| Iran | 2.47 | 2.45 | 2.5 | 2000 |
| Iran | 2.51 | 2.49 | 2.54 | 2001 |
| Iran | 2.56 | 2.54 | 2.59 | 2002 |
| Iran | 2.63 | 2.6 | 2.65 | 2003 |
| Iran | 2.71 | 2.68 | 2.73 | 2004 |
| Iran | 2.8 | 2.78 | 2.83 | 2005 |
| Iran | 2.91 | 2.88 | 2.93 | 2006 |
| Iran | 3.03 | 3 | 3.05 | 2007 |
| Iran | 3.15 | 3.13 | 3.18 | 2008 |
| Iran | 3.29 | 3.26 | 3.31 | 2009 |
| Iran | 3.41 | 3.39 | 3.44 | 2010 |
| Iran | 3.52 | 3.49 | 3.55 | 2011 |
| Iran | 3.61 | 3.58 | 3.63 | 2012 |
| Iran | 3.65 | 3.62 | 3.68 | 2013 |
| Iran | 3.64 | 3.62 | 3.67 | 2014 |
| Iran | 3.59 | 3.57 | 3.62 | 2015 |
| Iran | 3.51 | 3.49 | 3.54 | 2016 |
| Iran | 3.43 | 3.39 | 3.46 | 2017 |
| Iran | 3.34 | 3.27 | 3.41 | 2018 |
| Iran | 3.26 | 3.15 | 3.36 | 2019 |
| Iran | 3.18 | 3.03 | 3.32 | 2020 |
| Iran | 3.1 | 2.91 | 3.29 | 2021 |
| Iran | 3.03 | 2.79 | 3.26 | 2022 |
| Iran | 2.95 | 2.67 | 3.24 | 2023 |
| Iran | 2.89 | 2.54 | 3.23 | 2024 |
| Iran | 2.82 | 2.43 | 3.21 | 2025 |
| Iran | 2.75 | 2.31 | 3.2 | 2026 |
| Iran | 2.69 | 2.19 | 3.18 | 2027 |
| Iran | 2.62 | 2.07 | 3.17 | 2028 |
| Iran | 2.56 | 1.95 | 3.16 | 2029 |
| Iran | 2.5 | 1.84 | 3.15 | 2030 |
| Iraq | 2.93 | 2.86 | 3 | 1990 |
| Iraq | 2.99 | 2.93 | 3.05 | 1991 |
| Iraq | 3.05 | 3.01 | 3.1 | 1992 |
| Iraq | 3.12 | 3.07 | 3.16 | 1993 |
| Iraq | 3.18 | 3.14 | 3.22 | 1994 |
| Iraq | 3.24 | 3.2 | 3.28 | 1995 |
| Iraq | 3.3 | 3.26 | 3.34 | 1996 |
| Iraq | 3.36 | 3.32 | 3.4 | 1997 |
| Iraq | 3.41 | 3.37 | 3.45 | 1998 |
| Iraq | 3.47 | 3.42 | 3.51 | 1999 |
| Iraq | 3.52 | 3.47 | 3.56 | 2000 |
| Iraq | 3.56 | 3.52 | 3.6 | 2001 |
| Iraq | 3.6 | 3.56 | 3.64 | 2002 |
| Iraq | 3.63 | 3.59 | 3.67 | 2003 |
| Iraq | 3.64 | 3.6 | 3.68 | 2004 |
| Iraq | 3.65 | 3.61 | 3.69 | 2005 |
| Iraq | 3.65 | 3.61 | 3.69 | 2006 |
| Iraq | 3.65 | 3.61 | 3.69 | 2007 |
| Iraq | 3.65 | 3.61 | 3.69 | 2008 |
| Iraq | 3.66 | 3.62 | 3.69 | 2009 |
| Iraq | 3.66 | 3.62 | 3.7 | 2010 |
| Iraq | 3.67 | 3.63 | 3.71 | 2011 |
| Iraq | 3.68 | 3.64 | 3.72 | 2012 |
| Iraq | 3.68 | 3.64 | 3.72 | 2013 |
| Iraq | 3.67 | 3.63 | 3.71 | 2014 |
| Iraq | 3.65 | 3.61 | 3.69 | 2015 |
| Iraq | 3.62 | 3.58 | 3.67 | 2016 |
| Iraq | 3.59 | 3.54 | 3.65 | 2017 |
| Iraq | 3.56 | 3.49 | 3.64 | 2018 |
| Iraq | 3.53 | 3.43 | 3.63 | 2019 |
| Iraq | 3.5 | 3.37 | 3.63 | 2020 |
| Iraq | 3.47 | 3.31 | 3.63 | 2021 |
| Iraq | 3.44 | 3.24 | 3.63 | 2022 |
| Iraq | 3.4 | 3.17 | 3.63 | 2023 |
| Iraq | 3.37 | 3.1 | 3.64 | 2024 |
| Iraq | 3.34 | 3.03 | 3.64 | 2025 |
| Iraq | 3.3 | 2.96 | 3.65 | 2026 |
| Iraq | 3.27 | 2.89 | 3.65 | 2027 |
| Iraq | 3.24 | 2.81 | 3.66 | 2028 |
| Iraq | 3.2 | 2.74 | 3.67 | 2029 |
| Iraq | 3.17 | 2.66 | 3.68 | 2030 |
| Ireland | 6.19 | 6.02 | 6.36 | 1990 |
| Ireland | 6.33 | 6.19 | 6.46 | 1991 |
| Ireland | 6.46 | 6.34 | 6.58 | 1992 |
| Ireland | 6.6 | 6.49 | 6.71 | 1993 |
| Ireland | 6.75 | 6.64 | 6.85 | 1994 |
| Ireland | 6.92 | 6.81 | 7.02 | 1995 |
| Ireland | 7.1 | 7 | 7.21 | 1996 |
| Ireland | 7.32 | 7.21 | 7.42 | 1997 |
| Ireland | 7.55 | 7.44 | 7.66 | 1998 |
| Ireland | 7.79 | 7.68 | 7.9 | 1999 |
| Ireland | 8.03 | 7.92 | 8.14 | 2000 |
| Ireland | 8.26 | 8.15 | 8.37 | 2001 |
| Ireland | 8.48 | 8.37 | 8.59 | 2002 |
| Ireland | 8.66 | 8.55 | 8.78 | 2003 |
| Ireland | 8.82 | 8.71 | 8.93 | 2004 |
| Ireland | 8.94 | 8.83 | 9.06 | 2005 |
| Ireland | 9.03 | 8.92 | 9.14 | 2006 |
| Ireland | 9.08 | 8.97 | 9.19 | 2007 |
| Ireland | 9.09 | 8.98 | 9.2 | 2008 |
| Ireland | 9.07 | 8.96 | 9.18 | 2009 |
| Ireland | 9.01 | 8.9 | 9.11 | 2010 |
| Ireland | 8.93 | 8.82 | 9.03 | 2011 |
| Ireland | 8.83 | 8.73 | 8.94 | 2012 |
| Ireland | 8.73 | 8.63 | 8.84 | 2013 |
| Ireland | 8.62 | 8.52 | 8.73 | 2014 |
| Ireland | 8.51 | 8.4 | 8.62 | 2015 |
| Ireland | 8.39 | 8.27 | 8.52 | 2016 |
| Ireland | 8.29 | 8.12 | 8.45 | 2017 |
| Ireland | 8.18 | 7.96 | 8.4 | 2018 |
| Ireland | 8.08 | 7.79 | 8.37 | 2019 |
| Ireland | 7.98 | 7.61 | 8.36 | 2020 |
| Ireland | 7.89 | 7.43 | 8.36 | 2021 |
| Ireland | 7.8 | 7.24 | 8.36 | 2022 |
| Ireland | 7.72 | 7.05 | 8.38 | 2023 |
| Ireland | 7.64 | 6.87 | 8.41 | 2024 |
| Ireland | 7.56 | 6.67 | 8.44 | 2025 |
| Ireland | 7.48 | 6.48 | 8.48 | 2026 |
| Ireland | 7.4 | 6.29 | 8.52 | 2027 |
| Ireland | 7.33 | 6.09 | 8.57 | 2028 |
| Ireland | 7.26 | 5.9 | 8.62 | 2029 |
| Ireland | 7.19 | 5.7 | 8.68 | 2030 |
| Israel | 6.39 | 6.24 | 6.55 | 1990 |
| Israel | 6.51 | 6.38 | 6.64 | 1991 |
| Israel | 6.63 | 6.52 | 6.73 | 1992 |
| Israel | 6.74 | 6.64 | 6.83 | 1993 |
| Israel | 6.84 | 6.75 | 6.93 | 1994 |
| Israel | 6.93 | 6.84 | 7.02 | 1995 |
| Israel | 7.01 | 6.92 | 7.1 | 1996 |
| Israel | 7.07 | 6.98 | 7.16 | 1997 |
| Israel | 7.12 | 7.04 | 7.21 | 1998 |
| Israel | 7.17 | 7.08 | 7.25 | 1999 |
| Israel | 7.21 | 7.12 | 7.29 | 2000 |
| Israel | 7.25 | 7.16 | 7.33 | 2001 |
| Israel | 7.29 | 7.21 | 7.37 | 2002 |
| Israel | 7.33 | 7.25 | 7.42 | 2003 |
| Israel | 7.38 | 7.3 | 7.46 | 2004 |
| Israel | 7.42 | 7.34 | 7.5 | 2005 |
| Israel | 7.44 | 7.36 | 7.52 | 2006 |
| Israel | 7.44 | 7.36 | 7.53 | 2007 |
| Israel | 7.42 | 7.34 | 7.5 | 2008 |
| Israel | 7.37 | 7.29 | 7.45 | 2009 |
| Israel | 7.3 | 7.22 | 7.38 | 2010 |
| Israel | 7.22 | 7.14 | 7.29 | 2011 |
| Israel | 7.13 | 7.06 | 7.21 | 2012 |
| Israel | 7.05 | 6.97 | 7.12 | 2013 |
| Israel | 6.97 | 6.89 | 7.04 | 2014 |
| Israel | 6.89 | 6.81 | 6.97 | 2015 |
| Israel | 6.82 | 6.73 | 6.91 | 2016 |
| Israel | 6.75 | 6.63 | 6.87 | 2017 |
| Israel | 6.68 | 6.53 | 6.84 | 2018 |
| Israel | 6.62 | 6.41 | 6.82 | 2019 |
| Israel | 6.55 | 6.29 | 6.81 | 2020 |
| Israel | 6.49 | 6.16 | 6.81 | 2021 |
| Israel | 6.42 | 6.03 | 6.81 | 2022 |
| Israel | 6.36 | 5.9 | 6.81 | 2023 |
| Israel | 6.29 | 5.77 | 6.82 | 2024 |
| Israel | 6.23 | 5.63 | 6.84 | 2025 |
| Israel | 6.17 | 5.49 | 6.85 | 2026 |
| Israel | 6.11 | 5.35 | 6.87 | 2027 |
| Israel | 6.05 | 5.21 | 6.89 | 2028 |
| Israel | 5.99 | 5.06 | 6.91 | 2029 |
| Israel | 5.93 | 4.92 | 6.93 | 2030 |
| Italy | 8.81 | 8.76 | 8.87 | 1990 |
| Italy | 8.9 | 8.86 | 8.94 | 1991 |
| Italy | 8.96 | 8.93 | 9 | 1992 |
| Italy | 9.01 | 8.97 | 9.05 | 1993 |
| Italy | 9.04 | 9 | 9.08 | 1994 |
| Italy | 9.05 | 9.01 | 9.08 | 1995 |
| Italy | 9.04 | 9 | 9.08 | 1996 |
| Italy | 9.04 | 9 | 9.08 | 1997 |
| Italy | 9.06 | 9.02 | 9.1 | 1998 |
| Italy | 9.08 | 9.04 | 9.11 | 1999 |
| Italy | 9.11 | 9.08 | 9.15 | 2000 |
| Italy | 9.19 | 9.15 | 9.23 | 2001 |
| Italy | 9.31 | 9.28 | 9.35 | 2002 |
| Italy | 9.47 | 9.44 | 9.51 | 2003 |
| Italy | 9.62 | 9.58 | 9.66 | 2004 |
| Italy | 9.75 | 9.72 | 9.79 | 2005 |
| Italy | 9.86 | 9.82 | 9.9 | 2006 |
| Italy | 9.93 | 9.89 | 9.96 | 2007 |
| Italy | 9.94 | 9.9 | 9.97 | 2008 |
| Italy | 9.87 | 9.83 | 9.91 | 2009 |
| Italy | 9.75 | 9.72 | 9.79 | 2010 |
| Italy | 9.63 | 9.59 | 9.67 | 2011 |
| Italy | 9.51 | 9.47 | 9.55 | 2012 |
| Italy | 9.39 | 9.36 | 9.43 | 2013 |
| Italy | 9.28 | 9.25 | 9.32 | 2014 |
| Italy | 9.18 | 9.14 | 9.21 | 2015 |
| Italy | 9.05 | 9.01 | 9.08 | 2016 |
| Italy | 8.91 | 8.86 | 8.96 | 2017 |
| Italy | 8.79 | 8.7 | 8.87 | 2018 |
| Italy | 8.66 | 8.52 | 8.8 | 2019 |
| Italy | 8.54 | 8.34 | 8.74 | 2020 |
| Italy | 8.42 | 8.15 | 8.68 | 2021 |
| Italy | 8.3 | 7.96 | 8.64 | 2022 |
| Italy | 8.18 | 7.77 | 8.6 | 2023 |
| Italy | 8.06 | 7.57 | 8.56 | 2024 |
| Italy | 7.94 | 7.37 | 8.51 | 2025 |
| Italy | 7.82 | 7.16 | 8.47 | 2026 |
| Italy | 7.7 | 6.95 | 8.44 | 2027 |
| Italy | 7.58 | 6.75 | 8.41 | 2028 |
| Italy | 7.47 | 6.55 | 8.39 | 2029 |
| Italy | 7.36 | 6.35 | 8.37 | 2030 |
| Jamaica | 4.63 | 4.43 | 4.83 | 1990 |
| Jamaica | 4.45 | 4.29 | 4.61 | 1991 |
| Jamaica | 4.27 | 4.14 | 4.4 | 1992 |
| Jamaica | 4.1 | 3.99 | 4.22 | 1993 |
| Jamaica | 3.95 | 3.84 | 4.05 | 1994 |
| Jamaica | 3.8 | 3.71 | 3.9 | 1995 |
| Jamaica | 3.68 | 3.59 | 3.78 | 1996 |
| Jamaica | 3.58 | 3.49 | 3.67 | 1997 |
| Jamaica | 3.49 | 3.4 | 3.58 | 1998 |
| Jamaica | 3.41 | 3.32 | 3.5 | 1999 |
| Jamaica | 3.34 | 3.25 | 3.42 | 2000 |
| Jamaica | 3.27 | 3.19 | 3.36 | 2001 |
| Jamaica | 3.22 | 3.13 | 3.3 | 2002 |
| Jamaica | 3.17 | 3.09 | 3.25 | 2003 |
| Jamaica | 3.13 | 3.05 | 3.21 | 2004 |
| Jamaica | 3.11 | 3.03 | 3.19 | 2005 |
| Jamaica | 3.11 | 3.04 | 3.19 | 2006 |
| Jamaica | 3.13 | 3.05 | 3.21 | 2007 |
| Jamaica | 3.17 | 3.09 | 3.25 | 2008 |
| Jamaica | 3.21 | 3.13 | 3.29 | 2009 |
| Jamaica | 3.27 | 3.19 | 3.35 | 2010 |
| Jamaica | 3.33 | 3.25 | 3.41 | 2011 |
| Jamaica | 3.41 | 3.32 | 3.49 | 2012 |
| Jamaica | 3.48 | 3.4 | 3.57 | 2013 |
| Jamaica | 3.56 | 3.47 | 3.65 | 2014 |
| Jamaica | 3.64 | 3.54 | 3.74 | 2015 |
| Jamaica | 3.72 | 3.6 | 3.84 | 2016 |
| Jamaica | 3.8 | 3.65 | 3.95 | 2017 |
| Jamaica | 3.88 | 3.69 | 4.07 | 2018 |
| Jamaica | 3.96 | 3.72 | 4.21 | 2019 |
| Jamaica | 4.05 | 3.74 | 4.36 | 2020 |
| Jamaica | 4.14 | 3.76 | 4.52 | 2021 |
| Jamaica | 4.23 | 3.77 | 4.69 | 2022 |
| Jamaica | 4.32 | 3.78 | 4.87 | 2023 |
| Jamaica | 4.42 | 3.78 | 5.07 | 2024 |
| Jamaica | 4.52 | 3.77 | 5.27 | 2025 |
| Jamaica | 4.62 | 3.75 | 5.49 | 2026 |
| Jamaica | 4.72 | 3.73 | 5.72 | 2027 |
| Jamaica | 4.83 | 3.7 | 5.95 | 2028 |
| Jamaica | 4.93 | 3.66 | 6.2 | 2029 |
| Jamaica | 5.04 | 3.62 | 6.46 | 2030 |
| Japan | 3.52 | 3.5 | 3.55 | 1990 |
| Japan | 3.63 | 3.61 | 3.65 | 1991 |
| Japan | 3.74 | 3.73 | 3.76 | 1992 |
| Japan | 3.86 | 3.84 | 3.87 | 1993 |
| Japan | 3.97 | 3.95 | 3.98 | 1994 |
| Japan | 4.07 | 4.06 | 4.09 | 1995 |
| Japan | 4.17 | 4.16 | 4.19 | 1996 |
| Japan | 4.27 | 4.25 | 4.28 | 1997 |
| Japan | 4.35 | 4.34 | 4.37 | 1998 |
| Japan | 4.42 | 4.4 | 4.44 | 1999 |
| Japan | 4.47 | 4.46 | 4.49 | 2000 |
| Japan | 4.52 | 4.5 | 4.54 | 2001 |
| Japan | 4.58 | 4.56 | 4.59 | 2002 |
| Japan | 4.63 | 4.61 | 4.65 | 2003 |
| Japan | 4.67 | 4.66 | 4.69 | 2004 |
| Japan | 4.7 | 4.68 | 4.72 | 2005 |
| Japan | 4.71 | 4.69 | 4.73 | 2006 |
| Japan | 4.71 | 4.69 | 4.73 | 2007 |
| Japan | 4.71 | 4.69 | 4.72 | 2008 |
| Japan | 4.7 | 4.68 | 4.72 | 2009 |
| Japan | 4.7 | 4.68 | 4.71 | 2010 |
| Japan | 4.7 | 4.68 | 4.71 | 2011 |
| Japan | 4.69 | 4.67 | 4.71 | 2012 |
| Japan | 4.67 | 4.65 | 4.68 | 2013 |
| Japan | 4.63 | 4.61 | 4.65 | 2014 |
| Japan | 4.59 | 4.57 | 4.6 | 2015 |
| Japan | 4.55 | 4.53 | 4.57 | 2016 |
| Japan | 4.52 | 4.5 | 4.54 | 2017 |
| Japan | 4.49 | 4.45 | 4.53 | 2018 |
| Japan | 4.45 | 4.39 | 4.51 | 2019 |
| Japan | 4.42 | 4.33 | 4.5 | 2020 |
| Japan | 4.38 | 4.27 | 4.5 | 2021 |
| Japan | 4.36 | 4.21 | 4.5 | 2022 |
| Japan | 4.33 | 4.15 | 4.51 | 2023 |
| Japan | 4.3 | 4.09 | 4.52 | 2024 |
| Japan | 4.27 | 4.01 | 4.52 | 2025 |
| Japan | 4.23 | 3.94 | 4.53 | 2026 |
| Japan | 4.2 | 3.87 | 4.54 | 2027 |
| Japan | 4.18 | 3.8 | 4.55 | 2028 |
| Japan | 4.15 | 3.73 | 4.57 | 2029 |
| Japan | 4.13 | 3.66 | 4.59 | 2030 |
| Jordan | 1.63 | 1.54 | 1.72 | 1990 |
| Jordan | 1.67 | 1.59 | 1.75 | 1991 |
| Jordan | 1.7 | 1.63 | 1.77 | 1992 |
| Jordan | 1.74 | 1.67 | 1.8 | 1993 |
| Jordan | 1.77 | 1.71 | 1.83 | 1994 |
| Jordan | 1.81 | 1.75 | 1.87 | 1995 |
| Jordan | 1.85 | 1.79 | 1.9 | 1996 |
| Jordan | 1.89 | 1.84 | 1.94 | 1997 |
| Jordan | 1.93 | 1.88 | 1.98 | 1998 |
| Jordan | 1.98 | 1.92 | 2.03 | 1999 |
| Jordan | 2.02 | 1.97 | 2.07 | 2000 |
| Jordan | 2.07 | 2.02 | 2.12 | 2001 |
| Jordan | 2.12 | 2.07 | 2.17 | 2002 |
| Jordan | 2.17 | 2.12 | 2.23 | 2003 |
| Jordan | 2.23 | 2.18 | 2.28 | 2004 |
| Jordan | 2.28 | 2.23 | 2.33 | 2005 |
| Jordan | 2.34 | 2.28 | 2.39 | 2006 |
| Jordan | 2.39 | 2.34 | 2.44 | 2007 |
| Jordan | 2.44 | 2.39 | 2.49 | 2008 |
| Jordan | 2.49 | 2.44 | 2.55 | 2009 |
| Jordan | 2.55 | 2.49 | 2.6 | 2010 |
| Jordan | 2.6 | 2.55 | 2.65 | 2011 |
| Jordan | 2.65 | 2.6 | 2.7 | 2012 |
| Jordan | 2.7 | 2.65 | 2.76 | 2013 |
| Jordan | 2.76 | 2.7 | 2.81 | 2014 |
| Jordan | 2.81 | 2.74 | 2.87 | 2015 |
| Jordan | 2.86 | 2.79 | 2.93 | 2016 |
| Jordan | 2.91 | 2.82 | 3 | 2017 |
| Jordan | 2.96 | 2.85 | 3.07 | 2018 |
| Jordan | 3.01 | 2.87 | 3.14 | 2019 |
| Jordan | 3.06 | 2.89 | 3.22 | 2020 |
| Jordan | 3.1 | 2.9 | 3.3 | 2021 |
| Jordan | 3.15 | 2.91 | 3.39 | 2022 |
| Jordan | 3.2 | 2.92 | 3.48 | 2023 |
| Jordan | 3.24 | 2.92 | 3.57 | 2024 |
| Jordan | 3.29 | 2.91 | 3.66 | 2025 |
| Jordan | 3.33 | 2.91 | 3.76 | 2026 |
| Jordan | 3.38 | 2.89 | 3.86 | 2027 |
| Jordan | 3.42 | 2.88 | 3.96 | 2028 |
| Jordan | 3.46 | 2.86 | 4.06 | 2029 |
| Jordan | 3.5 | 2.84 | 4.17 | 2030 |
| Kazakhstan | 8.85 | 8.72 | 8.99 | 1990 |
| Kazakhstan | 9.15 | 9.05 | 9.26 | 1991 |
| Kazakhstan | 9.48 | 9.39 | 9.58 | 1992 |
| Kazakhstan | 9.84 | 9.74 | 9.94 | 1993 |
| Kazakhstan | 10.16 | 10.06 | 10.26 | 1994 |
| Kazakhstan | 10.41 | 10.31 | 10.52 | 1995 |
| Kazakhstan | 10.53 | 10.43 | 10.64 | 1996 |
| Kazakhstan | 10.55 | 10.45 | 10.66 | 1997 |
| Kazakhstan | 10.53 | 10.42 | 10.63 | 1998 |
| Kazakhstan | 10.51 | 10.4 | 10.61 | 1999 |
| Kazakhstan | 10.54 | 10.43 | 10.64 | 2000 |
| Kazakhstan | 10.59 | 10.48 | 10.69 | 2001 |
| Kazakhstan | 10.65 | 10.55 | 10.76 | 2002 |
| Kazakhstan | 10.71 | 10.61 | 10.82 | 2003 |
| Kazakhstan | 10.71 | 10.61 | 10.82 | 2004 |
| Kazakhstan | 10.62 | 10.52 | 10.73 | 2005 |
| Kazakhstan | 10.42 | 10.32 | 10.52 | 2006 |
| Kazakhstan | 10.16 | 10.06 | 10.26 | 2007 |
| Kazakhstan | 9.95 | 9.86 | 10.05 | 2008 |
| Kazakhstan | 9.77 | 9.68 | 9.87 | 2009 |
| Kazakhstan | 9.58 | 9.48 | 9.67 | 2010 |
| Kazakhstan | 9.27 | 9.18 | 9.36 | 2011 |
| Kazakhstan | 8.84 | 8.75 | 8.93 | 2012 |
| Kazakhstan | 8.36 | 8.28 | 8.44 | 2013 |
| Kazakhstan | 7.96 | 7.88 | 8.04 | 2014 |
| Kazakhstan | 7.71 | 7.63 | 7.79 | 2015 |
| Kazakhstan | 7.58 | 7.5 | 7.66 | 2016 |
| Kazakhstan | 7.47 | 7.36 | 7.58 | 2017 |
| Kazakhstan | 7.36 | 7.15 | 7.56 | 2018 |
| Kazakhstan | 7.26 | 6.94 | 7.57 | 2019 |
| Kazakhstan | 7.19 | 6.74 | 7.63 | 2020 |
| Kazakhstan | 7.12 | 6.53 | 7.71 | 2021 |
| Kazakhstan | 7.03 | 6.29 | 7.77 | 2022 |
| Kazakhstan | 6.93 | 6.03 | 7.84 | 2023 |
| Kazakhstan | 6.85 | 5.77 | 7.92 | 2024 |
| Kazakhstan | 6.78 | 5.53 | 8.03 | 2025 |
| Kazakhstan | 6.72 | 5.29 | 8.14 | 2026 |
| Kazakhstan | 6.64 | 5.02 | 8.25 | 2027 |
| Kazakhstan | 6.55 | 4.75 | 8.35 | 2028 |
| Kazakhstan | 6.47 | 4.48 | 8.47 | 2029 |
| Kazakhstan | 6.42 | 4.22 | 8.62 | 2030 |
| Kenya | 1.2 | 1.16 | 1.24 | 1990 |
| Kenya | 1.22 | 1.19 | 1.26 | 1991 |
| Kenya | 1.25 | 1.22 | 1.27 | 1992 |
| Kenya | 1.27 | 1.24 | 1.29 | 1993 |
| Kenya | 1.29 | 1.27 | 1.31 | 1994 |
| Kenya | 1.31 | 1.29 | 1.34 | 1995 |
| Kenya | 1.33 | 1.31 | 1.36 | 1996 |
| Kenya | 1.35 | 1.33 | 1.38 | 1997 |
| Kenya | 1.38 | 1.35 | 1.4 | 1998 |
| Kenya | 1.4 | 1.37 | 1.42 | 1999 |
| Kenya | 1.41 | 1.39 | 1.44 | 2000 |
| Kenya | 1.43 | 1.41 | 1.46 | 2001 |
| Kenya | 1.45 | 1.43 | 1.48 | 2002 |
| Kenya | 1.47 | 1.45 | 1.49 | 2003 |
| Kenya | 1.49 | 1.47 | 1.51 | 2004 |
| Kenya | 1.51 | 1.49 | 1.53 | 2005 |
| Kenya | 1.53 | 1.5 | 1.55 | 2006 |
| Kenya | 1.54 | 1.52 | 1.56 | 2007 |
| Kenya | 1.55 | 1.53 | 1.57 | 2008 |
| Kenya | 1.56 | 1.54 | 1.59 | 2009 |
| Kenya | 1.57 | 1.55 | 1.59 | 2010 |
| Kenya | 1.58 | 1.56 | 1.6 | 2011 |
| Kenya | 1.58 | 1.56 | 1.61 | 2012 |
| Kenya | 1.59 | 1.57 | 1.61 | 2013 |
| Kenya | 1.59 | 1.57 | 1.61 | 2014 |
| Kenya | 1.59 | 1.56 | 1.61 | 2015 |
| Kenya | 1.59 | 1.56 | 1.62 | 2016 |
| Kenya | 1.59 | 1.55 | 1.62 | 2017 |
| Kenya | 1.59 | 1.55 | 1.63 | 2018 |
| Kenya | 1.59 | 1.53 | 1.64 | 2019 |
| Kenya | 1.59 | 1.52 | 1.65 | 2020 |
| Kenya | 1.58 | 1.5 | 1.67 | 2021 |
| Kenya | 1.58 | 1.49 | 1.68 | 2022 |
| Kenya | 1.58 | 1.47 | 1.69 | 2023 |
| Kenya | 1.58 | 1.45 | 1.71 | 2024 |
| Kenya | 1.57 | 1.43 | 1.72 | 2025 |
| Kenya | 1.57 | 1.4 | 1.74 | 2026 |
| Kenya | 1.57 | 1.38 | 1.76 | 2027 |
| Kenya | 1.56 | 1.35 | 1.77 | 2028 |
| Kenya | 1.56 | 1.33 | 1.79 | 2029 |
| Kenya | 1.55 | 1.3 | 1.81 | 2030 |
| Kiribati | 0 | 0 | 0.01 | 1990 |
| Kiribati | 0.01 | 0 | 0.02 | 1991 |
| Kiribati | 0.01 | 0 | 0.02 | 1992 |
| Kiribati | 0.01 | 0 | 0.03 | 1993 |
| Kiribati | 0.02 | 0 | 0.04 | 1994 |
| Kiribati | 0.03 | 0 | 0.05 | 1995 |
| Kiribati | 0.04 | 0.01 | 0.07 | 1996 |
| Kiribati | 0.05 | 0.02 | 0.09 | 1997 |
| Kiribati | 0.07 | 0.03 | 0.12 | 1998 |
| Kiribati | 0.1 | 0.04 | 0.15 | 1999 |
| Kiribati | 0.13 | 0.06 | 0.2 | 2000 |
| Kiribati | 0.17 | 0.09 | 0.25 | 2001 |
| Kiribati | 0.23 | 0.13 | 0.32 | 2002 |
| Kiribati | 0.29 | 0.18 | 0.41 | 2003 |
| Kiribati | 0.37 | 0.24 | 0.5 | 2004 |
| Kiribati | 0.47 | 0.32 | 0.62 | 2005 |
| Kiribati | 0.58 | 0.41 | 0.74 | 2006 |
| Kiribati | 0.69 | 0.51 | 0.87 | 2007 |
| Kiribati | 0.8 | 0.6 | 1 | 2008 |
| Kiribati | 0.9 | 0.7 | 1.11 | 2009 |
| Kiribati | 1 | 0.78 | 1.21 | 2010 |
| Kiribati | 1.07 | 0.85 | 1.29 | 2011 |
| Kiribati | 1.13 | 0.9 | 1.37 | 2012 |
| Kiribati | 1.18 | 0.93 | 1.43 | 2013 |
| Kiribati | 1.2 | 0.93 | 1.47 | 2014 |
| Kiribati | 1.2 | 0.89 | 1.5 | 2015 |
| Kiribati | 1.17 | 0.83 | 1.5 | 2016 |
| Kiribati | 1.12 | 0.75 | 1.49 | 2017 |
| Kiribati | 1.06 | 0.65 | 1.46 | 2018 |
| Kiribati | 0.99 | 0.55 | 1.42 | 2019 |
| Kiribati | 0.91 | 0.45 | 1.38 | 2020 |
| Kiribati | 0.84 | 0.35 | 1.32 | 2021 |
| Kiribati | 0.76 | 0.25 | 1.26 | 2022 |
| Kiribati | 0.68 | 0.17 | 1.2 | 2023 |
| Kiribati | 0.61 | 0.09 | 1.14 | 2024 |
| Kiribati | 0.55 | 0.02 | 1.08 | 2025 |
| Kiribati | 0.49 | -0.03 | 1.01 | 2026 |
| Kiribati | 0.43 | -0.08 | 0.95 | 2027 |
| Kiribati | 0.38 | -0.12 | 0.89 | 2028 |
| Kiribati | 0.34 | -0.15 | 0.83 | 2029 |
| Kiribati | 0.29 | -0.18 | 0.77 | 2030 |
| Kuwait | 3.22 | 2.97 | 3.48 | 1990 |
| Kuwait | 3.3 | 3.07 | 3.53 | 1991 |
| Kuwait | 3.38 | 3.17 | 3.59 | 1992 |
| Kuwait | 3.46 | 3.27 | 3.65 | 1993 |
| Kuwait | 3.54 | 3.36 | 3.72 | 1994 |
| Kuwait | 3.61 | 3.43 | 3.78 | 1995 |
| Kuwait | 3.67 | 3.5 | 3.84 | 1996 |
| Kuwait | 3.72 | 3.56 | 3.89 | 1997 |
| Kuwait | 3.76 | 3.6 | 3.92 | 1998 |
| Kuwait | 3.79 | 3.63 | 3.94 | 1999 |
| Kuwait | 3.8 | 3.64 | 3.95 | 2000 |
| Kuwait | 3.79 | 3.64 | 3.94 | 2001 |
| Kuwait | 3.77 | 3.62 | 3.91 | 2002 |
| Kuwait | 3.73 | 3.59 | 3.87 | 2003 |
| Kuwait | 3.68 | 3.54 | 3.82 | 2004 |
| Kuwait | 3.62 | 3.48 | 3.75 | 2005 |
| Kuwait | 3.55 | 3.42 | 3.68 | 2006 |
| Kuwait | 3.47 | 3.35 | 3.6 | 2007 |
| Kuwait | 3.4 | 3.27 | 3.52 | 2008 |
| Kuwait | 3.32 | 3.2 | 3.44 | 2009 |
| Kuwait | 3.25 | 3.13 | 3.37 | 2010 |
| Kuwait | 3.18 | 3.06 | 3.29 | 2011 |
| Kuwait | 3.11 | 3 | 3.23 | 2012 |
| Kuwait | 3.06 | 2.94 | 3.17 | 2013 |
| Kuwait | 3 | 2.89 | 3.12 | 2014 |
| Kuwait | 2.96 | 2.84 | 3.08 | 2015 |
| Kuwait | 2.91 | 2.78 | 3.04 | 2016 |
| Kuwait | 2.87 | 2.72 | 3.02 | 2017 |
| Kuwait | 2.83 | 2.66 | 3 | 2018 |
| Kuwait | 2.79 | 2.58 | 2.99 | 2019 |
| Kuwait | 2.75 | 2.51 | 2.99 | 2020 |
| Kuwait | 2.71 | 2.43 | 2.99 | 2021 |
| Kuwait | 2.67 | 2.36 | 2.99 | 2022 |
| Kuwait | 2.64 | 2.28 | 3 | 2023 |
| Kuwait | 2.6 | 2.19 | 3.01 | 2024 |
| Kuwait | 2.57 | 2.11 | 3.02 | 2025 |
| Kuwait | 2.53 | 2.03 | 3.04 | 2026 |
| Kuwait | 2.5 | 1.95 | 3.05 | 2027 |
| Kuwait | 2.47 | 1.87 | 3.07 | 2028 |
| Kuwait | 2.44 | 1.78 | 3.09 | 2029 |
| Kuwait | 2.4 | 1.7 | 3.11 | 2030 |
| Kyrgyzstan | 3.38 | 3.22 | 3.53 | 1990 |
| Kyrgyzstan | 3.69 | 3.57 | 3.82 | 1991 |
| Kyrgyzstan | 4.03 | 3.92 | 4.13 | 1992 |
| Kyrgyzstan | 4.36 | 4.26 | 4.47 | 1993 |
| Kyrgyzstan | 4.66 | 4.55 | 4.77 | 1994 |
| Kyrgyzstan | 4.91 | 4.8 | 5.03 | 1995 |
| Kyrgyzstan | 5.09 | 4.97 | 5.21 | 1996 |
| Kyrgyzstan | 5.2 | 5.08 | 5.31 | 1997 |
| Kyrgyzstan | 5.25 | 5.14 | 5.36 | 1998 |
| Kyrgyzstan | 5.28 | 5.17 | 5.39 | 1999 |
| Kyrgyzstan | 5.29 | 5.18 | 5.4 | 2000 |
| Kyrgyzstan | 5.32 | 5.21 | 5.43 | 2001 |
| Kyrgyzstan | 5.35 | 5.24 | 5.45 | 2002 |
| Kyrgyzstan | 5.37 | 5.26 | 5.48 | 2003 |
| Kyrgyzstan | 5.38 | 5.27 | 5.49 | 2004 |
| Kyrgyzstan | 5.36 | 5.26 | 5.47 | 2005 |
| Kyrgyzstan | 5.32 | 5.22 | 5.43 | 2006 |
| Kyrgyzstan | 5.26 | 5.15 | 5.37 | 2007 |
| Kyrgyzstan | 5.19 | 5.08 | 5.29 | 2008 |
| Kyrgyzstan | 5.11 | 5 | 5.21 | 2009 |
| Kyrgyzstan | 5.04 | 4.94 | 5.14 | 2010 |
| Kyrgyzstan | 4.98 | 4.88 | 5.08 | 2011 |
| Kyrgyzstan | 4.94 | 4.84 | 5.04 | 2012 |
| Kyrgyzstan | 4.9 | 4.8 | 5 | 2013 |
| Kyrgyzstan | 4.87 | 4.77 | 4.97 | 2014 |
| Kyrgyzstan | 4.84 | 4.74 | 4.94 | 2015 |
| Kyrgyzstan | 4.79 | 4.68 | 4.91 | 2016 |
| Kyrgyzstan | 4.75 | 4.6 | 4.9 | 2017 |
| Kyrgyzstan | 4.7 | 4.49 | 4.91 | 2018 |
| Kyrgyzstan | 4.65 | 4.36 | 4.95 | 2019 |
| Kyrgyzstan | 4.61 | 4.22 | 5 | 2020 |
| Kyrgyzstan | 4.56 | 4.07 | 5.06 | 2021 |
| Kyrgyzstan | 4.52 | 3.91 | 5.13 | 2022 |
| Kyrgyzstan | 4.47 | 3.75 | 5.2 | 2023 |
| Kyrgyzstan | 4.43 | 3.58 | 5.29 | 2024 |
| Kyrgyzstan | 4.39 | 3.4 | 5.37 | 2025 |
| Kyrgyzstan | 4.35 | 3.22 | 5.47 | 2026 |
| Kyrgyzstan | 4.3 | 3.04 | 5.57 | 2027 |
| Kyrgyzstan | 4.26 | 2.86 | 5.67 | 2028 |
| Kyrgyzstan | 4.23 | 2.67 | 5.78 | 2029 |
| Kyrgyzstan | 4.19 | 2.48 | 5.89 | 2030 |
| Laos | 3.11 | 2.99 | 3.23 | 1990 |
| Laos | 3.15 | 3.05 | 3.26 | 1991 |
| Laos | 3.19 | 3.1 | 3.28 | 1992 |
| Laos | 3.24 | 3.16 | 3.32 | 1993 |
| Laos | 3.28 | 3.21 | 3.36 | 1994 |
| Laos | 3.33 | 3.25 | 3.4 | 1995 |
| Laos | 3.37 | 3.3 | 3.44 | 1996 |
| Laos | 3.42 | 3.35 | 3.49 | 1997 |
| Laos | 3.47 | 3.4 | 3.54 | 1998 |
| Laos | 3.52 | 3.46 | 3.59 | 1999 |
| Laos | 3.57 | 3.51 | 3.64 | 2000 |
| Laos | 3.62 | 3.55 | 3.69 | 2001 |
| Laos | 3.67 | 3.6 | 3.74 | 2002 |
| Laos | 3.71 | 3.64 | 3.78 | 2003 |
| Laos | 3.74 | 3.67 | 3.81 | 2004 |
| Laos | 3.77 | 3.7 | 3.84 | 2005 |
| Laos | 3.8 | 3.73 | 3.86 | 2006 |
| Laos | 3.81 | 3.74 | 3.88 | 2007 |
| Laos | 3.83 | 3.76 | 3.89 | 2008 |
| Laos | 3.83 | 3.77 | 3.9 | 2009 |
| Laos | 3.84 | 3.77 | 3.9 | 2010 |
| Laos | 3.84 | 3.77 | 3.9 | 2011 |
| Laos | 3.83 | 3.77 | 3.9 | 2012 |
| Laos | 3.83 | 3.76 | 3.9 | 2013 |
| Laos | 3.83 | 3.75 | 3.9 | 2014 |
| Laos | 3.82 | 3.74 | 3.9 | 2015 |
| Laos | 3.81 | 3.72 | 3.91 | 2016 |
| Laos | 3.81 | 3.7 | 3.92 | 2017 |
| Laos | 3.8 | 3.66 | 3.93 | 2018 |
| Laos | 3.79 | 3.63 | 3.96 | 2019 |
| Laos | 3.78 | 3.58 | 3.98 | 2020 |
| Laos | 3.77 | 3.54 | 4.01 | 2021 |
| Laos | 3.76 | 3.49 | 4.04 | 2022 |
| Laos | 3.75 | 3.43 | 4.07 | 2023 |
| Laos | 3.74 | 3.38 | 4.1 | 2024 |
| Laos | 3.73 | 3.32 | 4.13 | 2025 |
| Laos | 3.71 | 3.25 | 4.17 | 2026 |
| Laos | 3.7 | 3.19 | 4.21 | 2027 |
| Laos | 3.68 | 3.12 | 4.24 | 2028 |
| Laos | 3.67 | 3.06 | 4.28 | 2029 |
| Laos | 3.65 | 2.99 | 4.32 | 2030 |
| Latvia | 3.43 | 3.29 | 3.56 | 1990 |
| Latvia | 3.85 | 3.74 | 3.97 | 1991 |
| Latvia | 4.32 | 4.22 | 4.43 | 1992 |
| Latvia | 4.84 | 4.74 | 4.94 | 1993 |
| Latvia | 5.39 | 5.28 | 5.5 | 1994 |
| Latvia | 5.95 | 5.84 | 6.06 | 1995 |
| Latvia | 6.51 | 6.39 | 6.64 | 1996 |
| Latvia | 7.08 | 6.95 | 7.2 | 1997 |
| Latvia | 7.62 | 7.49 | 7.75 | 1998 |
| Latvia | 8.13 | 7.99 | 8.27 | 1999 |
| Latvia | 8.6 | 8.46 | 8.74 | 2000 |
| Latvia | 9.03 | 8.88 | 9.17 | 2001 |
| Latvia | 9.42 | 9.27 | 9.57 | 2002 |
| Latvia | 9.79 | 9.64 | 9.94 | 2003 |
| Latvia | 10.16 | 10 | 10.32 | 2004 |
| Latvia | 10.51 | 10.35 | 10.67 | 2005 |
| Latvia | 10.82 | 10.66 | 10.98 | 2006 |
| Latvia | 11.05 | 10.89 | 11.21 | 2007 |
| Latvia | 11.21 | 11.04 | 11.38 | 2008 |
| Latvia | 11.31 | 11.14 | 11.47 | 2009 |
| Latvia | 11.35 | 11.18 | 11.52 | 2010 |
| Latvia | 11.35 | 11.18 | 11.52 | 2011 |
| Latvia | 11.32 | 11.15 | 11.49 | 2012 |
| Latvia | 11.25 | 11.08 | 11.42 | 2013 |
| Latvia | 11.14 | 10.97 | 11.31 | 2014 |
| Latvia | 10.98 | 10.8 | 11.15 | 2015 |
| Latvia | 10.78 | 10.58 | 10.98 | 2016 |
| Latvia | 10.57 | 10.3 | 10.83 | 2017 |
| Latvia | 10.36 | 9.99 | 10.73 | 2018 |
| Latvia | 10.15 | 9.65 | 10.65 | 2019 |
| Latvia | 9.94 | 9.28 | 10.59 | 2020 |
| Latvia | 9.73 | 8.91 | 10.54 | 2021 |
| Latvia | 9.52 | 8.53 | 10.51 | 2022 |
| Latvia | 9.31 | 8.15 | 10.48 | 2023 |
| Latvia | 9.11 | 7.76 | 10.45 | 2024 |
| Latvia | 8.9 | 7.37 | 10.43 | 2025 |
| Latvia | 8.7 | 6.98 | 10.41 | 2026 |
| Latvia | 8.5 | 6.6 | 10.4 | 2027 |
| Latvia | 8.3 | 6.21 | 10.38 | 2028 |
| Latvia | 8.1 | 5.83 | 10.37 | 2029 |
| Latvia | 7.9 | 5.46 | 10.35 | 2030 |
| Lebanon | 2.47 | 2.36 | 2.59 | 1990 |
| Lebanon | 2.53 | 2.43 | 2.63 | 1991 |
| Lebanon | 2.59 | 2.5 | 2.68 | 1992 |
| Lebanon | 2.65 | 2.57 | 2.73 | 1993 |
| Lebanon | 2.71 | 2.63 | 2.78 | 1994 |
| Lebanon | 2.77 | 2.7 | 2.84 | 1995 |
| Lebanon | 2.83 | 2.76 | 2.9 | 1996 |
| Lebanon | 2.89 | 2.83 | 2.96 | 1997 |
| Lebanon | 2.96 | 2.89 | 3.03 | 1998 |
| Lebanon | 3.03 | 2.96 | 3.1 | 1999 |
| Lebanon | 3.1 | 3.03 | 3.17 | 2000 |
| Lebanon | 3.17 | 3.1 | 3.24 | 2001 |
| Lebanon | 3.24 | 3.17 | 3.3 | 2002 |
| Lebanon | 3.3 | 3.23 | 3.37 | 2003 |
| Lebanon | 3.37 | 3.3 | 3.43 | 2004 |
| Lebanon | 3.42 | 3.36 | 3.49 | 2005 |
| Lebanon | 3.48 | 3.41 | 3.55 | 2006 |
| Lebanon | 3.53 | 3.46 | 3.6 | 2007 |
| Lebanon | 3.57 | 3.5 | 3.63 | 2008 |
| Lebanon | 3.6 | 3.53 | 3.66 | 2009 |
| Lebanon | 3.62 | 3.55 | 3.68 | 2010 |
| Lebanon | 3.63 | 3.57 | 3.7 | 2011 |
| Lebanon | 3.64 | 3.57 | 3.7 | 2012 |
| Lebanon | 3.64 | 3.57 | 3.7 | 2013 |
| Lebanon | 3.63 | 3.57 | 3.7 | 2014 |
| Lebanon | 3.63 | 3.56 | 3.7 | 2015 |
| Lebanon | 3.62 | 3.54 | 3.71 | 2016 |
| Lebanon | 3.62 | 3.51 | 3.72 | 2017 |
| Lebanon | 3.61 | 3.48 | 3.74 | 2018 |
| Lebanon | 3.6 | 3.44 | 3.77 | 2019 |
| Lebanon | 3.6 | 3.4 | 3.8 | 2020 |
| Lebanon | 3.59 | 3.36 | 3.83 | 2021 |
| Lebanon | 3.59 | 3.31 | 3.87 | 2022 |
| Lebanon | 3.59 | 3.26 | 3.91 | 2023 |
| Lebanon | 3.58 | 3.21 | 3.96 | 2024 |
| Lebanon | 3.58 | 3.15 | 4.01 | 2025 |
| Lebanon | 3.58 | 3.1 | 4.06 | 2026 |
| Lebanon | 3.58 | 3.04 | 4.11 | 2027 |
| Lebanon | 3.58 | 2.98 | 4.17 | 2028 |
| Lebanon | 3.58 | 2.92 | 4.23 | 2029 |
| Lebanon | 3.58 | 2.86 | 4.3 | 2030 |
| Lesotho | 2.14 | 1.99 | 2.29 | 1990 |
| Lesotho | 2.21 | 2.07 | 2.35 | 1991 |
| Lesotho | 2.28 | 2.16 | 2.4 | 1992 |
| Lesotho | 2.36 | 2.24 | 2.47 | 1993 |
| Lesotho | 2.44 | 2.33 | 2.54 | 1994 |
| Lesotho | 2.52 | 2.42 | 2.62 | 1995 |
| Lesotho | 2.61 | 2.51 | 2.71 | 1996 |
| Lesotho | 2.7 | 2.6 | 2.8 | 1997 |
| Lesotho | 2.79 | 2.69 | 2.89 | 1998 |
| Lesotho | 2.89 | 2.79 | 2.99 | 1999 |
| Lesotho | 2.98 | 2.88 | 3.08 | 2000 |
| Lesotho | 3.08 | 2.98 | 3.18 | 2001 |
| Lesotho | 3.17 | 3.06 | 3.27 | 2002 |
| Lesotho | 3.25 | 3.15 | 3.36 | 2003 |
| Lesotho | 3.33 | 3.22 | 3.44 | 2004 |
| Lesotho | 3.4 | 3.29 | 3.51 | 2005 |
| Lesotho | 3.46 | 3.36 | 3.57 | 2006 |
| Lesotho | 3.52 | 3.41 | 3.63 | 2007 |
| Lesotho | 3.57 | 3.46 | 3.68 | 2008 |
| Lesotho | 3.61 | 3.5 | 3.72 | 2009 |
| Lesotho | 3.64 | 3.53 | 3.75 | 2010 |
| Lesotho | 3.67 | 3.56 | 3.78 | 2011 |
| Lesotho | 3.69 | 3.58 | 3.8 | 2012 |
| Lesotho | 3.71 | 3.59 | 3.83 | 2013 |
| Lesotho | 3.72 | 3.6 | 3.85 | 2014 |
| Lesotho | 3.74 | 3.6 | 3.88 | 2015 |
| Lesotho | 3.75 | 3.58 | 3.91 | 2016 |
| Lesotho | 3.76 | 3.56 | 3.95 | 2017 |
| Lesotho | 3.77 | 3.54 | 4 | 2018 |
| Lesotho | 3.78 | 3.5 | 4.06 | 2019 |
| Lesotho | 3.79 | 3.46 | 4.12 | 2020 |
| Lesotho | 3.8 | 3.41 | 4.19 | 2021 |
| Lesotho | 3.81 | 3.36 | 4.26 | 2022 |
| Lesotho | 3.82 | 3.31 | 4.34 | 2023 |
| Lesotho | 3.83 | 3.25 | 4.42 | 2024 |
| Lesotho | 3.85 | 3.18 | 4.51 | 2025 |
| Lesotho | 3.86 | 3.12 | 4.6 | 2026 |
| Lesotho | 3.87 | 3.05 | 4.69 | 2027 |
| Lesotho | 3.88 | 2.97 | 4.79 | 2028 |
| Lesotho | 3.89 | 2.9 | 4.88 | 2029 |
| Lesotho | 3.9 | 2.82 | 4.99 | 2030 |
| Liberia | 3.16 | 2.95 | 3.37 | 1990 |
| Liberia | 3.05 | 2.88 | 3.23 | 1991 |
| Liberia | 2.95 | 2.8 | 3.1 | 1992 |
| Liberia | 2.86 | 2.73 | 2.98 | 1993 |
| Liberia | 2.76 | 2.65 | 2.88 | 1994 |
| Liberia | 2.68 | 2.57 | 2.78 | 1995 |
| Liberia | 2.6 | 2.5 | 2.69 | 1996 |
| Liberia | 2.52 | 2.43 | 2.61 | 1997 |
| Liberia | 2.46 | 2.37 | 2.55 | 1998 |
| Liberia | 2.41 | 2.32 | 2.49 | 1999 |
| Liberia | 2.37 | 2.28 | 2.45 | 2000 |
| Liberia | 2.34 | 2.26 | 2.42 | 2001 |
| Liberia | 2.32 | 2.25 | 2.4 | 2002 |
| Liberia | 2.32 | 2.25 | 2.4 | 2003 |
| Liberia | 2.34 | 2.26 | 2.42 | 2004 |
| Liberia | 2.36 | 2.29 | 2.44 | 2005 |
| Liberia | 2.4 | 2.33 | 2.48 | 2006 |
| Liberia | 2.45 | 2.38 | 2.53 | 2007 |
| Liberia | 2.51 | 2.43 | 2.59 | 2008 |
| Liberia | 2.58 | 2.5 | 2.65 | 2009 |
| Liberia | 2.65 | 2.57 | 2.73 | 2010 |
| Liberia | 2.72 | 2.64 | 2.8 | 2011 |
| Liberia | 2.8 | 2.72 | 2.88 | 2012 |
| Liberia | 2.87 | 2.79 | 2.96 | 2013 |
| Liberia | 2.94 | 2.85 | 3.03 | 2014 |
| Liberia | 3.01 | 2.91 | 3.1 | 2015 |
| Liberia | 3.07 | 2.96 | 3.18 | 2016 |
| Liberia | 3.13 | 2.99 | 3.27 | 2017 |
| Liberia | 3.2 | 3.02 | 3.37 | 2018 |
| Liberia | 3.27 | 3.05 | 3.49 | 2019 |
| Liberia | 3.34 | 3.06 | 3.61 | 2020 |
| Liberia | 3.41 | 3.07 | 3.75 | 2021 |
| Liberia | 3.49 | 3.08 | 3.9 | 2022 |
| Liberia | 3.57 | 3.08 | 4.06 | 2023 |
| Liberia | 3.65 | 3.07 | 4.23 | 2024 |
| Liberia | 3.73 | 3.06 | 4.41 | 2025 |
| Liberia | 3.82 | 3.05 | 4.6 | 2026 |
| Liberia | 3.91 | 3.03 | 4.8 | 2027 |
| Liberia | 4 | 3 | 5.01 | 2028 |
| Liberia | 4.09 | 2.96 | 5.23 | 2029 |
| Liberia | 4.19 | 2.92 | 5.46 | 2030 |
| Libya | 2.73 | 2.61 | 2.86 | 1990 |
| Libya | 2.78 | 2.68 | 2.89 | 1991 |
| Libya | 2.83 | 2.74 | 2.92 | 1992 |
| Libya | 2.88 | 2.8 | 2.96 | 1993 |
| Libya | 2.94 | 2.86 | 3.01 | 1994 |
| Libya | 2.99 | 2.92 | 3.07 | 1995 |
| Libya | 3.06 | 2.99 | 3.13 | 1996 |
| Libya | 3.12 | 3.05 | 3.19 | 1997 |
| Libya | 3.19 | 3.13 | 3.26 | 1998 |
| Libya | 3.27 | 3.2 | 3.34 | 1999 |
| Libya | 3.35 | 3.28 | 3.42 | 2000 |
| Libya | 3.44 | 3.37 | 3.51 | 2001 |
| Libya | 3.53 | 3.46 | 3.6 | 2002 |
| Libya | 3.63 | 3.56 | 3.7 | 2003 |
| Libya | 3.74 | 3.67 | 3.81 | 2004 |
| Libya | 3.86 | 3.78 | 3.93 | 2005 |
| Libya | 3.98 | 3.91 | 4.05 | 2006 |
| Libya | 4.11 | 4.03 | 4.18 | 2007 |
| Libya | 4.24 | 4.16 | 4.31 | 2008 |
| Libya | 4.37 | 4.29 | 4.45 | 2009 |
| Libya | 4.49 | 4.42 | 4.57 | 2010 |
| Libya | 4.61 | 4.53 | 4.69 | 2011 |
| Libya | 4.72 | 4.64 | 4.8 | 2012 |
| Libya | 4.82 | 4.73 | 4.9 | 2013 |
| Libya | 4.91 | 4.82 | 4.99 | 2014 |
| Libya | 4.99 | 4.89 | 5.08 | 2015 |
| Libya | 5.07 | 4.95 | 5.18 | 2016 |
| Libya | 5.15 | 5 | 5.29 | 2017 |
| Libya | 5.22 | 5.04 | 5.4 | 2018 |
| Libya | 5.31 | 5.08 | 5.54 | 2019 |
| Libya | 5.39 | 5.1 | 5.68 | 2020 |
| Libya | 5.47 | 5.12 | 5.83 | 2021 |
| Libya | 5.56 | 5.13 | 5.99 | 2022 |
| Libya | 5.65 | 5.14 | 6.16 | 2023 |
| Libya | 5.74 | 5.14 | 6.34 | 2024 |
| Libya | 5.83 | 5.14 | 6.53 | 2025 |
| Libya | 5.93 | 5.13 | 6.72 | 2026 |
| Libya | 6.02 | 5.12 | 6.93 | 2027 |
| Libya | 6.12 | 5.1 | 7.14 | 2028 |
| Libya | 6.22 | 5.08 | 7.37 | 2029 |
| Libya | 6.33 | 5.05 | 7.6 | 2030 |
| Lithuania | 4.21 | 4.06 | 4.35 | 1990 |
| Lithuania | 4.6 | 4.48 | 4.71 | 1991 |
| Lithuania | 5.01 | 4.91 | 5.11 | 1992 |
| Lithuania | 5.43 | 5.32 | 5.53 | 1993 |
| Lithuania | 5.81 | 5.7 | 5.91 | 1994 |
| Lithuania | 6.16 | 6.05 | 6.27 | 1995 |
| Lithuania | 6.53 | 6.41 | 6.64 | 1996 |
| Lithuania | 6.97 | 6.85 | 7.09 | 1997 |
| Lithuania | 7.51 | 7.39 | 7.63 | 1998 |
| Lithuania | 8.11 | 7.98 | 8.24 | 1999 |
| Lithuania | 8.73 | 8.6 | 8.87 | 2000 |
| Lithuania | 9.35 | 9.21 | 9.49 | 2001 |
| Lithuania | 9.94 | 9.8 | 10.08 | 2002 |
| Lithuania | 10.53 | 10.38 | 10.68 | 2003 |
| Lithuania | 11.14 | 10.98 | 11.29 | 2004 |
| Lithuania | 11.73 | 11.57 | 11.89 | 2005 |
| Lithuania | 12.24 | 12.07 | 12.41 | 2006 |
| Lithuania | 12.56 | 12.39 | 12.73 | 2007 |
| Lithuania | 12.65 | 12.48 | 12.82 | 2008 |
| Lithuania | 12.56 | 12.4 | 12.73 | 2009 |
| Lithuania | 12.41 | 12.24 | 12.58 | 2010 |
| Lithuania | 12.24 | 12.07 | 12.4 | 2011 |
| Lithuania | 12.07 | 11.91 | 12.24 | 2012 |
| Lithuania | 11.91 | 11.75 | 12.08 | 2013 |
| Lithuania | 11.75 | 11.59 | 11.92 | 2014 |
| Lithuania | 11.6 | 11.43 | 11.76 | 2015 |
| Lithuania | 11.44 | 11.26 | 11.62 | 2016 |
| Lithuania | 11.27 | 11.03 | 11.52 | 2017 |
| Lithuania | 11.1 | 10.72 | 11.47 | 2018 |
| Lithuania | 10.92 | 10.37 | 11.46 | 2019 |
| Lithuania | 10.74 | 10 | 11.48 | 2020 |
| Lithuania | 10.56 | 9.6 | 11.52 | 2021 |
| Lithuania | 10.38 | 9.18 | 11.57 | 2022 |
| Lithuania | 10.19 | 8.75 | 11.63 | 2023 |
| Lithuania | 10.01 | 8.31 | 11.7 | 2024 |
| Lithuania | 9.82 | 7.87 | 11.77 | 2025 |
| Lithuania | 9.63 | 7.42 | 11.84 | 2026 |
| Lithuania | 9.44 | 6.97 | 11.92 | 2027 |
| Lithuania | 9.25 | 6.51 | 11.99 | 2028 |
| Lithuania | 9.06 | 6.05 | 12.06 | 2029 |
| Lithuania | 8.87 | 5.6 | 12.14 | 2030 |
| Luxembourg | 4.17 | 3.89 | 4.44 | 1990 |
| Luxembourg | 4.17 | 3.92 | 4.42 | 1991 |
| Luxembourg | 4.17 | 3.95 | 4.39 | 1992 |
| Luxembourg | 4.18 | 3.98 | 4.38 | 1993 |
| Luxembourg | 4.18 | 4 | 4.36 | 1994 |
| Luxembourg | 4.18 | 4.01 | 4.35 | 1995 |
| Luxembourg | 4.19 | 4.02 | 4.35 | 1996 |
| Luxembourg | 4.19 | 4.03 | 4.35 | 1997 |
| Luxembourg | 4.19 | 4.03 | 4.34 | 1998 |
| Luxembourg | 4.19 | 4.03 | 4.34 | 1999 |
| Luxembourg | 4.19 | 4.04 | 4.34 | 2000 |
| Luxembourg | 4.18 | 4.03 | 4.33 | 2001 |
| Luxembourg | 4.18 | 4.03 | 4.33 | 2002 |
| Luxembourg | 4.17 | 4.03 | 4.32 | 2003 |
| Luxembourg | 4.17 | 4.02 | 4.31 | 2004 |
| Luxembourg | 4.16 | 4.01 | 4.3 | 2005 |
| Luxembourg | 4.15 | 4.01 | 4.29 | 2006 |
| Luxembourg | 4.14 | 4 | 4.28 | 2007 |
| Luxembourg | 4.13 | 3.99 | 4.27 | 2008 |
| Luxembourg | 4.12 | 3.98 | 4.26 | 2009 |
| Luxembourg | 4.11 | 3.97 | 4.25 | 2010 |
| Luxembourg | 4.1 | 3.96 | 4.24 | 2011 |
| Luxembourg | 4.09 | 3.95 | 4.24 | 2012 |
| Luxembourg | 4.08 | 3.93 | 4.24 | 2013 |
| Luxembourg | 4.07 | 3.91 | 4.24 | 2014 |
| Luxembourg | 4.06 | 3.88 | 4.25 | 2015 |
| Luxembourg | 4.05 | 3.85 | 4.26 | 2016 |
| Luxembourg | 4.04 | 3.8 | 4.27 | 2017 |
| Luxembourg | 4.02 | 3.75 | 4.29 | 2018 |
| Luxembourg | 4 | 3.7 | 4.31 | 2019 |
| Luxembourg | 3.98 | 3.63 | 4.34 | 2020 |
| Luxembourg | 3.96 | 3.56 | 4.36 | 2021 |
| Luxembourg | 3.94 | 3.49 | 4.39 | 2022 |
| Luxembourg | 3.91 | 3.41 | 4.42 | 2023 |
| Luxembourg | 3.88 | 3.32 | 4.44 | 2024 |
| Luxembourg | 3.85 | 3.24 | 4.47 | 2025 |
| Luxembourg | 3.82 | 3.15 | 4.5 | 2026 |
| Luxembourg | 3.79 | 3.06 | 4.52 | 2027 |
| Luxembourg | 3.75 | 2.96 | 4.55 | 2028 |
| Luxembourg | 3.72 | 2.86 | 4.57 | 2029 |
| Luxembourg | 3.68 | 2.76 | 4.6 | 2030 |
| Macedonia | 2.07 | 1.94 | 2.2 | 1990 |
| Macedonia | 2.1 | 1.99 | 2.21 | 1991 |
| Macedonia | 2.13 | 2.04 | 2.23 | 1992 |
| Macedonia | 2.17 | 2.09 | 2.25 | 1993 |
| Macedonia | 2.21 | 2.14 | 2.29 | 1994 |
| Macedonia | 2.27 | 2.19 | 2.34 | 1995 |
| Macedonia | 2.33 | 2.25 | 2.41 | 1996 |
| Macedonia | 2.4 | 2.32 | 2.48 | 1997 |
| Macedonia | 2.49 | 2.41 | 2.58 | 1998 |
| Macedonia | 2.61 | 2.52 | 2.69 | 1999 |
| Macedonia | 2.74 | 2.66 | 2.83 | 2000 |
| Macedonia | 2.91 | 2.82 | 3 | 2001 |
| Macedonia | 3.1 | 3.01 | 3.19 | 2002 |
| Macedonia | 3.32 | 3.22 | 3.41 | 2003 |
| Macedonia | 3.55 | 3.45 | 3.64 | 2004 |
| Macedonia | 3.79 | 3.69 | 3.89 | 2005 |
| Macedonia | 4.01 | 3.91 | 4.12 | 2006 |
| Macedonia | 4.22 | 4.11 | 4.33 | 2007 |
| Macedonia | 4.39 | 4.28 | 4.5 | 2008 |
| Macedonia | 4.52 | 4.41 | 4.64 | 2009 |
| Macedonia | 4.61 | 4.5 | 4.73 | 2010 |
| Macedonia | 4.68 | 4.56 | 4.79 | 2011 |
| Macedonia | 4.72 | 4.6 | 4.83 | 2012 |
| Macedonia | 4.75 | 4.63 | 4.86 | 2013 |
| Macedonia | 4.77 | 4.66 | 4.89 | 2014 |
| Macedonia | 4.8 | 4.68 | 4.93 | 2015 |
| Macedonia | 4.83 | 4.68 | 4.98 | 2016 |
| Macedonia | 4.86 | 4.67 | 5.05 | 2017 |
| Macedonia | 4.89 | 4.63 | 5.15 | 2018 |
| Macedonia | 4.92 | 4.57 | 5.27 | 2019 |
| Macedonia | 4.95 | 4.49 | 5.41 | 2020 |
| Macedonia | 4.98 | 4.41 | 5.56 | 2021 |
| Macedonia | 5.01 | 4.31 | 5.72 | 2022 |
| Macedonia | 5.05 | 4.2 | 5.9 | 2023 |
| Macedonia | 5.08 | 4.08 | 6.09 | 2024 |
| Macedonia | 5.12 | 3.95 | 6.29 | 2025 |
| Macedonia | 5.16 | 3.82 | 6.5 | 2026 |
| Macedonia | 5.2 | 3.67 | 6.73 | 2027 |
| Macedonia | 5.24 | 3.52 | 6.97 | 2028 |
| Macedonia | 5.28 | 3.35 | 7.22 | 2029 |
| Macedonia | 5.33 | 3.18 | 7.48 | 2030 |
| Madagascar | 1.83 | 1.77 | 1.88 | 1990 |
| Madagascar | 1.8 | 1.75 | 1.85 | 1991 |
| Madagascar | 1.78 | 1.74 | 1.82 | 1992 |
| Madagascar | 1.76 | 1.72 | 1.79 | 1993 |
| Madagascar | 1.74 | 1.7 | 1.77 | 1994 |
| Madagascar | 1.72 | 1.68 | 1.75 | 1995 |
| Madagascar | 1.7 | 1.66 | 1.73 | 1996 |
| Madagascar | 1.68 | 1.65 | 1.71 | 1997 |
| Madagascar | 1.66 | 1.63 | 1.69 | 1998 |
| Madagascar | 1.65 | 1.62 | 1.67 | 1999 |
| Madagascar | 1.63 | 1.6 | 1.66 | 2000 |
| Madagascar | 1.62 | 1.59 | 1.65 | 2001 |
| Madagascar | 1.61 | 1.59 | 1.64 | 2002 |
| Madagascar | 1.61 | 1.58 | 1.64 | 2003 |
| Madagascar | 1.61 | 1.58 | 1.64 | 2004 |
| Madagascar | 1.61 | 1.59 | 1.64 | 2005 |
| Madagascar | 1.62 | 1.6 | 1.65 | 2006 |
| Madagascar | 1.63 | 1.61 | 1.66 | 2007 |
| Madagascar | 1.64 | 1.62 | 1.67 | 2008 |
| Madagascar | 1.66 | 1.63 | 1.68 | 2009 |
| Madagascar | 1.67 | 1.65 | 1.7 | 2010 |
| Madagascar | 1.69 | 1.66 | 1.71 | 2011 |
| Madagascar | 1.7 | 1.67 | 1.73 | 2012 |
| Madagascar | 1.71 | 1.69 | 1.74 | 2013 |
| Madagascar | 1.73 | 1.7 | 1.76 | 2014 |
| Madagascar | 1.74 | 1.71 | 1.77 | 2015 |
| Madagascar | 1.75 | 1.72 | 1.79 | 2016 |
| Madagascar | 1.76 | 1.72 | 1.81 | 2017 |
| Madagascar | 1.77 | 1.72 | 1.83 | 2018 |
| Madagascar | 1.79 | 1.72 | 1.85 | 2019 |
| Madagascar | 1.79 | 1.71 | 1.88 | 2020 |
| Madagascar | 1.8 | 1.71 | 1.9 | 2021 |
| Madagascar | 1.81 | 1.69 | 1.93 | 2022 |
| Madagascar | 1.82 | 1.68 | 1.96 | 2023 |
| Madagascar | 1.83 | 1.67 | 1.99 | 2024 |
| Madagascar | 1.84 | 1.65 | 2.02 | 2025 |
| Madagascar | 1.84 | 1.63 | 2.05 | 2026 |
| Madagascar | 1.85 | 1.61 | 2.08 | 2027 |
| Madagascar | 1.85 | 1.59 | 2.11 | 2028 |
| Madagascar | 1.86 | 1.57 | 2.14 | 2029 |
| Madagascar | 1.86 | 1.54 | 2.18 | 2030 |
| Malawi | 2.9 | 2.81 | 2.99 | 1990 |
| Malawi | 2.94 | 2.86 | 3.01 | 1991 |
| Malawi | 2.97 | 2.9 | 3.03 | 1992 |
| Malawi | 2.99 | 2.93 | 3.05 | 1993 |
| Malawi | 3 | 2.94 | 3.05 | 1994 |
| Malawi | 2.99 | 2.93 | 3.04 | 1995 |
| Malawi | 2.96 | 2.91 | 3.01 | 1996 |
| Malawi | 2.92 | 2.87 | 2.97 | 1997 |
| Malawi | 2.87 | 2.83 | 2.92 | 1998 |
| Malawi | 2.83 | 2.78 | 2.87 | 1999 |
| Malawi | 2.78 | 2.73 | 2.83 | 2000 |
| Malawi | 2.73 | 2.69 | 2.78 | 2001 |
| Malawi | 2.69 | 2.65 | 2.74 | 2002 |
| Malawi | 2.66 | 2.61 | 2.7 | 2003 |
| Malawi | 2.63 | 2.59 | 2.68 | 2004 |
| Malawi | 2.62 | 2.57 | 2.66 | 2005 |
| Malawi | 2.61 | 2.57 | 2.66 | 2006 |
| Malawi | 2.62 | 2.58 | 2.66 | 2007 |
| Malawi | 2.64 | 2.59 | 2.68 | 2008 |
| Malawi | 2.66 | 2.62 | 2.71 | 2009 |
| Malawi | 2.7 | 2.66 | 2.74 | 2010 |
| Malawi | 2.73 | 2.69 | 2.78 | 2011 |
| Malawi | 2.77 | 2.72 | 2.81 | 2012 |
| Malawi | 2.79 | 2.75 | 2.84 | 2013 |
| Malawi | 2.81 | 2.76 | 2.86 | 2014 |
| Malawi | 2.82 | 2.77 | 2.87 | 2015 |
| Malawi | 2.83 | 2.77 | 2.88 | 2016 |
| Malawi | 2.83 | 2.76 | 2.9 | 2017 |
| Malawi | 2.83 | 2.74 | 2.93 | 2018 |
| Malawi | 2.83 | 2.71 | 2.96 | 2019 |
| Malawi | 2.84 | 2.68 | 2.99 | 2020 |
| Malawi | 2.84 | 2.64 | 3.03 | 2021 |
| Malawi | 2.84 | 2.6 | 3.08 | 2022 |
| Malawi | 2.84 | 2.56 | 3.12 | 2023 |
| Malawi | 2.84 | 2.51 | 3.17 | 2024 |
| Malawi | 2.84 | 2.46 | 3.22 | 2025 |
| Malawi | 2.84 | 2.41 | 3.27 | 2026 |
| Malawi | 2.84 | 2.35 | 3.33 | 2027 |
| Malawi | 2.84 | 2.29 | 3.38 | 2028 |
| Malawi | 2.83 | 2.23 | 3.44 | 2029 |
| Malawi | 2.83 | 2.17 | 3.49 | 2030 |
| Malaysia | 2.32 | 2.27 | 2.38 | 1990 |
| Malaysia | 2.36 | 2.31 | 2.41 | 1991 |
| Malaysia | 2.4 | 2.36 | 2.44 | 1992 |
| Malaysia | 2.44 | 2.4 | 2.47 | 1993 |
| Malaysia | 2.48 | 2.44 | 2.51 | 1994 |
| Malaysia | 2.52 | 2.49 | 2.56 | 1995 |
| Malaysia | 2.57 | 2.54 | 2.6 | 1996 |
| Malaysia | 2.62 | 2.58 | 2.65 | 1997 |
| Malaysia | 2.67 | 2.63 | 2.7 | 1998 |
| Malaysia | 2.72 | 2.69 | 2.75 | 1999 |
| Malaysia | 2.77 | 2.74 | 2.8 | 2000 |
| Malaysia | 2.82 | 2.79 | 2.85 | 2001 |
| Malaysia | 2.87 | 2.83 | 2.9 | 2002 |
| Malaysia | 2.91 | 2.88 | 2.94 | 2003 |
| Malaysia | 2.96 | 2.92 | 2.99 | 2004 |
| Malaysia | 3 | 2.97 | 3.03 | 2005 |
| Malaysia | 3.04 | 3.01 | 3.07 | 2006 |
| Malaysia | 3.08 | 3.05 | 3.11 | 2007 |
| Malaysia | 3.11 | 3.08 | 3.15 | 2008 |
| Malaysia | 3.15 | 3.12 | 3.18 | 2009 |
| Malaysia | 3.18 | 3.14 | 3.21 | 2010 |
| Malaysia | 3.2 | 3.17 | 3.23 | 2011 |
| Malaysia | 3.22 | 3.19 | 3.25 | 2012 |
| Malaysia | 3.24 | 3.21 | 3.27 | 2013 |
| Malaysia | 3.26 | 3.23 | 3.29 | 2014 |
| Malaysia | 3.27 | 3.23 | 3.3 | 2015 |
| Malaysia | 3.27 | 3.23 | 3.31 | 2016 |
| Malaysia | 3.27 | 3.22 | 3.32 | 2017 |
| Malaysia | 3.27 | 3.2 | 3.34 | 2018 |
| Malaysia | 3.27 | 3.18 | 3.36 | 2019 |
| Malaysia | 3.27 | 3.16 | 3.39 | 2020 |
| Malaysia | 3.27 | 3.13 | 3.41 | 2021 |
| Malaysia | 3.27 | 3.1 | 3.44 | 2022 |
| Malaysia | 3.27 | 3.07 | 3.48 | 2023 |
| Malaysia | 3.27 | 3.03 | 3.51 | 2024 |
| Malaysia | 3.27 | 2.99 | 3.54 | 2025 |
| Malaysia | 3.27 | 2.95 | 3.58 | 2026 |
| Malaysia | 3.26 | 2.91 | 3.61 | 2027 |
| Malaysia | 3.26 | 2.86 | 3.65 | 2028 |
| Malaysia | 3.25 | 2.82 | 3.69 | 2029 |
| Malaysia | 3.25 | 2.77 | 3.73 | 2030 |
| Mali | 1.79 | 1.73 | 1.85 | 1990 |
| Mali | 1.81 | 1.75 | 1.86 | 1991 |
| Mali | 1.83 | 1.78 | 1.87 | 1992 |
| Mali | 1.85 | 1.8 | 1.89 | 1993 |
| Mali | 1.86 | 1.82 | 1.9 | 1994 |
| Mali | 1.88 | 1.84 | 1.92 | 1995 |
| Mali | 1.9 | 1.86 | 1.94 | 1996 |
| Mali | 1.92 | 1.89 | 1.96 | 1997 |
| Mali | 1.95 | 1.91 | 1.98 | 1998 |
| Mali | 1.97 | 1.94 | 2.01 | 1999 |
| Mali | 2 | 1.96 | 2.04 | 2000 |
| Mali | 2.03 | 2 | 2.07 | 2001 |
| Mali | 2.07 | 2.03 | 2.1 | 2002 |
| Mali | 2.1 | 2.07 | 2.14 | 2003 |
| Mali | 2.14 | 2.1 | 2.18 | 2004 |
| Mali | 2.18 | 2.15 | 2.22 | 2005 |
| Mali | 2.23 | 2.19 | 2.27 | 2006 |
| Mali | 2.27 | 2.24 | 2.31 | 2007 |
| Mali | 2.32 | 2.28 | 2.36 | 2008 |
| Mali | 2.37 | 2.33 | 2.41 | 2009 |
| Mali | 2.43 | 2.39 | 2.47 | 2010 |
| Mali | 2.48 | 2.44 | 2.52 | 2011 |
| Mali | 2.54 | 2.5 | 2.58 | 2012 |
| Mali | 2.6 | 2.56 | 2.65 | 2013 |
| Mali | 2.67 | 2.63 | 2.72 | 2014 |
| Mali | 2.75 | 2.7 | 2.8 | 2015 |
| Mali | 2.83 | 2.77 | 2.89 | 2016 |
| Mali | 2.91 | 2.84 | 2.98 | 2017 |
| Mali | 2.99 | 2.9 | 3.09 | 2018 |
| Mali | 3.07 | 2.95 | 3.19 | 2019 |
| Mali | 3.16 | 3 | 3.31 | 2020 |
| Mali | 3.24 | 3.05 | 3.43 | 2021 |
| Mali | 3.33 | 3.09 | 3.56 | 2022 |
| Mali | 3.41 | 3.13 | 3.69 | 2023 |
| Mali | 3.5 | 3.17 | 3.83 | 2024 |
| Mali | 3.59 | 3.2 | 3.97 | 2025 |
| Mali | 3.68 | 3.23 | 4.12 | 2026 |
| Mali | 3.77 | 3.26 | 4.27 | 2027 |
| Mali | 3.86 | 3.28 | 4.43 | 2028 |
| Mali | 3.95 | 3.3 | 4.6 | 2029 |
| Mali | 4.04 | 3.31 | 4.77 | 2030 |
| Malta | 4.72 | 4.4 | 5.04 | 1990 |
| Malta | 4.78 | 4.49 | 5.06 | 1991 |
| Malta | 4.83 | 4.58 | 5.09 | 1992 |
| Malta | 4.89 | 4.66 | 5.13 | 1993 |
| Malta | 4.96 | 4.74 | 5.17 | 1994 |
| Malta | 5.02 | 4.82 | 5.23 | 1995 |
| Malta | 5.09 | 4.9 | 5.29 | 1996 |
| Malta | 5.17 | 4.98 | 5.36 | 1997 |
| Malta | 5.26 | 5.07 | 5.44 | 1998 |
| Malta | 5.34 | 5.16 | 5.53 | 1999 |
| Malta | 5.44 | 5.26 | 5.62 | 2000 |
| Malta | 5.54 | 5.36 | 5.73 | 2001 |
| Malta | 5.64 | 5.46 | 5.83 | 2002 |
| Malta | 5.75 | 5.57 | 5.94 | 2003 |
| Malta | 5.86 | 5.67 | 6.05 | 2004 |
| Malta | 5.97 | 5.78 | 6.16 | 2005 |
| Malta | 6.08 | 5.89 | 6.27 | 2006 |
| Malta | 6.19 | 6 | 6.38 | 2007 |
| Malta | 6.29 | 6.1 | 6.48 | 2008 |
| Malta | 6.39 | 6.2 | 6.58 | 2009 |
| Malta | 6.48 | 6.29 | 6.68 | 2010 |
| Malta | 6.58 | 6.38 | 6.78 | 2011 |
| Malta | 6.67 | 6.47 | 6.87 | 2012 |
| Malta | 6.76 | 6.55 | 6.98 | 2013 |
| Malta | 6.86 | 6.62 | 7.09 | 2014 |
| Malta | 6.96 | 6.69 | 7.22 | 2015 |
| Malta | 7.06 | 6.75 | 7.37 | 2016 |
| Malta | 7.17 | 6.8 | 7.53 | 2017 |
| Malta | 7.28 | 6.84 | 7.71 | 2018 |
| Malta | 7.4 | 6.88 | 7.92 | 2019 |
| Malta | 7.52 | 6.9 | 8.13 | 2020 |
| Malta | 7.65 | 6.92 | 8.37 | 2021 |
| Malta | 7.78 | 6.93 | 8.63 | 2022 |
| Malta | 7.92 | 6.94 | 8.9 | 2023 |
| Malta | 8.07 | 6.95 | 9.19 | 2024 |
| Malta | 8.22 | 6.94 | 9.5 | 2025 |
| Malta | 8.38 | 6.93 | 9.82 | 2026 |
| Malta | 8.54 | 6.92 | 10.17 | 2027 |
| Malta | 8.72 | 6.9 | 10.54 | 2028 |
| Malta | 8.9 | 6.87 | 10.92 | 2029 |
| Malta | 9.08 | 6.83 | 11.33 | 2030 |
| Mauritania | 2.71 | 2.55 | 2.87 | 1990 |
| Mauritania | 2.74 | 2.59 | 2.88 | 1991 |
| Mauritania | 2.77 | 2.64 | 2.9 | 1992 |
| Mauritania | 2.8 | 2.68 | 2.92 | 1993 |
| Mauritania | 2.83 | 2.72 | 2.94 | 1994 |
| Mauritania | 2.86 | 2.76 | 2.97 | 1995 |
| Mauritania | 2.9 | 2.8 | 3 | 1996 |
| Mauritania | 2.94 | 2.84 | 3.03 | 1997 |
| Mauritania | 2.98 | 2.88 | 3.07 | 1998 |
| Mauritania | 3.02 | 2.93 | 3.11 | 1999 |
| Mauritania | 3.06 | 2.97 | 3.15 | 2000 |
| Mauritania | 3.1 | 3.01 | 3.19 | 2001 |
| Mauritania | 3.14 | 3.06 | 3.23 | 2002 |
| Mauritania | 3.19 | 3.1 | 3.27 | 2003 |
| Mauritania | 3.23 | 3.14 | 3.32 | 2004 |
| Mauritania | 3.27 | 3.18 | 3.36 | 2005 |
| Mauritania | 3.31 | 3.22 | 3.39 | 2006 |
| Mauritania | 3.34 | 3.26 | 3.43 | 2007 |
| Mauritania | 3.38 | 3.29 | 3.46 | 2008 |
| Mauritania | 3.41 | 3.32 | 3.49 | 2009 |
| Mauritania | 3.43 | 3.35 | 3.52 | 2010 |
| Mauritania | 3.46 | 3.37 | 3.54 | 2011 |
| Mauritania | 3.48 | 3.39 | 3.57 | 2012 |
| Mauritania | 3.5 | 3.4 | 3.59 | 2013 |
| Mauritania | 3.51 | 3.42 | 3.61 | 2014 |
| Mauritania | 3.53 | 3.42 | 3.63 | 2015 |
| Mauritania | 3.54 | 3.42 | 3.66 | 2016 |
| Mauritania | 3.55 | 3.41 | 3.7 | 2017 |
| Mauritania | 3.57 | 3.4 | 3.73 | 2018 |
| Mauritania | 3.58 | 3.38 | 3.78 | 2019 |
| Mauritania | 3.59 | 3.35 | 3.82 | 2020 |
| Mauritania | 3.6 | 3.32 | 3.88 | 2021 |
| Mauritania | 3.61 | 3.29 | 3.93 | 2022 |
| Mauritania | 3.62 | 3.25 | 3.99 | 2023 |
| Mauritania | 3.63 | 3.21 | 4.05 | 2024 |
| Mauritania | 3.64 | 3.17 | 4.11 | 2025 |
| Mauritania | 3.65 | 3.13 | 4.18 | 2026 |
| Mauritania | 3.66 | 3.08 | 4.24 | 2027 |
| Mauritania | 3.67 | 3.03 | 4.32 | 2028 |
| Mauritania | 3.68 | 2.98 | 4.39 | 2029 |
| Mauritania | 3.7 | 2.93 | 4.47 | 2030 |
| Mauritius | 2.83 | 2.64 | 3.02 | 1990 |
| Mauritius | 2.87 | 2.7 | 3.04 | 1991 |
| Mauritius | 2.91 | 2.76 | 3.06 | 1992 |
| Mauritius | 2.95 | 2.81 | 3.08 | 1993 |
| Mauritius | 2.99 | 2.86 | 3.11 | 1994 |
| Mauritius | 3.03 | 2.92 | 3.15 | 1995 |
| Mauritius | 3.08 | 2.97 | 3.2 | 1996 |
| Mauritius | 3.14 | 3.03 | 3.25 | 1997 |
| Mauritius | 3.19 | 3.08 | 3.3 | 1998 |
| Mauritius | 3.25 | 3.14 | 3.36 | 1999 |
| Mauritius | 3.31 | 3.2 | 3.42 | 2000 |
| Mauritius | 3.36 | 3.25 | 3.47 | 2001 |
| Mauritius | 3.42 | 3.31 | 3.53 | 2002 |
| Mauritius | 3.47 | 3.36 | 3.57 | 2003 |
| Mauritius | 3.51 | 3.4 | 3.62 | 2004 |
| Mauritius | 3.54 | 3.43 | 3.65 | 2005 |
| Mauritius | 3.57 | 3.46 | 3.68 | 2006 |
| Mauritius | 3.59 | 3.48 | 3.7 | 2007 |
| Mauritius | 3.6 | 3.5 | 3.71 | 2008 |
| Mauritius | 3.61 | 3.5 | 3.72 | 2009 |
| Mauritius | 3.61 | 3.5 | 3.72 | 2010 |
| Mauritius | 3.61 | 3.5 | 3.71 | 2011 |
| Mauritius | 3.6 | 3.5 | 3.71 | 2012 |
| Mauritius | 3.6 | 3.49 | 3.71 | 2013 |
| Mauritius | 3.59 | 3.47 | 3.71 | 2014 |
| Mauritius | 3.59 | 3.45 | 3.72 | 2015 |
| Mauritius | 3.58 | 3.43 | 3.73 | 2016 |
| Mauritius | 3.57 | 3.39 | 3.75 | 2017 |
| Mauritius | 3.56 | 3.35 | 3.77 | 2018 |
| Mauritius | 3.55 | 3.3 | 3.8 | 2019 |
| Mauritius | 3.54 | 3.25 | 3.83 | 2020 |
| Mauritius | 3.53 | 3.2 | 3.87 | 2021 |
| Mauritius | 3.52 | 3.13 | 3.91 | 2022 |
| Mauritius | 3.51 | 3.07 | 3.95 | 2023 |
| Mauritius | 3.5 | 3 | 3.99 | 2024 |
| Mauritius | 3.49 | 2.93 | 4.04 | 2025 |
| Mauritius | 3.47 | 2.86 | 4.08 | 2026 |
| Mauritius | 3.46 | 2.79 | 4.13 | 2027 |
| Mauritius | 3.45 | 2.71 | 4.18 | 2028 |
| Mauritius | 3.43 | 2.63 | 4.23 | 2029 |
| Mauritius | 3.41 | 2.55 | 4.28 | 2030 |
| Mexico | 4.92 | 4.88 | 4.97 | 1990 |
| Mexico | 5 | 4.97 | 5.04 | 1991 |
| Mexico | 5.09 | 5.06 | 5.12 | 1992 |
| Mexico | 5.19 | 5.16 | 5.22 | 1993 |
| Mexico | 5.29 | 5.27 | 5.32 | 1994 |
| Mexico | 5.4 | 5.38 | 5.43 | 1995 |
| Mexico | 5.52 | 5.49 | 5.54 | 1996 |
| Mexico | 5.63 | 5.6 | 5.66 | 1997 |
| Mexico | 5.74 | 5.71 | 5.77 | 1998 |
| Mexico | 5.84 | 5.81 | 5.87 | 1999 |
| Mexico | 5.94 | 5.91 | 5.97 | 2000 |
| Mexico | 6.05 | 6.02 | 6.08 | 2001 |
| Mexico | 6.17 | 6.14 | 6.19 | 2002 |
| Mexico | 6.29 | 6.26 | 6.32 | 2003 |
| Mexico | 6.4 | 6.37 | 6.43 | 2004 |
| Mexico | 6.5 | 6.47 | 6.53 | 2005 |
| Mexico | 6.59 | 6.56 | 6.62 | 2006 |
| Mexico | 6.68 | 6.65 | 6.71 | 2007 |
| Mexico | 6.77 | 6.74 | 6.8 | 2008 |
| Mexico | 6.84 | 6.81 | 6.87 | 2009 |
| Mexico | 6.9 | 6.87 | 6.93 | 2010 |
| Mexico | 6.95 | 6.92 | 6.98 | 2011 |
| Mexico | 7 | 6.97 | 7.03 | 2012 |
| Mexico | 7.06 | 7.03 | 7.09 | 2013 |
| Mexico | 7.11 | 7.08 | 7.13 | 2014 |
| Mexico | 7.14 | 7.11 | 7.17 | 2015 |
| Mexico | 7.17 | 7.14 | 7.2 | 2016 |
| Mexico | 7.2 | 7.16 | 7.24 | 2017 |
| Mexico | 7.23 | 7.16 | 7.3 | 2018 |
| Mexico | 7.26 | 7.15 | 7.36 | 2019 |
| Mexico | 7.28 | 7.13 | 7.42 | 2020 |
| Mexico | 7.29 | 7.11 | 7.48 | 2021 |
| Mexico | 7.31 | 7.07 | 7.54 | 2022 |
| Mexico | 7.32 | 7.03 | 7.62 | 2023 |
| Mexico | 7.34 | 6.99 | 7.69 | 2024 |
| Mexico | 7.35 | 6.94 | 7.76 | 2025 |
| Mexico | 7.36 | 6.88 | 7.83 | 2026 |
| Mexico | 7.36 | 6.82 | 7.91 | 2027 |
| Mexico | 7.37 | 6.75 | 7.99 | 2028 |
| Mexico | 7.38 | 6.68 | 8.07 | 2029 |
| Mexico | 7.38 | 6.61 | 8.14 | 2030 |
| Moldova | 6.25 | 6.09 | 6.41 | 1990 |
| Moldova | 6.26 | 6.14 | 6.39 | 1991 |
| Moldova | 6.27 | 6.16 | 6.38 | 1992 |
| Moldova | 6.27 | 6.17 | 6.37 | 1993 |
| Moldova | 6.26 | 6.16 | 6.35 | 1994 |
| Moldova | 6.22 | 6.12 | 6.32 | 1995 |
| Moldova | 6.16 | 6.07 | 6.26 | 1996 |
| Moldova | 6.1 | 6 | 6.19 | 1997 |
| Moldova | 6.04 | 5.95 | 6.13 | 1998 |
| Moldova | 6.01 | 5.91 | 6.1 | 1999 |
| Moldova | 6 | 5.9 | 6.09 | 2000 |
| Moldova | 6.02 | 5.93 | 6.12 | 2001 |
| Moldova | 6.07 | 5.98 | 6.17 | 2002 |
| Moldova | 6.15 | 6.06 | 6.24 | 2003 |
| Moldova | 6.25 | 6.15 | 6.34 | 2004 |
| Moldova | 6.35 | 6.26 | 6.45 | 2005 |
| Moldova | 6.46 | 6.37 | 6.56 | 2006 |
| Moldova | 6.57 | 6.47 | 6.66 | 2007 |
| Moldova | 6.66 | 6.56 | 6.76 | 2008 |
| Moldova | 6.74 | 6.64 | 6.84 | 2009 |
| Moldova | 6.8 | 6.71 | 6.9 | 2010 |
| Moldova | 6.85 | 6.75 | 6.95 | 2011 |
| Moldova | 6.89 | 6.8 | 6.99 | 2012 |
| Moldova | 6.93 | 6.83 | 7.03 | 2013 |
| Moldova | 6.96 | 6.86 | 7.06 | 2014 |
| Moldova | 6.97 | 6.86 | 7.08 | 2015 |
| Moldova | 6.98 | 6.85 | 7.1 | 2016 |
| Moldova | 6.97 | 6.8 | 7.13 | 2017 |
| Moldova | 6.95 | 6.74 | 7.17 | 2018 |
| Moldova | 6.94 | 6.65 | 7.23 | 2019 |
| Moldova | 6.92 | 6.55 | 7.29 | 2020 |
| Moldova | 6.9 | 6.44 | 7.35 | 2021 |
| Moldova | 6.87 | 6.32 | 7.42 | 2022 |
| Moldova | 6.84 | 6.19 | 7.49 | 2023 |
| Moldova | 6.81 | 6.05 | 7.56 | 2024 |
| Moldova | 6.77 | 5.91 | 7.64 | 2025 |
| Moldova | 6.74 | 5.76 | 7.72 | 2026 |
| Moldova | 6.69 | 5.6 | 7.79 | 2027 |
| Moldova | 6.65 | 5.43 | 7.87 | 2028 |
| Moldova | 6.61 | 5.26 | 7.95 | 2029 |
| Moldova | 6.56 | 5.09 | 8.03 | 2030 |
| Mongolia | 3.32 | 3.13 | 3.5 | 1990 |
| Mongolia | 3.44 | 3.28 | 3.6 | 1991 |
| Mongolia | 3.56 | 3.42 | 3.7 | 1992 |
| Mongolia | 3.69 | 3.56 | 3.82 | 1993 |
| Mongolia | 3.83 | 3.7 | 3.95 | 1994 |
| Mongolia | 3.97 | 3.85 | 4.09 | 1995 |
| Mongolia | 4.11 | 3.99 | 4.23 | 1996 |
| Mongolia | 4.25 | 4.13 | 4.37 | 1997 |
| Mongolia | 4.39 | 4.26 | 4.51 | 1998 |
| Mongolia | 4.52 | 4.4 | 4.65 | 1999 |
| Mongolia | 4.66 | 4.53 | 4.78 | 2000 |
| Mongolia | 4.79 | 4.66 | 4.91 | 2001 |
| Mongolia | 4.91 | 4.78 | 5.04 | 2002 |
| Mongolia | 5.03 | 4.9 | 5.15 | 2003 |
| Mongolia | 5.13 | 5 | 5.26 | 2004 |
| Mongolia | 5.22 | 5.09 | 5.35 | 2005 |
| Mongolia | 5.29 | 5.16 | 5.42 | 2006 |
| Mongolia | 5.35 | 5.22 | 5.48 | 2007 |
| Mongolia | 5.39 | 5.26 | 5.52 | 2008 |
| Mongolia | 5.42 | 5.29 | 5.55 | 2009 |
| Mongolia | 5.44 | 5.31 | 5.56 | 2010 |
| Mongolia | 5.44 | 5.31 | 5.57 | 2011 |
| Mongolia | 5.43 | 5.31 | 5.56 | 2012 |
| Mongolia | 5.42 | 5.3 | 5.55 | 2013 |
| Mongolia | 5.41 | 5.28 | 5.54 | 2014 |
| Mongolia | 5.39 | 5.25 | 5.54 | 2015 |
| Mongolia | 5.38 | 5.21 | 5.55 | 2016 |
| Mongolia | 5.37 | 5.17 | 5.57 | 2017 |
| Mongolia | 5.36 | 5.11 | 5.61 | 2018 |
| Mongolia | 5.35 | 5.04 | 5.66 | 2019 |
| Mongolia | 5.35 | 4.97 | 5.73 | 2020 |
| Mongolia | 5.35 | 4.89 | 5.8 | 2021 |
| Mongolia | 5.35 | 4.81 | 5.88 | 2022 |
| Mongolia | 5.35 | 4.72 | 5.97 | 2023 |
| Mongolia | 5.35 | 4.63 | 6.07 | 2024 |
| Mongolia | 5.36 | 4.54 | 6.18 | 2025 |
| Mongolia | 5.37 | 4.44 | 6.29 | 2026 |
| Mongolia | 5.38 | 4.34 | 6.41 | 2027 |
| Mongolia | 5.39 | 4.24 | 6.53 | 2028 |
| Mongolia | 5.4 | 4.13 | 6.66 | 2029 |
| Mongolia | 5.41 | 4.02 | 6.8 | 2030 |
| Montenegro | 5.73 | 5.41 | 6.05 | 1990 |
| Montenegro | 5.73 | 5.45 | 6 | 1991 |
| Montenegro | 5.73 | 5.49 | 5.97 | 1992 |
| Montenegro | 5.72 | 5.51 | 5.94 | 1993 |
| Montenegro | 5.72 | 5.52 | 5.91 | 1994 |
| Montenegro | 5.71 | 5.52 | 5.89 | 1995 |
| Montenegro | 5.7 | 5.52 | 5.87 | 1996 |
| Montenegro | 5.69 | 5.52 | 5.86 | 1997 |
| Montenegro | 5.67 | 5.51 | 5.84 | 1998 |
| Montenegro | 5.66 | 5.49 | 5.82 | 1999 |
| Montenegro | 5.64 | 5.48 | 5.8 | 2000 |
| Montenegro | 5.62 | 5.46 | 5.79 | 2001 |
| Montenegro | 5.61 | 5.45 | 5.77 | 2002 |
| Montenegro | 5.59 | 5.43 | 5.75 | 2003 |
| Montenegro | 5.57 | 5.41 | 5.73 | 2004 |
| Montenegro | 5.55 | 5.39 | 5.71 | 2005 |
| Montenegro | 5.53 | 5.37 | 5.69 | 2006 |
| Montenegro | 5.51 | 5.36 | 5.66 | 2007 |
| Montenegro | 5.49 | 5.33 | 5.64 | 2008 |
| Montenegro | 5.47 | 5.31 | 5.62 | 2009 |
| Montenegro | 5.44 | 5.29 | 5.6 | 2010 |
| Montenegro | 5.42 | 5.27 | 5.57 | 2011 |
| Montenegro | 5.4 | 5.24 | 5.55 | 2012 |
| Montenegro | 5.37 | 5.2 | 5.53 | 2013 |
| Montenegro | 5.34 | 5.16 | 5.52 | 2014 |
| Montenegro | 5.31 | 5.11 | 5.5 | 2015 |
| Montenegro | 5.27 | 5.05 | 5.49 | 2016 |
| Montenegro | 5.23 | 4.97 | 5.48 | 2017 |
| Montenegro | 5.18 | 4.88 | 5.48 | 2018 |
| Montenegro | 5.13 | 4.79 | 5.47 | 2019 |
| Montenegro | 5.07 | 4.68 | 5.47 | 2020 |
| Montenegro | 5.01 | 4.57 | 5.46 | 2021 |
| Montenegro | 4.95 | 4.44 | 5.45 | 2022 |
| Montenegro | 4.88 | 4.31 | 5.44 | 2023 |
| Montenegro | 4.8 | 4.18 | 5.42 | 2024 |
| Montenegro | 4.72 | 4.04 | 5.4 | 2025 |
| Montenegro | 4.63 | 3.89 | 5.37 | 2026 |
| Montenegro | 4.54 | 3.74 | 5.34 | 2027 |
| Montenegro | 4.45 | 3.59 | 5.3 | 2028 |
| Montenegro | 4.35 | 3.43 | 5.26 | 2029 |
| Montenegro | 4.24 | 3.28 | 5.21 | 2030 |
| Morocco | 1.38 | 1.34 | 1.42 | 1990 |
| Morocco | 1.4 | 1.36 | 1.43 | 1991 |
| Morocco | 1.41 | 1.39 | 1.44 | 1992 |
| Morocco | 1.43 | 1.41 | 1.46 | 1993 |
| Morocco | 1.45 | 1.43 | 1.47 | 1994 |
| Morocco | 1.47 | 1.45 | 1.49 | 1995 |
| Morocco | 1.49 | 1.47 | 1.51 | 1996 |
| Morocco | 1.51 | 1.49 | 1.53 | 1997 |
| Morocco | 1.53 | 1.51 | 1.55 | 1998 |
| Morocco | 1.54 | 1.52 | 1.56 | 1999 |
| Morocco | 1.56 | 1.54 | 1.58 | 2000 |
| Morocco | 1.57 | 1.55 | 1.59 | 2001 |
| Morocco | 1.59 | 1.57 | 1.61 | 2002 |
| Morocco | 1.6 | 1.58 | 1.62 | 2003 |
| Morocco | 1.62 | 1.6 | 1.64 | 2004 |
| Morocco | 1.63 | 1.61 | 1.65 | 2005 |
| Morocco | 1.65 | 1.63 | 1.67 | 2006 |
| Morocco | 1.67 | 1.65 | 1.69 | 2007 |
| Morocco | 1.7 | 1.68 | 1.71 | 2008 |
| Morocco | 1.72 | 1.7 | 1.74 | 2009 |
| Morocco | 1.74 | 1.72 | 1.76 | 2010 |
| Morocco | 1.76 | 1.74 | 1.78 | 2011 |
| Morocco | 1.78 | 1.76 | 1.8 | 2012 |
| Morocco | 1.8 | 1.78 | 1.82 | 2013 |
| Morocco | 1.82 | 1.8 | 1.84 | 2014 |
| Morocco | 1.84 | 1.82 | 1.86 | 2015 |
| Morocco | 1.86 | 1.83 | 1.88 | 2016 |
| Morocco | 1.87 | 1.84 | 1.91 | 2017 |
| Morocco | 1.89 | 1.85 | 1.93 | 2018 |
| Morocco | 1.9 | 1.85 | 1.96 | 2019 |
| Morocco | 1.92 | 1.85 | 1.99 | 2020 |
| Morocco | 1.93 | 1.84 | 2.02 | 2021 |
| Morocco | 1.95 | 1.84 | 2.06 | 2022 |
| Morocco | 1.96 | 1.83 | 2.09 | 2023 |
| Morocco | 1.98 | 1.83 | 2.13 | 2024 |
| Morocco | 1.99 | 1.82 | 2.16 | 2025 |
| Morocco | 2 | 1.81 | 2.2 | 2026 |
| Morocco | 2.02 | 1.79 | 2.24 | 2027 |
| Morocco | 2.03 | 1.78 | 2.28 | 2028 |
| Morocco | 2.04 | 1.76 | 2.32 | 2029 |
| Morocco | 2.06 | 1.75 | 2.37 | 2030 |
| Mozambique | 2.05 | 1.99 | 2.12 | 1990 |
| Mozambique | 1.99 | 1.94 | 2.04 | 1991 |
| Mozambique | 1.93 | 1.88 | 1.97 | 1992 |
| Mozambique | 1.87 | 1.83 | 1.91 | 1993 |
| Mozambique | 1.81 | 1.78 | 1.85 | 1994 |
| Mozambique | 1.76 | 1.73 | 1.8 | 1995 |
| Mozambique | 1.72 | 1.69 | 1.75 | 1996 |
| Mozambique | 1.68 | 1.65 | 1.71 | 1997 |
| Mozambique | 1.66 | 1.63 | 1.68 | 1998 |
| Mozambique | 1.63 | 1.61 | 1.66 | 1999 |
| Mozambique | 1.62 | 1.59 | 1.65 | 2000 |
| Mozambique | 1.62 | 1.59 | 1.64 | 2001 |
| Mozambique | 1.62 | 1.59 | 1.65 | 2002 |
| Mozambique | 1.63 | 1.61 | 1.66 | 2003 |
| Mozambique | 1.66 | 1.63 | 1.68 | 2004 |
| Mozambique | 1.69 | 1.66 | 1.72 | 2005 |
| Mozambique | 1.73 | 1.7 | 1.76 | 2006 |
| Mozambique | 1.77 | 1.74 | 1.8 | 2007 |
| Mozambique | 1.81 | 1.79 | 1.84 | 2008 |
| Mozambique | 1.86 | 1.83 | 1.88 | 2009 |
| Mozambique | 1.89 | 1.87 | 1.92 | 2010 |
| Mozambique | 1.93 | 1.9 | 1.96 | 2011 |
| Mozambique | 1.97 | 1.94 | 2 | 2012 |
| Mozambique | 2.01 | 1.98 | 2.04 | 2013 |
| Mozambique | 2.05 | 2.02 | 2.08 | 2014 |
| Mozambique | 2.09 | 2.06 | 2.12 | 2015 |
| Mozambique | 2.13 | 2.09 | 2.17 | 2016 |
| Mozambique | 2.17 | 2.12 | 2.22 | 2017 |
| Mozambique | 2.21 | 2.14 | 2.28 | 2018 |
| Mozambique | 2.25 | 2.16 | 2.34 | 2019 |
| Mozambique | 2.29 | 2.17 | 2.41 | 2020 |
| Mozambique | 2.33 | 2.18 | 2.49 | 2021 |
| Mozambique | 2.37 | 2.18 | 2.57 | 2022 |
| Mozambique | 2.42 | 2.18 | 2.65 | 2023 |
| Mozambique | 2.46 | 2.18 | 2.74 | 2024 |
| Mozambique | 2.5 | 2.18 | 2.83 | 2025 |
| Mozambique | 2.54 | 2.17 | 2.92 | 2026 |
| Mozambique | 2.59 | 2.15 | 3.02 | 2027 |
| Mozambique | 2.63 | 2.14 | 3.12 | 2028 |
| Mozambique | 2.67 | 2.11 | 3.23 | 2029 |
| Mozambique | 2.71 | 2.09 | 3.34 | 2030 |
| Myanmar | 3.66 | 3.61 | 3.71 | 1990 |
| Myanmar | 3.72 | 3.68 | 3.75 | 1991 |
| Myanmar | 3.77 | 3.74 | 3.81 | 1992 |
| Myanmar | 3.83 | 3.8 | 3.86 | 1993 |
| Myanmar | 3.89 | 3.86 | 3.92 | 1994 |
| Myanmar | 3.95 | 3.92 | 3.98 | 1995 |
| Myanmar | 4.02 | 3.99 | 4.05 | 1996 |
| Myanmar | 4.09 | 4.06 | 4.13 | 1997 |
| Myanmar | 4.17 | 4.14 | 4.2 | 1998 |
| Myanmar | 4.25 | 4.22 | 4.28 | 1999 |
| Myanmar | 4.32 | 4.29 | 4.35 | 2000 |
| Myanmar | 4.4 | 4.37 | 4.43 | 2001 |
| Myanmar | 4.47 | 4.44 | 4.5 | 2002 |
| Myanmar | 4.54 | 4.51 | 4.57 | 2003 |
| Myanmar | 4.6 | 4.57 | 4.63 | 2004 |
| Myanmar | 4.64 | 4.61 | 4.68 | 2005 |
| Myanmar | 4.68 | 4.64 | 4.71 | 2006 |
| Myanmar | 4.69 | 4.66 | 4.72 | 2007 |
| Myanmar | 4.69 | 4.66 | 4.72 | 2008 |
| Myanmar | 4.67 | 4.64 | 4.71 | 2009 |
| Myanmar | 4.65 | 4.61 | 4.68 | 2010 |
| Myanmar | 4.61 | 4.58 | 4.64 | 2011 |
| Myanmar | 4.58 | 4.55 | 4.61 | 2012 |
| Myanmar | 4.55 | 4.52 | 4.58 | 2013 |
| Myanmar | 4.52 | 4.49 | 4.55 | 2014 |
| Myanmar | 4.49 | 4.46 | 4.53 | 2015 |
| Myanmar | 4.47 | 4.44 | 4.51 | 2016 |
| Myanmar | 4.45 | 4.4 | 4.5 | 2017 |
| Myanmar | 4.43 | 4.36 | 4.5 | 2018 |
| Myanmar | 4.4 | 4.31 | 4.5 | 2019 |
| Myanmar | 4.38 | 4.25 | 4.51 | 2020 |
| Myanmar | 4.36 | 4.2 | 4.52 | 2021 |
| Myanmar | 4.33 | 4.13 | 4.53 | 2022 |
| Myanmar | 4.31 | 4.07 | 4.54 | 2023 |
| Myanmar | 4.28 | 4 | 4.56 | 2024 |
| Myanmar | 4.25 | 3.93 | 4.58 | 2025 |
| Myanmar | 4.23 | 3.86 | 4.6 | 2026 |
| Myanmar | 4.2 | 3.79 | 4.62 | 2027 |
| Myanmar | 4.18 | 3.71 | 4.64 | 2028 |
| Myanmar | 4.15 | 3.64 | 4.66 | 2029 |
| Myanmar | 4.12 | 3.56 | 4.69 | 2030 |
| Namibia | 2.11 | 1.94 | 2.29 | 1990 |
| Namibia | 2.16 | 2.01 | 2.31 | 1991 |
| Namibia | 2.21 | 2.07 | 2.34 | 1992 |
| Namibia | 2.26 | 2.13 | 2.38 | 1993 |
| Namibia | 2.31 | 2.19 | 2.42 | 1994 |
| Namibia | 2.36 | 2.25 | 2.46 | 1995 |
| Namibia | 2.41 | 2.3 | 2.51 | 1996 |
| Namibia | 2.46 | 2.35 | 2.56 | 1997 |
| Namibia | 2.5 | 2.4 | 2.6 | 1998 |
| Namibia | 2.55 | 2.45 | 2.65 | 1999 |
| Namibia | 2.59 | 2.49 | 2.69 | 2000 |
| Namibia | 2.62 | 2.52 | 2.72 | 2001 |
| Namibia | 2.65 | 2.55 | 2.74 | 2002 |
| Namibia | 2.66 | 2.56 | 2.76 | 2003 |
| Namibia | 2.67 | 2.57 | 2.77 | 2004 |
| Namibia | 2.67 | 2.57 | 2.77 | 2005 |
| Namibia | 2.66 | 2.57 | 2.76 | 2006 |
| Namibia | 2.65 | 2.56 | 2.75 | 2007 |
| Namibia | 2.63 | 2.54 | 2.73 | 2008 |
| Namibia | 2.61 | 2.52 | 2.7 | 2009 |
| Namibia | 2.58 | 2.49 | 2.67 | 2010 |
| Namibia | 2.55 | 2.46 | 2.65 | 2011 |
| Namibia | 2.53 | 2.43 | 2.62 | 2012 |
| Namibia | 2.5 | 2.4 | 2.59 | 2013 |
| Namibia | 2.47 | 2.37 | 2.57 | 2014 |
| Namibia | 2.44 | 2.33 | 2.56 | 2015 |
| Namibia | 2.42 | 2.29 | 2.54 | 2016 |
| Namibia | 2.39 | 2.25 | 2.54 | 2017 |
| Namibia | 2.37 | 2.2 | 2.54 | 2018 |
| Namibia | 2.35 | 2.15 | 2.54 | 2019 |
| Namibia | 2.32 | 2.1 | 2.55 | 2020 |
| Namibia | 2.3 | 2.04 | 2.56 | 2021 |
| Namibia | 2.28 | 1.99 | 2.57 | 2022 |
| Namibia | 2.26 | 1.93 | 2.59 | 2023 |
| Namibia | 2.24 | 1.87 | 2.6 | 2024 |
| Namibia | 2.22 | 1.81 | 2.62 | 2025 |
| Namibia | 2.2 | 1.75 | 2.65 | 2026 |
| Namibia | 2.18 | 1.69 | 2.67 | 2027 |
| Namibia | 2.16 | 1.63 | 2.7 | 2028 |
| Namibia | 2.15 | 1.57 | 2.73 | 2029 |
| Namibia | 2.13 | 1.51 | 2.76 | 2030 |
| Nepal | 1.21 | 1.17 | 1.25 | 1990 |
| Nepal | 1.2 | 1.17 | 1.24 | 1991 |
| Nepal | 1.19 | 1.17 | 1.22 | 1992 |
| Nepal | 1.19 | 1.16 | 1.21 | 1993 |
| Nepal | 1.18 | 1.16 | 1.2 | 1994 |
| Nepal | 1.17 | 1.15 | 1.2 | 1995 |
| Nepal | 1.17 | 1.15 | 1.19 | 1996 |
| Nepal | 1.16 | 1.14 | 1.19 | 1997 |
| Nepal | 1.16 | 1.14 | 1.18 | 1998 |
| Nepal | 1.17 | 1.14 | 1.19 | 1999 |
| Nepal | 1.17 | 1.15 | 1.19 | 2000 |
| Nepal | 1.18 | 1.16 | 1.2 | 2001 |
| Nepal | 1.19 | 1.17 | 1.21 | 2002 |
| Nepal | 1.21 | 1.19 | 1.23 | 2003 |
| Nepal | 1.24 | 1.22 | 1.26 | 2004 |
| Nepal | 1.27 | 1.24 | 1.29 | 2005 |
| Nepal | 1.3 | 1.28 | 1.32 | 2006 |
| Nepal | 1.34 | 1.32 | 1.36 | 2007 |
| Nepal | 1.38 | 1.36 | 1.4 | 2008 |
| Nepal | 1.43 | 1.41 | 1.45 | 2009 |
| Nepal | 1.48 | 1.46 | 1.5 | 2010 |
| Nepal | 1.54 | 1.51 | 1.56 | 2011 |
| Nepal | 1.59 | 1.57 | 1.61 | 2012 |
| Nepal | 1.65 | 1.62 | 1.67 | 2013 |
| Nepal | 1.7 | 1.68 | 1.73 | 2014 |
| Nepal | 1.76 | 1.73 | 1.79 | 2015 |
| Nepal | 1.81 | 1.78 | 1.84 | 2016 |
| Nepal | 1.87 | 1.83 | 1.91 | 2017 |
| Nepal | 1.92 | 1.86 | 1.98 | 2018 |
| Nepal | 1.98 | 1.9 | 2.05 | 2019 |
| Nepal | 2.03 | 1.93 | 2.14 | 2020 |
| Nepal | 2.09 | 1.96 | 2.22 | 2021 |
| Nepal | 2.15 | 1.99 | 2.31 | 2022 |
| Nepal | 2.21 | 2.01 | 2.41 | 2023 |
| Nepal | 2.27 | 2.03 | 2.51 | 2024 |
| Nepal | 2.33 | 2.05 | 2.62 | 2025 |
| Nepal | 2.4 | 2.06 | 2.73 | 2026 |
| Nepal | 2.46 | 2.07 | 2.84 | 2027 |
| Nepal | 2.52 | 2.08 | 2.96 | 2028 |
| Nepal | 2.59 | 2.09 | 3.09 | 2029 |
| Nepal | 2.65 | 2.09 | 3.22 | 2030 |
| Netherlands | 7.59 | 7.49 | 7.68 | 1990 |
| Netherlands | 7.74 | 7.66 | 7.81 | 1991 |
| Netherlands | 7.87 | 7.81 | 7.93 | 1992 |
| Netherlands | 7.99 | 7.93 | 8.05 | 1993 |
| Netherlands | 8.08 | 8.02 | 8.14 | 1994 |
| Netherlands | 8.18 | 8.12 | 8.24 | 1995 |
| Netherlands | 8.32 | 8.26 | 8.38 | 1996 |
| Netherlands | 8.48 | 8.42 | 8.54 | 1997 |
| Netherlands | 8.65 | 8.59 | 8.71 | 1998 |
| Netherlands | 8.82 | 8.76 | 8.88 | 1999 |
| Netherlands | 8.97 | 8.91 | 9.03 | 2000 |
| Netherlands | 9.13 | 9.07 | 9.18 | 2001 |
| Netherlands | 9.26 | 9.2 | 9.32 | 2002 |
| Netherlands | 9.37 | 9.31 | 9.43 | 2003 |
| Netherlands | 9.46 | 9.4 | 9.52 | 2004 |
| Netherlands | 9.51 | 9.45 | 9.57 | 2005 |
| Netherlands | 9.56 | 9.5 | 9.62 | 2006 |
| Netherlands | 9.6 | 9.54 | 9.66 | 2007 |
| Netherlands | 9.62 | 9.56 | 9.68 | 2008 |
| Netherlands | 9.62 | 9.56 | 9.68 | 2009 |
| Netherlands | 9.59 | 9.54 | 9.65 | 2010 |
| Netherlands | 9.55 | 9.49 | 9.61 | 2011 |
| Netherlands | 9.48 | 9.43 | 9.54 | 2012 |
| Netherlands | 9.41 | 9.35 | 9.46 | 2013 |
| Netherlands | 9.32 | 9.26 | 9.38 | 2014 |
| Netherlands | 9.24 | 9.19 | 9.3 | 2015 |
| Netherlands | 9.18 | 9.11 | 9.24 | 2016 |
| Netherlands | 9.12 | 9.03 | 9.21 | 2017 |
| Netherlands | 9.06 | 8.93 | 9.19 | 2018 |
| Netherlands | 9 | 8.81 | 9.18 | 2019 |
| Netherlands | 8.94 | 8.69 | 9.19 | 2020 |
| Netherlands | 8.88 | 8.56 | 9.2 | 2021 |
| Netherlands | 8.82 | 8.42 | 9.22 | 2022 |
| Netherlands | 8.76 | 8.28 | 9.25 | 2023 |
| Netherlands | 8.7 | 8.13 | 9.27 | 2024 |
| Netherlands | 8.64 | 7.98 | 9.3 | 2025 |
| Netherlands | 8.58 | 7.82 | 9.33 | 2026 |
| Netherlands | 8.52 | 7.66 | 9.37 | 2027 |
| Netherlands | 8.45 | 7.5 | 9.41 | 2028 |
| Netherlands | 8.39 | 7.33 | 9.45 | 2029 |
| Netherlands | 8.32 | 7.15 | 9.48 | 2030 |
| New Zealand | 8.05 | 7.86 | 8.23 | 1990 |
| New Zealand | 8.06 | 7.91 | 8.21 | 1991 |
| New Zealand | 8.08 | 7.95 | 8.2 | 1992 |
| New Zealand | 8.08 | 7.97 | 8.2 | 1993 |
| New Zealand | 8.09 | 7.98 | 8.2 | 1994 |
| New Zealand | 8.1 | 7.99 | 8.21 | 1995 |
| New Zealand | 8.12 | 8.01 | 8.22 | 1996 |
| New Zealand | 8.15 | 8.05 | 8.26 | 1997 |
| New Zealand | 8.2 | 8.09 | 8.3 | 1998 |
| New Zealand | 8.26 | 8.16 | 8.36 | 1999 |
| New Zealand | 8.34 | 8.23 | 8.44 | 2000 |
| New Zealand | 8.43 | 8.33 | 8.53 | 2001 |
| New Zealand | 8.53 | 8.43 | 8.63 | 2002 |
| New Zealand | 8.64 | 8.54 | 8.74 | 2003 |
| New Zealand | 8.74 | 8.64 | 8.85 | 2004 |
| New Zealand | 8.84 | 8.74 | 8.94 | 2005 |
| New Zealand | 8.93 | 8.82 | 9.03 | 2006 |
| New Zealand | 9 | 8.89 | 9.1 | 2007 |
| New Zealand | 9.04 | 8.94 | 9.15 | 2008 |
| New Zealand | 9.07 | 8.97 | 9.17 | 2009 |
| New Zealand | 9.07 | 8.97 | 9.17 | 2010 |
| New Zealand | 9.05 | 8.95 | 9.15 | 2011 |
| New Zealand | 9.02 | 8.92 | 9.12 | 2012 |
| New Zealand | 8.97 | 8.88 | 9.07 | 2013 |
| New Zealand | 8.92 | 8.82 | 9.02 | 2014 |
| New Zealand | 8.86 | 8.75 | 8.97 | 2015 |
| New Zealand | 8.8 | 8.68 | 8.93 | 2016 |
| New Zealand | 8.74 | 8.59 | 8.9 | 2017 |
| New Zealand | 8.69 | 8.48 | 8.9 | 2018 |
| New Zealand | 8.63 | 8.36 | 8.91 | 2019 |
| New Zealand | 8.58 | 8.23 | 8.93 | 2020 |
| New Zealand | 8.53 | 8.1 | 8.96 | 2021 |
| New Zealand | 8.48 | 7.96 | 8.99 | 2022 |
| New Zealand | 8.43 | 7.82 | 9.03 | 2023 |
| New Zealand | 8.38 | 7.67 | 9.08 | 2024 |
| New Zealand | 8.33 | 7.52 | 9.14 | 2025 |
| New Zealand | 8.28 | 7.37 | 9.2 | 2026 |
| New Zealand | 8.24 | 7.21 | 9.26 | 2027 |
| New Zealand | 8.19 | 7.05 | 9.33 | 2028 |
| New Zealand | 8.14 | 6.89 | 9.4 | 2029 |
| New Zealand | 8.1 | 6.72 | 9.47 | 2030 |
| Nicaragua | 3.1 | 2.98 | 3.23 | 1990 |
| Nicaragua | 3.09 | 2.99 | 3.2 | 1991 |
| Nicaragua | 3.08 | 2.99 | 3.17 | 1992 |
| Nicaragua | 3.07 | 2.98 | 3.15 | 1993 |
| Nicaragua | 3.05 | 2.98 | 3.13 | 1994 |
| Nicaragua | 3.04 | 2.97 | 3.11 | 1995 |
| Nicaragua | 3.03 | 2.96 | 3.1 | 1996 |
| Nicaragua | 3.02 | 2.96 | 3.09 | 1997 |
| Nicaragua | 3.02 | 2.96 | 3.09 | 1998 |
| Nicaragua | 3.03 | 2.96 | 3.1 | 1999 |
| Nicaragua | 3.05 | 2.98 | 3.11 | 2000 |
| Nicaragua | 3.07 | 3 | 3.13 | 2001 |
| Nicaragua | 3.1 | 3.03 | 3.16 | 2002 |
| Nicaragua | 3.13 | 3.07 | 3.19 | 2003 |
| Nicaragua | 3.16 | 3.1 | 3.22 | 2004 |
| Nicaragua | 3.19 | 3.13 | 3.26 | 2005 |
| Nicaragua | 3.22 | 3.16 | 3.28 | 2006 |
| Nicaragua | 3.25 | 3.18 | 3.31 | 2007 |
| Nicaragua | 3.27 | 3.2 | 3.33 | 2008 |
| Nicaragua | 3.28 | 3.22 | 3.35 | 2009 |
| Nicaragua | 3.3 | 3.23 | 3.36 | 2010 |
| Nicaragua | 3.31 | 3.25 | 3.37 | 2011 |
| Nicaragua | 3.32 | 3.25 | 3.38 | 2012 |
| Nicaragua | 3.32 | 3.26 | 3.39 | 2013 |
| Nicaragua | 3.33 | 3.26 | 3.39 | 2014 |
| Nicaragua | 3.33 | 3.26 | 3.4 | 2015 |
| Nicaragua | 3.33 | 3.25 | 3.42 | 2016 |
| Nicaragua | 3.33 | 3.23 | 3.44 | 2017 |
| Nicaragua | 3.33 | 3.21 | 3.46 | 2018 |
| Nicaragua | 3.33 | 3.17 | 3.49 | 2019 |
| Nicaragua | 3.33 | 3.14 | 3.52 | 2020 |
| Nicaragua | 3.33 | 3.1 | 3.55 | 2021 |
| Nicaragua | 3.32 | 3.06 | 3.59 | 2022 |
| Nicaragua | 3.31 | 3.01 | 3.62 | 2023 |
| Nicaragua | 3.31 | 2.96 | 3.66 | 2024 |
| Nicaragua | 3.3 | 2.9 | 3.7 | 2025 |
| Nicaragua | 3.29 | 2.84 | 3.73 | 2026 |
| Nicaragua | 3.28 | 2.78 | 3.77 | 2027 |
| Nicaragua | 3.26 | 2.72 | 3.81 | 2028 |
| Nicaragua | 3.25 | 2.65 | 3.85 | 2029 |
| Nicaragua | 3.23 | 2.58 | 3.88 | 2030 |
| Niger | 1.9 | 1.82 | 1.97 | 1990 |
| Niger | 1.87 | 1.81 | 1.94 | 1991 |
| Niger | 1.85 | 1.79 | 1.9 | 1992 |
| Niger | 1.83 | 1.78 | 1.87 | 1993 |
| Niger | 1.81 | 1.76 | 1.85 | 1994 |
| Niger | 1.79 | 1.75 | 1.83 | 1995 |
| Niger | 1.77 | 1.73 | 1.81 | 1996 |
| Niger | 1.76 | 1.73 | 1.8 | 1997 |
| Niger | 1.76 | 1.72 | 1.8 | 1998 |
| Niger | 1.76 | 1.72 | 1.79 | 1999 |
| Niger | 1.76 | 1.73 | 1.8 | 2000 |
| Niger | 1.77 | 1.74 | 1.81 | 2001 |
| Niger | 1.79 | 1.75 | 1.82 | 2002 |
| Niger | 1.8 | 1.77 | 1.83 | 2003 |
| Niger | 1.82 | 1.79 | 1.85 | 2004 |
| Niger | 1.84 | 1.8 | 1.87 | 2005 |
| Niger | 1.85 | 1.82 | 1.89 | 2006 |
| Niger | 1.87 | 1.84 | 1.9 | 2007 |
| Niger | 1.88 | 1.85 | 1.92 | 2008 |
| Niger | 1.9 | 1.87 | 1.93 | 2009 |
| Niger | 1.92 | 1.88 | 1.95 | 2010 |
| Niger | 1.93 | 1.9 | 1.97 | 2011 |
| Niger | 1.96 | 1.92 | 1.99 | 2012 |
| Niger | 1.98 | 1.95 | 2.02 | 2013 |
| Niger | 2.01 | 1.97 | 2.04 | 2014 |
| Niger | 2.04 | 2 | 2.08 | 2015 |
| Niger | 2.07 | 2.02 | 2.11 | 2016 |
| Niger | 2.1 | 2.04 | 2.15 | 2017 |
| Niger | 2.13 | 2.06 | 2.2 | 2018 |
| Niger | 2.16 | 2.07 | 2.24 | 2019 |
| Niger | 2.19 | 2.08 | 2.29 | 2020 |
| Niger | 2.22 | 2.09 | 2.35 | 2021 |
| Niger | 2.25 | 2.09 | 2.41 | 2022 |
| Niger | 2.28 | 2.09 | 2.47 | 2023 |
| Niger | 2.31 | 2.09 | 2.53 | 2024 |
| Niger | 2.34 | 2.09 | 2.59 | 2025 |
| Niger | 2.37 | 2.08 | 2.66 | 2026 |
| Niger | 2.4 | 2.08 | 2.73 | 2027 |
| Niger | 2.44 | 2.07 | 2.8 | 2028 |
| Niger | 2.47 | 2.05 | 2.88 | 2029 |
| Niger | 2.5 | 2.04 | 2.96 | 2030 |
| Nigeria | 2.31 | 2.27 | 2.34 | 1990 |
| Nigeria | 2.34 | 2.31 | 2.36 | 1991 |
| Nigeria | 2.37 | 2.35 | 2.39 | 1992 |
| Nigeria | 2.41 | 2.39 | 2.43 | 1993 |
| Nigeria | 2.44 | 2.42 | 2.46 | 1994 |
| Nigeria | 2.47 | 2.45 | 2.49 | 1995 |
| Nigeria | 2.5 | 2.48 | 2.52 | 1996 |
| Nigeria | 2.52 | 2.5 | 2.54 | 1997 |
| Nigeria | 2.54 | 2.52 | 2.56 | 1998 |
| Nigeria | 2.55 | 2.53 | 2.57 | 1999 |
| Nigeria | 2.55 | 2.53 | 2.57 | 2000 |
| Nigeria | 2.54 | 2.52 | 2.56 | 2001 |
| Nigeria | 2.52 | 2.51 | 2.54 | 2002 |
| Nigeria | 2.51 | 2.49 | 2.53 | 2003 |
| Nigeria | 2.5 | 2.48 | 2.51 | 2004 |
| Nigeria | 2.49 | 2.47 | 2.5 | 2005 |
| Nigeria | 2.48 | 2.47 | 2.5 | 2006 |
| Nigeria | 2.49 | 2.47 | 2.5 | 2007 |
| Nigeria | 2.49 | 2.47 | 2.51 | 2008 |
| Nigeria | 2.51 | 2.49 | 2.52 | 2009 |
| Nigeria | 2.53 | 2.52 | 2.55 | 2010 |
| Nigeria | 2.58 | 2.56 | 2.59 | 2011 |
| Nigeria | 2.63 | 2.61 | 2.65 | 2012 |
| Nigeria | 2.69 | 2.68 | 2.71 | 2013 |
| Nigeria | 2.76 | 2.75 | 2.78 | 2014 |
| Nigeria | 2.84 | 2.82 | 2.85 | 2015 |
| Nigeria | 2.91 | 2.89 | 2.93 | 2016 |
| Nigeria | 2.97 | 2.94 | 2.99 | 2017 |
| Nigeria | 3.03 | 2.99 | 3.07 | 2018 |
| Nigeria | 3.1 | 3.03 | 3.16 | 2019 |
| Nigeria | 3.16 | 3.07 | 3.25 | 2020 |
| Nigeria | 3.23 | 3.11 | 3.35 | 2021 |
| Nigeria | 3.3 | 3.14 | 3.45 | 2022 |
| Nigeria | 3.37 | 3.17 | 3.56 | 2023 |
| Nigeria | 3.44 | 3.2 | 3.67 | 2024 |
| Nigeria | 3.51 | 3.23 | 3.79 | 2025 |
| Nigeria | 3.58 | 3.25 | 3.91 | 2026 |
| Nigeria | 3.66 | 3.27 | 4.05 | 2027 |
| Nigeria | 3.74 | 3.29 | 4.18 | 2028 |
| Nigeria | 3.82 | 3.31 | 4.33 | 2029 |
| Nigeria | 3.9 | 3.32 | 4.48 | 2030 |
| North Korea | 3.71 | 3.64 | 3.77 | 1990 |
| North Korea | 3.7 | 3.65 | 3.75 | 1991 |
| North Korea | 3.69 | 3.65 | 3.74 | 1992 |
| North Korea | 3.69 | 3.65 | 3.73 | 1993 |
| North Korea | 3.69 | 3.65 | 3.73 | 1994 |
| North Korea | 3.69 | 3.66 | 3.73 | 1995 |
| North Korea | 3.7 | 3.66 | 3.74 | 1996 |
| North Korea | 3.71 | 3.68 | 3.75 | 1997 |
| North Korea | 3.74 | 3.7 | 3.77 | 1998 |
| North Korea | 3.77 | 3.73 | 3.81 | 1999 |
| North Korea | 3.8 | 3.77 | 3.84 | 2000 |
| North Korea | 3.83 | 3.8 | 3.87 | 2001 |
| North Korea | 3.86 | 3.83 | 3.9 | 2002 |
| North Korea | 3.89 | 3.86 | 3.93 | 2003 |
| North Korea | 3.93 | 3.89 | 3.96 | 2004 |
| North Korea | 3.95 | 3.91 | 3.99 | 2005 |
| North Korea | 3.97 | 3.93 | 4 | 2006 |
| North Korea | 3.98 | 3.94 | 4.01 | 2007 |
| North Korea | 3.99 | 3.96 | 4.03 | 2008 |
| North Korea | 4 | 3.97 | 4.04 | 2009 |
| North Korea | 4 | 3.97 | 4.04 | 2010 |
| North Korea | 4 | 3.96 | 4.03 | 2011 |
| North Korea | 3.99 | 3.95 | 4.02 | 2012 |
| North Korea | 3.97 | 3.94 | 4.01 | 2013 |
| North Korea | 3.96 | 3.93 | 4 | 2014 |
| North Korea | 3.95 | 3.91 | 3.98 | 2015 |
| North Korea | 3.92 | 3.88 | 3.96 | 2016 |
| North Korea | 3.89 | 3.84 | 3.95 | 2017 |
| North Korea | 3.87 | 3.8 | 3.94 | 2018 |
| North Korea | 3.85 | 3.76 | 3.95 | 2019 |
| North Korea | 3.84 | 3.71 | 3.96 | 2020 |
| North Korea | 3.82 | 3.66 | 3.97 | 2021 |
| North Korea | 3.8 | 3.61 | 3.98 | 2022 |
| North Korea | 3.78 | 3.56 | 4 | 2023 |
| North Korea | 3.77 | 3.51 | 4.03 | 2024 |
| North Korea | 3.76 | 3.46 | 4.06 | 2025 |
| North Korea | 3.75 | 3.41 | 4.09 | 2026 |
| North Korea | 3.74 | 3.36 | 4.12 | 2027 |
| North Korea | 3.73 | 3.31 | 4.16 | 2028 |
| North Korea | 3.73 | 3.26 | 4.2 | 2029 |
| North Korea | 3.73 | 3.21 | 4.25 | 2030 |
| Norway | 7.3 | 7.15 | 7.46 | 1990 |
| Norway | 7.5 | 7.37 | 7.62 | 1991 |
| Norway | 7.69 | 7.59 | 7.8 | 1992 |
| Norway | 7.9 | 7.8 | 8 | 1993 |
| Norway | 8.12 | 8.02 | 8.22 | 1994 |
| Norway | 8.35 | 8.25 | 8.45 | 1995 |
| Norway | 8.6 | 8.5 | 8.71 | 1996 |
| Norway | 8.88 | 8.78 | 8.99 | 1997 |
| Norway | 9.19 | 9.08 | 9.29 | 1998 |
| Norway | 9.5 | 9.39 | 9.61 | 1999 |
| Norway | 9.82 | 9.71 | 9.92 | 2000 |
| Norway | 10.12 | 10.01 | 10.23 | 2001 |
| Norway | 10.41 | 10.3 | 10.52 | 2002 |
| Norway | 10.69 | 10.57 | 10.8 | 2003 |
| Norway | 10.95 | 10.84 | 11.07 | 2004 |
| Norway | 11.21 | 11.09 | 11.32 | 2005 |
| Norway | 11.45 | 11.34 | 11.57 | 2006 |
| Norway | 11.69 | 11.57 | 11.81 | 2007 |
| Norway | 11.9 | 11.79 | 12.02 | 2008 |
| Norway | 12.08 | 11.96 | 12.2 | 2009 |
| Norway | 12.19 | 12.07 | 12.31 | 2010 |
| Norway | 12.22 | 12.1 | 12.34 | 2011 |
| Norway | 12.15 | 12.03 | 12.27 | 2012 |
| Norway | 12.01 | 11.89 | 12.13 | 2013 |
| Norway | 11.82 | 11.7 | 11.93 | 2014 |
| Norway | 11.59 | 11.48 | 11.71 | 2015 |
| Norway | 11.37 | 11.24 | 11.5 | 2016 |
| Norway | 11.15 | 10.97 | 11.33 | 2017 |
| Norway | 10.93 | 10.68 | 11.18 | 2018 |
| Norway | 10.71 | 10.37 | 11.05 | 2019 |
| Norway | 10.49 | 10.05 | 10.93 | 2020 |
| Norway | 10.27 | 9.71 | 10.82 | 2021 |
| Norway | 10.05 | 9.38 | 10.72 | 2022 |
| Norway | 9.83 | 9.03 | 10.62 | 2023 |
| Norway | 9.61 | 8.69 | 10.52 | 2024 |
| Norway | 9.39 | 8.35 | 10.43 | 2025 |
| Norway | 9.17 | 8 | 10.34 | 2026 |
| Norway | 8.95 | 7.66 | 10.25 | 2027 |
| Norway | 8.74 | 7.32 | 10.16 | 2028 |
| Norway | 8.52 | 6.98 | 10.07 | 2029 |
| Norway | 8.31 | 6.64 | 9.98 | 2030 |
| Oman | 1.48 | 1.35 | 1.6 | 1990 |
| Oman | 1.51 | 1.4 | 1.63 | 1991 |
| Oman | 1.55 | 1.44 | 1.65 | 1992 |
| Oman | 1.59 | 1.49 | 1.68 | 1993 |
| Oman | 1.63 | 1.54 | 1.72 | 1994 |
| Oman | 1.67 | 1.58 | 1.75 | 1995 |
| Oman | 1.71 | 1.63 | 1.79 | 1996 |
| Oman | 1.75 | 1.67 | 1.83 | 1997 |
| Oman | 1.79 | 1.72 | 1.87 | 1998 |
| Oman | 1.84 | 1.76 | 1.91 | 1999 |
| Oman | 1.88 | 1.81 | 1.96 | 2000 |
| Oman | 1.93 | 1.85 | 2 | 2001 |
| Oman | 1.97 | 1.9 | 2.05 | 2002 |
| Oman | 2.02 | 1.94 | 2.09 | 2003 |
| Oman | 2.07 | 1.99 | 2.14 | 2004 |
| Oman | 2.12 | 2.04 | 2.19 | 2005 |
| Oman | 2.17 | 2.09 | 2.24 | 2006 |
| Oman | 2.22 | 2.14 | 2.3 | 2007 |
| Oman | 2.27 | 2.2 | 2.35 | 2008 |
| Oman | 2.33 | 2.25 | 2.4 | 2009 |
| Oman | 2.38 | 2.31 | 2.46 | 2010 |
| Oman | 2.44 | 2.36 | 2.52 | 2011 |
| Oman | 2.5 | 2.41 | 2.58 | 2012 |
| Oman | 2.55 | 2.47 | 2.64 | 2013 |
| Oman | 2.61 | 2.52 | 2.7 | 2014 |
| Oman | 2.66 | 2.56 | 2.76 | 2015 |
| Oman | 2.71 | 2.6 | 2.83 | 2016 |
| Oman | 2.76 | 2.63 | 2.9 | 2017 |
| Oman | 2.82 | 2.66 | 2.97 | 2018 |
| Oman | 2.87 | 2.68 | 3.05 | 2019 |
| Oman | 2.92 | 2.7 | 3.14 | 2020 |
| Oman | 2.97 | 2.71 | 3.23 | 2021 |
| Oman | 3.02 | 2.72 | 3.32 | 2022 |
| Oman | 3.07 | 2.73 | 3.42 | 2023 |
| Oman | 3.12 | 2.73 | 3.52 | 2024 |
| Oman | 3.17 | 2.73 | 3.62 | 2025 |
| Oman | 3.22 | 2.72 | 3.73 | 2026 |
| Oman | 3.27 | 2.71 | 3.84 | 2027 |
| Oman | 3.32 | 2.69 | 3.95 | 2028 |
| Oman | 3.37 | 2.67 | 4.07 | 2029 |
| Oman | 3.42 | 2.65 | 4.19 | 2030 |
| Pakistan | 1.31 | 1.29 | 1.33 | 1990 |
| Pakistan | 1.35 | 1.33 | 1.36 | 1991 |
| Pakistan | 1.38 | 1.37 | 1.4 | 1992 |
| Pakistan | 1.42 | 1.41 | 1.43 | 1993 |
| Pakistan | 1.46 | 1.45 | 1.47 | 1994 |
| Pakistan | 1.5 | 1.49 | 1.51 | 1995 |
| Pakistan | 1.55 | 1.53 | 1.56 | 1996 |
| Pakistan | 1.59 | 1.58 | 1.6 | 1997 |
| Pakistan | 1.64 | 1.63 | 1.65 | 1998 |
| Pakistan | 1.68 | 1.67 | 1.69 | 1999 |
| Pakistan | 1.72 | 1.71 | 1.73 | 2000 |
| Pakistan | 1.76 | 1.74 | 1.77 | 2001 |
| Pakistan | 1.79 | 1.78 | 1.8 | 2002 |
| Pakistan | 1.82 | 1.81 | 1.83 | 2003 |
| Pakistan | 1.85 | 1.84 | 1.86 | 2004 |
| Pakistan | 1.88 | 1.87 | 1.89 | 2005 |
| Pakistan | 1.91 | 1.9 | 1.92 | 2006 |
| Pakistan | 1.95 | 1.93 | 1.96 | 2007 |
| Pakistan | 1.98 | 1.97 | 1.99 | 2008 |
| Pakistan | 2.01 | 2 | 2.03 | 2009 |
| Pakistan | 2.04 | 2.03 | 2.06 | 2010 |
| Pakistan | 2.07 | 2.06 | 2.08 | 2011 |
| Pakistan | 2.1 | 2.08 | 2.11 | 2012 |
| Pakistan | 2.12 | 2.11 | 2.13 | 2013 |
| Pakistan | 2.14 | 2.13 | 2.15 | 2014 |
| Pakistan | 2.16 | 2.15 | 2.17 | 2015 |
| Pakistan | 2.18 | 2.16 | 2.19 | 2016 |
| Pakistan | 2.19 | 2.17 | 2.21 | 2017 |
| Pakistan | 2.21 | 2.18 | 2.24 | 2018 |
| Pakistan | 2.22 | 2.18 | 2.27 | 2019 |
| Pakistan | 2.24 | 2.18 | 2.3 | 2020 |
| Pakistan | 2.26 | 2.18 | 2.33 | 2021 |
| Pakistan | 2.27 | 2.18 | 2.37 | 2022 |
| Pakistan | 2.29 | 2.17 | 2.4 | 2023 |
| Pakistan | 2.3 | 2.17 | 2.44 | 2024 |
| Pakistan | 2.32 | 2.16 | 2.48 | 2025 |
| Pakistan | 2.34 | 2.15 | 2.52 | 2026 |
| Pakistan | 2.35 | 2.14 | 2.57 | 2027 |
| Pakistan | 2.37 | 2.12 | 2.61 | 2028 |
| Pakistan | 2.39 | 2.11 | 2.66 | 2029 |
| Pakistan | 2.4 | 2.1 | 2.71 | 2030 |
| Palestine | 3.91 | 3.68 | 4.13 | 1990 |
| Palestine | 3.88 | 3.69 | 4.07 | 1991 |
| Palestine | 3.86 | 3.7 | 4.03 | 1992 |
| Palestine | 3.85 | 3.7 | 3.99 | 1993 |
| Palestine | 3.83 | 3.7 | 3.96 | 1994 |
| Palestine | 3.82 | 3.69 | 3.94 | 1995 |
| Palestine | 3.81 | 3.69 | 3.93 | 1996 |
| Palestine | 3.81 | 3.7 | 3.92 | 1997 |
| Palestine | 3.82 | 3.71 | 3.92 | 1998 |
| Palestine | 3.83 | 3.73 | 3.94 | 1999 |
| Palestine | 3.86 | 3.75 | 3.96 | 2000 |
| Palestine | 3.89 | 3.79 | 3.99 | 2001 |
| Palestine | 3.93 | 3.83 | 4.03 | 2002 |
| Palestine | 3.98 | 3.88 | 4.08 | 2003 |
| Palestine | 4.03 | 3.93 | 4.13 | 2004 |
| Palestine | 4.09 | 3.99 | 4.19 | 2005 |
| Palestine | 4.15 | 4.05 | 4.25 | 2006 |
| Palestine | 4.21 | 4.11 | 4.31 | 2007 |
| Palestine | 4.27 | 4.17 | 4.37 | 2008 |
| Palestine | 4.33 | 4.23 | 4.43 | 2009 |
| Palestine | 4.38 | 4.28 | 4.48 | 2010 |
| Palestine | 4.44 | 4.34 | 4.53 | 2011 |
| Palestine | 4.49 | 4.39 | 4.59 | 2012 |
| Palestine | 4.53 | 4.43 | 4.63 | 2013 |
| Palestine | 4.58 | 4.47 | 4.68 | 2014 |
| Palestine | 4.62 | 4.5 | 4.74 | 2015 |
| Palestine | 4.66 | 4.52 | 4.79 | 2016 |
| Palestine | 4.69 | 4.53 | 4.86 | 2017 |
| Palestine | 4.73 | 4.53 | 4.93 | 2018 |
| Palestine | 4.77 | 4.53 | 5.01 | 2019 |
| Palestine | 4.81 | 4.51 | 5.1 | 2020 |
| Palestine | 4.85 | 4.49 | 5.2 | 2021 |
| Palestine | 4.89 | 4.47 | 5.3 | 2022 |
| Palestine | 4.92 | 4.44 | 5.41 | 2023 |
| Palestine | 4.96 | 4.4 | 5.52 | 2024 |
| Palestine | 5 | 4.36 | 5.63 | 2025 |
| Palestine | 5.04 | 4.32 | 5.75 | 2026 |
| Palestine | 5.07 | 4.27 | 5.88 | 2027 |
| Palestine | 5.11 | 4.21 | 6 | 2028 |
| Palestine | 5.14 | 4.15 | 6.13 | 2029 |
| Palestine | 5.18 | 4.09 | 6.26 | 2030 |
| Panama | 2.02 | 1.91 | 2.13 | 1990 |
| Panama | 2.07 | 1.97 | 2.17 | 1991 |
| Panama | 2.12 | 2.04 | 2.21 | 1992 |
| Panama | 2.18 | 2.1 | 2.26 | 1993 |
| Panama | 2.24 | 2.17 | 2.31 | 1994 |
| Panama | 2.3 | 2.23 | 2.37 | 1995 |
| Panama | 2.37 | 2.3 | 2.44 | 1996 |
| Panama | 2.45 | 2.38 | 2.51 | 1997 |
| Panama | 2.52 | 2.46 | 2.59 | 1998 |
| Panama | 2.6 | 2.54 | 2.67 | 1999 |
| Panama | 2.69 | 2.62 | 2.76 | 2000 |
| Panama | 2.78 | 2.71 | 2.85 | 2001 |
| Panama | 2.88 | 2.81 | 2.94 | 2002 |
| Panama | 2.97 | 2.91 | 3.04 | 2003 |
| Panama | 3.08 | 3.01 | 3.14 | 2004 |
| Panama | 3.18 | 3.11 | 3.25 | 2005 |
| Panama | 3.29 | 3.22 | 3.36 | 2006 |
| Panama | 3.4 | 3.33 | 3.47 | 2007 |
| Panama | 3.52 | 3.45 | 3.59 | 2008 |
| Panama | 3.64 | 3.57 | 3.71 | 2009 |
| Panama | 3.77 | 3.69 | 3.84 | 2010 |
| Panama | 3.89 | 3.82 | 3.97 | 2011 |
| Panama | 4.02 | 3.95 | 4.1 | 2012 |
| Panama | 4.15 | 4.07 | 4.23 | 2013 |
| Panama | 4.28 | 4.19 | 4.36 | 2014 |
| Panama | 4.4 | 4.3 | 4.49 | 2015 |
| Panama | 4.52 | 4.41 | 4.63 | 2016 |
| Panama | 4.64 | 4.5 | 4.78 | 2017 |
| Panama | 4.76 | 4.59 | 4.94 | 2018 |
| Panama | 4.89 | 4.67 | 5.11 | 2019 |
| Panama | 5.02 | 4.75 | 5.3 | 2020 |
| Panama | 5.16 | 4.82 | 5.5 | 2021 |
| Panama | 5.29 | 4.88 | 5.7 | 2022 |
| Panama | 5.43 | 4.94 | 5.92 | 2023 |
| Panama | 5.58 | 5 | 6.15 | 2024 |
| Panama | 5.72 | 5.05 | 6.39 | 2025 |
| Panama | 5.87 | 5.1 | 6.65 | 2026 |
| Panama | 6.03 | 5.14 | 6.91 | 2027 |
| Panama | 6.18 | 5.18 | 7.19 | 2028 |
| Panama | 6.35 | 5.21 | 7.48 | 2029 |
| Panama | 6.51 | 5.24 | 7.78 | 2030 |
| Papua New Guinea | 2.36 | 2.26 | 2.46 | 1990 |
| Papua New Guinea | 2.35 | 2.27 | 2.44 | 1991 |
| Papua New Guinea | 2.35 | 2.27 | 2.43 | 1992 |
| Papua New Guinea | 2.34 | 2.28 | 2.41 | 1993 |
| Papua New Guinea | 2.34 | 2.28 | 2.4 | 1994 |
| Papua New Guinea | 2.34 | 2.28 | 2.4 | 1995 |
| Papua New Guinea | 2.34 | 2.29 | 2.4 | 1996 |
| Papua New Guinea | 2.35 | 2.29 | 2.4 | 1997 |
| Papua New Guinea | 2.36 | 2.3 | 2.41 | 1998 |
| Papua New Guinea | 2.37 | 2.32 | 2.42 | 1999 |
| Papua New Guinea | 2.38 | 2.33 | 2.44 | 2000 |
| Papua New Guinea | 2.4 | 2.35 | 2.45 | 2001 |
| Papua New Guinea | 2.42 | 2.37 | 2.47 | 2002 |
| Papua New Guinea | 2.45 | 2.4 | 2.5 | 2003 |
| Papua New Guinea | 2.47 | 2.42 | 2.52 | 2004 |
| Papua New Guinea | 2.5 | 2.45 | 2.55 | 2005 |
| Papua New Guinea | 2.52 | 2.47 | 2.57 | 2006 |
| Papua New Guinea | 2.55 | 2.5 | 2.6 | 2007 |
| Papua New Guinea | 2.57 | 2.52 | 2.62 | 2008 |
| Papua New Guinea | 2.6 | 2.55 | 2.65 | 2009 |
| Papua New Guinea | 2.62 | 2.57 | 2.67 | 2010 |
| Papua New Guinea | 2.64 | 2.59 | 2.69 | 2011 |
| Papua New Guinea | 2.66 | 2.61 | 2.71 | 2012 |
| Papua New Guinea | 2.68 | 2.63 | 2.73 | 2013 |
| Papua New Guinea | 2.7 | 2.64 | 2.75 | 2014 |
| Papua New Guinea | 2.72 | 2.66 | 2.78 | 2015 |
| Papua New Guinea | 2.74 | 2.67 | 2.8 | 2016 |
| Papua New Guinea | 2.76 | 2.67 | 2.84 | 2017 |
| Papua New Guinea | 2.78 | 2.67 | 2.88 | 2018 |
| Papua New Guinea | 2.8 | 2.67 | 2.92 | 2019 |
| Papua New Guinea | 2.82 | 2.67 | 2.97 | 2020 |
| Papua New Guinea | 2.84 | 2.66 | 3.02 | 2021 |
| Papua New Guinea | 2.86 | 2.65 | 3.08 | 2022 |
| Papua New Guinea | 2.89 | 2.64 | 3.14 | 2023 |
| Papua New Guinea | 2.91 | 2.62 | 3.2 | 2024 |
| Papua New Guinea | 2.94 | 2.61 | 3.27 | 2025 |
| Papua New Guinea | 2.97 | 2.59 | 3.34 | 2026 |
| Papua New Guinea | 2.99 | 2.57 | 3.42 | 2027 |
| Papua New Guinea | 3.02 | 2.55 | 3.5 | 2028 |
| Papua New Guinea | 3.05 | 2.53 | 3.58 | 2029 |
| Papua New Guinea | 3.08 | 2.5 | 3.66 | 2030 |
| Paraguay | 4.36 | 4.21 | 4.51 | 1990 |
| Paraguay | 4.35 | 4.23 | 4.48 | 1991 |
| Paraguay | 4.35 | 4.24 | 4.46 | 1992 |
| Paraguay | 4.35 | 4.25 | 4.44 | 1993 |
| Paraguay | 4.34 | 4.25 | 4.43 | 1994 |
| Paraguay | 4.33 | 4.25 | 4.42 | 1995 |
| Paraguay | 4.32 | 4.24 | 4.4 | 1996 |
| Paraguay | 4.31 | 4.24 | 4.39 | 1997 |
| Paraguay | 4.31 | 4.23 | 4.38 | 1998 |
| Paraguay | 4.31 | 4.23 | 4.38 | 1999 |
| Paraguay | 4.31 | 4.24 | 4.39 | 2000 |
| Paraguay | 4.32 | 4.25 | 4.39 | 2001 |
| Paraguay | 4.33 | 4.26 | 4.4 | 2002 |
| Paraguay | 4.34 | 4.27 | 4.41 | 2003 |
| Paraguay | 4.36 | 4.28 | 4.43 | 2004 |
| Paraguay | 4.37 | 4.29 | 4.44 | 2005 |
| Paraguay | 4.37 | 4.3 | 4.44 | 2006 |
| Paraguay | 4.38 | 4.31 | 4.45 | 2007 |
| Paraguay | 4.39 | 4.32 | 4.46 | 2008 |
| Paraguay | 4.4 | 4.33 | 4.47 | 2009 |
| Paraguay | 4.41 | 4.34 | 4.48 | 2010 |
| Paraguay | 4.43 | 4.36 | 4.5 | 2011 |
| Paraguay | 4.45 | 4.38 | 4.51 | 2012 |
| Paraguay | 4.47 | 4.4 | 4.54 | 2013 |
| Paraguay | 4.49 | 4.42 | 4.56 | 2014 |
| Paraguay | 4.51 | 4.43 | 4.59 | 2015 |
| Paraguay | 4.53 | 4.44 | 4.63 | 2016 |
| Paraguay | 4.55 | 4.44 | 4.67 | 2017 |
| Paraguay | 4.58 | 4.43 | 4.72 | 2018 |
| Paraguay | 4.59 | 4.41 | 4.77 | 2019 |
| Paraguay | 4.61 | 4.39 | 4.83 | 2020 |
| Paraguay | 4.63 | 4.36 | 4.89 | 2021 |
| Paraguay | 4.64 | 4.33 | 4.96 | 2022 |
| Paraguay | 4.66 | 4.29 | 5.02 | 2023 |
| Paraguay | 4.67 | 4.24 | 5.09 | 2024 |
| Paraguay | 4.68 | 4.19 | 5.16 | 2025 |
| Paraguay | 4.68 | 4.14 | 5.23 | 2026 |
| Paraguay | 4.69 | 4.08 | 5.29 | 2027 |
| Paraguay | 4.69 | 4.02 | 5.36 | 2028 |
| Paraguay | 4.69 | 3.95 | 5.43 | 2029 |
| Paraguay | 4.69 | 3.88 | 5.5 | 2030 |
| Peru | 5.4 | 5.3 | 5.5 | 1990 |
| Peru | 5.61 | 5.54 | 5.68 | 1991 |
| Peru | 5.84 | 5.77 | 5.91 | 1992 |
| Peru | 6.07 | 6 | 6.14 | 1993 |
| Peru | 6.27 | 6.2 | 6.35 | 1994 |
| Peru | 6.41 | 6.33 | 6.48 | 1995 |
| Peru | 6.42 | 6.35 | 6.5 | 1996 |
| Peru | 6.32 | 6.25 | 6.39 | 1997 |
| Peru | 6.11 | 6.04 | 6.18 | 1998 |
| Peru | 5.83 | 5.76 | 5.89 | 1999 |
| Peru | 5.55 | 5.49 | 5.61 | 2000 |
| Peru | 5.32 | 5.26 | 5.38 | 2001 |
| Peru | 5.16 | 5.1 | 5.21 | 2002 |
| Peru | 5.06 | 5 | 5.11 | 2003 |
| Peru | 5 | 4.94 | 5.05 | 2004 |
| Peru | 4.96 | 4.91 | 5.02 | 2005 |
| Peru | 4.95 | 4.9 | 5.01 | 2006 |
| Peru | 4.99 | 4.94 | 5.05 | 2007 |
| Peru | 5.1 | 5.04 | 5.15 | 2008 |
| Peru | 5.23 | 5.18 | 5.29 | 2009 |
| Peru | 5.33 | 5.27 | 5.38 | 2010 |
| Peru | 5.36 | 5.3 | 5.41 | 2011 |
| Peru | 5.34 | 5.29 | 5.4 | 2012 |
| Peru | 5.31 | 5.26 | 5.36 | 2013 |
| Peru | 5.27 | 5.22 | 5.32 | 2014 |
| Peru | 5.25 | 5.19 | 5.3 | 2015 |
| Peru | 5.24 | 5.18 | 5.29 | 2016 |
| Peru | 5.23 | 5.15 | 5.31 | 2017 |
| Peru | 5.22 | 5.08 | 5.36 | 2018 |
| Peru | 5.21 | 4.99 | 5.43 | 2019 |
| Peru | 5.2 | 4.87 | 5.52 | 2020 |
| Peru | 5.18 | 4.74 | 5.63 | 2021 |
| Peru | 5.16 | 4.59 | 5.74 | 2022 |
| Peru | 5.15 | 4.44 | 5.86 | 2023 |
| Peru | 5.12 | 4.27 | 5.98 | 2024 |
| Peru | 5.1 | 4.09 | 6.11 | 2025 |
| Peru | 5.07 | 3.9 | 6.24 | 2026 |
| Peru | 5.04 | 3.71 | 6.38 | 2027 |
| Peru | 5.01 | 3.51 | 6.52 | 2028 |
| Peru | 4.98 | 3.3 | 6.66 | 2029 |
| Peru | 4.94 | 3.08 | 6.81 | 2030 |
| Philippines | 3.25 | 3.19 | 3.3 | 1990 |
| Philippines | 3.28 | 3.24 | 3.32 | 1991 |
| Philippines | 3.3 | 3.26 | 3.34 | 1992 |
| Philippines | 3.3 | 3.26 | 3.34 | 1993 |
| Philippines | 3.26 | 3.22 | 3.3 | 1994 |
| Philippines | 3.18 | 3.14 | 3.21 | 1995 |
| Philippines | 3.05 | 3.02 | 3.08 | 1996 |
| Philippines | 2.95 | 2.91 | 2.98 | 1997 |
| Philippines | 3.01 | 2.97 | 3.04 | 1998 |
| Philippines | 3.25 | 3.21 | 3.28 | 1999 |
| Philippines | 3.48 | 3.45 | 3.52 | 2000 |
| Philippines | 3.67 | 3.64 | 3.71 | 2001 |
| Philippines | 3.82 | 3.78 | 3.85 | 2002 |
| Philippines | 3.91 | 3.87 | 3.94 | 2003 |
| Philippines | 3.96 | 3.92 | 3.99 | 2004 |
| Philippines | 4 | 3.97 | 4.04 | 2005 |
| Philippines | 4.03 | 4 | 4.07 | 2006 |
| Philippines | 4.03 | 3.99 | 4.07 | 2007 |
| Philippines | 4.02 | 3.98 | 4.05 | 2008 |
| Philippines | 4.02 | 3.99 | 4.06 | 2009 |
| Philippines | 4.08 | 4.05 | 4.12 | 2010 |
| Philippines | 4.18 | 4.14 | 4.21 | 2011 |
| Philippines | 4.29 | 4.26 | 4.33 | 2012 |
| Philippines | 4.35 | 4.32 | 4.39 | 2013 |
| Philippines | 4.38 | 4.35 | 4.42 | 2014 |
| Philippines | 4.4 | 4.37 | 4.44 | 2015 |
| Philippines | 4.38 | 4.34 | 4.41 | 2016 |
| Philippines | 4.32 | 4.28 | 4.37 | 2017 |
| Philippines | 4.27 | 4.16 | 4.39 | 2018 |
| Philippines | 4.22 | 4.01 | 4.43 | 2019 |
| Philippines | 4.17 | 3.84 | 4.5 | 2020 |
| Philippines | 4.13 | 3.66 | 4.59 | 2021 |
| Philippines | 4.08 | 3.47 | 4.68 | 2022 |
| Philippines | 4.03 | 3.28 | 4.79 | 2023 |
| Philippines | 3.99 | 3.07 | 4.91 | 2024 |
| Philippines | 3.95 | 2.86 | 5.03 | 2025 |
| Philippines | 3.9 | 2.64 | 5.17 | 2026 |
| Philippines | 3.86 | 2.41 | 5.31 | 2027 |
| Philippines | 3.82 | 2.18 | 5.46 | 2028 |
| Philippines | 3.78 | 1.95 | 5.61 | 2029 |
| Philippines | 3.74 | 1.72 | 5.77 | 2030 |
| Poland | 7.14 | 7.08 | 7.21 | 1990 |
| Poland | 7.19 | 7.14 | 7.23 | 1991 |
| Poland | 7.22 | 7.17 | 7.26 | 1992 |
| Poland | 7.24 | 7.2 | 7.28 | 1993 |
| Poland | 7.27 | 7.23 | 7.31 | 1994 |
| Poland | 7.32 | 7.27 | 7.36 | 1995 |
| Poland | 7.38 | 7.34 | 7.42 | 1996 |
| Poland | 7.46 | 7.42 | 7.5 | 1997 |
| Poland | 7.56 | 7.52 | 7.6 | 1998 |
| Poland | 7.66 | 7.62 | 7.7 | 1999 |
| Poland | 7.76 | 7.71 | 7.8 | 2000 |
| Poland | 7.84 | 7.8 | 7.88 | 2001 |
| Poland | 7.92 | 7.88 | 7.96 | 2002 |
| Poland | 8.01 | 7.97 | 8.05 | 2003 |
| Poland | 8.1 | 8.06 | 8.15 | 2004 |
| Poland | 8.21 | 8.17 | 8.25 | 2005 |
| Poland | 8.33 | 8.29 | 8.37 | 2006 |
| Poland | 8.45 | 8.41 | 8.5 | 2007 |
| Poland | 8.56 | 8.52 | 8.6 | 2008 |
| Poland | 8.64 | 8.6 | 8.68 | 2009 |
| Poland | 8.68 | 8.64 | 8.72 | 2010 |
| Poland | 8.68 | 8.64 | 8.73 | 2011 |
| Poland | 8.66 | 8.62 | 8.7 | 2012 |
| Poland | 8.6 | 8.56 | 8.65 | 2013 |
| Poland | 8.52 | 8.48 | 8.57 | 2014 |
| Poland | 8.44 | 8.4 | 8.48 | 2015 |
| Poland | 8.34 | 8.3 | 8.39 | 2016 |
| Poland | 8.24 | 8.18 | 8.3 | 2017 |
| Poland | 8.14 | 8.04 | 8.24 | 2018 |
| Poland | 8.04 | 7.9 | 8.18 | 2019 |
| Poland | 7.94 | 7.75 | 8.14 | 2020 |
| Poland | 7.85 | 7.59 | 8.11 | 2021 |
| Poland | 7.75 | 7.43 | 8.07 | 2022 |
| Poland | 7.65 | 7.26 | 8.04 | 2023 |
| Poland | 7.55 | 7.08 | 8.01 | 2024 |
| Poland | 7.45 | 6.91 | 7.99 | 2025 |
| Poland | 7.35 | 6.73 | 7.96 | 2026 |
| Poland | 7.25 | 6.56 | 7.94 | 2027 |
| Poland | 7.15 | 6.38 | 7.93 | 2028 |
| Poland | 7.06 | 6.2 | 7.92 | 2029 |
| Poland | 6.97 | 6.03 | 7.91 | 2030 |
| Portugal | 6.16 | 6.05 | 6.28 | 1990 |
| Portugal | 6.34 | 6.26 | 6.43 | 1991 |
| Portugal | 6.51 | 6.43 | 6.59 | 1992 |
| Portugal | 6.66 | 6.58 | 6.73 | 1993 |
| Portugal | 6.78 | 6.71 | 6.86 | 1994 |
| Portugal | 6.89 | 6.82 | 6.97 | 1995 |
| Portugal | 6.96 | 6.88 | 7.04 | 1996 |
| Portugal | 6.97 | 6.9 | 7.05 | 1997 |
| Portugal | 6.93 | 6.85 | 7 | 1998 |
| Portugal | 6.83 | 6.76 | 6.91 | 1999 |
| Portugal | 6.74 | 6.67 | 6.82 | 2000 |
| Portugal | 6.71 | 6.63 | 6.79 | 2001 |
| Portugal | 6.81 | 6.73 | 6.88 | 2002 |
| Portugal | 6.96 | 6.89 | 7.03 | 2003 |
| Portugal | 7.12 | 7.05 | 7.19 | 2004 |
| Portugal | 7.26 | 7.19 | 7.34 | 2005 |
| Portugal | 7.37 | 7.3 | 7.45 | 2006 |
| Portugal | 7.45 | 7.38 | 7.52 | 2007 |
| Portugal | 7.49 | 7.42 | 7.56 | 2008 |
| Portugal | 7.5 | 7.43 | 7.58 | 2009 |
| Portugal | 7.48 | 7.41 | 7.55 | 2010 |
| Portugal | 7.41 | 7.34 | 7.49 | 2011 |
| Portugal | 7.32 | 7.25 | 7.39 | 2012 |
| Portugal | 7.2 | 7.13 | 7.27 | 2013 |
| Portugal | 7.07 | 7 | 7.14 | 2014 |
| Portugal | 6.95 | 6.88 | 7.02 | 2015 |
| Portugal | 6.86 | 6.78 | 6.94 | 2016 |
| Portugal | 6.78 | 6.67 | 6.88 | 2017 |
| Portugal | 6.69 | 6.53 | 6.86 | 2018 |
| Portugal | 6.61 | 6.36 | 6.86 | 2019 |
| Portugal | 6.52 | 6.18 | 6.87 | 2020 |
| Portugal | 6.43 | 5.98 | 6.89 | 2021 |
| Portugal | 6.34 | 5.78 | 6.91 | 2022 |
| Portugal | 6.25 | 5.57 | 6.94 | 2023 |
| Portugal | 6.16 | 5.35 | 6.97 | 2024 |
| Portugal | 6.06 | 5.12 | 7 | 2025 |
| Portugal | 5.96 | 4.89 | 7.04 | 2026 |
| Portugal | 5.86 | 4.66 | 7.07 | 2027 |
| Portugal | 5.76 | 4.42 | 7.1 | 2028 |
| Portugal | 5.65 | 4.18 | 7.13 | 2029 |
| Portugal | 5.55 | 3.93 | 7.16 | 2030 |
| Puerto Rico | 4.8 | 4.64 | 4.95 | 1990 |
| Puerto Rico | 4.7 | 4.58 | 4.82 | 1991 |
| Puerto Rico | 4.61 | 4.51 | 4.71 | 1992 |
| Puerto Rico | 4.51 | 4.43 | 4.6 | 1993 |
| Puerto Rico | 4.43 | 4.34 | 4.51 | 1994 |
| Puerto Rico | 4.35 | 4.27 | 4.43 | 1995 |
| Puerto Rico | 4.28 | 4.21 | 4.36 | 1996 |
| Puerto Rico | 4.23 | 4.16 | 4.31 | 1997 |
| Puerto Rico | 4.2 | 4.13 | 4.28 | 1998 |
| Puerto Rico | 4.19 | 4.12 | 4.27 | 1999 |
| Puerto Rico | 4.2 | 4.12 | 4.27 | 2000 |
| Puerto Rico | 4.21 | 4.14 | 4.29 | 2001 |
| Puerto Rico | 4.25 | 4.17 | 4.32 | 2002 |
| Puerto Rico | 4.29 | 4.22 | 4.37 | 2003 |
| Puerto Rico | 4.35 | 4.27 | 4.42 | 2004 |
| Puerto Rico | 4.4 | 4.33 | 4.48 | 2005 |
| Puerto Rico | 4.45 | 4.38 | 4.53 | 2006 |
| Puerto Rico | 4.5 | 4.43 | 4.58 | 2007 |
| Puerto Rico | 4.54 | 4.47 | 4.62 | 2008 |
| Puerto Rico | 4.57 | 4.5 | 4.65 | 2009 |
| Puerto Rico | 4.6 | 4.52 | 4.68 | 2010 |
| Puerto Rico | 4.62 | 4.54 | 4.7 | 2011 |
| Puerto Rico | 4.64 | 4.56 | 4.71 | 2012 |
| Puerto Rico | 4.65 | 4.57 | 4.73 | 2013 |
| Puerto Rico | 4.66 | 4.58 | 4.75 | 2014 |
| Puerto Rico | 4.68 | 4.59 | 4.77 | 2015 |
| Puerto Rico | 4.7 | 4.59 | 4.8 | 2016 |
| Puerto Rico | 4.72 | 4.59 | 4.85 | 2017 |
| Puerto Rico | 4.73 | 4.56 | 4.9 | 2018 |
| Puerto Rico | 4.74 | 4.53 | 4.96 | 2019 |
| Puerto Rico | 4.75 | 4.48 | 5.03 | 2020 |
| Puerto Rico | 4.76 | 4.43 | 5.1 | 2021 |
| Puerto Rico | 4.77 | 4.37 | 5.17 | 2022 |
| Puerto Rico | 4.78 | 4.31 | 5.24 | 2023 |
| Puerto Rico | 4.78 | 4.23 | 5.32 | 2024 |
| Puerto Rico | 4.78 | 4.16 | 5.4 | 2025 |
| Puerto Rico | 4.78 | 4.07 | 5.48 | 2026 |
| Puerto Rico | 4.77 | 3.98 | 5.57 | 2027 |
| Puerto Rico | 4.77 | 3.89 | 5.65 | 2028 |
| Puerto Rico | 4.76 | 3.79 | 5.73 | 2029 |
| Puerto Rico | 4.75 | 3.68 | 5.82 | 2030 |
| Qatar | 4.78 | 4.21 | 5.34 | 1990 |
| Qatar | 4.76 | 4.26 | 5.27 | 1991 |
| Qatar | 4.75 | 4.29 | 5.2 | 1992 |
| Qatar | 4.72 | 4.31 | 5.14 | 1993 |
| Qatar | 4.7 | 4.33 | 5.07 | 1994 |
| Qatar | 4.67 | 4.33 | 5.01 | 1995 |
| Qatar | 4.63 | 4.32 | 4.95 | 1996 |
| Qatar | 4.59 | 4.29 | 4.89 | 1997 |
| Qatar | 4.54 | 4.26 | 4.82 | 1998 |
| Qatar | 4.48 | 4.22 | 4.75 | 1999 |
| Qatar | 4.41 | 4.16 | 4.67 | 2000 |
| Qatar | 4.34 | 4.1 | 4.58 | 2001 |
| Qatar | 4.26 | 4.03 | 4.49 | 2002 |
| Qatar | 4.18 | 3.95 | 4.4 | 2003 |
| Qatar | 4.09 | 3.87 | 4.3 | 2004 |
| Qatar | 3.99 | 3.78 | 4.2 | 2005 |
| Qatar | 3.89 | 3.69 | 4.09 | 2006 |
| Qatar | 3.79 | 3.6 | 3.98 | 2007 |
| Qatar | 3.68 | 3.5 | 3.87 | 2008 |
| Qatar | 3.58 | 3.4 | 3.76 | 2009 |
| Qatar | 3.48 | 3.31 | 3.65 | 2010 |
| Qatar | 3.38 | 3.22 | 3.55 | 2011 |
| Qatar | 3.29 | 3.13 | 3.46 | 2012 |
| Qatar | 3.21 | 3.04 | 3.37 | 2013 |
| Qatar | 3.13 | 2.96 | 3.29 | 2014 |
| Qatar | 3.05 | 2.88 | 3.22 | 2015 |
| Qatar | 2.98 | 2.8 | 3.16 | 2016 |
| Qatar | 2.91 | 2.71 | 3.11 | 2017 |
| Qatar | 2.84 | 2.63 | 3.06 | 2018 |
| Qatar | 2.78 | 2.54 | 3.02 | 2019 |
| Qatar | 2.72 | 2.46 | 2.99 | 2020 |
| Qatar | 2.67 | 2.37 | 2.96 | 2021 |
| Qatar | 2.61 | 2.29 | 2.94 | 2022 |
| Qatar | 2.57 | 2.21 | 2.93 | 2023 |
| Qatar | 2.52 | 2.13 | 2.92 | 2024 |
| Qatar | 2.48 | 2.05 | 2.91 | 2025 |
| Qatar | 2.44 | 1.97 | 2.91 | 2026 |
| Qatar | 2.41 | 1.9 | 2.91 | 2027 |
| Qatar | 2.38 | 1.83 | 2.92 | 2028 |
| Qatar | 2.35 | 1.76 | 2.94 | 2029 |
| Qatar | 2.33 | 1.7 | 2.96 | 2030 |
| Romania | 5.68 | 5.61 | 5.75 | 1990 |
| Romania | 5.78 | 5.73 | 5.84 | 1991 |
| Romania | 5.88 | 5.83 | 5.93 | 1992 |
| Romania | 5.97 | 5.93 | 6.02 | 1993 |
| Romania | 6.05 | 6 | 6.1 | 1994 |
| Romania | 6.1 | 6.05 | 6.15 | 1995 |
| Romania | 6.12 | 6.07 | 6.17 | 1996 |
| Romania | 6.1 | 6.06 | 6.15 | 1997 |
| Romania | 6.07 | 6.02 | 6.11 | 1998 |
| Romania | 6.03 | 5.99 | 6.08 | 1999 |
| Romania | 6.03 | 5.98 | 6.08 | 2000 |
| Romania | 6.06 | 6.02 | 6.11 | 2001 |
| Romania | 6.13 | 6.08 | 6.17 | 2002 |
| Romania | 6.21 | 6.16 | 6.25 | 2003 |
| Romania | 6.29 | 6.25 | 6.34 | 2004 |
| Romania | 6.38 | 6.34 | 6.43 | 2005 |
| Romania | 6.48 | 6.43 | 6.52 | 2006 |
| Romania | 6.59 | 6.54 | 6.63 | 2007 |
| Romania | 6.71 | 6.66 | 6.75 | 2008 |
| Romania | 6.82 | 6.77 | 6.87 | 2009 |
| Romania | 6.91 | 6.86 | 6.96 | 2010 |
| Romania | 6.98 | 6.94 | 7.03 | 2011 |
| Romania | 7.05 | 7 | 7.1 | 2012 |
| Romania | 7.11 | 7.06 | 7.16 | 2013 |
| Romania | 7.16 | 7.11 | 7.21 | 2014 |
| Romania | 7.2 | 7.15 | 7.25 | 2015 |
| Romania | 7.24 | 7.18 | 7.3 | 2016 |
| Romania | 7.28 | 7.2 | 7.36 | 2017 |
| Romania | 7.31 | 7.19 | 7.43 | 2018 |
| Romania | 7.34 | 7.16 | 7.52 | 2019 |
| Romania | 7.36 | 7.12 | 7.61 | 2020 |
| Romania | 7.39 | 7.06 | 7.71 | 2021 |
| Romania | 7.41 | 7 | 7.81 | 2022 |
| Romania | 7.42 | 6.93 | 7.92 | 2023 |
| Romania | 7.44 | 6.84 | 8.03 | 2024 |
| Romania | 7.44 | 6.74 | 8.14 | 2025 |
| Romania | 7.45 | 6.64 | 8.25 | 2026 |
| Romania | 7.45 | 6.53 | 8.37 | 2027 |
| Romania | 7.44 | 6.4 | 8.48 | 2028 |
| Romania | 7.44 | 6.28 | 8.6 | 2029 |
| Romania | 7.43 | 6.14 | 8.71 | 2030 |
| Russian Federation | 10.23 | 10.17 | 10.28 | 1990 |
| Russian Federation | 10.43 | 10.38 | 10.48 | 1991 |
| Russian Federation | 11.17 | 11.12 | 11.22 | 1992 |
| Russian Federation | 12.5 | 12.44 | 12.55 | 1993 |
| Russian Federation | 13.17 | 13.11 | 13.22 | 1994 |
| Russian Federation | 12.71 | 12.66 | 12.76 | 1995 |
| Russian Federation | 11.93 | 11.88 | 11.98 | 1996 |
| Russian Federation | 11.39 | 11.34 | 11.44 | 1997 |
| Russian Federation | 11.35 | 11.3 | 11.4 | 1998 |
| Russian Federation | 11.66 | 11.61 | 11.71 | 1999 |
| Russian Federation | 11.77 | 11.72 | 11.82 | 2000 |
| Russian Federation | 11.72 | 11.67 | 11.77 | 2001 |
| Russian Federation | 11.82 | 11.77 | 11.87 | 2002 |
| Russian Federation | 12 | 11.95 | 12.06 | 2003 |
| Russian Federation | 11.97 | 11.92 | 12.02 | 2004 |
| Russian Federation | 12.19 | 12.14 | 12.24 | 2005 |
| Russian Federation | 11.45 | 11.4 | 11.5 | 2006 |
| Russian Federation | 11 | 10.95 | 11.05 | 2007 |
| Russian Federation | 11.17 | 11.12 | 11.22 | 2008 |
| Russian Federation | 11.16 | 11.11 | 11.21 | 2009 |
| Russian Federation | 11.28 | 11.24 | 11.33 | 2010 |
| Russian Federation | 10.79 | 10.75 | 10.84 | 2011 |
| Russian Federation | 10.73 | 10.68 | 10.77 | 2012 |
| Russian Federation | 10.83 | 10.78 | 10.87 | 2013 |
| Russian Federation | 11.11 | 11.07 | 11.16 | 2014 |
| Russian Federation | 10.98 | 10.93 | 11.03 | 2015 |
| Russian Federation | 10.74 | 10.7 | 10.79 | 2016 |
| Russian Federation | 10.62 | 10.57 | 10.67 | 2017 |
| Russian Federation | 10.65 | 10.35 | 10.95 | 2018 |
| Russian Federation | 10.64 | 10 | 11.29 | 2019 |
| Russian Federation | 10.52 | 9.47 | 11.57 | 2020 |
| Russian Federation | 10.32 | 8.82 | 11.81 | 2021 |
| Russian Federation | 10.22 | 8.23 | 12.2 | 2022 |
| Russian Federation | 10.27 | 7.7 | 12.83 | 2023 |
| Russian Federation | 10.26 | 7.09 | 13.43 | 2024 |
| Russian Federation | 10.13 | 6.35 | 13.9 | 2025 |
| Russian Federation | 9.91 | 5.55 | 14.27 | 2026 |
| Russian Federation | 9.79 | 4.79 | 14.79 | 2027 |
| Russian Federation | 9.83 | 4.08 | 15.58 | 2028 |
| Russian Federation | 9.84 | 3.32 | 16.35 | 2029 |
| Russian Federation | 9.75 | 2.5 | 16.99 | 2030 |
| Rwanda | 2.18 | 2.06 | 2.29 | 1990 |
| Rwanda | 2.41 | 2.31 | 2.5 | 1991 |
| Rwanda | 2.66 | 2.57 | 2.74 | 1992 |
| Rwanda | 2.9 | 2.81 | 2.99 | 1993 |
| Rwanda | 3.1 | 3.01 | 3.2 | 1994 |
| Rwanda | 3.23 | 3.13 | 3.33 | 1995 |
| Rwanda | 3.25 | 3.16 | 3.35 | 1996 |
| Rwanda | 3.18 | 3.09 | 3.27 | 1997 |
| Rwanda | 3.02 | 2.93 | 3.1 | 1998 |
| Rwanda | 2.81 | 2.74 | 2.89 | 1999 |
| Rwanda | 2.61 | 2.54 | 2.68 | 2000 |
| Rwanda | 2.44 | 2.37 | 2.5 | 2001 |
| Rwanda | 2.32 | 2.26 | 2.38 | 2002 |
| Rwanda | 2.26 | 2.2 | 2.32 | 2003 |
| Rwanda | 2.24 | 2.18 | 2.3 | 2004 |
| Rwanda | 2.23 | 2.17 | 2.29 | 2005 |
| Rwanda | 2.23 | 2.17 | 2.29 | 2006 |
| Rwanda | 2.22 | 2.16 | 2.28 | 2007 |
| Rwanda | 2.2 | 2.15 | 2.26 | 2008 |
| Rwanda | 2.19 | 2.13 | 2.25 | 2009 |
| Rwanda | 2.17 | 2.12 | 2.23 | 2010 |
| Rwanda | 2.17 | 2.11 | 2.22 | 2011 |
| Rwanda | 2.17 | 2.11 | 2.23 | 2012 |
| Rwanda | 2.19 | 2.13 | 2.25 | 2013 |
| Rwanda | 2.22 | 2.16 | 2.28 | 2014 |
| Rwanda | 2.26 | 2.2 | 2.32 | 2015 |
| Rwanda | 2.29 | 2.23 | 2.36 | 2016 |
| Rwanda | 2.33 | 2.24 | 2.42 | 2017 |
| Rwanda | 2.36 | 2.22 | 2.51 | 2018 |
| Rwanda | 2.4 | 2.18 | 2.61 | 2019 |
| Rwanda | 2.43 | 2.13 | 2.74 | 2020 |
| Rwanda | 2.47 | 2.06 | 2.88 | 2021 |
| Rwanda | 2.5 | 1.97 | 3.04 | 2022 |
| Rwanda | 2.54 | 1.88 | 3.2 | 2023 |
| Rwanda | 2.58 | 1.77 | 3.38 | 2024 |
| Rwanda | 2.61 | 1.65 | 3.58 | 2025 |
| Rwanda | 2.65 | 1.52 | 3.78 | 2026 |
| Rwanda | 2.69 | 1.38 | 4 | 2027 |
| Rwanda | 2.73 | 1.22 | 4.23 | 2028 |
| Rwanda | 2.76 | 1.06 | 4.47 | 2029 |
| Rwanda | 2.8 | 0.88 | 4.73 | 2030 |
| Saint Lucia | 4.99 | 4.33 | 5.65 | 1990 |
| Saint Lucia | 4.91 | 4.33 | 5.5 | 1991 |
| Saint Lucia | 4.83 | 4.31 | 5.36 | 1992 |
| Saint Lucia | 4.75 | 4.28 | 5.22 | 1993 |
| Saint Lucia | 4.67 | 4.24 | 5.1 | 1994 |
| Saint Lucia | 4.59 | 4.19 | 4.99 | 1995 |
| Saint Lucia | 4.51 | 4.14 | 4.89 | 1996 |
| Saint Lucia | 4.44 | 4.09 | 4.79 | 1997 |
| Saint Lucia | 4.38 | 4.04 | 4.71 | 1998 |
| Saint Lucia | 4.32 | 3.99 | 4.65 | 1999 |
| Saint Lucia | 4.27 | 3.96 | 4.59 | 2000 |
| Saint Lucia | 4.24 | 3.93 | 4.55 | 2001 |
| Saint Lucia | 4.21 | 3.91 | 4.51 | 2002 |
| Saint Lucia | 4.19 | 3.9 | 4.49 | 2003 |
| Saint Lucia | 4.18 | 3.89 | 4.47 | 2004 |
| Saint Lucia | 4.16 | 3.88 | 4.45 | 2005 |
| Saint Lucia | 4.15 | 3.87 | 4.43 | 2006 |
| Saint Lucia | 4.13 | 3.85 | 4.41 | 2007 |
| Saint Lucia | 4.11 | 3.83 | 4.39 | 2008 |
| Saint Lucia | 4.09 | 3.81 | 4.36 | 2009 |
| Saint Lucia | 4.06 | 3.78 | 4.34 | 2010 |
| Saint Lucia | 4.03 | 3.75 | 4.31 | 2011 |
| Saint Lucia | 4 | 3.7 | 4.29 | 2012 |
| Saint Lucia | 3.96 | 3.65 | 4.27 | 2013 |
| Saint Lucia | 3.92 | 3.6 | 4.25 | 2014 |
| Saint Lucia | 3.88 | 3.53 | 4.23 | 2015 |
| Saint Lucia | 3.84 | 3.45 | 4.22 | 2016 |
| Saint Lucia | 3.79 | 3.36 | 4.21 | 2017 |
| Saint Lucia | 3.74 | 3.27 | 4.2 | 2018 |
| Saint Lucia | 3.68 | 3.16 | 4.2 | 2019 |
| Saint Lucia | 3.62 | 3.05 | 4.19 | 2020 |
| Saint Lucia | 3.56 | 2.93 | 4.19 | 2021 |
| Saint Lucia | 3.5 | 2.81 | 4.18 | 2022 |
| Saint Lucia | 3.43 | 2.69 | 4.18 | 2023 |
| Saint Lucia | 3.37 | 2.56 | 4.17 | 2024 |
| Saint Lucia | 3.3 | 2.43 | 4.16 | 2025 |
| Saint Lucia | 3.23 | 2.3 | 4.15 | 2026 |
| Saint Lucia | 3.16 | 2.17 | 4.14 | 2027 |
| Saint Lucia | 3.08 | 2.04 | 4.13 | 2028 |
| Saint Lucia | 3.01 | 1.91 | 4.11 | 2029 |
| Saint Lucia | 2.93 | 1.78 | 4.09 | 2030 |
| Sao Tome and Principe | 0.86 | 0.53 | 1.2 | 1990 |
| Sao Tome and Principe | 0.81 | 0.59 | 1.04 | 1991 |
| Sao Tome and Principe | 0.79 | 0.59 | 1 | 1992 |
| Sao Tome and Principe | 0.79 | 0.58 | 0.99 | 1993 |
| Sao Tome and Principe | 0.78 | 0.58 | 0.99 | 1994 |
| Sao Tome and Principe | 0.78 | 0.58 | 0.98 | 1995 |
| Sao Tome and Principe | 0.78 | 0.58 | 0.98 | 1996 |
| Sao Tome and Principe | 0.79 | 0.59 | 0.99 | 1997 |
| Sao Tome and Principe | 0.81 | 0.61 | 1.01 | 1998 |
| Sao Tome and Principe | 0.83 | 0.63 | 1.04 | 1999 |
| Sao Tome and Principe | 0.85 | 0.64 | 1.05 | 2000 |
| Sao Tome and Principe | 0.79 | 0.6 | 0.99 | 2001 |
| Sao Tome and Principe | 0.73 | 0.55 | 0.92 | 2002 |
| Sao Tome and Principe | 0.69 | 0.51 | 0.86 | 2003 |
| Sao Tome and Principe | 0.66 | 0.49 | 0.82 | 2004 |
| Sao Tome and Principe | 0.63 | 0.47 | 0.8 | 2005 |
| Sao Tome and Principe | 0.62 | 0.46 | 0.78 | 2006 |
| Sao Tome and Principe | 0.61 | 0.45 | 0.77 | 2007 |
| Sao Tome and Principe | 0.6 | 0.45 | 0.76 | 2008 |
| Sao Tome and Principe | 0.6 | 0.44 | 0.75 | 2009 |
| Sao Tome and Principe | 0.59 | 0.44 | 0.74 | 2010 |
| Sao Tome and Principe | 0.59 | 0.44 | 0.74 | 2011 |
| Sao Tome and Principe | 0.58 | 0.43 | 0.73 | 2012 |
| Sao Tome and Principe | 0.57 | 0.42 | 0.72 | 2013 |
| Sao Tome and Principe | 0.56 | 0.42 | 0.71 | 2014 |
| Sao Tome and Principe | 0.55 | 0.41 | 0.69 | 2015 |
| Sao Tome and Principe | 0.54 | 0.39 | 0.69 | 2016 |
| Sao Tome and Principe | 0.55 | 0.34 | 0.77 | 2017 |
| Sao Tome and Principe | 0.53 | 0.17 | 0.89 | 2018 |
| Sao Tome and Principe | 0.5 | -0.04 | 1.05 | 2019 |
| Sao Tome and Principe | 0.48 | -0.26 | 1.22 | 2020 |
| Sao Tome and Principe | 0.46 | -0.49 | 1.41 | 2021 |
| Sao Tome and Principe | 0.44 | -0.73 | 1.61 | 2022 |
| Sao Tome and Principe | 0.42 | -0.97 | 1.81 | 2023 |
| Sao Tome and Principe | 0.4 | -1.2 | 2.01 | 2024 |
| Sao Tome and Principe | 0.39 | -1.44 | 2.21 | 2025 |
| Sao Tome and Principe | 0.37 | -1.66 | 2.41 | 2026 |
| Sao Tome and Principe | 0.36 | -1.89 | 2.6 | 2027 |
| Sao Tome and Principe | 0.34 | -2.1 | 2.79 | 2028 |
| Sao Tome and Principe | 0.33 | -2.31 | 2.97 | 2029 |
| Sao Tome and Principe | 0.32 | -2.51 | 3.14 | 2030 |
| Saudi Arabia | 1.4 | 1.35 | 1.46 | 1990 |
| Saudi Arabia | 1.45 | 1.41 | 1.5 | 1991 |
| Saudi Arabia | 1.5 | 1.47 | 1.54 | 1992 |
| Saudi Arabia | 1.56 | 1.52 | 1.59 | 1993 |
| Saudi Arabia | 1.62 | 1.59 | 1.65 | 1994 |
| Saudi Arabia | 1.68 | 1.65 | 1.72 | 1995 |
| Saudi Arabia | 1.75 | 1.72 | 1.79 | 1996 |
| Saudi Arabia | 1.83 | 1.8 | 1.86 | 1997 |
| Saudi Arabia | 1.91 | 1.88 | 1.95 | 1998 |
| Saudi Arabia | 2 | 1.97 | 2.03 | 1999 |
| Saudi Arabia | 2.09 | 2.06 | 2.13 | 2000 |
| Saudi Arabia | 2.18 | 2.15 | 2.22 | 2001 |
| Saudi Arabia | 2.28 | 2.24 | 2.31 | 2002 |
| Saudi Arabia | 2.36 | 2.33 | 2.4 | 2003 |
| Saudi Arabia | 2.44 | 2.41 | 2.48 | 2004 |
| Saudi Arabia | 2.52 | 2.48 | 2.56 | 2005 |
| Saudi Arabia | 2.59 | 2.55 | 2.63 | 2006 |
| Saudi Arabia | 2.65 | 2.62 | 2.69 | 2007 |
| Saudi Arabia | 2.71 | 2.67 | 2.75 | 2008 |
| Saudi Arabia | 2.76 | 2.72 | 2.79 | 2009 |
| Saudi Arabia | 2.79 | 2.76 | 2.83 | 2010 |
| Saudi Arabia | 2.82 | 2.79 | 2.86 | 2011 |
| Saudi Arabia | 2.84 | 2.8 | 2.88 | 2012 |
| Saudi Arabia | 2.85 | 2.82 | 2.89 | 2013 |
| Saudi Arabia | 2.86 | 2.83 | 2.9 | 2014 |
| Saudi Arabia | 2.87 | 2.83 | 2.9 | 2015 |
| Saudi Arabia | 2.87 | 2.83 | 2.91 | 2016 |
| Saudi Arabia | 2.87 | 2.82 | 2.93 | 2017 |
| Saudi Arabia | 2.88 | 2.8 | 2.95 | 2018 |
| Saudi Arabia | 2.88 | 2.78 | 2.98 | 2019 |
| Saudi Arabia | 2.89 | 2.76 | 3.02 | 2020 |
| Saudi Arabia | 2.89 | 2.73 | 3.06 | 2021 |
| Saudi Arabia | 2.9 | 2.7 | 3.1 | 2022 |
| Saudi Arabia | 2.91 | 2.67 | 3.15 | 2023 |
| Saudi Arabia | 2.91 | 2.63 | 3.2 | 2024 |
| Saudi Arabia | 2.92 | 2.59 | 3.25 | 2025 |
| Saudi Arabia | 2.93 | 2.55 | 3.3 | 2026 |
| Saudi Arabia | 2.94 | 2.51 | 3.36 | 2027 |
| Saudi Arabia | 2.94 | 2.47 | 3.42 | 2028 |
| Saudi Arabia | 2.95 | 2.42 | 3.48 | 2029 |
| Saudi Arabia | 2.96 | 2.37 | 3.54 | 2030 |
| Senegal | 2.19 | 2.1 | 2.27 | 1990 |
| Senegal | 2.21 | 2.13 | 2.28 | 1991 |
| Senegal | 2.23 | 2.17 | 2.29 | 1992 |
| Senegal | 2.25 | 2.2 | 2.31 | 1993 |
| Senegal | 2.28 | 2.23 | 2.34 | 1994 |
| Senegal | 2.32 | 2.27 | 2.37 | 1995 |
| Senegal | 2.36 | 2.31 | 2.41 | 1996 |
| Senegal | 2.41 | 2.36 | 2.46 | 1997 |
| Senegal | 2.46 | 2.42 | 2.51 | 1998 |
| Senegal | 2.53 | 2.48 | 2.57 | 1999 |
| Senegal | 2.6 | 2.55 | 2.64 | 2000 |
| Senegal | 2.67 | 2.62 | 2.72 | 2001 |
| Senegal | 2.75 | 2.7 | 2.79 | 2002 |
| Senegal | 2.82 | 2.77 | 2.87 | 2003 |
| Senegal | 2.89 | 2.84 | 2.94 | 2004 |
| Senegal | 2.96 | 2.91 | 3 | 2005 |
| Senegal | 3.01 | 2.96 | 3.06 | 2006 |
| Senegal | 3.06 | 3.01 | 3.11 | 2007 |
| Senegal | 3.1 | 3.05 | 3.15 | 2008 |
| Senegal | 3.13 | 3.08 | 3.18 | 2009 |
| Senegal | 3.16 | 3.11 | 3.21 | 2010 |
| Senegal | 3.2 | 3.15 | 3.25 | 2011 |
| Senegal | 3.24 | 3.19 | 3.29 | 2012 |
| Senegal | 3.28 | 3.23 | 3.33 | 2013 |
| Senegal | 3.32 | 3.27 | 3.38 | 2014 |
| Senegal | 3.37 | 3.31 | 3.43 | 2015 |
| Senegal | 3.41 | 3.34 | 3.48 | 2016 |
| Senegal | 3.46 | 3.37 | 3.55 | 2017 |
| Senegal | 3.5 | 3.39 | 3.61 | 2018 |
| Senegal | 3.55 | 3.41 | 3.69 | 2019 |
| Senegal | 3.59 | 3.41 | 3.77 | 2020 |
| Senegal | 3.63 | 3.42 | 3.85 | 2021 |
| Senegal | 3.68 | 3.41 | 3.94 | 2022 |
| Senegal | 3.72 | 3.4 | 4.03 | 2023 |
| Senegal | 3.76 | 3.39 | 4.13 | 2024 |
| Senegal | 3.8 | 3.37 | 4.22 | 2025 |
| Senegal | 3.83 | 3.35 | 4.32 | 2026 |
| Senegal | 3.87 | 3.32 | 4.42 | 2027 |
| Senegal | 3.9 | 3.29 | 4.52 | 2028 |
| Senegal | 3.94 | 3.25 | 4.62 | 2029 |
| Senegal | 3.97 | 3.21 | 4.72 | 2030 |
| Serbia | 7.04 | 6.92 | 7.16 | 1990 |
| Serbia | 7.02 | 6.93 | 7.11 | 1991 |
| Serbia | 7.02 | 6.94 | 7.09 | 1992 |
| Serbia | 7.02 | 6.94 | 7.09 | 1993 |
| Serbia | 7.02 | 6.95 | 7.09 | 1994 |
| Serbia | 7.03 | 6.96 | 7.11 | 1995 |
| Serbia | 7.05 | 6.98 | 7.12 | 1996 |
| Serbia | 7.07 | 7 | 7.14 | 1997 |
| Serbia | 7.11 | 7.04 | 7.18 | 1998 |
| Serbia | 7.18 | 7.11 | 7.25 | 1999 |
| Serbia | 7.29 | 7.22 | 7.36 | 2000 |
| Serbia | 7.44 | 7.37 | 7.51 | 2001 |
| Serbia | 7.62 | 7.54 | 7.69 | 2002 |
| Serbia | 7.8 | 7.73 | 7.87 | 2003 |
| Serbia | 7.98 | 7.9 | 8.05 | 2004 |
| Serbia | 8.13 | 8.05 | 8.2 | 2005 |
| Serbia | 8.24 | 8.17 | 8.32 | 2006 |
| Serbia | 8.34 | 8.26 | 8.41 | 2007 |
| Serbia | 8.42 | 8.34 | 8.49 | 2008 |
| Serbia | 8.48 | 8.4 | 8.55 | 2009 |
| Serbia | 8.51 | 8.44 | 8.59 | 2010 |
| Serbia | 8.53 | 8.45 | 8.6 | 2011 |
| Serbia | 8.52 | 8.45 | 8.6 | 2012 |
| Serbia | 8.5 | 8.42 | 8.57 | 2013 |
| Serbia | 8.45 | 8.37 | 8.53 | 2014 |
| Serbia | 8.37 | 8.29 | 8.45 | 2015 |
| Serbia | 8.27 | 8.18 | 8.36 | 2016 |
| Serbia | 8.16 | 8.04 | 8.28 | 2017 |
| Serbia | 8.05 | 7.88 | 8.22 | 2018 |
| Serbia | 7.94 | 7.7 | 8.17 | 2019 |
| Serbia | 7.82 | 7.51 | 8.13 | 2020 |
| Serbia | 7.7 | 7.3 | 8.09 | 2021 |
| Serbia | 7.57 | 7.09 | 8.05 | 2022 |
| Serbia | 7.45 | 6.88 | 8.02 | 2023 |
| Serbia | 7.32 | 6.66 | 7.99 | 2024 |
| Serbia | 7.19 | 6.43 | 7.95 | 2025 |
| Serbia | 7.06 | 6.21 | 7.92 | 2026 |
| Serbia | 6.93 | 5.98 | 7.89 | 2027 |
| Serbia | 6.81 | 5.75 | 7.86 | 2028 |
| Serbia | 6.69 | 5.53 | 7.84 | 2029 |
| Serbia | 6.57 | 5.32 | 7.83 | 2030 |
| Sierra Leone | 2.23 | 2.1 | 2.35 | 1990 |
| Sierra Leone | 2.25 | 2.14 | 2.36 | 1991 |
| Sierra Leone | 2.28 | 2.18 | 2.38 | 1992 |
| Sierra Leone | 2.3 | 2.21 | 2.39 | 1993 |
| Sierra Leone | 2.33 | 2.25 | 2.41 | 1994 |
| Sierra Leone | 2.36 | 2.28 | 2.44 | 1995 |
| Sierra Leone | 2.39 | 2.31 | 2.47 | 1996 |
| Sierra Leone | 2.42 | 2.35 | 2.5 | 1997 |
| Sierra Leone | 2.46 | 2.38 | 2.53 | 1998 |
| Sierra Leone | 2.49 | 2.42 | 2.56 | 1999 |
| Sierra Leone | 2.53 | 2.46 | 2.6 | 2000 |
| Sierra Leone | 2.57 | 2.5 | 2.64 | 2001 |
| Sierra Leone | 2.61 | 2.54 | 2.68 | 2002 |
| Sierra Leone | 2.66 | 2.59 | 2.73 | 2003 |
| Sierra Leone | 2.71 | 2.63 | 2.78 | 2004 |
| Sierra Leone | 2.76 | 2.69 | 2.83 | 2005 |
| Sierra Leone | 2.81 | 2.74 | 2.89 | 2006 |
| Sierra Leone | 2.88 | 2.8 | 2.95 | 2007 |
| Sierra Leone | 2.94 | 2.87 | 3.01 | 2008 |
| Sierra Leone | 3.01 | 2.94 | 3.08 | 2009 |
| Sierra Leone | 3.08 | 3.01 | 3.16 | 2010 |
| Sierra Leone | 3.16 | 3.09 | 3.24 | 2011 |
| Sierra Leone | 3.24 | 3.16 | 3.32 | 2012 |
| Sierra Leone | 3.32 | 3.24 | 3.4 | 2013 |
| Sierra Leone | 3.4 | 3.32 | 3.49 | 2014 |
| Sierra Leone | 3.48 | 3.39 | 3.58 | 2015 |
| Sierra Leone | 3.57 | 3.46 | 3.67 | 2016 |
| Sierra Leone | 3.65 | 3.52 | 3.78 | 2017 |
| Sierra Leone | 3.74 | 3.59 | 3.89 | 2018 |
| Sierra Leone | 3.83 | 3.64 | 4.02 | 2019 |
| Sierra Leone | 3.92 | 3.7 | 4.15 | 2020 |
| Sierra Leone | 4.02 | 3.75 | 4.29 | 2021 |
| Sierra Leone | 4.12 | 3.8 | 4.44 | 2022 |
| Sierra Leone | 4.22 | 3.84 | 4.6 | 2023 |
| Sierra Leone | 4.33 | 3.88 | 4.77 | 2024 |
| Sierra Leone | 4.43 | 3.92 | 4.95 | 2025 |
| Sierra Leone | 4.54 | 3.96 | 5.13 | 2026 |
| Sierra Leone | 4.66 | 3.99 | 5.33 | 2027 |
| Sierra Leone | 4.77 | 4.02 | 5.53 | 2028 |
| Sierra Leone | 4.89 | 4.04 | 5.74 | 2029 |
| Sierra Leone | 5.01 | 4.06 | 5.96 | 2030 |
| Singapore | 2.67 | 2.55 | 2.78 | 1990 |
| Singapore | 2.67 | 2.57 | 2.77 | 1991 |
| Singapore | 2.68 | 2.59 | 2.76 | 1992 |
| Singapore | 2.68 | 2.61 | 2.76 | 1993 |
| Singapore | 2.69 | 2.62 | 2.76 | 1994 |
| Singapore | 2.7 | 2.64 | 2.76 | 1995 |
| Singapore | 2.71 | 2.65 | 2.77 | 1996 |
| Singapore | 2.72 | 2.66 | 2.78 | 1997 |
| Singapore | 2.74 | 2.68 | 2.8 | 1998 |
| Singapore | 2.76 | 2.7 | 2.81 | 1999 |
| Singapore | 2.77 | 2.72 | 2.83 | 2000 |
| Singapore | 2.79 | 2.74 | 2.85 | 2001 |
| Singapore | 2.81 | 2.76 | 2.87 | 2002 |
| Singapore | 2.83 | 2.78 | 2.89 | 2003 |
| Singapore | 2.85 | 2.8 | 2.91 | 2004 |
| Singapore | 2.88 | 2.82 | 2.93 | 2005 |
| Singapore | 2.9 | 2.84 | 2.95 | 2006 |
| Singapore | 2.92 | 2.86 | 2.97 | 2007 |
| Singapore | 2.93 | 2.88 | 2.98 | 2008 |
| Singapore | 2.94 | 2.89 | 2.99 | 2009 |
| Singapore | 2.95 | 2.9 | 3 | 2010 |
| Singapore | 2.95 | 2.9 | 3 | 2011 |
| Singapore | 2.95 | 2.9 | 2.99 | 2012 |
| Singapore | 2.94 | 2.89 | 2.99 | 2013 |
| Singapore | 2.92 | 2.87 | 2.97 | 2014 |
| Singapore | 2.91 | 2.85 | 2.96 | 2015 |
| Singapore | 2.89 | 2.82 | 2.95 | 2016 |
| Singapore | 2.87 | 2.79 | 2.95 | 2017 |
| Singapore | 2.85 | 2.76 | 2.95 | 2018 |
| Singapore | 2.84 | 2.72 | 2.96 | 2019 |
| Singapore | 2.82 | 2.67 | 2.97 | 2020 |
| Singapore | 2.81 | 2.63 | 2.99 | 2021 |
| Singapore | 2.79 | 2.58 | 3 | 2022 |
| Singapore | 2.78 | 2.53 | 3.02 | 2023 |
| Singapore | 2.76 | 2.49 | 3.04 | 2024 |
| Singapore | 2.75 | 2.44 | 3.07 | 2025 |
| Singapore | 2.74 | 2.38 | 3.09 | 2026 |
| Singapore | 2.73 | 2.33 | 3.12 | 2027 |
| Singapore | 2.72 | 2.28 | 3.15 | 2028 |
| Singapore | 2.71 | 2.23 | 3.18 | 2029 |
| Singapore | 2.69 | 2.17 | 3.22 | 2030 |
| Slovakia | 5.61 | 5.47 | 5.76 | 1990 |
| Slovakia | 6.26 | 6.14 | 6.38 | 1991 |
| Slovakia | 6.98 | 6.87 | 7.09 | 1992 |
| Slovakia | 7.8 | 7.69 | 7.91 | 1993 |
| Slovakia | 8.7 | 8.58 | 8.82 | 1994 |
| Slovakia | 9.66 | 9.53 | 9.79 | 1995 |
| Slovakia | 10.62 | 10.49 | 10.76 | 1996 |
| Slovakia | 11.54 | 11.4 | 11.68 | 1997 |
| Slovakia | 12.35 | 12.2 | 12.5 | 1998 |
| Slovakia | 13.01 | 12.86 | 13.16 | 1999 |
| Slovakia | 13.51 | 13.36 | 13.67 | 2000 |
| Slovakia | 13.91 | 13.75 | 14.06 | 2001 |
| Slovakia | 14.23 | 14.07 | 14.39 | 2002 |
| Slovakia | 14.54 | 14.38 | 14.7 | 2003 |
| Slovakia | 14.86 | 14.7 | 15.01 | 2004 |
| Slovakia | 15.2 | 15.04 | 15.36 | 2005 |
| Slovakia | 15.54 | 15.38 | 15.7 | 2006 |
| Slovakia | 15.87 | 15.71 | 16.03 | 2007 |
| Slovakia | 16.16 | 16 | 16.33 | 2008 |
| Slovakia | 16.38 | 16.22 | 16.55 | 2009 |
| Slovakia | 16.52 | 16.35 | 16.68 | 2010 |
| Slovakia | 16.57 | 16.4 | 16.73 | 2011 |
| Slovakia | 16.52 | 16.36 | 16.68 | 2012 |
| Slovakia | 16.34 | 16.18 | 16.5 | 2013 |
| Slovakia | 16.02 | 15.86 | 16.18 | 2014 |
| Slovakia | 15.57 | 15.42 | 15.73 | 2015 |
| Slovakia | 15.01 | 14.84 | 15.17 | 2016 |
| Slovakia | 14.42 | 14.19 | 14.64 | 2017 |
| Slovakia | 13.85 | 13.49 | 14.21 | 2018 |
| Slovakia | 13.29 | 12.76 | 13.83 | 2019 |
| Slovakia | 12.76 | 12.03 | 13.5 | 2020 |
| Slovakia | 12.25 | 11.31 | 13.19 | 2021 |
| Slovakia | 11.75 | 10.6 | 12.9 | 2022 |
| Slovakia | 11.27 | 9.91 | 12.63 | 2023 |
| Slovakia | 10.81 | 9.24 | 12.38 | 2024 |
| Slovakia | 10.37 | 8.59 | 12.15 | 2025 |
| Slovakia | 9.94 | 7.96 | 11.92 | 2026 |
| Slovakia | 9.53 | 7.36 | 11.7 | 2027 |
| Slovakia | 9.14 | 6.78 | 11.49 | 2028 |
| Slovakia | 8.76 | 6.23 | 11.29 | 2029 |
| Slovakia | 8.4 | 5.7 | 11.1 | 2030 |
| Slovenia | 5.63 | 5.45 | 5.82 | 1990 |
| Slovenia | 5.82 | 5.66 | 5.98 | 1991 |
| Slovenia | 6.01 | 5.87 | 6.15 | 1992 |
| Slovenia | 6.2 | 6.07 | 6.32 | 1993 |
| Slovenia | 6.38 | 6.26 | 6.5 | 1994 |
| Slovenia | 6.57 | 6.45 | 6.69 | 1995 |
| Slovenia | 6.77 | 6.65 | 6.88 | 1996 |
| Slovenia | 6.97 | 6.85 | 7.09 | 1997 |
| Slovenia | 7.18 | 7.06 | 7.3 | 1998 |
| Slovenia | 7.4 | 7.27 | 7.52 | 1999 |
| Slovenia | 7.62 | 7.49 | 7.74 | 2000 |
| Slovenia | 7.85 | 7.72 | 7.97 | 2001 |
| Slovenia | 8.07 | 7.95 | 8.2 | 2002 |
| Slovenia | 8.29 | 8.17 | 8.42 | 2003 |
| Slovenia | 8.49 | 8.36 | 8.62 | 2004 |
| Slovenia | 8.67 | 8.54 | 8.8 | 2005 |
| Slovenia | 8.81 | 8.67 | 8.94 | 2006 |
| Slovenia | 8.91 | 8.77 | 9.04 | 2007 |
| Slovenia | 8.96 | 8.83 | 9.09 | 2008 |
| Slovenia | 8.98 | 8.84 | 9.11 | 2009 |
| Slovenia | 8.95 | 8.82 | 9.08 | 2010 |
| Slovenia | 8.89 | 8.76 | 9.02 | 2011 |
| Slovenia | 8.81 | 8.68 | 8.94 | 2012 |
| Slovenia | 8.7 | 8.58 | 8.83 | 2013 |
| Slovenia | 8.58 | 8.45 | 8.71 | 2014 |
| Slovenia | 8.44 | 8.31 | 8.58 | 2015 |
| Slovenia | 8.3 | 8.14 | 8.46 | 2016 |
| Slovenia | 8.15 | 7.95 | 8.35 | 2017 |
| Slovenia | 8 | 7.74 | 8.26 | 2018 |
| Slovenia | 7.85 | 7.52 | 8.18 | 2019 |
| Slovenia | 7.7 | 7.3 | 8.11 | 2020 |
| Slovenia | 7.55 | 7.06 | 8.04 | 2021 |
| Slovenia | 7.4 | 6.82 | 7.97 | 2022 |
| Slovenia | 7.24 | 6.58 | 7.91 | 2023 |
| Slovenia | 7.09 | 6.33 | 7.85 | 2024 |
| Slovenia | 6.94 | 6.09 | 7.79 | 2025 |
| Slovenia | 6.79 | 5.84 | 7.73 | 2026 |
| Slovenia | 6.63 | 5.6 | 7.67 | 2027 |
| Slovenia | 6.48 | 5.36 | 7.61 | 2028 |
| Slovenia | 6.33 | 5.11 | 7.55 | 2029 |
| Slovenia | 6.18 | 4.87 | 7.49 | 2030 |
| Solomon Islands | 1.7 | 1.43 | 1.97 | 1990 |
| Solomon Islands | 1.8 | 1.54 | 2.06 | 1991 |
| Solomon Islands | 1.89 | 1.65 | 2.14 | 1992 |
| Solomon Islands | 1.99 | 1.75 | 2.23 | 1993 |
| Solomon Islands | 2.09 | 1.86 | 2.31 | 1994 |
| Solomon Islands | 2.18 | 1.96 | 2.4 | 1995 |
| Solomon Islands | 2.28 | 2.07 | 2.49 | 1996 |
| Solomon Islands | 2.37 | 2.16 | 2.57 | 1997 |
| Solomon Islands | 2.46 | 2.26 | 2.66 | 1998 |
| Solomon Islands | 2.55 | 2.35 | 2.74 | 1999 |
| Solomon Islands | 2.63 | 2.44 | 2.82 | 2000 |
| Solomon Islands | 2.71 | 2.52 | 2.9 | 2001 |
| Solomon Islands | 2.78 | 2.59 | 2.96 | 2002 |
| Solomon Islands | 2.84 | 2.66 | 3.03 | 2003 |
| Solomon Islands | 2.91 | 2.72 | 3.09 | 2004 |
| Solomon Islands | 2.96 | 2.78 | 3.14 | 2005 |
| Solomon Islands | 3.01 | 2.83 | 3.19 | 2006 |
| Solomon Islands | 3.05 | 2.87 | 3.24 | 2007 |
| Solomon Islands | 3.09 | 2.91 | 3.28 | 2008 |
| Solomon Islands | 3.12 | 2.94 | 3.31 | 2009 |
| Solomon Islands | 3.15 | 2.96 | 3.34 | 2010 |
| Solomon Islands | 3.17 | 2.98 | 3.37 | 2011 |
| Solomon Islands | 3.19 | 2.98 | 3.39 | 2012 |
| Solomon Islands | 3.2 | 2.98 | 3.41 | 2013 |
| Solomon Islands | 3.2 | 2.98 | 3.43 | 2014 |
| Solomon Islands | 3.21 | 2.96 | 3.45 | 2015 |
| Solomon Islands | 3.2 | 2.93 | 3.48 | 2016 |
| Solomon Islands | 3.2 | 2.9 | 3.5 | 2017 |
| Solomon Islands | 3.19 | 2.85 | 3.53 | 2018 |
| Solomon Islands | 3.19 | 2.8 | 3.57 | 2019 |
| Solomon Islands | 3.18 | 2.75 | 3.6 | 2020 |
| Solomon Islands | 3.17 | 2.69 | 3.65 | 2021 |
| Solomon Islands | 3.16 | 2.63 | 3.69 | 2022 |
| Solomon Islands | 3.15 | 2.57 | 3.74 | 2023 |
| Solomon Islands | 3.15 | 2.5 | 3.79 | 2024 |
| Solomon Islands | 3.14 | 2.43 | 3.85 | 2025 |
| Solomon Islands | 3.13 | 2.36 | 3.91 | 2026 |
| Solomon Islands | 3.13 | 2.28 | 3.98 | 2027 |
| Solomon Islands | 3.13 | 2.21 | 4.05 | 2028 |
| Solomon Islands | 3.12 | 2.13 | 4.12 | 2029 |
| Solomon Islands | 3.12 | 2.05 | 4.2 | 2030 |
| Somalia | 1.99 | 1.91 | 2.07 | 1990 |
| Somalia | 2.08 | 2.01 | 2.15 | 1991 |
| Somalia | 2.17 | 2.11 | 2.23 | 1992 |
| Somalia | 2.26 | 2.21 | 2.32 | 1993 |
| Somalia | 2.35 | 2.29 | 2.4 | 1994 |
| Somalia | 2.41 | 2.36 | 2.47 | 1995 |
| Somalia | 2.46 | 2.41 | 2.52 | 1996 |
| Somalia | 2.49 | 2.44 | 2.55 | 1997 |
| Somalia | 2.5 | 2.45 | 2.55 | 1998 |
| Somalia | 2.5 | 2.45 | 2.55 | 1999 |
| Somalia | 2.49 | 2.44 | 2.54 | 2000 |
| Somalia | 2.48 | 2.43 | 2.53 | 2001 |
| Somalia | 2.47 | 2.42 | 2.52 | 2002 |
| Somalia | 2.48 | 2.43 | 2.53 | 2003 |
| Somalia | 2.5 | 2.45 | 2.55 | 2004 |
| Somalia | 2.53 | 2.48 | 2.58 | 2005 |
| Somalia | 2.58 | 2.53 | 2.63 | 2006 |
| Somalia | 2.64 | 2.6 | 2.69 | 2007 |
| Somalia | 2.71 | 2.66 | 2.76 | 2008 |
| Somalia | 2.77 | 2.71 | 2.82 | 2009 |
| Somalia | 2.81 | 2.76 | 2.86 | 2010 |
| Somalia | 2.83 | 2.78 | 2.89 | 2011 |
| Somalia | 2.85 | 2.8 | 2.9 | 2012 |
| Somalia | 2.86 | 2.81 | 2.92 | 2013 |
| Somalia | 2.87 | 2.81 | 2.92 | 2014 |
| Somalia | 2.87 | 2.81 | 2.92 | 2015 |
| Somalia | 2.86 | 2.8 | 2.93 | 2016 |
| Somalia | 2.86 | 2.77 | 2.94 | 2017 |
| Somalia | 2.85 | 2.74 | 2.96 | 2018 |
| Somalia | 2.84 | 2.69 | 2.99 | 2019 |
| Somalia | 2.84 | 2.64 | 3.03 | 2020 |
| Somalia | 2.83 | 2.59 | 3.07 | 2021 |
| Somalia | 2.83 | 2.53 | 3.12 | 2022 |
| Somalia | 2.82 | 2.47 | 3.18 | 2023 |
| Somalia | 2.82 | 2.4 | 3.23 | 2024 |
| Somalia | 2.82 | 2.34 | 3.3 | 2025 |
| Somalia | 2.81 | 2.27 | 3.36 | 2026 |
| Somalia | 2.81 | 2.19 | 3.44 | 2027 |
| Somalia | 2.82 | 2.12 | 3.51 | 2028 |
| Somalia | 2.82 | 2.04 | 3.59 | 2029 |
| Somalia | 2.82 | 1.96 | 3.68 | 2030 |
| South Africa | 3.1 | 3.04 | 3.16 | 1990 |
| South Africa | 3.13 | 3.09 | 3.17 | 1991 |
| South Africa | 3.16 | 3.12 | 3.2 | 1992 |
| South Africa | 3.21 | 3.17 | 3.25 | 1993 |
| South Africa | 3.29 | 3.25 | 3.32 | 1994 |
| South Africa | 3.4 | 3.36 | 3.44 | 1995 |
| South Africa | 3.57 | 3.53 | 3.61 | 1996 |
| South Africa | 3.78 | 3.74 | 3.82 | 1997 |
| South Africa | 4 | 3.96 | 4.04 | 1998 |
| South Africa | 4.2 | 4.16 | 4.25 | 1999 |
| South Africa | 4.38 | 4.33 | 4.42 | 2000 |
| South Africa | 4.48 | 4.44 | 4.52 | 2001 |
| South Africa | 4.52 | 4.48 | 4.56 | 2002 |
| South Africa | 4.52 | 4.47 | 4.56 | 2003 |
| South Africa | 4.48 | 4.44 | 4.52 | 2004 |
| South Africa | 4.42 | 4.38 | 4.46 | 2005 |
| South Africa | 4.32 | 4.28 | 4.36 | 2006 |
| South Africa | 4.21 | 4.17 | 4.25 | 2007 |
| South Africa | 4.08 | 4.04 | 4.12 | 2008 |
| South Africa | 3.95 | 3.91 | 3.99 | 2009 |
| South Africa | 3.82 | 3.78 | 3.86 | 2010 |
| South Africa | 3.71 | 3.67 | 3.74 | 2011 |
| South Africa | 3.62 | 3.59 | 3.66 | 2012 |
| South Africa | 3.58 | 3.54 | 3.61 | 2013 |
| South Africa | 3.57 | 3.53 | 3.6 | 2014 |
| South Africa | 3.59 | 3.56 | 3.63 | 2015 |
| South Africa | 3.62 | 3.59 | 3.66 | 2016 |
| South Africa | 3.64 | 3.59 | 3.69 | 2017 |
| South Africa | 3.66 | 3.57 | 3.75 | 2018 |
| South Africa | 3.68 | 3.54 | 3.83 | 2019 |
| South Africa | 3.7 | 3.48 | 3.91 | 2020 |
| South Africa | 3.71 | 3.42 | 4 | 2021 |
| South Africa | 3.72 | 3.34 | 4.09 | 2022 |
| South Africa | 3.72 | 3.26 | 4.19 | 2023 |
| South Africa | 3.73 | 3.16 | 4.29 | 2024 |
| South Africa | 3.73 | 3.06 | 4.39 | 2025 |
| South Africa | 3.72 | 2.95 | 4.5 | 2026 |
| South Africa | 3.72 | 2.83 | 4.61 | 2027 |
| South Africa | 3.72 | 2.71 | 4.72 | 2028 |
| South Africa | 3.71 | 2.58 | 4.83 | 2029 |
| South Africa | 3.7 | 2.45 | 4.95 | 2030 |
| South Korea | 2.15 | 2.11 | 2.19 | 1990 |
| South Korea | 2.27 | 2.24 | 2.3 | 1991 |
| South Korea | 2.4 | 2.37 | 2.42 | 1992 |
| South Korea | 2.54 | 2.51 | 2.57 | 1993 |
| South Korea | 2.7 | 2.68 | 2.73 | 1994 |
| South Korea | 2.88 | 2.85 | 2.91 | 1995 |
| South Korea | 3.06 | 3.04 | 3.09 | 1996 |
| South Korea | 3.25 | 3.22 | 3.28 | 1997 |
| South Korea | 3.42 | 3.39 | 3.45 | 1998 |
| South Korea | 3.59 | 3.56 | 3.62 | 1999 |
| South Korea | 3.73 | 3.7 | 3.76 | 2000 |
| South Korea | 3.87 | 3.84 | 3.9 | 2001 |
| South Korea | 3.99 | 3.96 | 4.02 | 2002 |
| South Korea | 4.1 | 4.07 | 4.13 | 2003 |
| South Korea | 4.21 | 4.18 | 4.24 | 2004 |
| South Korea | 4.31 | 4.28 | 4.34 | 2005 |
| South Korea | 4.41 | 4.38 | 4.44 | 2006 |
| South Korea | 4.51 | 4.48 | 4.54 | 2007 |
| South Korea | 4.62 | 4.59 | 4.65 | 2008 |
| South Korea | 4.72 | 4.69 | 4.75 | 2009 |
| South Korea | 4.81 | 4.78 | 4.85 | 2010 |
| South Korea | 4.87 | 4.84 | 4.91 | 2011 |
| South Korea | 4.88 | 4.85 | 4.92 | 2012 |
| South Korea | 4.84 | 4.8 | 4.87 | 2013 |
| South Korea | 4.74 | 4.71 | 4.77 | 2014 |
| South Korea | 4.63 | 4.6 | 4.66 | 2015 |
| South Korea | 4.51 | 4.48 | 4.54 | 2016 |
| South Korea | 4.41 | 4.37 | 4.45 | 2017 |
| South Korea | 4.31 | 4.24 | 4.38 | 2018 |
| South Korea | 4.21 | 4.1 | 4.32 | 2019 |
| South Korea | 4.11 | 3.95 | 4.27 | 2020 |
| South Korea | 4.01 | 3.8 | 4.22 | 2021 |
| South Korea | 3.91 | 3.65 | 4.17 | 2022 |
| South Korea | 3.82 | 3.5 | 4.13 | 2023 |
| South Korea | 3.72 | 3.35 | 4.09 | 2024 |
| South Korea | 3.62 | 3.2 | 4.04 | 2025 |
| South Korea | 3.53 | 3.05 | 4 | 2026 |
| South Korea | 3.43 | 2.9 | 3.97 | 2027 |
| South Korea | 3.34 | 2.75 | 3.93 | 2028 |
| South Korea | 3.25 | 2.6 | 3.89 | 2029 |
| South Korea | 3.16 | 2.46 | 3.86 | 2030 |
| South Sudan | 2.54 | 2.43 | 2.65 | 1990 |
| South Sudan | 2.55 | 2.45 | 2.64 | 1991 |
| South Sudan | 2.55 | 2.47 | 2.63 | 1992 |
| South Sudan | 2.56 | 2.49 | 2.63 | 1993 |
| South Sudan | 2.55 | 2.48 | 2.62 | 1994 |
| South Sudan | 2.54 | 2.47 | 2.6 | 1995 |
| South Sudan | 2.51 | 2.44 | 2.57 | 1996 |
| South Sudan | 2.46 | 2.4 | 2.52 | 1997 |
| South Sudan | 2.41 | 2.36 | 2.47 | 1998 |
| South Sudan | 2.36 | 2.3 | 2.41 | 1999 |
| South Sudan | 2.31 | 2.26 | 2.36 | 2000 |
| South Sudan | 2.26 | 2.21 | 2.32 | 2001 |
| South Sudan | 2.23 | 2.18 | 2.28 | 2002 |
| South Sudan | 2.21 | 2.16 | 2.26 | 2003 |
| South Sudan | 2.2 | 2.15 | 2.25 | 2004 |
| South Sudan | 2.2 | 2.16 | 2.25 | 2005 |
| South Sudan | 2.21 | 2.16 | 2.25 | 2006 |
| South Sudan | 2.21 | 2.17 | 2.26 | 2007 |
| South Sudan | 2.22 | 2.17 | 2.26 | 2008 |
| South Sudan | 2.22 | 2.18 | 2.26 | 2009 |
| South Sudan | 2.22 | 2.17 | 2.26 | 2010 |
| South Sudan | 2.21 | 2.17 | 2.25 | 2011 |
| South Sudan | 2.21 | 2.16 | 2.25 | 2012 |
| South Sudan | 2.2 | 2.16 | 2.24 | 2013 |
| South Sudan | 2.19 | 2.15 | 2.23 | 2014 |
| South Sudan | 2.18 | 2.13 | 2.23 | 2015 |
| South Sudan | 2.17 | 2.11 | 2.22 | 2016 |
| South Sudan | 2.15 | 2.09 | 2.22 | 2017 |
| South Sudan | 2.14 | 2.05 | 2.22 | 2018 |
| South Sudan | 2.12 | 2.01 | 2.23 | 2019 |
| South Sudan | 2.11 | 1.97 | 2.25 | 2020 |
| South Sudan | 2.1 | 1.93 | 2.26 | 2021 |
| South Sudan | 2.08 | 1.88 | 2.29 | 2022 |
| South Sudan | 2.07 | 1.83 | 2.31 | 2023 |
| South Sudan | 2.06 | 1.78 | 2.34 | 2024 |
| South Sudan | 2.05 | 1.73 | 2.37 | 2025 |
| South Sudan | 2.03 | 1.67 | 2.4 | 2026 |
| South Sudan | 2.02 | 1.62 | 2.43 | 2027 |
| South Sudan | 2.01 | 1.56 | 2.46 | 2028 |
| South Sudan | 2 | 1.5 | 2.49 | 2029 |
| South Sudan | 1.99 | 1.44 | 2.53 | 2030 |
| Spain | 8.9 | 8.83 | 8.97 | 1990 |
| Spain | 9.25 | 9.2 | 9.3 | 1991 |
| Spain | 9.59 | 9.55 | 9.64 | 1992 |
| Spain | 9.94 | 9.9 | 9.99 | 1993 |
| Spain | 10.29 | 10.24 | 10.34 | 1994 |
| Spain | 10.62 | 10.57 | 10.66 | 1995 |
| Spain | 10.91 | 10.86 | 10.96 | 1996 |
| Spain | 11.15 | 11.1 | 11.2 | 1997 |
| Spain | 11.36 | 11.3 | 11.41 | 1998 |
| Spain | 11.51 | 11.46 | 11.56 | 1999 |
| Spain | 11.59 | 11.54 | 11.64 | 2000 |
| Spain | 11.64 | 11.59 | 11.69 | 2001 |
| Spain | 11.68 | 11.63 | 11.72 | 2002 |
| Spain | 11.71 | 11.66 | 11.76 | 2003 |
| Spain | 11.72 | 11.67 | 11.77 | 2004 |
| Spain | 11.72 | 11.67 | 11.77 | 2005 |
| Spain | 11.71 | 11.66 | 11.75 | 2006 |
| Spain | 11.71 | 11.66 | 11.76 | 2007 |
| Spain | 11.73 | 11.68 | 11.78 | 2008 |
| Spain | 11.75 | 11.71 | 11.8 | 2009 |
| Spain | 11.78 | 11.74 | 11.83 | 2010 |
| Spain | 11.82 | 11.78 | 11.87 | 2011 |
| Spain | 11.86 | 11.81 | 11.91 | 2012 |
| Spain | 11.89 | 11.84 | 11.94 | 2013 |
| Spain | 11.89 | 11.84 | 11.94 | 2014 |
| Spain | 11.83 | 11.78 | 11.87 | 2015 |
| Spain | 11.67 | 11.62 | 11.71 | 2016 |
| Spain | 11.48 | 11.41 | 11.55 | 2017 |
| Spain | 11.3 | 11.18 | 11.42 | 2018 |
| Spain | 11.11 | 10.93 | 11.3 | 2019 |
| Spain | 10.92 | 10.65 | 11.19 | 2020 |
| Spain | 10.72 | 10.37 | 11.07 | 2021 |
| Spain | 10.53 | 10.08 | 10.97 | 2022 |
| Spain | 10.34 | 9.8 | 10.89 | 2023 |
| Spain | 10.17 | 9.53 | 10.81 | 2024 |
| Spain | 9.99 | 9.25 | 10.74 | 2025 |
| Spain | 9.81 | 8.96 | 10.66 | 2026 |
| Spain | 9.63 | 8.67 | 10.58 | 2027 |
| Spain | 9.45 | 8.39 | 10.51 | 2028 |
| Spain | 9.26 | 8.09 | 10.43 | 2029 |
| Spain | 9.07 | 7.8 | 10.34 | 2030 |
| Sri Lanka | 7.94 | 7.79 | 8.09 | 1990 |
| Sri Lanka | 8.45 | 8.33 | 8.56 | 1991 |
| Sri Lanka | 9.16 | 9.04 | 9.28 | 1992 |
| Sri Lanka | 10.12 | 9.99 | 10.24 | 1993 |
| Sri Lanka | 11.36 | 11.23 | 11.49 | 1994 |
| Sri Lanka | 12.81 | 12.66 | 12.95 | 1995 |
| Sri Lanka | 14.28 | 14.13 | 14.43 | 1996 |
| Sri Lanka | 15.5 | 15.34 | 15.66 | 1997 |
| Sri Lanka | 15.74 | 15.58 | 15.9 | 1998 |
| Sri Lanka | 15.39 | 15.23 | 15.54 | 1999 |
| Sri Lanka | 14.41 | 14.27 | 14.56 | 2000 |
| Sri Lanka | 12.94 | 12.81 | 13.08 | 2001 |
| Sri Lanka | 11.47 | 11.34 | 11.59 | 2002 |
| Sri Lanka | 10.19 | 10.08 | 10.31 | 2003 |
| Sri Lanka | 9.24 | 9.13 | 9.34 | 2004 |
| Sri Lanka | 8.35 | 8.25 | 8.45 | 2005 |
| Sri Lanka | 7.67 | 7.58 | 7.77 | 2006 |
| Sri Lanka | 7.2 | 7.11 | 7.29 | 2007 |
| Sri Lanka | 6.68 | 6.6 | 6.77 | 2008 |
| Sri Lanka | 6.21 | 6.13 | 6.29 | 2009 |
| Sri Lanka | 6.03 | 5.95 | 6.11 | 2010 |
| Sri Lanka | 6.19 | 6.11 | 6.27 | 2011 |
| Sri Lanka | 6.45 | 6.37 | 6.53 | 2012 |
| Sri Lanka | 6.66 | 6.58 | 6.74 | 2013 |
| Sri Lanka | 6.72 | 6.64 | 6.8 | 2014 |
| Sri Lanka | 6.69 | 6.61 | 6.77 | 2015 |
| Sri Lanka | 6.6 | 6.52 | 6.68 | 2016 |
| Sri Lanka | 6.48 | 6.38 | 6.58 | 2017 |
| Sri Lanka | 6.36 | 6.12 | 6.61 | 2018 |
| Sri Lanka | 6.25 | 5.8 | 6.7 | 2019 |
| Sri Lanka | 6.13 | 5.44 | 6.82 | 2020 |
| Sri Lanka | 6.03 | 5.07 | 6.98 | 2021 |
| Sri Lanka | 5.92 | 4.67 | 7.17 | 2022 |
| Sri Lanka | 5.82 | 4.27 | 7.37 | 2023 |
| Sri Lanka | 5.72 | 3.85 | 7.59 | 2024 |
| Sri Lanka | 5.63 | 3.43 | 7.83 | 2025 |
| Sri Lanka | 5.54 | 2.99 | 8.08 | 2026 |
| Sri Lanka | 5.45 | 2.56 | 8.34 | 2027 |
| Sri Lanka | 5.36 | 2.12 | 8.61 | 2028 |
| Sri Lanka | 5.28 | 1.67 | 8.89 | 2029 |
| Sri Lanka | 5.2 | 1.22 | 9.19 | 2030 |
| Sudan | 2.15 | 2.09 | 2.2 | 1990 |
| Sudan | 2.11 | 2.07 | 2.16 | 1991 |
| Sudan | 2.07 | 2.04 | 2.11 | 1992 |
| Sudan | 2.04 | 2 | 2.07 | 1993 |
| Sudan | 2 | 1.97 | 2.03 | 1994 |
| Sudan | 1.97 | 1.94 | 2 | 1995 |
| Sudan | 1.94 | 1.91 | 1.97 | 1996 |
| Sudan | 1.92 | 1.89 | 1.95 | 1997 |
| Sudan | 1.9 | 1.88 | 1.93 | 1998 |
| Sudan | 1.9 | 1.87 | 1.93 | 1999 |
| Sudan | 1.9 | 1.88 | 1.93 | 2000 |
| Sudan | 1.91 | 1.89 | 1.94 | 2001 |
| Sudan | 1.93 | 1.91 | 1.96 | 2002 |
| Sudan | 1.96 | 1.93 | 1.98 | 2003 |
| Sudan | 1.99 | 1.97 | 2.02 | 2004 |
| Sudan | 2.03 | 2.01 | 2.06 | 2005 |
| Sudan | 2.08 | 2.05 | 2.1 | 2006 |
| Sudan | 2.12 | 2.09 | 2.14 | 2007 |
| Sudan | 2.16 | 2.13 | 2.18 | 2008 |
| Sudan | 2.19 | 2.17 | 2.22 | 2009 |
| Sudan | 2.23 | 2.2 | 2.25 | 2010 |
| Sudan | 2.26 | 2.23 | 2.29 | 2011 |
| Sudan | 2.29 | 2.27 | 2.32 | 2012 |
| Sudan | 2.32 | 2.3 | 2.35 | 2013 |
| Sudan | 2.35 | 2.33 | 2.38 | 2014 |
| Sudan | 2.38 | 2.35 | 2.41 | 2015 |
| Sudan | 2.4 | 2.37 | 2.44 | 2016 |
| Sudan | 2.43 | 2.38 | 2.47 | 2017 |
| Sudan | 2.45 | 2.39 | 2.51 | 2018 |
| Sudan | 2.47 | 2.39 | 2.56 | 2019 |
| Sudan | 2.5 | 2.38 | 2.61 | 2020 |
| Sudan | 2.52 | 2.38 | 2.66 | 2021 |
| Sudan | 2.55 | 2.37 | 2.72 | 2022 |
| Sudan | 2.57 | 2.36 | 2.78 | 2023 |
| Sudan | 2.6 | 2.35 | 2.85 | 2024 |
| Sudan | 2.63 | 2.33 | 2.92 | 2025 |
| Sudan | 2.65 | 2.31 | 2.99 | 2026 |
| Sudan | 2.68 | 2.29 | 3.07 | 2027 |
| Sudan | 2.71 | 2.27 | 3.15 | 2028 |
| Sudan | 2.74 | 2.24 | 3.23 | 2029 |
| Sudan | 2.77 | 2.22 | 3.32 | 2030 |
| Suriname | 5.31 | 4.88 | 5.75 | 1990 |
| Suriname | 5.21 | 4.84 | 5.59 | 1991 |
| Suriname | 5.11 | 4.79 | 5.44 | 1992 |
| Suriname | 5.02 | 4.73 | 5.3 | 1993 |
| Suriname | 4.93 | 4.67 | 5.19 | 1994 |
| Suriname | 4.85 | 4.62 | 5.09 | 1995 |
| Suriname | 4.79 | 4.56 | 5.02 | 1996 |
| Suriname | 4.74 | 4.52 | 4.96 | 1997 |
| Suriname | 4.7 | 4.49 | 4.92 | 1998 |
| Suriname | 4.68 | 4.48 | 4.89 | 1999 |
| Suriname | 4.68 | 4.47 | 4.88 | 2000 |
| Suriname | 4.69 | 4.48 | 4.89 | 2001 |
| Suriname | 4.7 | 4.5 | 4.9 | 2002 |
| Suriname | 4.73 | 4.53 | 4.93 | 2003 |
| Suriname | 4.76 | 4.56 | 4.96 | 2004 |
| Suriname | 4.79 | 4.6 | 4.99 | 2005 |
| Suriname | 4.83 | 4.63 | 5.03 | 2006 |
| Suriname | 4.86 | 4.67 | 5.06 | 2007 |
| Suriname | 4.9 | 4.7 | 5.1 | 2008 |
| Suriname | 4.94 | 4.74 | 5.14 | 2009 |
| Suriname | 4.98 | 4.78 | 5.18 | 2010 |
| Suriname | 5.02 | 4.81 | 5.22 | 2011 |
| Suriname | 5.05 | 4.85 | 5.26 | 2012 |
| Suriname | 5.09 | 4.87 | 5.31 | 2013 |
| Suriname | 5.13 | 4.89 | 5.36 | 2014 |
| Suriname | 5.16 | 4.9 | 5.42 | 2015 |
| Suriname | 5.2 | 4.9 | 5.5 | 2016 |
| Suriname | 5.24 | 4.89 | 5.58 | 2017 |
| Suriname | 5.27 | 4.87 | 5.67 | 2018 |
| Suriname | 5.3 | 4.83 | 5.77 | 2019 |
| Suriname | 5.33 | 4.78 | 5.88 | 2020 |
| Suriname | 5.36 | 4.72 | 6 | 2021 |
| Suriname | 5.39 | 4.66 | 6.12 | 2022 |
| Suriname | 5.42 | 4.58 | 6.25 | 2023 |
| Suriname | 5.44 | 4.5 | 6.38 | 2024 |
| Suriname | 5.46 | 4.41 | 6.51 | 2025 |
| Suriname | 5.48 | 4.32 | 6.65 | 2026 |
| Suriname | 5.5 | 4.21 | 6.79 | 2027 |
| Suriname | 5.52 | 4.1 | 6.94 | 2028 |
| Suriname | 5.54 | 3.99 | 7.09 | 2029 |
| Suriname | 5.55 | 3.87 | 7.24 | 2030 |
| Swaziland | 1.86 | 1.65 | 2.07 | 1990 |
| Swaziland | 2.01 | 1.81 | 2.2 | 1991 |
| Swaziland | 2.16 | 1.98 | 2.34 | 1992 |
| Swaziland | 2.33 | 2.16 | 2.49 | 1993 |
| Swaziland | 2.51 | 2.35 | 2.67 | 1994 |
| Swaziland | 2.7 | 2.54 | 2.86 | 1995 |
| Swaziland | 2.9 | 2.74 | 3.06 | 1996 |
| Swaziland | 3.12 | 2.95 | 3.28 | 1997 |
| Swaziland | 3.33 | 3.17 | 3.5 | 1998 |
| Swaziland | 3.55 | 3.38 | 3.72 | 1999 |
| Swaziland | 3.77 | 3.59 | 3.95 | 2000 |
| Swaziland | 3.98 | 3.8 | 4.16 | 2001 |
| Swaziland | 4.18 | 3.99 | 4.36 | 2002 |
| Swaziland | 4.36 | 4.16 | 4.55 | 2003 |
| Swaziland | 4.51 | 4.32 | 4.71 | 2004 |
| Swaziland | 4.64 | 4.44 | 4.84 | 2005 |
| Swaziland | 4.73 | 4.53 | 4.93 | 2006 |
| Swaziland | 4.79 | 4.58 | 4.99 | 2007 |
| Swaziland | 4.81 | 4.61 | 5.01 | 2008 |
| Swaziland | 4.8 | 4.6 | 5 | 2009 |
| Swaziland | 4.76 | 4.56 | 4.96 | 2010 |
| Swaziland | 4.69 | 4.5 | 4.89 | 2011 |
| Swaziland | 4.61 | 4.41 | 4.8 | 2012 |
| Swaziland | 4.5 | 4.31 | 4.7 | 2013 |
| Swaziland | 4.39 | 4.19 | 4.59 | 2014 |
| Swaziland | 4.27 | 4.05 | 4.49 | 2015 |
| Swaziland | 4.15 | 3.9 | 4.4 | 2016 |
| Swaziland | 4.03 | 3.74 | 4.32 | 2017 |
| Swaziland | 3.91 | 3.56 | 4.27 | 2018 |
| Swaziland | 3.8 | 3.38 | 4.22 | 2019 |
| Swaziland | 3.69 | 3.2 | 4.19 | 2020 |
| Swaziland | 3.58 | 3.01 | 4.16 | 2021 |
| Swaziland | 3.48 | 2.83 | 4.13 | 2022 |
| Swaziland | 3.38 | 2.64 | 4.11 | 2023 |
| Swaziland | 3.28 | 2.46 | 4.1 | 2024 |
| Swaziland | 3.18 | 2.28 | 4.08 | 2025 |
| Swaziland | 3.09 | 2.11 | 4.07 | 2026 |
| Swaziland | 3 | 1.94 | 4.06 | 2027 |
| Swaziland | 2.91 | 1.77 | 4.05 | 2028 |
| Swaziland | 2.82 | 1.61 | 4.04 | 2029 |
| Swaziland | 2.74 | 1.45 | 4.03 | 2030 |
| Sweden | 7.13 | 7.03 | 7.24 | 1990 |
| Sweden | 7.05 | 6.97 | 7.13 | 1991 |
| Sweden | 6.98 | 6.91 | 7.04 | 1992 |
| Sweden | 6.91 | 6.85 | 6.97 | 1993 |
| Sweden | 6.86 | 6.8 | 6.92 | 1994 |
| Sweden | 6.83 | 6.77 | 6.89 | 1995 |
| Sweden | 6.82 | 6.76 | 6.88 | 1996 |
| Sweden | 6.84 | 6.78 | 6.9 | 1997 |
| Sweden | 6.88 | 6.82 | 6.94 | 1998 |
| Sweden | 6.93 | 6.87 | 6.99 | 1999 |
| Sweden | 7 | 6.94 | 7.06 | 2000 |
| Sweden | 7.08 | 7.02 | 7.14 | 2001 |
| Sweden | 7.17 | 7.11 | 7.23 | 2002 |
| Sweden | 7.27 | 7.21 | 7.33 | 2003 |
| Sweden | 7.37 | 7.31 | 7.44 | 2004 |
| Sweden | 7.48 | 7.42 | 7.54 | 2005 |
| Sweden | 7.58 | 7.52 | 7.64 | 2006 |
| Sweden | 7.68 | 7.61 | 7.74 | 2007 |
| Sweden | 7.76 | 7.7 | 7.83 | 2008 |
| Sweden | 7.84 | 7.77 | 7.9 | 2009 |
| Sweden | 7.9 | 7.84 | 7.96 | 2010 |
| Sweden | 7.95 | 7.89 | 8.02 | 2011 |
| Sweden | 7.99 | 7.93 | 8.06 | 2012 |
| Sweden | 8.01 | 7.95 | 8.08 | 2013 |
| Sweden | 8.01 | 7.95 | 8.08 | 2014 |
| Sweden | 7.99 | 7.92 | 8.05 | 2015 |
| Sweden | 7.94 | 7.86 | 8.02 | 2016 |
| Sweden | 7.89 | 7.79 | 8 | 2017 |
| Sweden | 7.85 | 7.7 | 7.99 | 2018 |
| Sweden | 7.8 | 7.6 | 8 | 2019 |
| Sweden | 7.75 | 7.49 | 8.02 | 2020 |
| Sweden | 7.71 | 7.38 | 8.04 | 2021 |
| Sweden | 7.67 | 7.27 | 8.08 | 2022 |
| Sweden | 7.64 | 7.15 | 8.12 | 2023 |
| Sweden | 7.6 | 7.03 | 8.17 | 2024 |
| Sweden | 7.57 | 6.91 | 8.23 | 2025 |
| Sweden | 7.55 | 6.79 | 8.3 | 2026 |
| Sweden | 7.52 | 6.67 | 8.37 | 2027 |
| Sweden | 7.5 | 6.55 | 8.45 | 2028 |
| Sweden | 7.48 | 6.43 | 8.54 | 2029 |
| Sweden | 7.47 | 6.3 | 8.63 | 2030 |
| Switzerland | 5.37 | 5.27 | 5.48 | 1990 |
| Switzerland | 5.55 | 5.46 | 5.63 | 1991 |
| Switzerland | 5.72 | 5.65 | 5.8 | 1992 |
| Switzerland | 5.91 | 5.84 | 5.97 | 1993 |
| Switzerland | 6.09 | 6.02 | 6.16 | 1994 |
| Switzerland | 6.27 | 6.2 | 6.34 | 1995 |
| Switzerland | 6.45 | 6.38 | 6.51 | 1996 |
| Switzerland | 6.62 | 6.55 | 6.68 | 1997 |
| Switzerland | 6.77 | 6.7 | 6.84 | 1998 |
| Switzerland | 6.92 | 6.85 | 6.99 | 1999 |
| Switzerland | 7.04 | 6.98 | 7.11 | 2000 |
| Switzerland | 7.15 | 7.08 | 7.22 | 2001 |
| Switzerland | 7.23 | 7.16 | 7.3 | 2002 |
| Switzerland | 7.29 | 7.22 | 7.36 | 2003 |
| Switzerland | 7.31 | 7.24 | 7.38 | 2004 |
| Switzerland | 7.31 | 7.24 | 7.38 | 2005 |
| Switzerland | 7.28 | 7.22 | 7.35 | 2006 |
| Switzerland | 7.23 | 7.16 | 7.3 | 2007 |
| Switzerland | 7.15 | 7.09 | 7.22 | 2008 |
| Switzerland | 7.06 | 7 | 7.13 | 2009 |
| Switzerland | 6.96 | 6.89 | 7.03 | 2010 |
| Switzerland | 6.85 | 6.79 | 6.92 | 2011 |
| Switzerland | 6.74 | 6.67 | 6.8 | 2012 |
| Switzerland | 6.61 | 6.55 | 6.68 | 2013 |
| Switzerland | 6.48 | 6.42 | 6.54 | 2014 |
| Switzerland | 6.35 | 6.28 | 6.41 | 2015 |
| Switzerland | 6.21 | 6.14 | 6.28 | 2016 |
| Switzerland | 6.08 | 5.98 | 6.17 | 2017 |
| Switzerland | 5.95 | 5.82 | 6.07 | 2018 |
| Switzerland | 5.82 | 5.65 | 5.99 | 2019 |
| Switzerland | 5.69 | 5.47 | 5.91 | 2020 |
| Switzerland | 5.57 | 5.3 | 5.84 | 2021 |
| Switzerland | 5.45 | 5.13 | 5.77 | 2022 |
| Switzerland | 5.33 | 4.96 | 5.71 | 2023 |
| Switzerland | 5.22 | 4.79 | 5.65 | 2024 |
| Switzerland | 5.11 | 4.62 | 5.6 | 2025 |
| Switzerland | 5 | 4.45 | 5.55 | 2026 |
| Switzerland | 4.89 | 4.28 | 5.5 | 2027 |
| Switzerland | 4.79 | 4.12 | 5.45 | 2028 |
| Switzerland | 4.68 | 3.96 | 5.41 | 2029 |
| Switzerland | 4.58 | 3.8 | 5.37 | 2030 |
| Syria | 1.6 | 1.54 | 1.66 | 1990 |
| Syria | 1.63 | 1.58 | 1.68 | 1991 |
| Syria | 1.65 | 1.61 | 1.69 | 1992 |
| Syria | 1.67 | 1.63 | 1.71 | 1993 |
| Syria | 1.69 | 1.66 | 1.73 | 1994 |
| Syria | 1.71 | 1.68 | 1.75 | 1995 |
| Syria | 1.73 | 1.7 | 1.77 | 1996 |
| Syria | 1.75 | 1.71 | 1.78 | 1997 |
| Syria | 1.76 | 1.73 | 1.79 | 1998 |
| Syria | 1.77 | 1.74 | 1.8 | 1999 |
| Syria | 1.78 | 1.75 | 1.81 | 2000 |
| Syria | 1.79 | 1.76 | 1.82 | 2001 |
| Syria | 1.8 | 1.77 | 1.83 | 2002 |
| Syria | 1.82 | 1.79 | 1.85 | 2003 |
| Syria | 1.84 | 1.81 | 1.87 | 2004 |
| Syria | 1.86 | 1.83 | 1.89 | 2005 |
| Syria | 1.89 | 1.86 | 1.93 | 2006 |
| Syria | 1.93 | 1.9 | 1.96 | 2007 |
| Syria | 1.97 | 1.94 | 2.01 | 2008 |
| Syria | 2.02 | 1.99 | 2.05 | 2009 |
| Syria | 2.07 | 2.04 | 2.1 | 2010 |
| Syria | 2.12 | 2.09 | 2.16 | 2011 |
| Syria | 2.18 | 2.15 | 2.21 | 2012 |
| Syria | 2.23 | 2.2 | 2.27 | 2013 |
| Syria | 2.29 | 2.26 | 2.33 | 2014 |
| Syria | 2.35 | 2.31 | 2.39 | 2015 |
| Syria | 2.41 | 2.36 | 2.45 | 2016 |
| Syria | 2.46 | 2.4 | 2.52 | 2017 |
| Syria | 2.52 | 2.44 | 2.6 | 2018 |
| Syria | 2.58 | 2.48 | 2.68 | 2019 |
| Syria | 2.64 | 2.51 | 2.77 | 2020 |
| Syria | 2.7 | 2.54 | 2.87 | 2021 |
| Syria | 2.76 | 2.57 | 2.96 | 2022 |
| Syria | 2.83 | 2.59 | 3.07 | 2023 |
| Syria | 2.89 | 2.61 | 3.17 | 2024 |
| Syria | 2.96 | 2.62 | 3.29 | 2025 |
| Syria | 3.02 | 2.64 | 3.4 | 2026 |
| Syria | 3.09 | 2.65 | 3.53 | 2027 |
| Syria | 3.15 | 2.65 | 3.65 | 2028 |
| Syria | 3.22 | 2.66 | 3.79 | 2029 |
| Syria | 3.29 | 2.66 | 3.92 | 2030 |
| Tajikistan | 5.54 | 5.36 | 5.71 | 1990 |
| Tajikistan | 5.52 | 5.38 | 5.66 | 1991 |
| Tajikistan | 5.5 | 5.39 | 5.62 | 1992 |
| Tajikistan | 5.47 | 5.37 | 5.58 | 1993 |
| Tajikistan | 5.42 | 5.32 | 5.53 | 1994 |
| Tajikistan | 5.35 | 5.25 | 5.45 | 1995 |
| Tajikistan | 5.26 | 5.16 | 5.36 | 1996 |
| Tajikistan | 5.16 | 5.06 | 5.25 | 1997 |
| Tajikistan | 5.05 | 4.95 | 5.14 | 1998 |
| Tajikistan | 4.94 | 4.85 | 5.03 | 1999 |
| Tajikistan | 4.85 | 4.76 | 4.94 | 2000 |
| Tajikistan | 4.78 | 4.69 | 4.87 | 2001 |
| Tajikistan | 4.73 | 4.65 | 4.82 | 2002 |
| Tajikistan | 4.72 | 4.63 | 4.8 | 2003 |
| Tajikistan | 4.73 | 4.65 | 4.81 | 2004 |
| Tajikistan | 4.77 | 4.68 | 4.85 | 2005 |
| Tajikistan | 4.82 | 4.74 | 4.9 | 2006 |
| Tajikistan | 4.9 | 4.82 | 4.98 | 2007 |
| Tajikistan | 4.98 | 4.9 | 5.06 | 2008 |
| Tajikistan | 5.07 | 4.99 | 5.16 | 2009 |
| Tajikistan | 5.17 | 5.09 | 5.26 | 2010 |
| Tajikistan | 5.28 | 5.19 | 5.36 | 2011 |
| Tajikistan | 5.38 | 5.3 | 5.47 | 2012 |
| Tajikistan | 5.49 | 5.41 | 5.58 | 2013 |
| Tajikistan | 5.6 | 5.52 | 5.69 | 2014 |
| Tajikistan | 5.72 | 5.63 | 5.81 | 2015 |
| Tajikistan | 5.84 | 5.73 | 5.94 | 2016 |
| Tajikistan | 5.95 | 5.81 | 6.09 | 2017 |
| Tajikistan | 6.07 | 5.88 | 6.26 | 2018 |
| Tajikistan | 6.19 | 5.93 | 6.45 | 2019 |
| Tajikistan | 6.32 | 5.97 | 6.66 | 2020 |
| Tajikistan | 6.44 | 6 | 6.88 | 2021 |
| Tajikistan | 6.57 | 6.02 | 7.12 | 2022 |
| Tajikistan | 6.7 | 6.04 | 7.37 | 2023 |
| Tajikistan | 6.84 | 6.04 | 7.64 | 2024 |
| Tajikistan | 6.98 | 6.03 | 7.93 | 2025 |
| Tajikistan | 7.12 | 6.02 | 8.22 | 2026 |
| Tajikistan | 7.27 | 6 | 8.54 | 2027 |
| Tajikistan | 7.42 | 5.97 | 8.87 | 2028 |
| Tajikistan | 7.57 | 5.92 | 9.22 | 2029 |
| Tajikistan | 7.73 | 5.87 | 9.58 | 2030 |
| Tanzania | 2.22 | 2.17 | 2.27 | 1990 |
| Tanzania | 2.22 | 2.18 | 2.26 | 1991 |
| Tanzania | 2.22 | 2.19 | 2.26 | 1992 |
| Tanzania | 2.22 | 2.19 | 2.26 | 1993 |
| Tanzania | 2.23 | 2.2 | 2.25 | 1994 |
| Tanzania | 2.23 | 2.2 | 2.25 | 1995 |
| Tanzania | 2.23 | 2.2 | 2.25 | 1996 |
| Tanzania | 2.23 | 2.2 | 2.26 | 1997 |
| Tanzania | 2.23 | 2.21 | 2.26 | 1998 |
| Tanzania | 2.24 | 2.21 | 2.26 | 1999 |
| Tanzania | 2.24 | 2.22 | 2.27 | 2000 |
| Tanzania | 2.25 | 2.23 | 2.28 | 2001 |
| Tanzania | 2.27 | 2.25 | 2.3 | 2002 |
| Tanzania | 2.29 | 2.27 | 2.32 | 2003 |
| Tanzania | 2.32 | 2.3 | 2.34 | 2004 |
| Tanzania | 2.35 | 2.33 | 2.37 | 2005 |
| Tanzania | 2.38 | 2.36 | 2.41 | 2006 |
| Tanzania | 2.42 | 2.39 | 2.44 | 2007 |
| Tanzania | 2.45 | 2.43 | 2.47 | 2008 |
| Tanzania | 2.49 | 2.46 | 2.51 | 2009 |
| Tanzania | 2.52 | 2.5 | 2.54 | 2010 |
| Tanzania | 2.55 | 2.53 | 2.58 | 2011 |
| Tanzania | 2.59 | 2.56 | 2.61 | 2012 |
| Tanzania | 2.62 | 2.59 | 2.64 | 2013 |
| Tanzania | 2.64 | 2.62 | 2.66 | 2014 |
| Tanzania | 2.66 | 2.64 | 2.69 | 2015 |
| Tanzania | 2.68 | 2.65 | 2.71 | 2016 |
| Tanzania | 2.7 | 2.66 | 2.74 | 2017 |
| Tanzania | 2.72 | 2.67 | 2.77 | 2018 |
| Tanzania | 2.74 | 2.67 | 2.81 | 2019 |
| Tanzania | 2.76 | 2.67 | 2.85 | 2020 |
| Tanzania | 2.77 | 2.66 | 2.89 | 2021 |
| Tanzania | 2.79 | 2.66 | 2.93 | 2022 |
| Tanzania | 2.81 | 2.65 | 2.98 | 2023 |
| Tanzania | 2.83 | 2.64 | 3.03 | 2024 |
| Tanzania | 2.85 | 2.62 | 3.08 | 2025 |
| Tanzania | 2.87 | 2.61 | 3.13 | 2026 |
| Tanzania | 2.89 | 2.59 | 3.18 | 2027 |
| Tanzania | 2.91 | 2.57 | 3.24 | 2028 |
| Tanzania | 2.92 | 2.55 | 3.3 | 2029 |
| Tanzania | 2.94 | 2.53 | 3.36 | 2030 |
| Thailand | 2.18 | 2.15 | 2.22 | 1990 |
| Thailand | 2.25 | 2.22 | 2.27 | 1991 |
| Thailand | 2.31 | 2.29 | 2.34 | 1992 |
| Thailand | 2.38 | 2.36 | 2.41 | 1993 |
| Thailand | 2.46 | 2.43 | 2.48 | 1994 |
| Thailand | 2.52 | 2.5 | 2.55 | 1995 |
| Thailand | 2.58 | 2.56 | 2.6 | 1996 |
| Thailand | 2.62 | 2.6 | 2.64 | 1997 |
| Thailand | 2.64 | 2.62 | 2.67 | 1998 |
| Thailand | 2.65 | 2.63 | 2.68 | 1999 |
| Thailand | 2.64 | 2.62 | 2.67 | 2000 |
| Thailand | 2.62 | 2.6 | 2.64 | 2001 |
| Thailand | 2.58 | 2.56 | 2.6 | 2002 |
| Thailand | 2.54 | 2.52 | 2.56 | 2003 |
| Thailand | 2.5 | 2.48 | 2.52 | 2004 |
| Thailand | 2.46 | 2.44 | 2.48 | 2005 |
| Thailand | 2.43 | 2.41 | 2.45 | 2006 |
| Thailand | 2.41 | 2.4 | 2.43 | 2007 |
| Thailand | 2.4 | 2.38 | 2.42 | 2008 |
| Thailand | 2.4 | 2.38 | 2.42 | 2009 |
| Thailand | 2.41 | 2.39 | 2.43 | 2010 |
| Thailand | 2.43 | 2.41 | 2.45 | 2011 |
| Thailand | 2.45 | 2.43 | 2.47 | 2012 |
| Thailand | 2.48 | 2.46 | 2.5 | 2013 |
| Thailand | 2.52 | 2.5 | 2.54 | 2014 |
| Thailand | 2.57 | 2.55 | 2.59 | 2015 |
| Thailand | 2.63 | 2.61 | 2.65 | 2016 |
| Thailand | 2.68 | 2.65 | 2.71 | 2017 |
| Thailand | 2.74 | 2.69 | 2.79 | 2018 |
| Thailand | 2.8 | 2.72 | 2.87 | 2019 |
| Thailand | 2.86 | 2.75 | 2.97 | 2020 |
| Thailand | 2.92 | 2.78 | 3.07 | 2021 |
| Thailand | 2.98 | 2.8 | 3.17 | 2022 |
| Thailand | 3.05 | 2.82 | 3.28 | 2023 |
| Thailand | 3.11 | 2.83 | 3.4 | 2024 |
| Thailand | 3.18 | 2.84 | 3.52 | 2025 |
| Thailand | 3.25 | 2.84 | 3.65 | 2026 |
| Thailand | 3.31 | 2.84 | 3.79 | 2027 |
| Thailand | 3.38 | 2.84 | 3.93 | 2028 |
| Thailand | 3.45 | 2.83 | 4.08 | 2029 |
| Thailand | 3.52 | 2.81 | 4.23 | 2030 |
| The Bahamas | 6.68 | 6.1 | 7.25 | 1990 |
| The Bahamas | 6.59 | 6.09 | 7.1 | 1991 |
| The Bahamas | 6.51 | 6.06 | 6.96 | 1992 |
| The Bahamas | 6.43 | 6.03 | 6.83 | 1993 |
| The Bahamas | 6.35 | 5.99 | 6.71 | 1994 |
| The Bahamas | 6.27 | 5.94 | 6.6 | 1995 |
| The Bahamas | 6.19 | 5.88 | 6.5 | 1996 |
| The Bahamas | 6.11 | 5.82 | 6.41 | 1997 |
| The Bahamas | 6.04 | 5.76 | 6.32 | 1998 |
| The Bahamas | 5.97 | 5.7 | 6.24 | 1999 |
| The Bahamas | 5.91 | 5.64 | 6.17 | 2000 |
| The Bahamas | 5.85 | 5.59 | 6.11 | 2001 |
| The Bahamas | 5.81 | 5.55 | 6.06 | 2002 |
| The Bahamas | 5.77 | 5.52 | 6.02 | 2003 |
| The Bahamas | 5.73 | 5.49 | 5.98 | 2004 |
| The Bahamas | 5.71 | 5.47 | 5.95 | 2005 |
| The Bahamas | 5.69 | 5.45 | 5.93 | 2006 |
| The Bahamas | 5.68 | 5.44 | 5.91 | 2007 |
| The Bahamas | 5.67 | 5.44 | 5.9 | 2008 |
| The Bahamas | 5.67 | 5.43 | 5.9 | 2009 |
| The Bahamas | 5.66 | 5.43 | 5.9 | 2010 |
| The Bahamas | 5.66 | 5.43 | 5.9 | 2011 |
| The Bahamas | 5.66 | 5.42 | 5.9 | 2012 |
| The Bahamas | 5.66 | 5.41 | 5.91 | 2013 |
| The Bahamas | 5.66 | 5.38 | 5.93 | 2014 |
| The Bahamas | 5.65 | 5.35 | 5.95 | 2015 |
| The Bahamas | 5.65 | 5.31 | 5.98 | 2016 |
| The Bahamas | 5.64 | 5.26 | 6.02 | 2017 |
| The Bahamas | 5.64 | 5.2 | 6.07 | 2018 |
| The Bahamas | 5.63 | 5.13 | 6.13 | 2019 |
| The Bahamas | 5.62 | 5.05 | 6.19 | 2020 |
| The Bahamas | 5.61 | 4.97 | 6.26 | 2021 |
| The Bahamas | 5.6 | 4.88 | 6.33 | 2022 |
| The Bahamas | 5.59 | 4.78 | 6.41 | 2023 |
| The Bahamas | 5.58 | 4.68 | 6.49 | 2024 |
| The Bahamas | 5.57 | 4.57 | 6.57 | 2025 |
| The Bahamas | 5.56 | 4.46 | 6.65 | 2026 |
| The Bahamas | 5.55 | 4.35 | 6.74 | 2027 |
| The Bahamas | 5.53 | 4.23 | 6.83 | 2028 |
| The Bahamas | 5.52 | 4.11 | 6.93 | 2029 |
| The Bahamas | 5.51 | 3.99 | 7.02 | 2030 |
| The Gambia | 1.7 | 1.52 | 1.88 | 1990 |
| The Gambia | 1.75 | 1.59 | 1.92 | 1991 |
| The Gambia | 1.81 | 1.65 | 1.96 | 1992 |
| The Gambia | 1.87 | 1.72 | 2.01 | 1993 |
| The Gambia | 1.92 | 1.79 | 2.06 | 1994 |
| The Gambia | 1.98 | 1.85 | 2.11 | 1995 |
| The Gambia | 2.04 | 1.91 | 2.16 | 1996 |
| The Gambia | 2.09 | 1.97 | 2.21 | 1997 |
| The Gambia | 2.15 | 2.03 | 2.27 | 1998 |
| The Gambia | 2.21 | 2.09 | 2.32 | 1999 |
| The Gambia | 2.27 | 2.15 | 2.38 | 2000 |
| The Gambia | 2.32 | 2.21 | 2.44 | 2001 |
| The Gambia | 2.38 | 2.27 | 2.49 | 2002 |
| The Gambia | 2.44 | 2.32 | 2.55 | 2003 |
| The Gambia | 2.49 | 2.38 | 2.6 | 2004 |
| The Gambia | 2.55 | 2.43 | 2.66 | 2005 |
| The Gambia | 2.6 | 2.49 | 2.71 | 2006 |
| The Gambia | 2.65 | 2.54 | 2.77 | 2007 |
| The Gambia | 2.71 | 2.59 | 2.82 | 2008 |
| The Gambia | 2.76 | 2.64 | 2.87 | 2009 |
| The Gambia | 2.81 | 2.69 | 2.93 | 2010 |
| The Gambia | 2.86 | 2.73 | 2.98 | 2011 |
| The Gambia | 2.91 | 2.78 | 3.04 | 2012 |
| The Gambia | 2.96 | 2.82 | 3.09 | 2013 |
| The Gambia | 3.01 | 2.86 | 3.15 | 2014 |
| The Gambia | 3.05 | 2.89 | 3.22 | 2015 |
| The Gambia | 3.1 | 2.92 | 3.29 | 2016 |
| The Gambia | 3.16 | 2.95 | 3.37 | 2017 |
| The Gambia | 3.21 | 2.97 | 3.45 | 2018 |
| The Gambia | 3.26 | 2.98 | 3.54 | 2019 |
| The Gambia | 3.32 | 2.99 | 3.64 | 2020 |
| The Gambia | 3.37 | 3 | 3.75 | 2021 |
| The Gambia | 3.43 | 3.01 | 3.86 | 2022 |
| The Gambia | 3.5 | 3.01 | 3.99 | 2023 |
| The Gambia | 3.56 | 3.01 | 4.12 | 2024 |
| The Gambia | 3.63 | 3.01 | 4.26 | 2025 |
| The Gambia | 3.71 | 3 | 4.41 | 2026 |
| The Gambia | 3.78 | 2.99 | 4.57 | 2027 |
| The Gambia | 3.86 | 2.98 | 4.74 | 2028 |
| The Gambia | 3.95 | 2.97 | 4.93 | 2029 |
| The Gambia | 4.04 | 2.96 | 5.12 | 2030 |
| Timor-Leste | 2.2 | 1.97 | 2.43 | 1990 |
| Timor-Leste | 2.24 | 2.03 | 2.45 | 1991 |
| Timor-Leste | 2.28 | 2.08 | 2.47 | 1992 |
| Timor-Leste | 2.31 | 2.13 | 2.49 | 1993 |
| Timor-Leste | 2.34 | 2.18 | 2.51 | 1994 |
| Timor-Leste | 2.38 | 2.22 | 2.54 | 1995 |
| Timor-Leste | 2.41 | 2.26 | 2.56 | 1996 |
| Timor-Leste | 2.44 | 2.3 | 2.59 | 1997 |
| Timor-Leste | 2.48 | 2.34 | 2.62 | 1998 |
| Timor-Leste | 2.51 | 2.38 | 2.65 | 1999 |
| Timor-Leste | 2.55 | 2.42 | 2.69 | 2000 |
| Timor-Leste | 2.59 | 2.46 | 2.73 | 2001 |
| Timor-Leste | 2.64 | 2.51 | 2.77 | 2002 |
| Timor-Leste | 2.68 | 2.55 | 2.82 | 2003 |
| Timor-Leste | 2.74 | 2.6 | 2.87 | 2004 |
| Timor-Leste | 2.79 | 2.66 | 2.92 | 2005 |
| Timor-Leste | 2.85 | 2.71 | 2.98 | 2006 |
| Timor-Leste | 2.91 | 2.77 | 3.04 | 2007 |
| Timor-Leste | 2.97 | 2.84 | 3.1 | 2008 |
| Timor-Leste | 3.04 | 2.9 | 3.17 | 2009 |
| Timor-Leste | 3.1 | 2.97 | 3.24 | 2010 |
| Timor-Leste | 3.17 | 3.03 | 3.32 | 2011 |
| Timor-Leste | 3.24 | 3.09 | 3.39 | 2012 |
| Timor-Leste | 3.32 | 3.16 | 3.48 | 2013 |
| Timor-Leste | 3.39 | 3.21 | 3.56 | 2014 |
| Timor-Leste | 3.46 | 3.27 | 3.65 | 2015 |
| Timor-Leste | 3.53 | 3.31 | 3.75 | 2016 |
| Timor-Leste | 3.6 | 3.35 | 3.86 | 2017 |
| Timor-Leste | 3.68 | 3.38 | 3.97 | 2018 |
| Timor-Leste | 3.75 | 3.41 | 4.09 | 2019 |
| Timor-Leste | 3.82 | 3.42 | 4.22 | 2020 |
| Timor-Leste | 3.89 | 3.43 | 4.35 | 2021 |
| Timor-Leste | 3.96 | 3.44 | 4.49 | 2022 |
| Timor-Leste | 4.04 | 3.43 | 4.64 | 2023 |
| Timor-Leste | 4.11 | 3.42 | 4.79 | 2024 |
| Timor-Leste | 4.18 | 3.41 | 4.95 | 2025 |
| Timor-Leste | 4.25 | 3.39 | 5.11 | 2026 |
| Timor-Leste | 4.32 | 3.36 | 5.28 | 2027 |
| Timor-Leste | 4.39 | 3.32 | 5.46 | 2028 |
| Timor-Leste | 4.46 | 3.28 | 5.63 | 2029 |
| Timor-Leste | 4.53 | 3.24 | 5.82 | 2030 |
| Togo | 1.56 | 1.47 | 1.65 | 1990 |
| Togo | 1.6 | 1.52 | 1.67 | 1991 |
| Togo | 1.63 | 1.57 | 1.7 | 1992 |
| Togo | 1.67 | 1.61 | 1.74 | 1993 |
| Togo | 1.71 | 1.65 | 1.77 | 1994 |
| Togo | 1.76 | 1.7 | 1.81 | 1995 |
| Togo | 1.8 | 1.74 | 1.85 | 1996 |
| Togo | 1.84 | 1.79 | 1.9 | 1997 |
| Togo | 1.89 | 1.84 | 1.94 | 1998 |
| Togo | 1.94 | 1.89 | 1.99 | 1999 |
| Togo | 1.99 | 1.94 | 2.04 | 2000 |
| Togo | 2.04 | 1.99 | 2.09 | 2001 |
| Togo | 2.09 | 2.04 | 2.14 | 2002 |
| Togo | 2.14 | 2.09 | 2.2 | 2003 |
| Togo | 2.2 | 2.14 | 2.25 | 2004 |
| Togo | 2.25 | 2.2 | 2.3 | 2005 |
| Togo | 2.3 | 2.25 | 2.36 | 2006 |
| Togo | 2.36 | 2.3 | 2.41 | 2007 |
| Togo | 2.41 | 2.36 | 2.47 | 2008 |
| Togo | 2.47 | 2.41 | 2.52 | 2009 |
| Togo | 2.52 | 2.46 | 2.58 | 2010 |
| Togo | 2.57 | 2.51 | 2.63 | 2011 |
| Togo | 2.62 | 2.56 | 2.68 | 2012 |
| Togo | 2.67 | 2.61 | 2.73 | 2013 |
| Togo | 2.72 | 2.65 | 2.78 | 2014 |
| Togo | 2.76 | 2.69 | 2.84 | 2015 |
| Togo | 2.81 | 2.72 | 2.89 | 2016 |
| Togo | 2.85 | 2.75 | 2.95 | 2017 |
| Togo | 2.9 | 2.77 | 3.02 | 2018 |
| Togo | 2.94 | 2.79 | 3.09 | 2019 |
| Togo | 2.98 | 2.8 | 3.16 | 2020 |
| Togo | 3.02 | 2.81 | 3.23 | 2021 |
| Togo | 3.06 | 2.82 | 3.31 | 2022 |
| Togo | 3.1 | 2.82 | 3.39 | 2023 |
| Togo | 3.14 | 2.81 | 3.48 | 2024 |
| Togo | 3.18 | 2.8 | 3.56 | 2025 |
| Togo | 3.22 | 2.79 | 3.65 | 2026 |
| Togo | 3.26 | 2.78 | 3.74 | 2027 |
| Togo | 3.29 | 2.76 | 3.83 | 2028 |
| Togo | 3.33 | 2.73 | 3.92 | 2029 |
| Togo | 3.36 | 2.71 | 4.02 | 2030 |
| Trinidad and Tobago | 9.54 | 9.12 | 9.95 | 1990 |
| Trinidad and Tobago | 9.34 | 9.01 | 9.66 | 1991 |
| Trinidad and Tobago | 9.13 | 8.86 | 9.4 | 1992 |
| Trinidad and Tobago | 8.9 | 8.66 | 9.14 | 1993 |
| Trinidad and Tobago | 8.65 | 8.42 | 8.87 | 1994 |
| Trinidad and Tobago | 8.35 | 8.13 | 8.57 | 1995 |
| Trinidad and Tobago | 8.02 | 7.81 | 8.24 | 1996 |
| Trinidad and Tobago | 7.67 | 7.47 | 7.87 | 1997 |
| Trinidad and Tobago | 7.31 | 7.12 | 7.5 | 1998 |
| Trinidad and Tobago | 6.95 | 6.77 | 7.14 | 1999 |
| Trinidad and Tobago | 6.63 | 6.45 | 6.81 | 2000 |
| Trinidad and Tobago | 6.34 | 6.17 | 6.51 | 2001 |
| Trinidad and Tobago | 6.1 | 5.94 | 6.27 | 2002 |
| Trinidad and Tobago | 5.91 | 5.75 | 6.07 | 2003 |
| Trinidad and Tobago | 5.76 | 5.6 | 5.92 | 2004 |
| Trinidad and Tobago | 5.64 | 5.49 | 5.8 | 2005 |
| Trinidad and Tobago | 5.56 | 5.41 | 5.72 | 2006 |
| Trinidad and Tobago | 5.51 | 5.36 | 5.66 | 2007 |
| Trinidad and Tobago | 5.49 | 5.34 | 5.64 | 2008 |
| Trinidad and Tobago | 5.48 | 5.34 | 5.63 | 2009 |
| Trinidad and Tobago | 5.49 | 5.35 | 5.64 | 2010 |
| Trinidad and Tobago | 5.51 | 5.36 | 5.66 | 2011 |
| Trinidad and Tobago | 5.54 | 5.39 | 5.68 | 2012 |
| Trinidad and Tobago | 5.57 | 5.42 | 5.72 | 2013 |
| Trinidad and Tobago | 5.6 | 5.45 | 5.76 | 2014 |
| Trinidad and Tobago | 5.64 | 5.47 | 5.81 | 2015 |
| Trinidad and Tobago | 5.68 | 5.48 | 5.88 | 2016 |
| Trinidad and Tobago | 5.72 | 5.47 | 5.97 | 2017 |
| Trinidad and Tobago | 5.76 | 5.44 | 6.09 | 2018 |
| Trinidad and Tobago | 5.8 | 5.39 | 6.21 | 2019 |
| Trinidad and Tobago | 5.84 | 5.32 | 6.36 | 2020 |
| Trinidad and Tobago | 5.88 | 5.25 | 6.51 | 2021 |
| Trinidad and Tobago | 5.92 | 5.16 | 6.68 | 2022 |
| Trinidad and Tobago | 5.96 | 5.06 | 6.86 | 2023 |
| Trinidad and Tobago | 6 | 4.95 | 7.04 | 2024 |
| Trinidad and Tobago | 6.04 | 4.83 | 7.24 | 2025 |
| Trinidad and Tobago | 6.08 | 4.71 | 7.45 | 2026 |
| Trinidad and Tobago | 6.12 | 4.57 | 7.66 | 2027 |
| Trinidad and Tobago | 6.16 | 4.42 | 7.89 | 2028 |
| Trinidad and Tobago | 6.2 | 4.27 | 8.12 | 2029 |
| Trinidad and Tobago | 6.24 | 4.11 | 8.37 | 2030 |
| Tunisia | 1.84 | 1.78 | 1.91 | 1990 |
| Tunisia | 1.86 | 1.81 | 1.92 | 1991 |
| Tunisia | 1.89 | 1.84 | 1.93 | 1992 |
| Tunisia | 1.91 | 1.87 | 1.95 | 1993 |
| Tunisia | 1.94 | 1.9 | 1.97 | 1994 |
| Tunisia | 1.96 | 1.92 | 2 | 1995 |
| Tunisia | 1.98 | 1.94 | 2.02 | 1996 |
| Tunisia | 2 | 1.97 | 2.04 | 1997 |
| Tunisia | 2.02 | 1.99 | 2.06 | 1998 |
| Tunisia | 2.04 | 2.01 | 2.08 | 1999 |
| Tunisia | 2.06 | 2.03 | 2.1 | 2000 |
| Tunisia | 2.08 | 2.04 | 2.11 | 2001 |
| Tunisia | 2.1 | 2.06 | 2.13 | 2002 |
| Tunisia | 2.11 | 2.08 | 2.15 | 2003 |
| Tunisia | 2.13 | 2.09 | 2.16 | 2004 |
| Tunisia | 2.14 | 2.11 | 2.18 | 2005 |
| Tunisia | 2.16 | 2.12 | 2.19 | 2006 |
| Tunisia | 2.17 | 2.14 | 2.2 | 2007 |
| Tunisia | 2.18 | 2.15 | 2.22 | 2008 |
| Tunisia | 2.2 | 2.16 | 2.23 | 2009 |
| Tunisia | 2.21 | 2.18 | 2.24 | 2010 |
| Tunisia | 2.22 | 2.19 | 2.26 | 2011 |
| Tunisia | 2.23 | 2.2 | 2.27 | 2012 |
| Tunisia | 2.25 | 2.21 | 2.28 | 2013 |
| Tunisia | 2.26 | 2.22 | 2.29 | 2014 |
| Tunisia | 2.27 | 2.23 | 2.3 | 2015 |
| Tunisia | 2.27 | 2.23 | 2.32 | 2016 |
| Tunisia | 2.28 | 2.23 | 2.34 | 2017 |
| Tunisia | 2.29 | 2.22 | 2.36 | 2018 |
| Tunisia | 2.3 | 2.21 | 2.38 | 2019 |
| Tunisia | 2.3 | 2.19 | 2.41 | 2020 |
| Tunisia | 2.31 | 2.18 | 2.44 | 2021 |
| Tunisia | 2.31 | 2.16 | 2.47 | 2022 |
| Tunisia | 2.32 | 2.14 | 2.5 | 2023 |
| Tunisia | 2.32 | 2.12 | 2.53 | 2024 |
| Tunisia | 2.33 | 2.09 | 2.56 | 2025 |
| Tunisia | 2.33 | 2.06 | 2.59 | 2026 |
| Tunisia | 2.33 | 2.03 | 2.63 | 2027 |
| Tunisia | 2.33 | 2 | 2.66 | 2028 |
| Tunisia | 2.33 | 1.97 | 2.69 | 2029 |
| Tunisia | 2.33 | 1.93 | 2.72 | 2030 |
| Turkey | 4.76 | 4.7 | 4.82 | 1990 |
| Turkey | 4.82 | 4.78 | 4.86 | 1991 |
| Turkey | 4.88 | 4.84 | 4.92 | 1992 |
| Turkey | 4.93 | 4.9 | 4.97 | 1993 |
| Turkey | 4.97 | 4.93 | 5.01 | 1994 |
| Turkey | 4.98 | 4.94 | 5.02 | 1995 |
| Turkey | 4.96 | 4.92 | 5 | 1996 |
| Turkey | 4.91 | 4.88 | 4.95 | 1997 |
| Turkey | 4.84 | 4.81 | 4.88 | 1998 |
| Turkey | 4.76 | 4.73 | 4.79 | 1999 |
| Turkey | 4.67 | 4.64 | 4.71 | 2000 |
| Turkey | 4.61 | 4.58 | 4.65 | 2001 |
| Turkey | 4.59 | 4.56 | 4.62 | 2002 |
| Turkey | 4.61 | 4.58 | 4.64 | 2003 |
| Turkey | 4.67 | 4.64 | 4.71 | 2004 |
| Turkey | 4.76 | 4.73 | 4.79 | 2005 |
| Turkey | 4.85 | 4.82 | 4.88 | 2006 |
| Turkey | 4.93 | 4.9 | 4.97 | 2007 |
| Turkey | 5.02 | 4.99 | 5.05 | 2008 |
| Turkey | 5.1 | 5.07 | 5.14 | 2009 |
| Turkey | 5.16 | 5.13 | 5.19 | 2010 |
| Turkey | 5.17 | 5.14 | 5.21 | 2011 |
| Turkey | 5.16 | 5.13 | 5.19 | 2012 |
| Turkey | 5.12 | 5.09 | 5.15 | 2013 |
| Turkey | 5.06 | 5.03 | 5.09 | 2014 |
| Turkey | 4.99 | 4.96 | 5.02 | 2015 |
| Turkey | 4.91 | 4.88 | 4.94 | 2016 |
| Turkey | 4.83 | 4.79 | 4.87 | 2017 |
| Turkey | 4.75 | 4.68 | 4.83 | 2018 |
| Turkey | 4.68 | 4.56 | 4.8 | 2019 |
| Turkey | 4.61 | 4.43 | 4.78 | 2020 |
| Turkey | 4.54 | 4.3 | 4.77 | 2021 |
| Turkey | 4.46 | 4.17 | 4.76 | 2022 |
| Turkey | 4.39 | 4.03 | 4.75 | 2023 |
| Turkey | 4.32 | 3.89 | 4.75 | 2024 |
| Turkey | 4.24 | 3.74 | 4.75 | 2025 |
| Turkey | 4.17 | 3.59 | 4.74 | 2026 |
| Turkey | 4.09 | 3.45 | 4.74 | 2027 |
| Turkey | 4.02 | 3.3 | 4.74 | 2028 |
| Turkey | 3.95 | 3.15 | 4.74 | 2029 |
| Turkey | 3.87 | 3 | 4.74 | 2030 |
| Turkmenistan | 6.71 | 6.5 | 6.91 | 1990 |
| Turkmenistan | 6.76 | 6.59 | 6.94 | 1991 |
| Turkmenistan | 6.82 | 6.67 | 6.97 | 1992 |
| Turkmenistan | 6.87 | 6.73 | 7 | 1993 |
| Turkmenistan | 6.91 | 6.78 | 7.03 | 1994 |
| Turkmenistan | 6.95 | 6.82 | 7.07 | 1995 |
| Turkmenistan | 6.97 | 6.86 | 7.09 | 1996 |
| Turkmenistan | 7 | 6.88 | 7.12 | 1997 |
| Turkmenistan | 7.02 | 6.91 | 7.14 | 1998 |
| Turkmenistan | 7.05 | 6.93 | 7.16 | 1999 |
| Turkmenistan | 7.09 | 6.97 | 7.2 | 2000 |
| Turkmenistan | 7.14 | 7.03 | 7.26 | 2001 |
| Turkmenistan | 7.21 | 7.1 | 7.33 | 2002 |
| Turkmenistan | 7.3 | 7.19 | 7.42 | 2003 |
| Turkmenistan | 7.4 | 7.29 | 7.52 | 2004 |
| Turkmenistan | 7.51 | 7.4 | 7.62 | 2005 |
| Turkmenistan | 7.61 | 7.5 | 7.72 | 2006 |
| Turkmenistan | 7.7 | 7.59 | 7.81 | 2007 |
| Turkmenistan | 7.77 | 7.66 | 7.89 | 2008 |
| Turkmenistan | 7.83 | 7.72 | 7.95 | 2009 |
| Turkmenistan | 7.88 | 7.77 | 8 | 2010 |
| Turkmenistan | 7.92 | 7.81 | 8.03 | 2011 |
| Turkmenistan | 7.94 | 7.83 | 8.05 | 2012 |
| Turkmenistan | 7.95 | 7.84 | 8.07 | 2013 |
| Turkmenistan | 7.95 | 7.84 | 8.07 | 2014 |
| Turkmenistan | 7.94 | 7.82 | 8.07 | 2015 |
| Turkmenistan | 7.93 | 7.79 | 8.08 | 2016 |
| Turkmenistan | 7.92 | 7.74 | 8.1 | 2017 |
| Turkmenistan | 7.91 | 7.68 | 8.14 | 2018 |
| Turkmenistan | 7.9 | 7.6 | 8.19 | 2019 |
| Turkmenistan | 7.89 | 7.52 | 8.26 | 2020 |
| Turkmenistan | 7.88 | 7.43 | 8.33 | 2021 |
| Turkmenistan | 7.87 | 7.33 | 8.41 | 2022 |
| Turkmenistan | 7.86 | 7.22 | 8.49 | 2023 |
| Turkmenistan | 7.85 | 7.11 | 8.58 | 2024 |
| Turkmenistan | 7.84 | 7 | 8.68 | 2025 |
| Turkmenistan | 7.83 | 6.88 | 8.78 | 2026 |
| Turkmenistan | 7.82 | 6.75 | 8.88 | 2027 |
| Turkmenistan | 7.8 | 6.62 | 8.99 | 2028 |
| Turkmenistan | 7.79 | 6.48 | 9.1 | 2029 |
| Turkmenistan | 7.78 | 6.34 | 9.21 | 2030 |
| Uganda | 1.48 | 1.43 | 1.53 | 1990 |
| Uganda | 1.48 | 1.44 | 1.52 | 1991 |
| Uganda | 1.48 | 1.45 | 1.52 | 1992 |
| Uganda | 1.49 | 1.46 | 1.52 | 1993 |
| Uganda | 1.5 | 1.47 | 1.53 | 1994 |
| Uganda | 1.51 | 1.48 | 1.54 | 1995 |
| Uganda | 1.53 | 1.5 | 1.56 | 1996 |
| Uganda | 1.56 | 1.53 | 1.59 | 1997 |
| Uganda | 1.59 | 1.57 | 1.62 | 1998 |
| Uganda | 1.64 | 1.61 | 1.66 | 1999 |
| Uganda | 1.69 | 1.66 | 1.71 | 2000 |
| Uganda | 1.74 | 1.71 | 1.77 | 2001 |
| Uganda | 1.79 | 1.77 | 1.82 | 2002 |
| Uganda | 1.84 | 1.82 | 1.87 | 2003 |
| Uganda | 1.89 | 1.86 | 1.92 | 2004 |
| Uganda | 1.92 | 1.89 | 1.95 | 2005 |
| Uganda | 1.95 | 1.92 | 1.98 | 2006 |
| Uganda | 1.98 | 1.95 | 2.01 | 2007 |
| Uganda | 2.01 | 1.98 | 2.03 | 2008 |
| Uganda | 2.04 | 2.01 | 2.07 | 2009 |
| Uganda | 2.07 | 2.04 | 2.1 | 2010 |
| Uganda | 2.11 | 2.08 | 2.14 | 2011 |
| Uganda | 2.14 | 2.11 | 2.17 | 2012 |
| Uganda | 2.16 | 2.13 | 2.19 | 2013 |
| Uganda | 2.17 | 2.14 | 2.2 | 2014 |
| Uganda | 2.15 | 2.12 | 2.18 | 2015 |
| Uganda | 2.13 | 2.09 | 2.16 | 2016 |
| Uganda | 2.1 | 2.05 | 2.15 | 2017 |
| Uganda | 2.07 | 2.01 | 2.14 | 2018 |
| Uganda | 2.04 | 1.96 | 2.13 | 2019 |
| Uganda | 2.02 | 1.91 | 2.13 | 2020 |
| Uganda | 1.99 | 1.85 | 2.13 | 2021 |
| Uganda | 1.96 | 1.79 | 2.13 | 2022 |
| Uganda | 1.94 | 1.74 | 2.14 | 2023 |
| Uganda | 1.91 | 1.68 | 2.14 | 2024 |
| Uganda | 1.89 | 1.62 | 2.15 | 2025 |
| Uganda | 1.86 | 1.56 | 2.16 | 2026 |
| Uganda | 1.84 | 1.51 | 2.17 | 2027 |
| Uganda | 1.81 | 1.45 | 2.18 | 2028 |
| Uganda | 1.79 | 1.39 | 2.19 | 2029 |
| Uganda | 1.77 | 1.33 | 2.21 | 2030 |
| Ukraine | 7.7 | 7.63 | 7.77 | 1990 |
| Ukraine | 7.96 | 7.89 | 8.03 | 1991 |
| Ukraine | 8.21 | 8.14 | 8.28 | 1992 |
| Ukraine | 8.43 | 8.36 | 8.49 | 1993 |
| Ukraine | 8.68 | 8.61 | 8.75 | 1994 |
| Ukraine | 9.04 | 8.97 | 9.11 | 1995 |
| Ukraine | 8.75 | 8.68 | 8.82 | 1996 |
| Ukraine | 8.23 | 8.16 | 8.3 | 1997 |
| Ukraine | 7.79 | 7.73 | 7.86 | 1998 |
| Ukraine | 7.82 | 7.75 | 7.89 | 1999 |
| Ukraine | 7.87 | 7.8 | 7.94 | 2000 |
| Ukraine | 7.83 | 7.76 | 7.89 | 2001 |
| Ukraine | 7.94 | 7.87 | 8.01 | 2002 |
| Ukraine | 8.08 | 8.01 | 8.15 | 2003 |
| Ukraine | 8.47 | 8.4 | 8.54 | 2004 |
| Ukraine | 8.92 | 8.85 | 9 | 2005 |
| Ukraine | 9.03 | 8.95 | 9.1 | 2006 |
| Ukraine | 9.42 | 9.35 | 9.5 | 2007 |
| Ukraine | 9.57 | 9.5 | 9.65 | 2008 |
| Ukraine | 9.12 | 9.04 | 9.19 | 2009 |
| Ukraine | 8.93 | 8.86 | 9 | 2010 |
| Ukraine | 8.81 | 8.74 | 8.89 | 2011 |
| Ukraine | 8.84 | 8.77 | 8.92 | 2012 |
| Ukraine | 8.73 | 8.66 | 8.8 | 2013 |
| Ukraine | 8.74 | 8.66 | 8.81 | 2014 |
| Ukraine | 10.62 | 10.54 | 10.7 | 2015 |
| Ukraine | 10.61 | 10.53 | 10.69 | 2016 |
| Ukraine | 9.88 | 9.8 | 9.96 | 2017 |
| Ukraine | 9.2 | 8.75 | 9.65 | 2018 |
| Ukraine | 8.56 | 7.65 | 9.47 | 2019 |
| Ukraine | 8 | 6.59 | 9.4 | 2020 |
| Ukraine | 7.46 | 5.56 | 9.37 | 2021 |
| Ukraine | 6.96 | 4.57 | 9.36 | 2022 |
| Ukraine | 6.5 | 3.63 | 9.37 | 2023 |
| Ukraine | 6.06 | 2.75 | 9.38 | 2024 |
| Ukraine | 5.67 | 1.93 | 9.41 | 2025 |
| Ukraine | 5.3 | 1.17 | 9.42 | 2026 |
| Ukraine | 4.95 | 0.48 | 9.42 | 2027 |
| Ukraine | 4.62 | -0.16 | 9.4 | 2028 |
| Ukraine | 4.32 | -0.75 | 9.38 | 2029 |
| Ukraine | 4.05 | -1.28 | 9.38 | 2030 |
| United Arab Emirates | 3.1 | 2.86 | 3.33 | 1990 |
| United Arab Emirates | 3.19 | 2.98 | 3.4 | 1991 |
| United Arab Emirates | 3.28 | 3.09 | 3.46 | 1992 |
| United Arab Emirates | 3.36 | 3.19 | 3.54 | 1993 |
| United Arab Emirates | 3.45 | 3.29 | 3.61 | 1994 |
| United Arab Emirates | 3.54 | 3.39 | 3.69 | 1995 |
| United Arab Emirates | 3.62 | 3.47 | 3.77 | 1996 |
| United Arab Emirates | 3.7 | 3.56 | 3.84 | 1997 |
| United Arab Emirates | 3.77 | 3.63 | 3.91 | 1998 |
| United Arab Emirates | 3.84 | 3.7 | 3.97 | 1999 |
| United Arab Emirates | 3.89 | 3.76 | 4.02 | 2000 |
| United Arab Emirates | 3.93 | 3.8 | 4.06 | 2001 |
| United Arab Emirates | 3.96 | 3.83 | 4.08 | 2002 |
| United Arab Emirates | 3.97 | 3.85 | 4.09 | 2003 |
| United Arab Emirates | 3.98 | 3.86 | 4.09 | 2004 |
| United Arab Emirates | 3.98 | 3.87 | 4.09 | 2005 |
| United Arab Emirates | 3.98 | 3.87 | 4.09 | 2006 |
| United Arab Emirates | 3.99 | 3.89 | 4.1 | 2007 |
| United Arab Emirates | 4.02 | 3.92 | 4.12 | 2008 |
| United Arab Emirates | 4.06 | 3.96 | 4.16 | 2009 |
| United Arab Emirates | 4.13 | 4.03 | 4.23 | 2010 |
| United Arab Emirates | 4.21 | 4.11 | 4.31 | 2011 |
| United Arab Emirates | 4.32 | 4.22 | 4.42 | 2012 |
| United Arab Emirates | 4.45 | 4.34 | 4.55 | 2013 |
| United Arab Emirates | 4.59 | 4.49 | 4.7 | 2014 |
| United Arab Emirates | 4.75 | 4.63 | 4.86 | 2015 |
| United Arab Emirates | 4.91 | 4.78 | 5.04 | 2016 |
| United Arab Emirates | 5.08 | 4.92 | 5.24 | 2017 |
| United Arab Emirates | 5.26 | 5.05 | 5.46 | 2018 |
| United Arab Emirates | 5.44 | 5.17 | 5.71 | 2019 |
| United Arab Emirates | 5.63 | 5.28 | 5.98 | 2020 |
| United Arab Emirates | 5.82 | 5.38 | 6.27 | 2021 |
| United Arab Emirates | 6.03 | 5.48 | 6.59 | 2022 |
| United Arab Emirates | 6.25 | 5.57 | 6.93 | 2023 |
| United Arab Emirates | 6.48 | 5.65 | 7.31 | 2024 |
| United Arab Emirates | 6.72 | 5.73 | 7.71 | 2025 |
| United Arab Emirates | 6.97 | 5.8 | 8.14 | 2026 |
| United Arab Emirates | 7.24 | 5.87 | 8.6 | 2027 |
| United Arab Emirates | 7.51 | 5.93 | 9.1 | 2028 |
| United Arab Emirates | 7.8 | 5.98 | 9.63 | 2029 |
| United Arab Emirates | 8.11 | 6.02 | 10.19 | 2030 |
| United Kingdom | 7.35 | 7.3 | 7.4 | 1990 |
| United Kingdom | 7.46 | 7.43 | 7.5 | 1991 |
| United Kingdom | 7.58 | 7.55 | 7.61 | 1992 |
| United Kingdom | 7.7 | 7.67 | 7.73 | 1993 |
| United Kingdom | 7.81 | 7.78 | 7.85 | 1994 |
| United Kingdom | 7.92 | 7.89 | 7.95 | 1995 |
| United Kingdom | 8.02 | 7.99 | 8.05 | 1996 |
| United Kingdom | 8.11 | 8.08 | 8.15 | 1997 |
| United Kingdom | 8.22 | 8.18 | 8.25 | 1998 |
| United Kingdom | 8.31 | 8.28 | 8.35 | 1999 |
| United Kingdom | 8.42 | 8.38 | 8.45 | 2000 |
| United Kingdom | 8.53 | 8.49 | 8.56 | 2001 |
| United Kingdom | 8.64 | 8.61 | 8.67 | 2002 |
| United Kingdom | 8.75 | 8.72 | 8.79 | 2003 |
| United Kingdom | 8.85 | 8.82 | 8.88 | 2004 |
| United Kingdom | 8.94 | 8.9 | 8.97 | 2005 |
| United Kingdom | 9.01 | 8.98 | 9.05 | 2006 |
| United Kingdom | 9.07 | 9.04 | 9.11 | 2007 |
| United Kingdom | 9.12 | 9.09 | 9.16 | 2008 |
| United Kingdom | 9.16 | 9.12 | 9.19 | 2009 |
| United Kingdom | 9.18 | 9.15 | 9.21 | 2010 |
| United Kingdom | 9.2 | 9.17 | 9.24 | 2011 |
| United Kingdom | 9.23 | 9.2 | 9.26 | 2012 |
| United Kingdom | 9.27 | 9.24 | 9.3 | 2013 |
| United Kingdom | 9.31 | 9.27 | 9.34 | 2014 |
| United Kingdom | 9.35 | 9.32 | 9.39 | 2015 |
| United Kingdom | 9.41 | 9.37 | 9.44 | 2016 |
| United Kingdom | 9.46 | 9.41 | 9.51 | 2017 |
| United Kingdom | 9.51 | 9.43 | 9.59 | 2018 |
| United Kingdom | 9.57 | 9.45 | 9.69 | 2019 |
| United Kingdom | 9.63 | 9.46 | 9.8 | 2020 |
| United Kingdom | 9.69 | 9.46 | 9.92 | 2021 |
| United Kingdom | 9.76 | 9.47 | 10.05 | 2022 |
| United Kingdom | 9.83 | 9.47 | 10.19 | 2023 |
| United Kingdom | 9.9 | 9.46 | 10.33 | 2024 |
| United Kingdom | 9.97 | 9.46 | 10.49 | 2025 |
| United Kingdom | 10.05 | 9.45 | 10.65 | 2026 |
| United Kingdom | 10.13 | 9.44 | 10.82 | 2027 |
| United Kingdom | 10.22 | 9.43 | 11.01 | 2028 |
| United Kingdom | 10.31 | 9.42 | 11.2 | 2029 |
| United Kingdom | 10.4 | 9.4 | 11.4 | 2030 |
| United States | 12.61 | 12.57 | 12.65 | 1990 |
| United States | 12.79 | 12.76 | 12.82 | 1991 |
| United States | 12.97 | 12.94 | 13 | 1992 |
| United States | 13.17 | 13.14 | 13.2 | 1993 |
| United States | 13.32 | 13.29 | 13.35 | 1994 |
| United States | 13.41 | 13.38 | 13.44 | 1995 |
| United States | 13.4 | 13.37 | 13.43 | 1996 |
| United States | 13.34 | 13.31 | 13.37 | 1997 |
| United States | 13.27 | 13.24 | 13.3 | 1998 |
| United States | 13.21 | 13.18 | 13.24 | 1999 |
| United States | 13.22 | 13.19 | 13.25 | 2000 |
| United States | 13.27 | 13.24 | 13.3 | 2001 |
| United States | 13.31 | 13.29 | 13.34 | 2002 |
| United States | 13.31 | 13.28 | 13.34 | 2003 |
| United States | 13.25 | 13.22 | 13.27 | 2004 |
| United States | 13.2 | 13.17 | 13.23 | 2005 |
| United States | 13.11 | 13.08 | 13.14 | 2006 |
| United States | 13.03 | 13 | 13.06 | 2007 |
| United States | 12.98 | 12.95 | 13 | 2008 |
| United States | 12.9 | 12.87 | 12.92 | 2009 |
| United States | 12.79 | 12.76 | 12.81 | 2010 |
| United States | 12.7 | 12.67 | 12.72 | 2011 |
| United States | 12.6 | 12.58 | 12.63 | 2012 |
| United States | 12.55 | 12.52 | 12.57 | 2013 |
| United States | 12.54 | 12.51 | 12.56 | 2014 |
| United States | 12.62 | 12.6 | 12.65 | 2015 |
| United States | 12.79 | 12.76 | 12.82 | 2016 |
| United States | 12.94 | 12.9 | 12.97 | 2017 |
| United States | 13.08 | 13 | 13.16 | 2018 |
| United States | 13.22 | 13.07 | 13.37 | 2019 |
| United States | 13.39 | 13.15 | 13.62 | 2020 |
| United States | 13.57 | 13.23 | 13.9 | 2021 |
| United States | 13.73 | 13.29 | 14.18 | 2022 |
| United States | 13.9 | 13.33 | 14.47 | 2023 |
| United States | 14.07 | 13.36 | 14.77 | 2024 |
| United States | 14.25 | 13.4 | 15.1 | 2025 |
| United States | 14.44 | 13.43 | 15.46 | 2026 |
| United States | 14.62 | 13.44 | 15.81 | 2027 |
| United States | 14.8 | 13.43 | 16.16 | 2028 |
| United States | 14.98 | 13.42 | 16.54 | 2029 |
| United States | 15.2 | 13.43 | 16.97 | 2030 |
| Uruguay | 14.74 | 14.45 | 15.03 | 1990 |
| Uruguay | 14.88 | 14.65 | 15.1 | 1991 |
| Uruguay | 15.02 | 14.83 | 15.21 | 1992 |
| Uruguay | 15.14 | 14.96 | 15.31 | 1993 |
| Uruguay | 15.2 | 15.02 | 15.37 | 1994 |
| Uruguay | 15.18 | 15.01 | 15.36 | 1995 |
| Uruguay | 15.1 | 14.93 | 15.28 | 1996 |
| Uruguay | 14.97 | 14.8 | 15.14 | 1997 |
| Uruguay | 14.81 | 14.65 | 14.98 | 1998 |
| Uruguay | 14.66 | 14.49 | 14.82 | 1999 |
| Uruguay | 14.51 | 14.35 | 14.67 | 2000 |
| Uruguay | 14.39 | 14.23 | 14.55 | 2001 |
| Uruguay | 14.31 | 14.15 | 14.46 | 2002 |
| Uruguay | 14.26 | 14.1 | 14.42 | 2003 |
| Uruguay | 14.26 | 14.1 | 14.42 | 2004 |
| Uruguay | 14.3 | 14.14 | 14.46 | 2005 |
| Uruguay | 14.38 | 14.22 | 14.54 | 2006 |
| Uruguay | 14.51 | 14.35 | 14.67 | 2007 |
| Uruguay | 14.66 | 14.5 | 14.82 | 2008 |
| Uruguay | 14.83 | 14.67 | 14.99 | 2009 |
| Uruguay | 15.01 | 14.85 | 15.17 | 2010 |
| Uruguay | 15.2 | 15.04 | 15.37 | 2011 |
| Uruguay | 15.39 | 15.22 | 15.55 | 2012 |
| Uruguay | 15.56 | 15.39 | 15.73 | 2013 |
| Uruguay | 15.72 | 15.55 | 15.89 | 2014 |
| Uruguay | 15.87 | 15.69 | 16.05 | 2015 |
| Uruguay | 16.01 | 15.8 | 16.23 | 2016 |
| Uruguay | 16.15 | 15.87 | 16.43 | 2017 |
| Uruguay | 16.28 | 15.9 | 16.67 | 2018 |
| Uruguay | 16.42 | 15.89 | 16.94 | 2019 |
| Uruguay | 16.54 | 15.85 | 17.23 | 2020 |
| Uruguay | 16.67 | 15.79 | 17.54 | 2021 |
| Uruguay | 16.79 | 15.71 | 17.87 | 2022 |
| Uruguay | 16.91 | 15.61 | 18.22 | 2023 |
| Uruguay | 17.03 | 15.49 | 18.57 | 2024 |
| Uruguay | 17.15 | 15.35 | 18.95 | 2025 |
| Uruguay | 17.26 | 15.19 | 19.33 | 2026 |
| Uruguay | 17.38 | 15.02 | 19.73 | 2027 |
| Uruguay | 17.49 | 14.83 | 20.15 | 2028 |
| Uruguay | 17.6 | 14.62 | 20.57 | 2029 |
| Uruguay | 17.71 | 14.4 | 21.01 | 2030 |
| Uzbekistan | 3.17 | 3.1 | 3.23 | 1990 |
| Uzbekistan | 3.19 | 3.13 | 3.24 | 1991 |
| Uzbekistan | 3.21 | 3.16 | 3.25 | 1992 |
| Uzbekistan | 3.23 | 3.18 | 3.27 | 1993 |
| Uzbekistan | 3.24 | 3.21 | 3.28 | 1994 |
| Uzbekistan | 3.26 | 3.22 | 3.3 | 1995 |
| Uzbekistan | 3.28 | 3.24 | 3.32 | 1996 |
| Uzbekistan | 3.31 | 3.27 | 3.34 | 1997 |
| Uzbekistan | 3.34 | 3.3 | 3.38 | 1998 |
| Uzbekistan | 3.38 | 3.34 | 3.42 | 1999 |
| Uzbekistan | 3.43 | 3.39 | 3.47 | 2000 |
| Uzbekistan | 3.49 | 3.45 | 3.53 | 2001 |
| Uzbekistan | 3.57 | 3.53 | 3.61 | 2002 |
| Uzbekistan | 3.65 | 3.61 | 3.69 | 2003 |
| Uzbekistan | 3.75 | 3.71 | 3.78 | 2004 |
| Uzbekistan | 3.84 | 3.8 | 3.88 | 2005 |
| Uzbekistan | 3.93 | 3.9 | 3.97 | 2006 |
| Uzbekistan | 4.02 | 3.98 | 4.06 | 2007 |
| Uzbekistan | 4.1 | 4.06 | 4.14 | 2008 |
| Uzbekistan | 4.16 | 4.12 | 4.2 | 2009 |
| Uzbekistan | 4.22 | 4.18 | 4.26 | 2010 |
| Uzbekistan | 4.26 | 4.22 | 4.3 | 2011 |
| Uzbekistan | 4.3 | 4.26 | 4.34 | 2012 |
| Uzbekistan | 4.34 | 4.3 | 4.38 | 2013 |
| Uzbekistan | 4.37 | 4.33 | 4.41 | 2014 |
| Uzbekistan | 4.4 | 4.36 | 4.44 | 2015 |
| Uzbekistan | 4.43 | 4.38 | 4.47 | 2016 |
| Uzbekistan | 4.45 | 4.39 | 4.51 | 2017 |
| Uzbekistan | 4.47 | 4.39 | 4.56 | 2018 |
| Uzbekistan | 4.5 | 4.38 | 4.62 | 2019 |
| Uzbekistan | 4.52 | 4.36 | 4.68 | 2020 |
| Uzbekistan | 4.55 | 4.35 | 4.76 | 2021 |
| Uzbekistan | 4.58 | 4.32 | 4.83 | 2022 |
| Uzbekistan | 4.6 | 4.3 | 4.91 | 2023 |
| Uzbekistan | 4.63 | 4.27 | 5 | 2024 |
| Uzbekistan | 4.66 | 4.24 | 5.09 | 2025 |
| Uzbekistan | 4.69 | 4.2 | 5.19 | 2026 |
| Uzbekistan | 4.73 | 4.16 | 5.29 | 2027 |
| Uzbekistan | 4.76 | 4.12 | 5.39 | 2028 |
| Uzbekistan | 4.79 | 4.08 | 5.5 | 2029 |
| Uzbekistan | 4.83 | 4.03 | 5.62 | 2030 |
| Venezuela | 7.16 | 7.03 | 7.29 | 1990 |
| Venezuela | 7.06 | 6.97 | 7.15 | 1991 |
| Venezuela | 6.96 | 6.88 | 7.05 | 1992 |
| Venezuela | 6.82 | 6.74 | 6.9 | 1993 |
| Venezuela | 6.59 | 6.51 | 6.67 | 1994 |
| Venezuela | 6.28 | 6.2 | 6.35 | 1995 |
| Venezuela | 5.93 | 5.86 | 6 | 1996 |
| Venezuela | 5.63 | 5.56 | 5.7 | 1997 |
| Venezuela | 5.43 | 5.36 | 5.49 | 1998 |
| Venezuela | 5.33 | 5.27 | 5.4 | 1999 |
| Venezuela | 5.34 | 5.28 | 5.4 | 2000 |
| Venezuela | 5.4 | 5.34 | 5.46 | 2001 |
| Venezuela | 5.46 | 5.4 | 5.52 | 2002 |
| Venezuela | 5.51 | 5.45 | 5.57 | 2003 |
| Venezuela | 5.54 | 5.48 | 5.6 | 2004 |
| Venezuela | 5.62 | 5.56 | 5.68 | 2005 |
| Venezuela | 5.76 | 5.7 | 5.82 | 2006 |
| Venezuela | 5.94 | 5.88 | 6 | 2007 |
| Venezuela | 6.12 | 6.06 | 6.18 | 2008 |
| Venezuela | 6.26 | 6.2 | 6.32 | 2009 |
| Venezuela | 6.39 | 6.33 | 6.45 | 2010 |
| Venezuela | 6.52 | 6.46 | 6.58 | 2011 |
| Venezuela | 6.67 | 6.61 | 6.73 | 2012 |
| Venezuela | 6.81 | 6.74 | 6.87 | 2013 |
| Venezuela | 6.92 | 6.85 | 6.98 | 2014 |
| Venezuela | 7 | 6.94 | 7.07 | 2015 |
| Venezuela | 7.07 | 7.01 | 7.14 | 2016 |
| Venezuela | 7.14 | 7.05 | 7.23 | 2017 |
| Venezuela | 7.2 | 7.03 | 7.37 | 2018 |
| Venezuela | 7.26 | 6.98 | 7.55 | 2019 |
| Venezuela | 7.32 | 6.9 | 7.75 | 2020 |
| Venezuela | 7.38 | 6.8 | 7.97 | 2021 |
| Venezuela | 7.44 | 6.67 | 8.21 | 2022 |
| Venezuela | 7.5 | 6.53 | 8.47 | 2023 |
| Venezuela | 7.55 | 6.37 | 8.73 | 2024 |
| Venezuela | 7.6 | 6.19 | 9.02 | 2025 |
| Venezuela | 7.66 | 5.99 | 9.32 | 2026 |
| Venezuela | 7.71 | 5.78 | 9.63 | 2027 |
| Venezuela | 7.76 | 5.56 | 9.96 | 2028 |
| Venezuela | 7.81 | 5.31 | 10.3 | 2029 |
| Venezuela | 7.85 | 5.06 | 10.65 | 2030 |
| Vietnam | 1.69 | 1.66 | 1.71 | 1990 |
| Vietnam | 1.69 | 1.67 | 1.71 | 1991 |
| Vietnam | 1.7 | 1.68 | 1.71 | 1992 |
| Vietnam | 1.7 | 1.68 | 1.71 | 1993 |
| Vietnam | 1.7 | 1.69 | 1.72 | 1994 |
| Vietnam | 1.71 | 1.69 | 1.72 | 1995 |
| Vietnam | 1.71 | 1.7 | 1.73 | 1996 |
| Vietnam | 1.72 | 1.71 | 1.74 | 1997 |
| Vietnam | 1.74 | 1.72 | 1.75 | 1998 |
| Vietnam | 1.75 | 1.73 | 1.76 | 1999 |
| Vietnam | 1.77 | 1.75 | 1.78 | 2000 |
| Vietnam | 1.78 | 1.77 | 1.8 | 2001 |
| Vietnam | 1.8 | 1.79 | 1.82 | 2002 |
| Vietnam | 1.83 | 1.81 | 1.84 | 2003 |
| Vietnam | 1.85 | 1.83 | 1.86 | 2004 |
| Vietnam | 1.87 | 1.85 | 1.88 | 2005 |
| Vietnam | 1.89 | 1.87 | 1.9 | 2006 |
| Vietnam | 1.9 | 1.89 | 1.92 | 2007 |
| Vietnam | 1.92 | 1.9 | 1.93 | 2008 |
| Vietnam | 1.93 | 1.92 | 1.94 | 2009 |
| Vietnam | 1.94 | 1.93 | 1.95 | 2010 |
| Vietnam | 1.95 | 1.94 | 1.96 | 2011 |
| Vietnam | 1.96 | 1.95 | 1.97 | 2012 |
| Vietnam | 1.97 | 1.96 | 1.98 | 2013 |
| Vietnam | 1.98 | 1.97 | 1.99 | 2014 |
| Vietnam | 1.99 | 1.97 | 2 | 2015 |
| Vietnam | 2 | 1.98 | 2.01 | 2016 |
| Vietnam | 2.01 | 1.98 | 2.03 | 2017 |
| Vietnam | 2.01 | 1.98 | 2.05 | 2018 |
| Vietnam | 2.02 | 1.98 | 2.07 | 2019 |
| Vietnam | 2.03 | 1.98 | 2.09 | 2020 |
| Vietnam | 2.04 | 1.97 | 2.11 | 2021 |
| Vietnam | 2.05 | 1.96 | 2.14 | 2022 |
| Vietnam | 2.06 | 1.95 | 2.16 | 2023 |
| Vietnam | 2.07 | 1.94 | 2.19 | 2024 |
| Vietnam | 2.07 | 1.93 | 2.22 | 2025 |
| Vietnam | 2.08 | 1.91 | 2.25 | 2026 |
| Vietnam | 2.09 | 1.9 | 2.28 | 2027 |
| Vietnam | 2.1 | 1.88 | 2.32 | 2028 |
| Vietnam | 2.11 | 1.86 | 2.35 | 2029 |
| Vietnam | 2.12 | 1.85 | 2.39 | 2030 |
| Yemen | 1.59 | 1.53 | 1.65 | 1990 |
| Yemen | 1.58 | 1.53 | 1.63 | 1991 |
| Yemen | 1.58 | 1.53 | 1.62 | 1992 |
| Yemen | 1.57 | 1.53 | 1.61 | 1993 |
| Yemen | 1.57 | 1.53 | 1.6 | 1994 |
| Yemen | 1.57 | 1.54 | 1.6 | 1995 |
| Yemen | 1.58 | 1.55 | 1.61 | 1996 |
| Yemen | 1.59 | 1.56 | 1.62 | 1997 |
| Yemen | 1.61 | 1.58 | 1.64 | 1998 |
| Yemen | 1.64 | 1.61 | 1.67 | 1999 |
| Yemen | 1.67 | 1.64 | 1.7 | 2000 |
| Yemen | 1.71 | 1.68 | 1.74 | 2001 |
| Yemen | 1.75 | 1.72 | 1.78 | 2002 |
| Yemen | 1.8 | 1.77 | 1.83 | 2003 |
| Yemen | 1.85 | 1.82 | 1.88 | 2004 |
| Yemen | 1.89 | 1.86 | 1.93 | 2005 |
| Yemen | 1.94 | 1.91 | 1.97 | 2006 |
| Yemen | 1.99 | 1.96 | 2.02 | 2007 |
| Yemen | 2.04 | 2 | 2.07 | 2008 |
| Yemen | 2.08 | 2.04 | 2.11 | 2009 |
| Yemen | 2.11 | 2.08 | 2.15 | 2010 |
| Yemen | 2.15 | 2.11 | 2.18 | 2011 |
| Yemen | 2.18 | 2.14 | 2.21 | 2012 |
| Yemen | 2.21 | 2.17 | 2.24 | 2013 |
| Yemen | 2.24 | 2.2 | 2.27 | 2014 |
| Yemen | 2.27 | 2.23 | 2.3 | 2015 |
| Yemen | 2.29 | 2.25 | 2.34 | 2016 |
| Yemen | 2.32 | 2.26 | 2.37 | 2017 |
| Yemen | 2.34 | 2.27 | 2.42 | 2018 |
| Yemen | 2.37 | 2.28 | 2.47 | 2019 |
| Yemen | 2.4 | 2.28 | 2.52 | 2020 |
| Yemen | 2.42 | 2.27 | 2.57 | 2021 |
| Yemen | 2.45 | 2.26 | 2.63 | 2022 |
| Yemen | 2.47 | 2.25 | 2.7 | 2023 |
| Yemen | 2.5 | 2.24 | 2.76 | 2024 |
| Yemen | 2.53 | 2.22 | 2.83 | 2025 |
| Yemen | 2.55 | 2.2 | 2.9 | 2026 |
| Yemen | 2.58 | 2.18 | 2.98 | 2027 |
| Yemen | 2.6 | 2.16 | 3.05 | 2028 |
| Yemen | 2.63 | 2.13 | 3.13 | 2029 |
| Yemen | 2.66 | 2.1 | 3.22 | 2030 |
| Zambia | 2.88 | 2.78 | 2.97 | 1990 |
| Zambia | 2.87 | 2.8 | 2.95 | 1991 |
| Zambia | 2.87 | 2.8 | 2.93 | 1992 |
| Zambia | 2.86 | 2.8 | 2.92 | 1993 |
| Zambia | 2.84 | 2.78 | 2.9 | 1994 |
| Zambia | 2.82 | 2.76 | 2.87 | 1995 |
| Zambia | 2.79 | 2.73 | 2.84 | 1996 |
| Zambia | 2.75 | 2.7 | 2.8 | 1997 |
| Zambia | 2.71 | 2.66 | 2.76 | 1998 |
| Zambia | 2.67 | 2.63 | 2.72 | 1999 |
| Zambia | 2.64 | 2.6 | 2.69 | 2000 |
| Zambia | 2.62 | 2.57 | 2.66 | 2001 |
| Zambia | 2.61 | 2.56 | 2.65 | 2002 |
| Zambia | 2.61 | 2.56 | 2.65 | 2003 |
| Zambia | 2.62 | 2.58 | 2.67 | 2004 |
| Zambia | 2.65 | 2.6 | 2.69 | 2005 |
| Zambia | 2.68 | 2.63 | 2.72 | 2006 |
| Zambia | 2.71 | 2.67 | 2.76 | 2007 |
| Zambia | 2.75 | 2.7 | 2.79 | 2008 |
| Zambia | 2.78 | 2.74 | 2.83 | 2009 |
| Zambia | 2.81 | 2.77 | 2.86 | 2010 |
| Zambia | 2.84 | 2.8 | 2.89 | 2011 |
| Zambia | 2.87 | 2.83 | 2.92 | 2012 |
| Zambia | 2.9 | 2.85 | 2.95 | 2013 |
| Zambia | 2.93 | 2.88 | 2.98 | 2014 |
| Zambia | 2.96 | 2.9 | 3.01 | 2015 |
| Zambia | 2.99 | 2.92 | 3.05 | 2016 |
| Zambia | 3.01 | 2.93 | 3.09 | 2017 |
| Zambia | 3.04 | 2.94 | 3.14 | 2018 |
| Zambia | 3.07 | 2.94 | 3.2 | 2019 |
| Zambia | 3.09 | 2.93 | 3.26 | 2020 |
| Zambia | 3.12 | 2.92 | 3.32 | 2021 |
| Zambia | 3.15 | 2.9 | 3.39 | 2022 |
| Zambia | 3.17 | 2.88 | 3.46 | 2023 |
| Zambia | 3.2 | 2.86 | 3.54 | 2024 |
| Zambia | 3.22 | 2.83 | 3.62 | 2025 |
| Zambia | 3.25 | 2.79 | 3.7 | 2026 |
| Zambia | 3.27 | 2.76 | 3.78 | 2027 |
| Zambia | 3.29 | 2.72 | 3.87 | 2028 |
| Zambia | 3.32 | 2.68 | 3.96 | 2029 |
| Zambia | 3.34 | 2.63 | 4.05 | 2030 |
| Zimbabwe | 1.54 | 1.47 | 1.61 | 1990 |
| Zimbabwe | 1.59 | 1.53 | 1.65 | 1991 |
| Zimbabwe | 1.65 | 1.6 | 1.7 | 1992 |
| Zimbabwe | 1.71 | 1.67 | 1.76 | 1993 |
| Zimbabwe | 1.78 | 1.74 | 1.83 | 1994 |
| Zimbabwe | 1.86 | 1.81 | 1.91 | 1995 |
| Zimbabwe | 1.94 | 1.9 | 1.99 | 1996 |
| Zimbabwe | 2.03 | 1.99 | 2.08 | 1997 |
| Zimbabwe | 2.13 | 2.08 | 2.18 | 1998 |
| Zimbabwe | 2.23 | 2.18 | 2.28 | 1999 |
| Zimbabwe | 2.33 | 2.28 | 2.38 | 2000 |
| Zimbabwe | 2.43 | 2.38 | 2.48 | 2001 |
| Zimbabwe | 2.51 | 2.45 | 2.56 | 2002 |
| Zimbabwe | 2.57 | 2.52 | 2.62 | 2003 |
| Zimbabwe | 2.61 | 2.56 | 2.66 | 2004 |
| Zimbabwe | 2.63 | 2.57 | 2.68 | 2005 |
| Zimbabwe | 2.62 | 2.57 | 2.68 | 2006 |
| Zimbabwe | 2.61 | 2.55 | 2.66 | 2007 |
| Zimbabwe | 2.57 | 2.52 | 2.62 | 2008 |
| Zimbabwe | 2.53 | 2.48 | 2.58 | 2009 |
| Zimbabwe | 2.49 | 2.44 | 2.54 | 2010 |
| Zimbabwe | 2.44 | 2.39 | 2.49 | 2011 |
| Zimbabwe | 2.39 | 2.35 | 2.44 | 2012 |
| Zimbabwe | 2.35 | 2.3 | 2.4 | 2013 |
| Zimbabwe | 2.31 | 2.27 | 2.36 | 2014 |
| Zimbabwe | 2.28 | 2.23 | 2.33 | 2015 |
| Zimbabwe | 2.25 | 2.19 | 2.3 | 2016 |
| Zimbabwe | 2.21 | 2.14 | 2.29 | 2017 |
| Zimbabwe | 2.18 | 2.09 | 2.28 | 2018 |
| Zimbabwe | 2.15 | 2.03 | 2.27 | 2019 |
| Zimbabwe | 2.12 | 1.96 | 2.27 | 2020 |
| Zimbabwe | 2.09 | 1.89 | 2.28 | 2021 |
| Zimbabwe | 2.05 | 1.82 | 2.28 | 2022 |
| Zimbabwe | 2.02 | 1.75 | 2.29 | 2023 |
| Zimbabwe | 1.99 | 1.68 | 2.3 | 2024 |
| Zimbabwe | 1.96 | 1.6 | 2.31 | 2025 |
| Zimbabwe | 1.93 | 1.53 | 2.32 | 2026 |
| Zimbabwe | 1.89 | 1.45 | 2.33 | 2027 |
| Zimbabwe | 1.86 | 1.38 | 2.35 | 2028 |
| Zimbabwe | 1.83 | 1.3 | 2.36 | 2029 |
| Zimbabwe | 1.8 | 1.23 | 2.37 | 2030 |
